# A novel sulfamoylphenyl-dihydro-thiadiazole derivative as a dual EGFR and carbonic anhydrase inhibitor for cancer therapy

**DOI:** 10.1371/journal.pone.0328305

**Published:** 2025-09-04

**Authors:** Ibrahim H. Eissa, Hazem Elkady, Walid E. Elgammal, Hazem A. Mahdy, Dalal Z. Husein, Fatma G. Amin, Asmaa A. A. Elsheshiny, Shimaa. S. Abdelfattah, Aisha A. Alsfouk, Eslam B. Elkaeed, Ahmed M. Metwaly

**Affiliations:** 1 Pharmaceutical Medicinal Chemistry & Drug Design Department, Faculty of Pharmacy (Boys), Al-Azhar University, Cairo, Egypt; 2 Department of Chemistry, Faculty of Science, Al-Azhar University, Nasr City, Cairo, Egypt; 3 Chemistry Department, Faculty of Science, New Valley University, El-Kharja, Egypt; 4 Physics Department, Faculty of Science, Alexandria University, Alexandria, Egypt; 5 Biophysics Branch, Physics Department, Faculty of Science (girls), Al-Azhar University, Nasr City, Cairo, Egypt; 6 Physics Department, Faculty of Science, Al-Azhar University (Girl’s Branch), Cairo, Egypt; 7 Department of Pharmaceutical Sciences, College of Pharmacy, Princess Nourah bint Abdulrahman University, Riyadh, Saudi Arabia; 8 Department of Pharmaceutical Sciences, College of Pharmacy, AlMaarefa University, Riyadh, Saudi Arabia; 9 Pharmacognosy and Medicinal Plants Department, Faculty of Pharmacy (Boys), Al-Azhar University, Cairo, Egypt; Vignan Pharmacy College, INDIA

## Abstract

A novel sulfamoylphenyl-dihydro-thiadiazole derivative (compound **14**) has been designed and synthesized as a dual inhibitor targeting EGFR and human carbonic anhydrases (hCA_IX and hCA_XII). Computational studies, including density functional theory (DFT), molecular docking, and molecular dynamics simulations, confirmed its stability, favorable binding interactions, and reactivity profiles. Compound **14** showed potent inhibition of EGFR (IC₅₀ = 10.12 ± 0.29 nM), hCA_IX and hCA_XII (IC₅₀ = 79 ± 1.2 nM and 58 ± 0.9 nM, respectively). Cytotoxicity assays demonstrated selective activity against cancer cells, with IC₅₀ values of 16.13 µM in MDA-MB-231 and 22.57 µM in MCF-7 cells, compared to 148.32 µM in non-cancerous Vero cells. Compared to acetazolamide, compound **14** exhibited improved selectivity for cancer cells. Apoptosis studies revealed significant cell death in MDA-MB-231 cells, with early and late apoptosis rates of 22.50% and 58.27%, respectively, alongside a marked G1-phase cell cycle arrest (49.10% in treated cells vs. 44.98% in controls). *In silico* toxicological evaluations indicated a favorable safety profile, with low irritancy and acceptable rat oral LD₅₀ (15.81 mg/kg) and carcinogenic potency (TD₅₀ = 36.95). Compound **14**’s potent dual inhibition and selective cytotoxicity make it a promising candidate for further optimization and *in vivo* studies.

## 1. Introduction

Cancer remains one of the most significant global health challenges [1], with its complexity arising from factors such as tumor heterogeneity, adaptive resistance, and the interplay of multiple signaling pathways [2]. Several strategies have been employed to combat cancer, including the inhibition of inflammation [3–5], genetic modification [6,7], overcoming drug resistance [8,9], and targeting specific proteins [10]. To address these challenges, there is a growing focus on developing multitargeted therapeutic agents capable of simultaneously modulating key cancer-promoting pathways [11,12]. Such strategies aim to enhance treatment efficacy while minimizing the risk of drug resistance [13]. Among these approaches, dual inhibition of epidermal growth factor receptor (EGFR) and tumor-associated human carbonic anhydrases (hCA_IX and hCA_XII) has emerged as a promising direction due to their critical roles in cancer progression.

EGFR is a well-established therapeutic target in oncology, as its dysregulation drives tumor proliferation, survival, and metastasis across various cancer types [14,15]. Meanwhile, hCA_IX and hCA_XII are overexpressed in hypoxic tumor environments and play vital roles in maintaining an acidic microenvironment that promotes cancer cell survival, metastasis, and resistance to conventional therapies [16,17]. Despite the availability of targeted therapies for EGFR, hCA_IX and hCA_XII, their single-target focus often limits therapeutic efficacy, as cancer cells can activate compensatory mechanisms to evade treatment [18]. This highlights the potential of compounds capable of dual inhibition, offering a more comprehensive therapeutic strategy against cancer.

Thiadiazoles have demonstrated a broad spectrum of anticancer effects, including the ability to inhibit key molecular targets such as kinases [19,20] that are dysregulated in cancer cells. Their efficacy has been particularly noted in targeting pathways involved in cell proliferation, angiogenesis, and apoptosis [21,22]. For example, several thiadiazole derivatives exhibit potent inhibition of receptor tyrosine kinases like EGFR, which is crucial in many aggressive cancers. Additionally, these compounds often target metabolic enzymes, such as hCA_IX and hCA_XII, which facilitate the acidic tumor microenvironment and support tumor survival and metastasis. The structural flexibility of thiadiazoles allows for modifications that enhance their pharmacokinetic properties, target specificity, and therapeutic efficacy, making them a valuable scaffold in the development of novel anticancer agents.

Building upon the aforementioned findings and our ongoing efforts to discover novel targeted anticancer agents [23,24], particularly EGFR inhibitors [25–28], we designed, synthesized, and evaluated a new sulfamoylphenyl-dihydro-thiadiazole derivative, compound **14**, as a dual inhibitor of EGFR, hCA_IX and hCA_XII. Computational methods, including density functional theory (DFT), molecular docking, and molecular dynamics (MD) simulations, revealed insights into its stability, reactivity, and binding interactions. Experimental validation confirmed its potent inhibitory activity, selective cytotoxicity toward cancer cells, and favorable toxicological profile. This work establishes compound **14** as a promising candidate for dual-target therapy and emphasizes the potential of multi-target drug design in addressing the challenges of cancer biology.

## 2.Rationale

**Fig 1:**
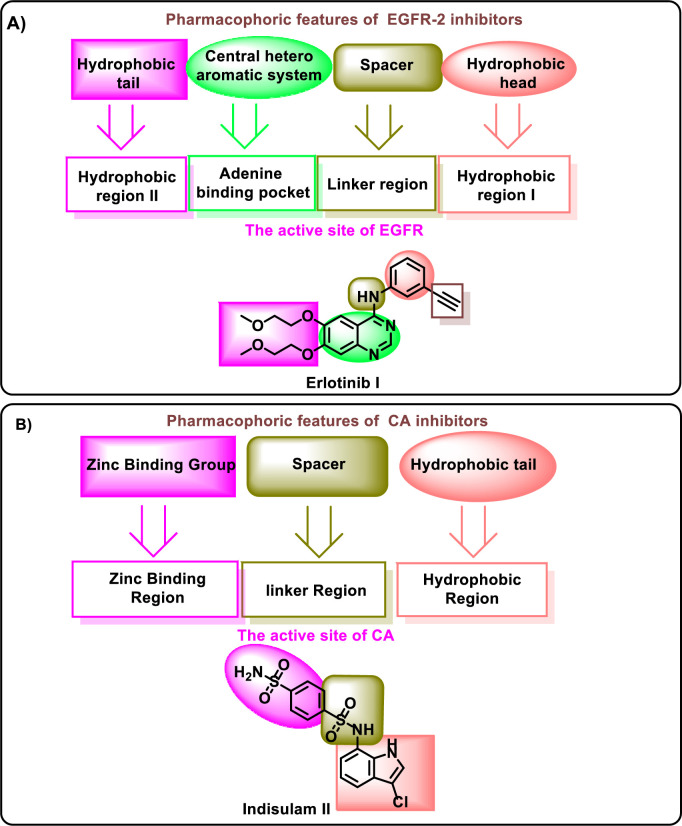
Pharmacophoric features for EGFRIs and CAIs.

There are four common pharmacophoric features for EGFRIs taking erlotinib **I** as an example: i) A flat hetero aromatic system to occupy the adenine binding pocket [29]. ii) A terminal hydrophobic head to occupy the hydrophobic region I [30]. iii) a linker NH group that can form important hydrogen bond interaction with amino acid residues in the linker region [31]. iv) A hydrophobic tail to occupy the hydrophobic region II [32,33]. On the other hand. CAIs have three common pharmacophoric features as appeared in indisulam **II**. i) Zinc-binding group (ZBGs) like sulfonamides which can interact with the zinc ion and H-bonds with the conserved residues in the zinc binding region. ii) A linker moiety to occupy the spacer region between the zinc-binding region and the hydrophobic region. iii) A hydrophobic tail which can occupy the hydrophobic pocket [34] (Fig 1).

Our research group discovered a pyrazole-thiazol-4-one hybrid (compound **III**) as a potent EGFR inhibitor (IC_50_ = 267 nM). In addition, it exhibited potent anti-proliferative actions against two cancer cell lines (T-47D and MDA-MB-231) with IC_50_ values of 0.62 and 3.14 μM, respectively. Worth noting, that compound **III** induced apoptosis in MDA-MB-231 cells, and resulted in a cell cycle arrest at the G2/M phase [35].

In this work, we used compound **III** as a lead compound to design a new cancer agent with dual activity against EGFR and CA. Compound **III** was selected as a lead structure based on its previously reported potent EGFR inhibitory activity (IC₅₀ = 267 nM) and significant anti-proliferative effects against T-47D and MDA-MB-231 breast cancer cell lines, with IC₅₀ values of 0.62 µM and 3.14 µM, respectively. Additionally, it demonstrated the ability to induce apoptosis and cause G2/M cell cycle arrest in MDA-MB-231 cells [35]. These promising biological results established compound **III** as a strong candidate for further optimization.

Moreover, compound **III**’s structural framework—featuring pyrazole and thiazolone moieties—provided a valuable basis for rational drug design. In developing the target compound **14**, we retained and enhanced key pharmacophoric features for EGFR inhibition while incorporating sulfonamide and thiadiazole functionalities to enable dual inhibition of both EGFR, hCA_IX and hCA_XII. This design strategy aimed to address tumor heterogeneity and resistance by targeting multiple oncogenic pathways.

**Fig 2:**
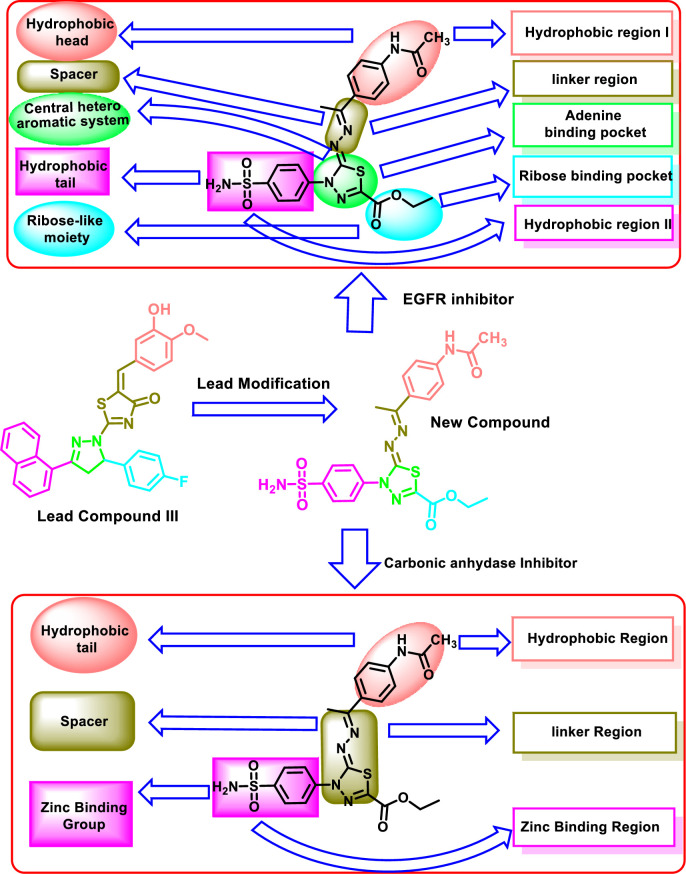
Rational of molecular design of dual EGFRIs and CAIs.

The design compound comprises 2,3-dihydro-1,3,4-thiadiazole and sulfonamide moieties as a core structure. In addition, the new compound catches the essential pharmacophoric features of both EGFRIs and CAIs as shown in Fig 2. For EGFR inhibition, the 2,3-dihydro-1,3,4-thiadiazole moiety acts as a central heterocyclic system to occupy the adenine binding pocket. The benzene sulfonamide moiety was utilized as a hydrophobic tail to occupy the hydrophobic pocket II. The ethylidene hydrazone moiety was utilized as a linker moiety. Moreover, the *N*-phenylacetamide moiety was utilized as a hydrophobic head to occupy the hydrophobic pocket I. The ester moiety at position 2 of 2,3-dihydro-1,3,4-thiadiazole moiety was incoprporated to occupy the ribose binding pocket of EGFR active site.

For hCA inhibition, the 2-(((*Z*)-ethylidene)hydrazono)-2,3-dihydro-1,3,4-thiadiazole moiety was utilized as a linker structure. The *N*-phenylacetamide moiety was utilized as a hydrophobic tail to occupy the hydrophobic region. The benzene sulfonamide moiety was utilized as a zinc-binding group to occupy the zinc-binding region.

The ester functionality in compound **14** may play a crucial role in multiple aspects of its pharmacological profile. i) The ester moiety can contribute to the overall molecular flexibility and assist in optimizing the spatial orientation of the compound within the active sites of EGFR, hCA_IX and hCA_XII, as confirmed by our docking and MD simulation studies. ii) The ester group may modulate the electronic distribution of the molecule, which influences key interactions with target residues, particularly through hydrogen bonding and van der Waals interactions in the active pocket. iii) The ester moiety can enhance lipophilicity, thereby potentially improving membrane permeability, which is critical for cellular uptake and bioactivity. iv) From a medicinal chemistry standpoint, the ester functionality could serve as a potential prodrug motif. It may undergo enzymatic hydrolysis *in vivo*, leading to a more polar carboxylic acid derivative that could have different activity or pharmacokinetics, offering a tunable approach for future optimization.

## 3.Results and discussions

### 3.1.Computational studies

#### 3.1.1.DFT and QTAIM studies.

**Fig 3:**
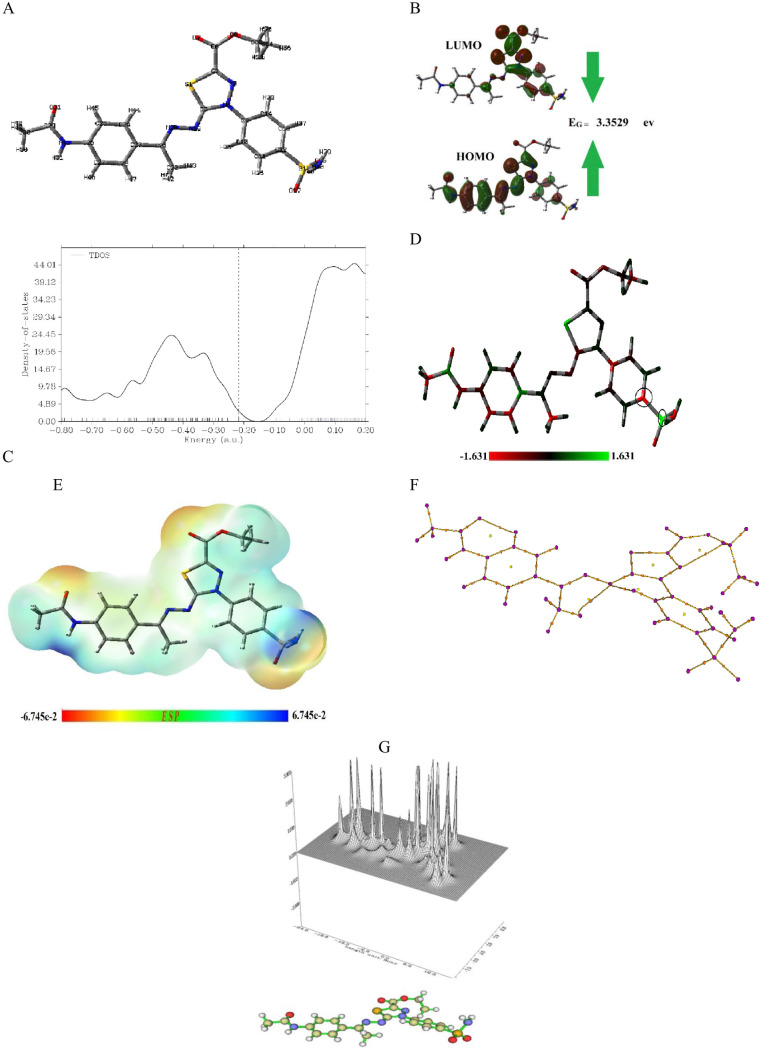
DFT and QTAIM parameters for compound 14. A: The optimized chemical structure, B: The optimized HOMO, LUMO, and energy gap, C: The TDOS, D: The Mulliken atomic distribution charge, E: The ESP, F: Bond paths and G: The relief map for electron density in a plan XY.

In this study, the DFT method is applied by Gaussian 09(D) software to optimize the geometry structure of compound **14** using the B3LYP computational approach which depends on a 6–31 + G(d,p) basis [36]. From optimization results, the quantum global parameters are calculated in Table 1. Additionally, the final structure of compound **14** after optimization is shown in Fig 3A with 56 atoms and 262 electrons, it seems that all molecular rings do not lie in one plan There is one terminal ring of benzene is perpendicular to the plane of the molecule, from optimization results the characteristic molecule parameters values are the total ground state energy (ET)is −62,980.37 ev with dipole moment (Dm) 3.9649 Debye which is measured the separation between opposite charge.

**Table 1:** The calculated global quantum parameters for compound 14.

Molecular parameter	Symbol	Value
Homo energy	E_HOMO_	−5.8958 ev
Lumo energy	E_LUMO_	−2.5429 ev
Ionization energy	E_Z_	5.8958 ev
Electron affinity	E_A_	2.5429 ev
Chemical potential	µ	4.2194 ev
Electronic negativity	Χ	−4.2194 ev
Electronic hardness	η	1.6764
Electronic softness	σ	0.5964 ev
Electrophilicity	ω	1.6764 ev
Dipole moment	D_M_	3.9649 Debye
Total ground energy	E_T_	−62,980.37 ev
Energy gap	E_G_	3.3529 ev
Energy change	Δ E	−1.6764 ev

From the quantum simulation of HOMO and LUMO energy levels, the energy gap (E_G_) is calculated to be 3.3529 eV, representing the difference between these two orbitals (Fig 3B). The energy gap is a crucial parameter for molecular reactivity, as a smaller gap indicates a higher possibility of charge transfer, enhancing the compound’s inhibitory reactivity. Additionally, based on the optimization results, global quantum parameters were calculated. As shown in Table 1, the molecular softness (σ) is relatively low, while the hardness (η) is high. Furthermore, the electrophilicity index is notably high, suggesting that compound **14** has a strong ability to attract electrons from the target site. This implies that the compound could possess significant inhibitory reactivity. Furthermore, the diagram illustrating the total density of states (TDOS) was generated using the Multiwfn software and is presented in Fig 3C. This visualization provides valuable insights into the electronic structure of the system.

The charge distribution of compound **14** was analyzed using Mulliken charge distribution, which identifies the charge on each atom within the molecule. The results reveal that the highest negative charge is localized on atom C12, while the highest positive charge is found on atom S15, as shown in Fig 3D. This distribution highlights the potential for interactions between compound **14** and its target. Specifically, the molecule can form bonds with the target surface through the donor-acceptor relation, where the negatively charged sites act as electron donors, and the positively charged sites serve as electron acceptors, as illustrated in Fig 3E. These findings suggest that compound **14** has a strong capacity to establish stable interactions with its target.

The electrostatic surface potential (ESP) of molecule **14** is analysed in Fig 3E, with the ESP map generated based on DFT optimization. Generally, the ESP map is a valuable tool for predicting the connection between a drug molecule and its receptor by identifying regions of electrophilic interaction and inhibitory reactivity. Each color on the map provides insights into the electron density and atom type: red indicates regions rich in electrons, yellow signifies electron-rich regions but less so than red, blue represents electron-deficient areas and green marks neutral regions. In this case, the ring and carbon atoms are localized in the green (neutral) region. The oxygen atoms are in the red and yellow regions, which are characterized by negative electrostatic potential. Meanwhile, the hydrogen atoms are situated in the blue region, indicating positive electrostatic potential. This blue region corresponds to the drug’s active site, where strong electrophilic interactions with the target site are likely to occur.

The quantum electronic properties of atoms within compound **14** were investigated using the Multiwfn and AIMALL program packages. This analysis focused on the topology of the bond critical points (BCPs), providing detailed insight into the molecule’s electronic structure. The calculated parameters as well as their relations are summarized in Table S.1 in S1 Data, including electron density (ρ), Laplacian of electronic charge density (∇²ρ), Hamiltonian kinetic energy (K(r)), kinetic electron energy density (G(r)), potential electron energy density (V(r)), and total energy density (H(r)).

The analysis indicates that all bonds in compound **14** are covalent, as evidenced by the consistently negative values of ∇²ρ and H(r), alongside positive values for K(r). However, certain pairs of atoms—N19-H43, N19-H35, N3-H53, and C10-H35—exhibit positive ∇²ρ and H(r) values, paired with negative K(r) values. These findings suggest that these atom pairs are associated with weak attractive electrostatic interactions, rather than strong covalent bonding.

Finally, Fig 3F provides a comprehensive visualization of all the bond paths present within compound **14**, offering a clear representation of its molecular structure. Additionally, Fig 3G presents the electron density distribution on an XY plane, providing detailed insight into the spatial variation of electron density within the compound.

#### 3.1.2. Molecular docking investigations.

**Fig 4:**
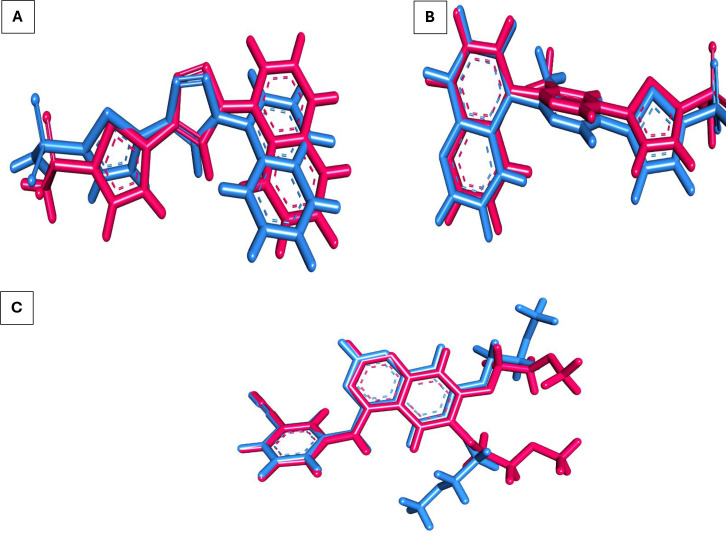
Superimposition of the native ligand (pink) and the re-docked ligand (blue) within A) hCA_IX active site 5FL4 (RMSD 0.96 Å), B) hCA_XII active site 8CO3 (RMSD 0.82 Å), and C) and EGFR active site 4HJO (RMSD 1.33 Å).

Molecular docking investigations are used to anticipate atomic-level interactions between the designed candidates and various enzymes. This study used two human carbonic anhydrase enzymes including hCA_IX (PDB: 5FL4), hCA_XII (PDB: 8CO3), and one EGFR enzyme (PDB: 4HJO) to perform molecular docking investigations of a novel sulfamoylphenyl-dihydro-thiadiazole derivative **14**. To validate the docking methodology, the identical conformer of the co-crystallized ligands for the three enzymes was redocked. The low RMSD values for each receptor showed the fidelity of this methodology (Fig 4).

Compound **14** was subsequently docked employing the same validation step parameters to examine its critical interactions within the active sites.

**Fig 5:**
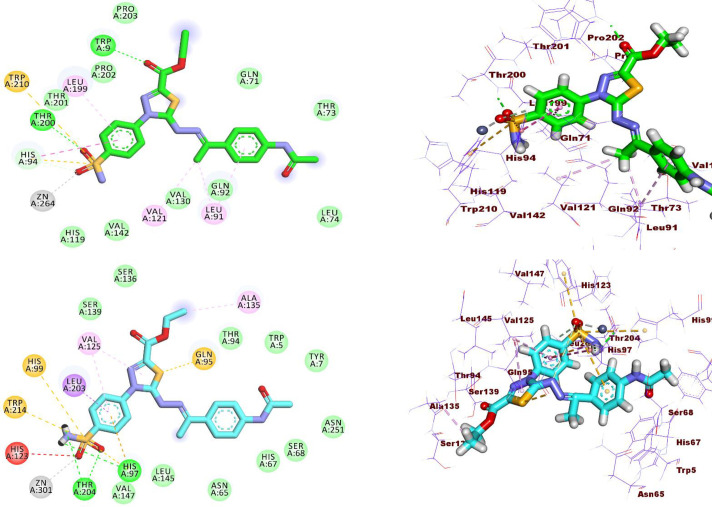
A) 2D and 3D docking images of compound 14 within the hCA_IX active pocket (code: 5FL4). B) 2D and 3D docking images of compound 14 within hCA_XII active pocket (code: 8CO3).

Initially, the docking of compound **14** within hCA isoforms IX and XII demonstrated the typical function of the SO_2_NH_2_ moiety as a zinc-binding entity, engaging with the Zn (II) ion (Fig 5A and B). In the active site of hCA_IX, the SO_2_NH_2_ moiety of compound **14** established a hydrogen bond with Thr200. Furthermore, an additional hydrogen bond was noted between thiadiazole-2-carboxylate and Trp 9.

Furthermore, within the catalytic site of hCA_IX, the 4-sulfamoyl phenyl ring engaged in hydrophobic interactions with Leu199, His94, and Trp210. Moreover, the 4-acetamidophenyl ring exhibited an orientation directed towards Leu91, whereas the ethylidene moiety was positioned towards both Leu91 and Val121 (Fig 5A).

Likewise, the docking of compound **14** into hCA_XII demonstrated three hydrogen bond interactions between the SO_2_NH_2_ group and Thr204 and His97. Moreover, the 4-sulfamoyl phenyl ring engaged in hydrophobic contact with Val125, Leu203, His99, His123, and Trp214 residues, whereas the thiadiazole ring was orientated towards Val125 and Gln95 (Fig 5B).

**Fig 6:**
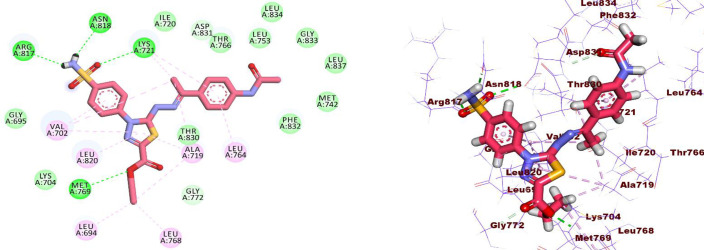
2D and 3D docking images of compound 14 inside the active site of EGFR (code: 8CO3).

Regarding EGFR, compound **14** interacted through four hydrogen bonds through the SO_2_NH_2_ group (Arg817, Asn818, Lys721 901) and the 2-carboxylate oxygen (Met769). Furthermore, the 4-sulfamoyl phenyl ring interacted with the side chain of Val702 *via* pi-pi stacking interaction. The thiadiazole ring participated in hydrophobic interaction with Val702, Ala719, and Leu820 while the 4-acetamidophenyl ring was oriented towards Leu764 and Lys721. In addition, the methyl group of the 2-ethylcarboxylate moiety formed three hydrophobic interactions with Leu694, Leu768, and Ala719 residues (Fig 6).

#### 3.1.3. Molecular dynamic (MD) simulations.

**MD simulations against hCA_IX.** The structural stability of the hCA_IX enzyme, both in its free form and in complex with Compound **14**, was evaluated during a 200 ns MD simulation. This assessment involved calculating the root mean square deviation (RMSD) of atomic positions relative to the starting structures. Additionally, other key parameters, including the radius of gyration (Rg), solvent-accessible surface area (SASA), and the total number of hydrogen bonds (H-bonds) in both free and bound states, were monitored throughout the simulation.

**Fig 7:**
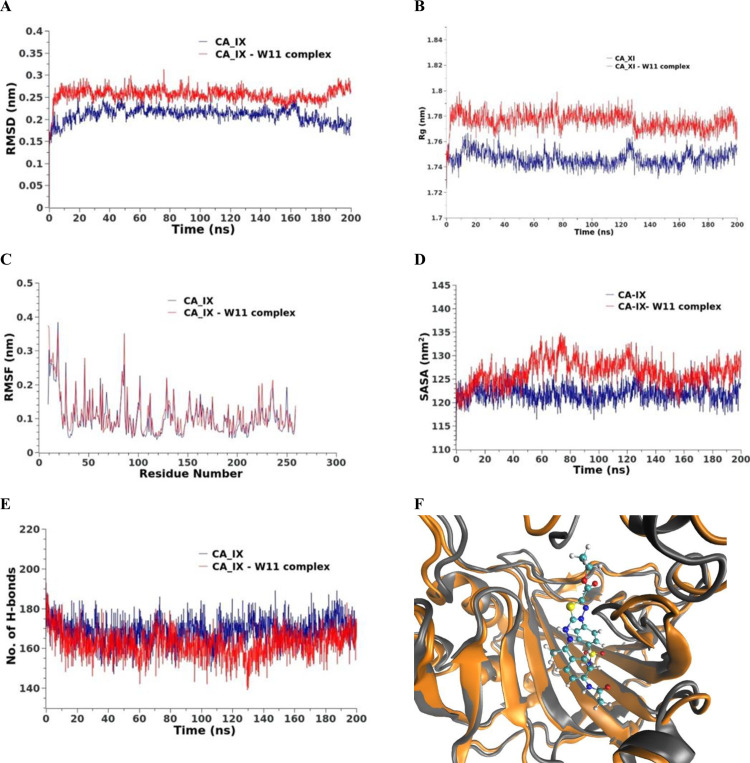
MD results for hCA_IX (blue line) and hCA IX – compound 14 complex (red line). A) RMSD values from the trajectory, B) Radius of gyration, C) RMSF, D) SASA, E) total number of H-bonds as a function of time, and F) the induced conformation the active site upon the compound 14 binding over the last 10 ns, the reference is colored in gray and the complex in orange.

The RMSD values for the free protein increased slightly from 0.2 to 0.22 nm, indicating structural stability over time (Fig 7A). A similar behavior was observed for the protein-drug complex, with an initial RMSD shift from 0.25 to 0.27 nm, followed by an increase to 0.32 nm during the final 20 ns. This increase in RMSD for the complex reflects conformational changes induced in the active site upon drug binding. The average radius of gyration (Rg) and SASA values for both systems showed a noticeable increase between the 20th and 120th ns compared to the reference state, suggesting slight structural expansion during this period. However, these values decreased during the final 80 ns, returning closer to the reference values, as shown in Fig 7B and Fig 7C, respectively. The total number of H-bonds within the protein was also analyzed (Fig 7D). For the protein-drug complex, there was a reduction in the number of H-bonds between the 20th and 120th ns, indicative of transient structural adjustments upon complex formation. By the final 80 ns, the number of H-bonds recovered, stabilizing to values similar to those in the free protein. To assess residue-level flexibility, the root mean square fluctuations (RMSF) of the protein residues were averaged over the 200 ns simulation (Fig 7E). The RMSF analysis showed no significant changes in residue flexibility in the protein-drug complex compared to the free protein, indicating that the overall structural dynamics of the protein were preserved despite drug binding. These findings collectively highlight the stability of the CA_IX enzyme and its complex with compound **14**, with transient conformational changes during the simulation attributable to drug interaction with the active site.

**Fig 8:**
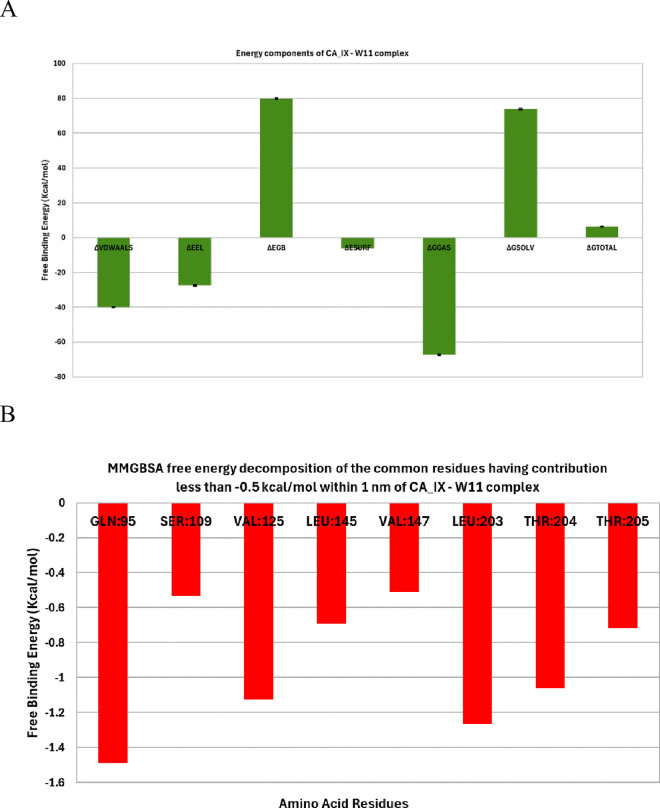
MM-GBSA analysis of CA_IX – compound 14 complex.

***MM-GBSA studies.*** The binding free energy calculations for the complexes were performed using the MM-GBSA method, as illustrated in Fig 8. For hCA_IX – compound **14** complex (Fig 8A), the analysis revealed that Van der Waals (−39.98 kcal/mol) and electrostatic (−27.35 kcal/mol) interactions played a dominant role in stabilizing the protein-drug binding. An energy decomposition analysis was conducted to evaluate the contributions of individual amino acids within 1 nm proximity to the ligand (Fig 8A). This analysis identified four key amino acids—Gln95, Val125, Leu203, and Thr204—that significantly influenced the binding of compound **14** to the hCA_IX enzyme. Among these, Gln95 exhibited the highest contribution to the binding energy at −1.49 kcal/mol, followed by Leu203 with a contribution of −1.26 kcal/mol. Val125 and Thr204 contributed positively to the binding, with values of 1.125 kcal/mol and 1.06 kcal/mol, respectively.

**Fig 9:**
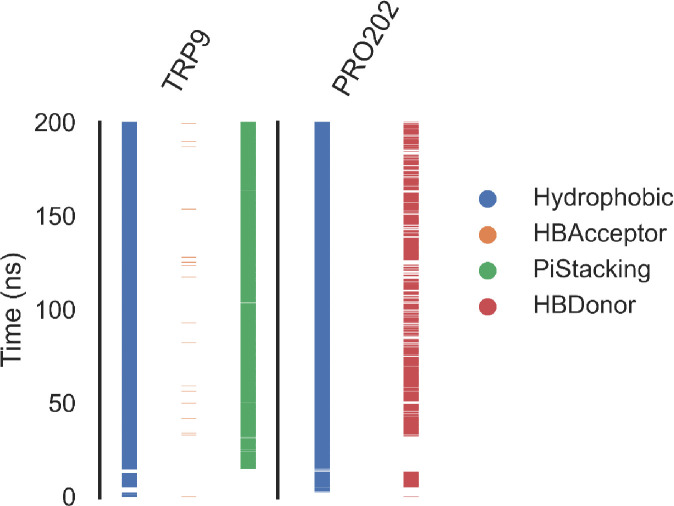
Amino acids and interaction types with the hCA_IX – compound 14 complex.

***Protein-ligand interaction fingerprints (ProLIF) analysis.*** ProLIF analysis provided valuable insights into the specific amino acids involved in compound **14** interactions within the binding pocket of hCA_IX. As shown in Fig 9, two amino acids exhibited a notably high propensity for hydrophobic interactions, occurring in more than 80% of the frames analyzed during the simulation. These key residues are TRP9, with a hydrophobic interaction occurrence of 95.9%, and PRO, with an occurrence of 92.3%. This analysis highlights the critical role of TRP9 and PRO in stabilizing compound **14** within the binding pocket through persistent hydrophobic interactions. Overall, ProLIF analysis provides a detailed understanding of the ligand-protein interactions, emphasizing the importance of these residues in the binding affinity and stability of the hCA_IX- compound **14** complex.

**Fig 10:**
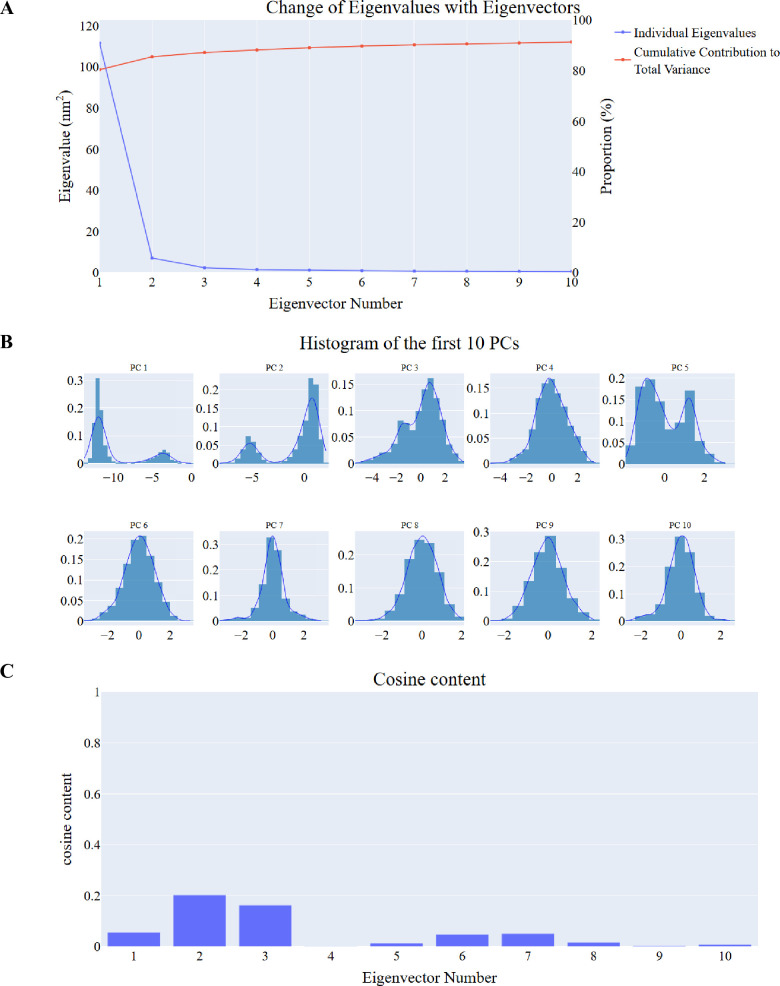
MM-GBSA analysis of hCA_IX – compound 14 complex. A: the change in the eigenvalues with increasing the eigenvectors (blue) and the cumulative variance retained in the eigenvectors (red), B: The distribution of the first ten eigenvectors, C: cosine content values of the first ten eigenvectors.

***Principal component analysis (PCA).*** Our method of choice for identifying coordinated movements of the α-carbon atoms in the hCA_IX complex was PCA. Several factors listed in the Methods section have to be carefully considered to determine the reduced subspace’s proper dimensions. These included the cumulative variance explained by increasing the number of principal components (PCs), the scree plot, and the eigenvector distribution. The scree plot (Fig 10A) showed that the slope began to significantly reduce at the third PC, indicating a possible point of inflection for the selection of dimensionality. As seen in Fig 10, the first eigenvector alone accounted for a significant 80.36% of the overall variance, while the first three PCs combined contributed roughly 87.14%. This suggested that these three PCs accounted for a sizable fraction of the total protein movements. The distributions of the first five PCs differed from the Gaussian distribution, which further supported this decision (Fig 10B). A non-Gaussian distribution frequently indicates the existence of significant, non-random motions that these components can record. We thus chose the top five PCs as examples of the crucial subspace based on a combination of the scree plot, variance collected, and non-Gaussian eigenvector distributions. We subsequently examined the cosine content of the first ten principal components (PCs) for the hCA_IX system (Fig 10C) to assess the randomness and possible redundancy within the identified critical subspaces. Except for the second PC (0.2 for hCA_IX _compound **14** complex) and the third PC of the complex (0.162), all other cosine values for the first ten PCs remained below 0.2. This suggests that the identified essential motions captured by the PCs are not random.

**Fig 11:**
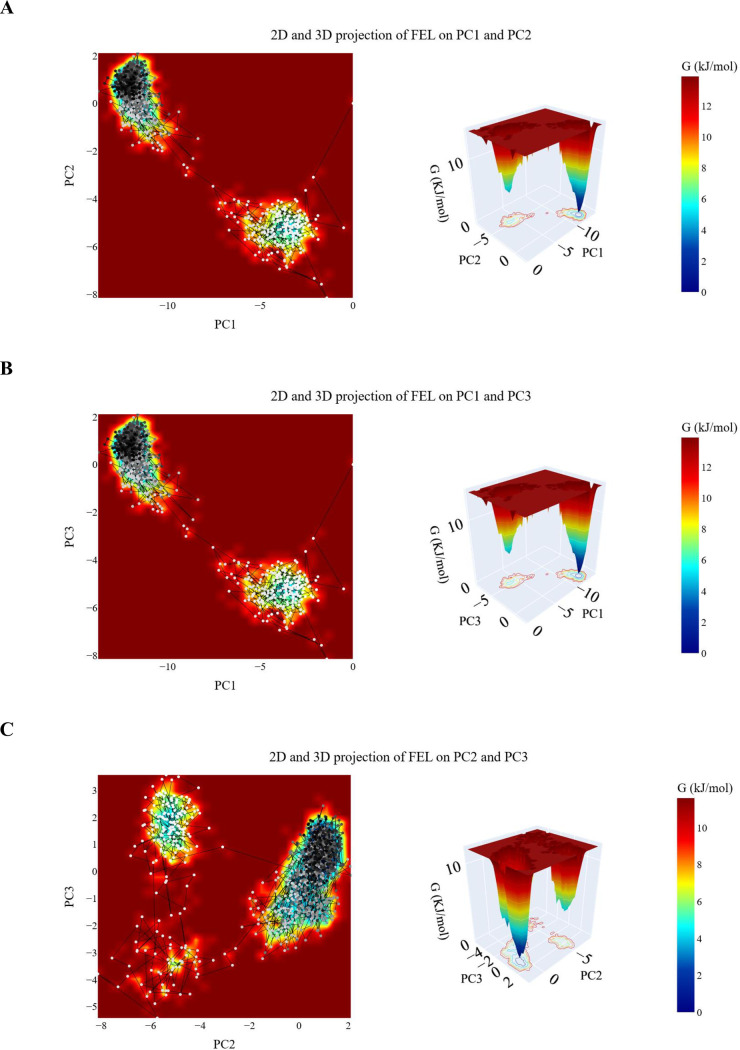
The 2D and 3D projections of the hCA_IX _compound 14 trajectory’s FEL on A: the first two, B: the first and third, and C: the second and third eigenvectors. Small white-to-black dots are the frames of the hCA_IX in the hCA_IX_ compound **14** simulation colored based on time. The color of the energy levels in 2D and 3D projection is shown on the scale and corresponds to the free energy as calculated using the gmx sham command.

***Free energy landscape (FEL) Analysis.*** The projected trajectories onto various two-dimensional planes defined by the chosen PCs are displayed in Fig 11. Every graphic displays a different landscape with different basins that correspond to local minima on the FEL. These basins correspond to preferred conformations adopted by the protein-ligand complex during the simulation. For the projection on the first two PCs (Fig 11A), the sampling starts from (white and grey dots) basin and then moves to a transient basin (dark grey dots) before finally reaching the most stable one (black dots). There is a 3.25 KJ/mol difference between the global minimum and the next minimum. Fig 11B presents the PC1-PC3 projection, which shows a projection with two basins, the trajectories move from the transient basin to the stable basin. The energy difference between the global minimum and the next minimum is calculated to be 3.2 KJ/mol. Similarly, Fig 11C demonstrates the projection on the PC2 and PC3, revealing three basins. The trajectory begins in the upper basin and then transitions to two different basins before eventually reaching the most stable basin at the end of the simulation (black dots). The difference between the second and global minima is 1.62 KJ/mol.

**Fig 12:**
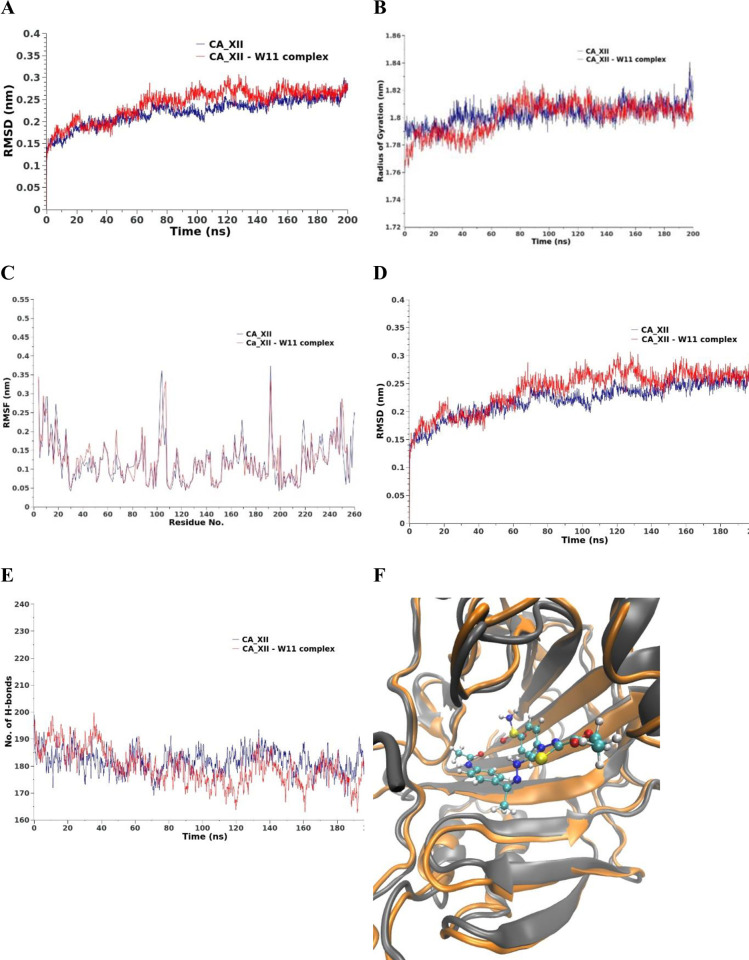
MD results for hCA_XII (blue line) and hCA_XII – compound 14 complex (red line). A) RMSD values from the trajectory, B) Radius of gyration, C) RMSF, D) SASA, E) total number of H-bonds as a function of time, and F) the induced conformation the active site upon the compound 14 binding over the last 10 ns, the reference is colored in gray and the complex in orange.

**MD Simulations against the hCA_XII.** For the hCA_XII enzyme, Fig 12A. shows a slight increase in RMSD for the protein-drug complex compared to the reference, with a deviation of approximately 0.6 nm observed from the 60th ns to the 140th ns. This period of fluctuation is followed by stabilization of the structure during the final 60 ns of the simulation. The Rg was also measured to assess changes in the protein’s compactness (Fig 12B). The results confirm that there are no significant changes in Rg from the 60th ns to the end of the 200 ns simulation, further supporting the structural stability of the protein in the complex. The SASA of the entire protein fluctuated within a narrow range of approximately 2 nm² compared to the reference state, as shown in Fig 12C. These minor fluctuations indicate that the overall exposure of the protein’s surface to the solvent remains relatively consistent, with no drastic structural alterations upon drug binding. The RMSF of individual protein residues were analysed to examine residue-specific flexibility (Fig 12D). The RMSF values demonstrate no significant differences between the free and bound states, indicating that the binding of the drug does not notably affect the flexibility of the protein residues. Lastly, the total number of hydrogen bonds in the protein was calculated for both the free and bound states (Fig 12E). In the complex, a slight decrease in the number of hydrogen bonds was observed between the 90th and 170th ns, suggesting transient disruptions in hydrogen bonding due to drug binding. However, by the final 30 ns of the simulation, the protein regains its hydrogen bonds, returning to values similar to the reference state. In summary, the binding of compound **14** to hCA_XII induces minor structural changes, primarily during the middle stages of the simulation, but the overall stability and flexibility of the protein are maintained throughout the course of the simulation.

**Fig 13:**
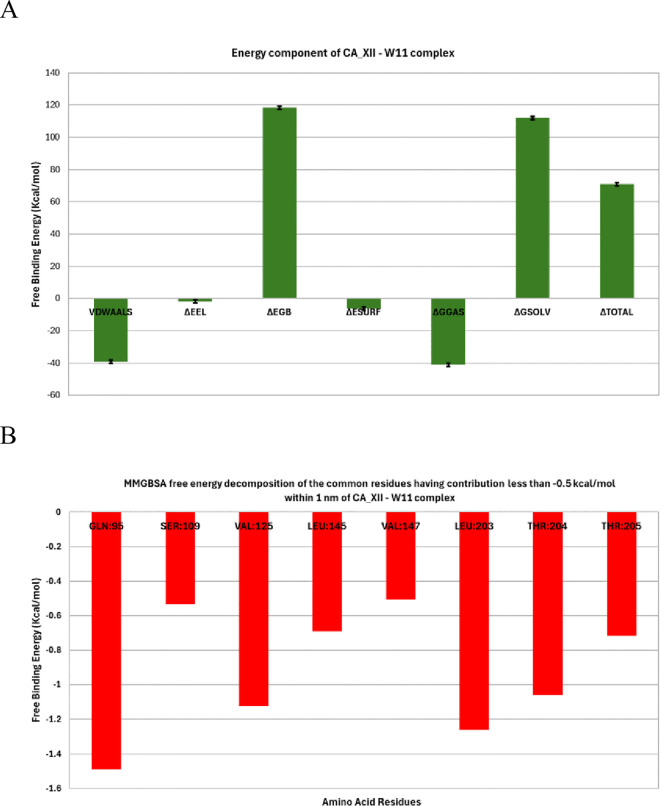
MM-GBSA components B) free binding energy decomposition of the hCA_XII _ compound 14 complex.

***MM-GBSA studies.*** For the hCA_XII enzyme in complex with compound **14**, binding free energy calculations using the MM-GBSA method highlighted the dominant role of Van der Waals interactions, which contributed −39.19 kcal/mol to the total binding energy (Fig 13A). These interactions are essential in stabilizing the ligand within the enzyme’s binding pocket, underscoring their critical influence on the complex’s stability.

A detailed energy decomposition analysis was conducted to identify the contributions of individual amino acids within the binding pocket to the total binding energy (Fig 13B). Four key residues—Gln95, Val125, Leu203, and Thr204—were identified as having a significant impact on the binding of compound **14** to hCA_XII. Among these, Gln95 exhibited the highest contribution, with a binding energy of −1.49 kcal/mol, reflecting its critical role in anchoring the ligand through favorable interactions. Leu203 also displayed a strong contribution with a binding energy of −1.26 kcal/mol, further stabilizing the complex.

In contrast, Val125 and Thr204 contributed positively to the binding energy, with values of 1.125 kcal/mol and 1.06 kcal/mol, respectively. These residues likely participate in structural support and alignment of the ligand within the active site, complementing the stronger stabilizing effects of Gln95 and Leu203.

**Fig 14:**
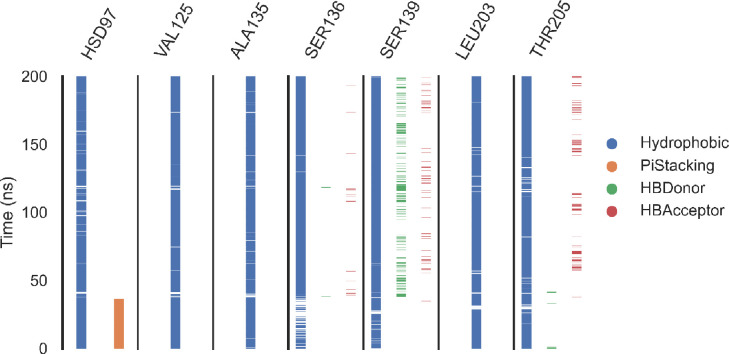
The amino acids, the types of interactions with the hCA_XII – compound 14 complex.

***Protein-ligand interaction fingerprints (ProLIF) studies.*** The ProLIF analysis provided detailed insights into the specific amino acids involved in interactions with compound **14** within the binding pocket of hCA_XII. As illustrated in Fig 14, two amino acids exhibited a notably high propensity for hydrophobic interactions, occurring in over 80% of the analyzed simulation frames. These key residues are LEU203, with an interaction frequency of 87.7%, and HSD97, with an interaction frequency of 86.8%. Such consistent interactions highlight the critical role of these residues in stabilizing the ligand within the binding site.

Additionally, the analysis identified other amino acids that also contributed significantly to hydrophobic interactions, albeit with slightly lower frequencies (>50% occurrence). These residues include ALA135 (67.2%), SER136 (61.11%), and THR205 (74.3%). Their contributions further support the hydrophobic environment essential for accommodating compound **14** in the binding pocket.

The ProLIF analysis underscores the importance of these interactions in the ligand’s binding affinity and stability. By identifying the amino acids most actively involved in hydrophobic interactions, it provides a comprehensive understanding of the molecular interactions between compound **14** and hCA_XII. These insights are valuable for refining drug design and optimizing molecular interactions for enhanced binding efficiency and specificity.

***Principal component analysis (PCA) studies.*** We employed the PCA principles to identify coordinated movements of the α-carbon atoms in the hCA_XII complex. Determining the appropriate dimensions of the reduced subspace required careful consideration of several factors outlined in the Methods section. These factors included the cumulative variance explained by increasing the number of principal components (PCs), the scree plot, and the distribution of eigenvectors.

**Fig 15:**
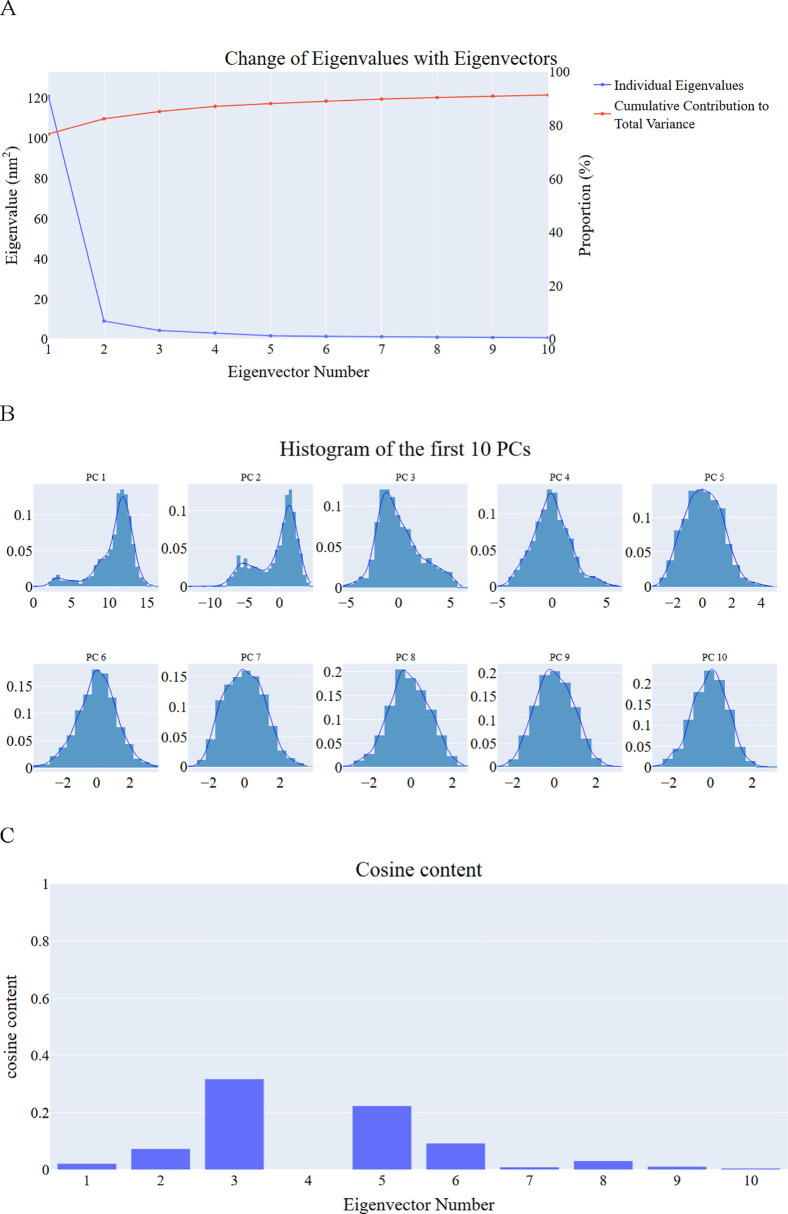
PCA analysis of hCA_XII – compound 14 complex. A: the change in the eigenvalues with increasing the eigenvectors (blue) and the cumulative variance retained in the eigenvectors (red), B: The distribution of the first ten eigenvectors, C: cosine content values of the first ten eigenvectors.

The scree plot (Fig 15A) revealed a noticeable reduction in slope starting at the third PC, indicating this as a potential inflection point for dimensionality selection. Notably, the first eigenvector accounted for 76.7% of the total variance, and the combined contribution of the first three PCs was approximately 85.1%, capturing a significant portion of the protein’s overall motion. The distributions of the first four PCs deviated from a Gaussian distribution (Fig 15B), suggesting the presence of meaningful, non-random motions. This non-Gaussian behavior further supported the selection of these three PCs as the most representative subspace.

To evaluate the randomness and potential redundancy of the identified critical subspaces, we analyzed the cosine content of the first ten PCs for the hCA_XII system (Fig 15C). Except for the second PC (0.1 for the hCA_XII_compound **14** complex) and the third PC of the complex (0.3), all cosine values for the first ten PCs remained below 0.3. This low cosine content indicates that the essential motions captured by the selected PCs are non-random and meaningful.

Based on the combined evidence from the scree plot, variance explained, and non-Gaussian eigenvector distributions, we selected the top three PCs as representative of the critical subspace for subsequent analysis. These PCs effectively describe the significant motions within the system.

**Fig 16:**
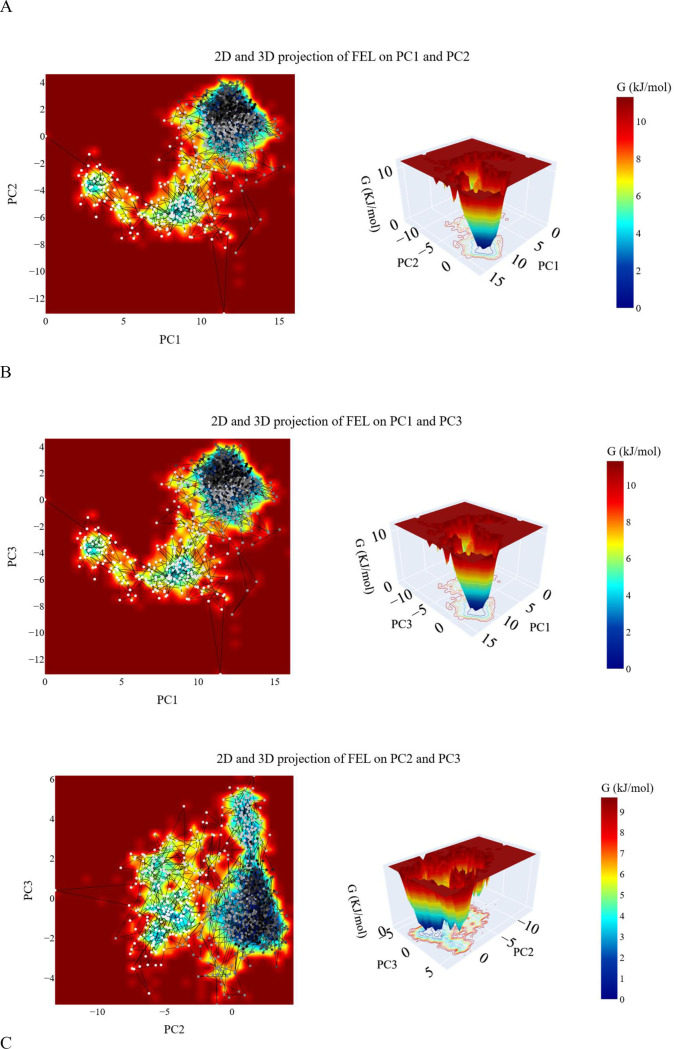
PCA analysis of the hCA_XII–compound 14 complex. (A) Eigenvalue variation with increasing eigenvectors (blue line) and the cumulative variance explained (red line); (B) distribution of the first ten eigenvectors; (C) cosine content values for the first ten eigenvectors.

***Free energy landscape (FEL) studies.*** The projected trajectories of the system onto various two-dimensional planes defined by the selected principal components (PCs) are illustrated in Fig 16. Each plot represents a distinct energy landscape characterized by basins corresponding to local minima on the FEL. These basins indicate preferred conformations adopted by the protein-ligand complex during the simulation.

In the projection onto the first two PCs (PC1-PC2, Fig 16A), the trajectory begins in a narrow basin (represented by white and grey dots), transitions through a transient basin (dark grey dots), and eventually settles into the most stable basin (black dots). The energy difference between the global minimum and the next local minimum is calculated to be 0.25 KJ/mol. The projection onto the PC1-PC3 plane (Fig 16B) reveals a landscape with three distinct basins: one wide basin and two narrower basins. The global minimum is located in the wide basin, and the energy difference between the global minimum and the next minimum is measured at 0.24 KJ/mol. Similarly, the projection onto the PC2-PC3 plane (Fig 16C) shows three basins. The trajectory starts in a large basin during the early stages of the simulation, transitions through two intermediate basins, and finally reaches the basin with the greatest stability, represented by black dots. The energy difference between the second local minimum and the global minimum is calculated to be 0.4 KJ/mol.

These projections provide a detailed visualization of the conformational transitions and stability of the protein-ligand complex, highlighting the energy differences between key states and offering insights into the system’s dynamic behavior.

**Fig 17:**
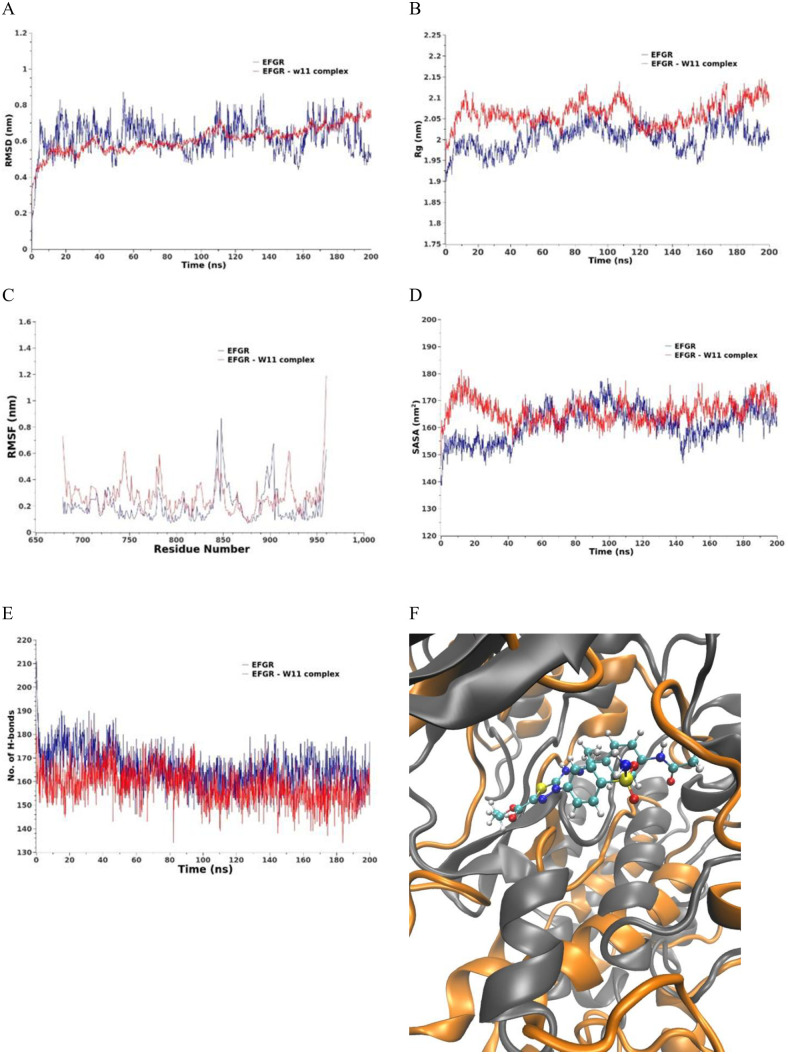
MD results for Epidermal growth factor receptor (EFGR) (blue line) and EFGR– compound 14 complex (red line). A) RMSD values from the trajectory, B) Radius of gyration, C) RMSF, D) SASA, E) total number of H-bonds as a function of time, and F) the induced conformation the active site upon the compound 14 binding over the last 10 ns, the reference is colored in gray and the complex in orange.

**MD simulations against EGFR.** The EGFR_compound **14** complex exhibits a slight increase in RMSD values, ranging from 0.4 to 0.8 nm over the 200 ns simulation period compared to the reference structure, as shown in Fig 17A. This indicates minor structural deviations upon drug binding while maintaining overall stability. The protein in the complex maintains an average Rg with fluctuations within a range of 0.15 nm compared to the reference, which fluctuates by 0.1 nm, as depicted in Fig 17B. This consistent Rg suggests that the compactness of the protein structure remains largely unaltered by drug binding. The SASA, calculated as a function of time, remains stable from the 40th to the 200th ns for the protein in the complex compared to the reference, as illustrated in Fig 17C. This stability in SASA indicates that the exposure of the protein to the solvent is not significantly affected by the presence of compound **14**. The flexibility of the protein residues, assessed through the RMSF averaged over the entire 200 ns simulation, shows a general increase in flexibility upon compound **14** binding. Notably, residues 825–910 exhibit a distinct elevation in flexibility, as shown in Fig 17D. This localized increase suggests dynamic changes in specific regions of the protein that may be crucial for ligand interaction and binding adaptability.

The total number of hydrogen bonds (H-bonds) formed in the drug-bound protein is slightly increased by an average of 10 bonds compared to the reference structure, as depicted in Fig 17E. This increase in H-bonding contributes to the stability of the protein-ligand complex.

The active site conformation of the EGFR protein, averaged over the last 10 ns of the simulation, is illustrated in Fig 17-F for both the bound and free states. This comparison highlights the conformational adjustments within the active site due to compound **14** binding, providing further insights into the molecular interactions stabilizing the complex.

Overall, these analyses underscore the structural and dynamic changes induced in EGFR upon binding to compound **14**, reflecting its potential impact on the protein’s stability and functional conformation.

**Fig 18:**
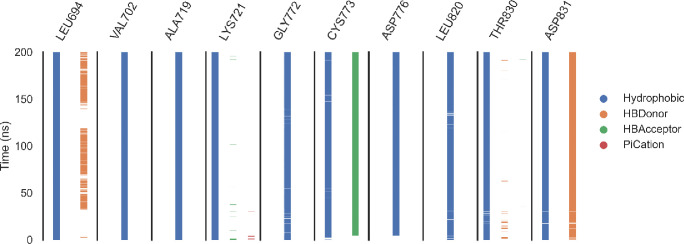
The amino acids and interactions with the EGFR_compound 14 complex.

***ProLIF analysis.*** ProLIF analysis identified the specific amino acids involved in ligand interactions within the EGFR binding pocket. As shown in Fig 18, ten amino acids demonstrated a high tendency for hydrophobic interactions and van der Waals (vdW) contacts, each occurring in more than 80% of the simulation. These key residues include LEU694, with 98% hydrophobic interactions and 90% vdW contact interactions; VAL702, exhibiting 95.5% hydrophobic interactions; ALA719, showing 99% hydrophobic interactions; and LYS721, contributing 96.6% hydrophobic interactions. GLY772 accounted for 90.2% hydrophobic interactions, while CYS773 displayed 96.5% H-bond acceptor interactions and 97.9% vdW contact interactions. ASP776 participated in 98.4% hydrophobic interactions, LEU820 in 92.2% hydrophobic interactions, THR830 in 91.3% hydrophobic interactions, and ASP831 in 94.5% H-bond donor and 98% vdW contact interactions.

***Principal component analysis.*** Principal Component Analysis (PCA) was employed to identify coordinated motions of the α-carbon atoms in the EGFR complex. Determining the appropriate dimensions of the reduced subspace required careful consideration of several factors outlined in the Methods section. These included the cumulative variance explained by adding principal components (PCs), the scree plot, and the eigenvector distribution.

**Fig 19:**
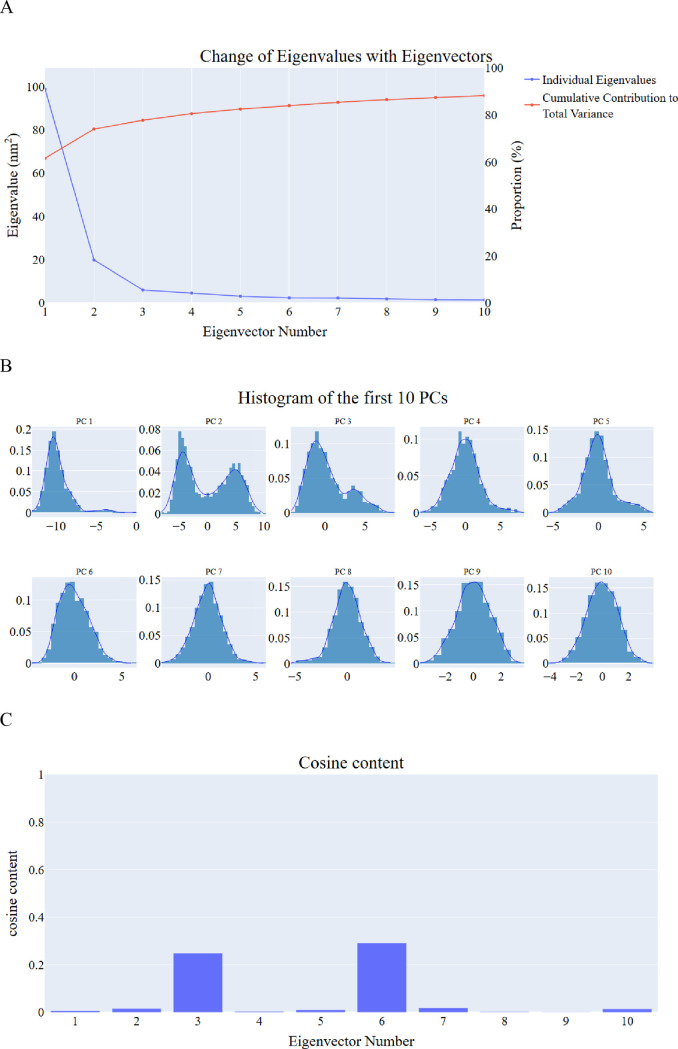
PCA analysis of EGFR – compound 14 complex. A: the change in the eigenvalues with increasing the eigenvectors (blue) and the cumulative variance retained in the eigenvectors (red), B: The distribution of the first ten eigenvectors, C: cosine content values of the first ten eigenvectors.

The scree plot (Fig 19 A) revealed a noticeable reduction in the slope after the third PC, indicating a potential point of inflection for dimensionality selection. As illustrated in Fig 19, the first eigenvector accounted for 61.6% of the total variance, while the first three PCs collectively captured approximately 77.7%, suggesting these three PCs represented a significant portion of the protein’s overall motion. The eigenvector distributions for the first four PCs deviated from a Gaussian distribution (Fig 19 B), further supporting the presence of significant, non-random motions captured by these components.

Based on the scree plot, the explained variance, and the non-Gaussian nature of the eigenvector distributions, the top three PCs were selected to define the critical subspace. To evaluate the randomness and potential redundancy within these subspaces, the cosine content of the first ten PCs was analyzed for the EGFR system (Fig 19C). Except for the second PC (0.015 for the EGFR_compound **14** complex), the third PC (0.25), and the sixth PC (0.29), all other cosine values for the first ten PCs remained below 0.25. These results indicate that the essential motions captured by the selected PCs are not random and effectively represent the key dynamics of the system.

**Fig 20:**
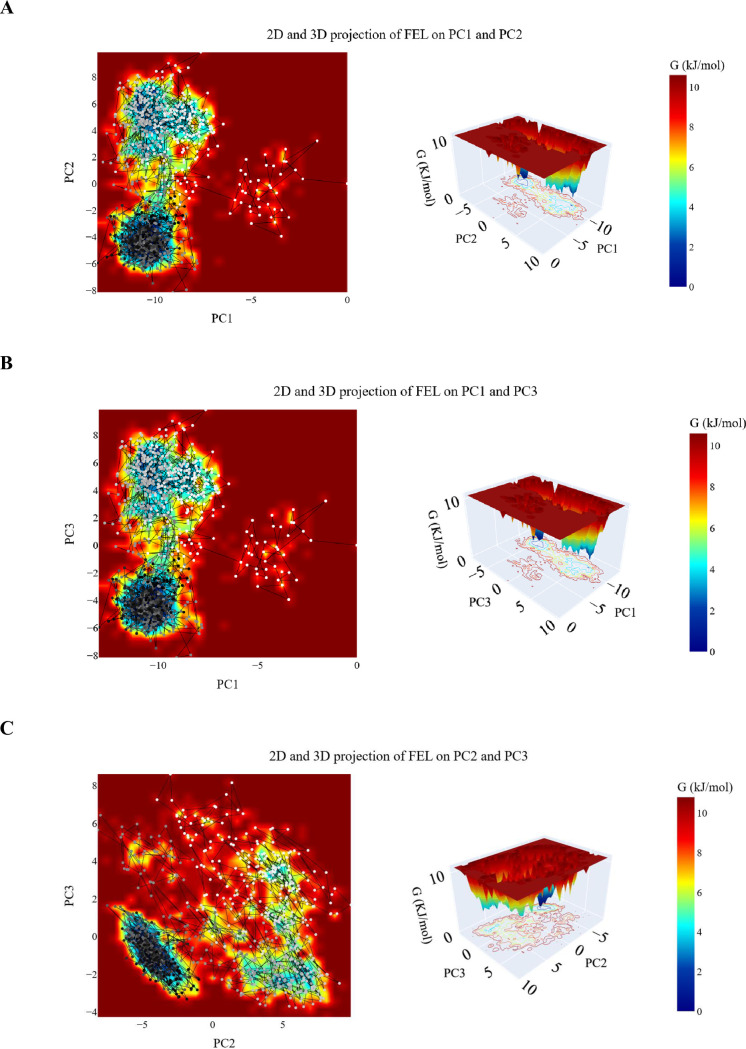
The 2D and 3D projections of the EGFR _compound 14 trajectory’s FEL on A: the first two, B: the first and third, and C: the second and third eigenvectors. Small white-to-black dots are the frames of the EGFR in the EGFR _ compound **14** simulations colored based on time. The color of the energy levels in 2D and 3D projection is shown on the scale and corresponds to the free energy as calculated using the gmx sham command.

***Free energy landscape analysis.*** The trajectories projected onto two-dimensional planes defined by the selected principal components (PCs) are shown in Fig 20. Each plot illustrates a unique energy landscape with distinct basins representing local minima on the Free Energy Landscape. These basins correspond to the favored conformations adopted by the protein-ligand complex during the simulation. In the PC1-PC2 projection (Fig 20A), the trajectory begins in a narrow basin (white dots), progresses through a transient basin (white and grey dots), and eventually settles into the most stable basin (dark grey and black dots). The energy difference between the global minimum and the second-lowest minimum is 0.126 KJ/mol. The PC1-PC3 projection (Fig 20B) shows three distinct basins: one small basin and two larger basins. The energy gap between the global minimum and the next minimum is 0.12 KJ/mol. In the PC2-PC3 projection (Fig 20C), two primary basins are observed. The trajectory starts in a large basin at the upper region of the plot and transitions to the most stable basin (black dots). The energy difference between the global minimum and the second minimum is 0.505 KJ/mol. These FEL projections provide a detailed understanding of the dynamic conformational states and stability of the protein-ligand complex throughout the simulation.

#### 3.1.4. *In silico* ADMET analysis.

An *in silico* ADMET (Absorption, Distribution, Metabolism, Excretion, and Toxicity) analysis was performed for compound **14** using Discovery Studio software to evaluate its pharmacokinetic properties. The results, summarized in Table 2, provide insights into the compound’s potential behavior within biological systems. Interestingly, compound **14** presents several potential advantages in its pharmacokinetic profile. One key benefit is its selective metabolism, as it is predicted not to be metabolized by CYP2D6. This reduces the risk of interactions with other drugs that are substrates for this enzyme, making compound **14** a suitable candidate for combination therapies. Another notable advantage of compound **14** is its high plasma protein binding (PPB), which suggests that the drug may remain in the bloodstream for an extended period. This prolonged circulation could result in sustained therapeutic effects, which is particularly beneficial for chronic conditions requiring continuous drug presence. The higher PPB also means a slower rate of renal clearance, which could contribute to reduced dosing frequency and potentially improve patient compliance, especially for long-term treatments. In addition, compound **14**’s very low BBB permeability indicates that it is less likely to cross into the central nervous system. While this may limit its use in CNS-targeting therapies, it presents a significant advantage for peripheral applications. Its limited CNS penetration reduces the risk of neurological side effects, making it a safer option for diseases that do not require brain involvement, such as peripheral cancers. This characteristic also makes it less likely to cause central nervous system toxicity, a common concern with drugs that penetrate the BBB. The low solubility and poor absorption of compound **14**, while posing challenges for oral bioavailability, could actually be leveraged in specialized drug delivery systems. For instance, controlled-release formulations or targeted drug delivery could overcome these limitations, ensuring that the compound reaches its intended site of action at effective concentrations. In conclusion, while compound **14** has some limitations in terms of solubility and absorption, its pharmacokinetic advantages, such as reduced CYP2D6 interactions, extended circulation time due to high PPB, and selective peripheral targeting, make it a promising candidate for therapeutic development. With further optimization of its delivery mechanisms, it has the potential to be effective in various therapeutic contexts, particularly in cases where CNS targeting is not required.

**Table 2:** Pharmacokinetic Properties of compound 14.

Comp.	BBB level	Solubility level	Absorption level	CYP2D6 prediction	PPB prediction
Compound **14**	Very low	Low	Poor	Non-inhibitor	> 90%
Erlotinib	Low	Low	Good	Non-inhibitor	> 90%
Acetazolamide	Very low	Optimal	Good	Non-inhibitor	< 90%

#### 3.1.5. *In silico* toxicity analysis.

An *in silico* toxicity analysis was carried out for compound **14** using Discovery Studio to assess its safety across various acute and chronic toxicity models. The results, detailed in Table 3, provide valuable insights into the compound’s potential toxicological risks. The toxicological assessment of compound **14** reveals several important safety features that make it an interesting candidate for further development. According to the Carcinogenic Potency TD_50_ in rats, compound **14** exhibits a value of 36.949, indicating a relatively low carcinogenic potency compared to acetazolamide, which shows a significantly higher carcinogenic score of 220.513. This suggests that compound **14** has a lower risk of inducing cancer in rats, making it a safer option in terms of long-term carcinogenic risk. Additionally, compound **14** is classified as a non-carcinogen based on the Mouse-Female FDA (MF-FDA) prediction, reinforcing its favorable safety profile.

**Table 3:** Compound 14 Potential Toxicological Potentials.

Toxicity Model	Compound 14	Erlotinib	Acetazolamide
TD_50_^a^	36.949	8.05746	220.513
MF-FDA	Non-Carcinogen	Non-Carcinogen	Single-Carcinogen
Ames	Non-Mutagen	Non-Mutagen	Non-Mutagen
DTP	Non-Toxic	Non-Toxic	Toxic
LD_50_^b^	15.8118	0.662169	17.4769
LOAEL ^b^	0.107924	0.0359487	0.230152
SI	None	None	None
OI	Mild	Mild	Mild

^a^: mg/kg/day; ^b^: g/kg.

In terms of genetic toxicity, compound **14** is predicted to be non-mutagenic according to the Ames test, a standard assay used to detect potential mutations in bacteria. This result is consistent with the prediction for Erlotinib and acetazolamide, both of which are also non-mutagenic. This lack of mutagenic potential suggests that compound **14** is unlikely to cause DNA damage that could lead to genetic mutations or cancerous growth, further supporting its safety for use in clinical settings. The rat oral LD_50_ of compound **14** is 15.8118, which is higher than that of Erlotinib (0.662169) but lower than acetazolamide (17.4769). This suggests that compound **14** is less toxic than acetazolamide but more toxic than Erlotinib in the event of an overdose. The Rat Chronic LOAEL of 0.107924 further supports this, indicating a relatively safe threshold for long-term exposure, though this still warrants cautious monitoring during extended use.

Compound **14** is classified as non-toxic based on its overall DTP (drug toxicity prediction), which is favorable compared to acetazolamide, which is predicted to be toxic. These findings suggest that while compound **14** is not entirely without risk, its overall toxicity is relatively low compared to other compounds, making it a potentially safer therapeutic option. Regarding irritancy, compound **14** is classified as having mild ocular irritancy (OI) and no skin irritancy (SI). This is consistent with the profile of Erlotinib, which also exhibits mild ocular irritancy and no skin irritancy. These irritancy levels suggest that while compound **14** may cause some mild eye discomfort, it is unlikely to cause significant adverse effects when handled or applied, which is an important consideration for both clinical and industrial applications.

In conclusion, compound **14** demonstrates a favorable toxicological profile with low carcinogenic and mutagenic risks, moderate acute toxicity, and mild irritancy. Compound **14** holds promise as a relatively safe candidate for development, with a lower risk of long-term toxic effects.

### 3.2.Chemistry

**Scheme 1:**
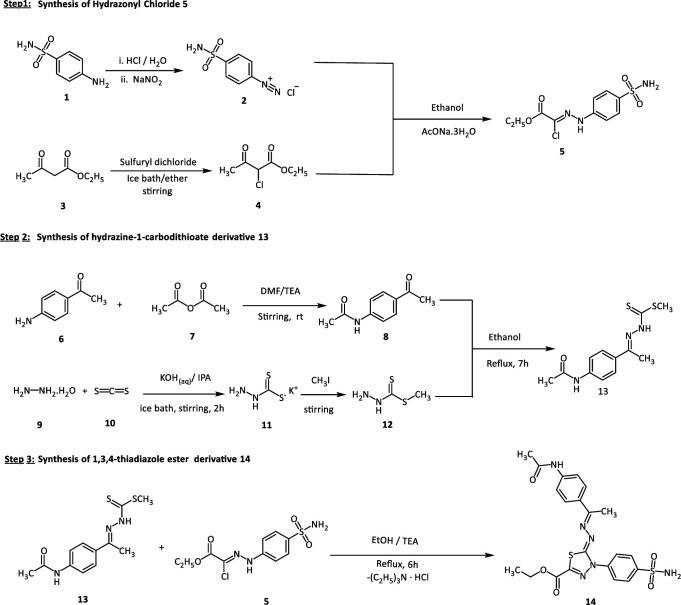
Synthetic pathway of compound 14.

The computational studies performed, encompassing DFT, molecular docking, and MD simulations, have validated the stability, favorable binding interactions, and reactive properties of compound **14**. Furthermore, its *in silico* ADMET analysis indicates promising pharmacokinetics and a favorable toxicity profile. Based on these findings, compound **14** has been synthesized according to Scheme 1 to experimentally evaluate these activities. Accordingly, the first stage in our synthesis, intermediate **4** was created by agitating ethyl acetoacetate and sulfuryl dichloride in anhydrous ethoxy ethane in a salt ice/NaCl bath [37]. Under basic conditions, coupling of intermediate **4** was implemented with 4-sulfamoylbenzenediazonium chloride **2** [obtained by diazotization of sulfanilamide **1** with the sodium salt of nitrous acid in the existence of hydrochloric acid at 0–5°C, succeeding in the protocol of the literature [38] to yield suitable hydrazonoyl chloride **5**. On the other hand, *N*-(4-acetylphenyl)acetamide **8** was synthesised from the acylation reaction of p-aminoacetophenone **6** with acetylating agents such as acetic anhydride at ambient temperature in dimethyl formamide and triethyl amine, which underwent condensation reaction with methyl hydrazinecarbodithioate **12** [39] to afford an important intermediate **13**. In the end, a cyclocondensation reaction between **13** and **5** under the EtOH/ TEA system achieved the target 1,3,4-thiadiazole ester derivative **14** with excellent yield.

The structural elucidation of the 1,3,4-thiadiazole ester derivative **14** was achieved through comprehensive characterization using FT-IR, ^1^H, and ^13^C NMR spectroscopy, and mass spectrometry. The FTIR spectrum displayed absorption bands assignable to the N–H and N-H_2_ groups at 3260, 3187, and 3120 cm ⁻ ¹, respectively. Both ester and amide carbonyl groups exhibited absorbance in the 1690–1665 cm ⁻ ¹ region; additionally, imine (C = N) group absorbance was observed near 1619 cm ⁻ ¹. Furthermore, S = O stretching vibrations for the sulfonamide group were observed at 1365 and 1166 cm ⁻ ¹. Whereas the ^1^H NMR exhibited the two singlet signals at *δ*_H_ = 10.10 and 7.42 ppm owing to protons of N-H and N-H_2_, respectively. Four doublet signals in the *δ*_H_ = 8.19–7.62 ppm range indicate the aromatic protons. Additionally, the signals corresponding to the ethyl group (CH_3_–CH_2_–) were observed as a quartet triplet pattern at *δ*_H_ = 4.35 and 1.29 ppm. Moreover, two singlet resonances, assigned to the protons of two methyl groups, appeared at *δ*_H_ = 2.36 and 2.03 ppm. The ^13^C NMR spectrum reveals distinct signals that correspond to the unique carbon atoms within the molecule. Notably, the carbon atoms from the 1,3,4-thiadiazole ring appear at *δ*_C_ values of 161.15 and 158.47 ppm. Additionally, two carbonyl carbon resonances in the range of *δ*_C_ = 170.00–162.00 ppm confirm the presence of both the ester and amide groups. Aromatic carbon signals can be observed between *δ*_C_ = 144.31 and 119.05 ppm. Furthermore, the carbons from the ethoxy group and two methyl groups are detected at *δ*_C_ = 63.47, 24.63, 15.89, and 14.50 ppm, respectively. The distinct peaks provide valuable information regarding the molecular structure and functional groups present. Overall, this analysis confirms the successful synthesis of the compound with the expected structural features. Ultimately, the Mass Spectrometry (MS) analysis of the compound revealed a molecular weight of 502.56 [M+], which aligns with the molecular formula C_21_H_22_N_6_O_5_S2.

### 3.3.Biological evaluations

#### 3.3.1.*In vitro* enzyme inhibition.

The inhibitory activities (IC₅₀ values) of compound **14**, Erlotinib, and Acetazolamide against EGFR, hCA_IX, and hCA_XII were presented in Table 4. Compound **14** exhibited strong EGFR inhibition with an IC₅₀ of 10.12 ± 0.29 nM, which is comparable to the clinically approved EGFR inhibitor Erlotinib (IC₅₀ = 7.74 ± 0.59 nM). This indicates that compound **14** holds promise as a potent EGFR-targeted therapeutic agent.

**Table 4:** Inhibitory Activities (IC₅₀) of compound 14, Erlotinib, and Acetazolamide Against EGFR, hCA_IX, and hCA_XII.

Comp	EGFR, IC_50_ (nM) ^a^ ± SEM	hCA_IX IC _50_ (nM) ^a^ ± SEM	hCA_XII IC _50_ (nM) ^a^ ± SEM
Compound **14**	10.12 ± 0.29	79 ± 0.3	58 ± 0.3
Erlotinib	7.74 ± 0. 59	–	–
Acetazolamide	–	86 ± 0.5	33 ± 0.2

^**a**^ Values are given as mean ± SEM of three independent experiments.

For hCA_IX and hCA_XII, compound **14** showed IC₅₀ values of 79 ± 0.3 nM and 58 ± 0.3 nM, respectively, demonstrating significant inhibitory activity against these isoforms. When compared to Acetazolamide, a standard human Carbonic Anhydrase inhibitor, compound **14** was more potent against hCA_IX (Acetazolamide IC₅₀ = 86 ± 0.5 nM) but was less effective against hCA_XII (Acetazolamide IC₅₀ = 33 ± 0.2 nM). Despite this, compound **14**’s ability to inhibit hCA_XII with considerable potency suggests its potential for targeting tumor environments characterized by hypoxia or high acidity, where these enzymes play critical roles.

The dual inhibitory activity of compound **14** against EGFR, hCA_IX and hCA_XII positions it as a promising candidate for dual-target therapies, particularly in cancers where EGFR signaling is dysregulated and hypoxic conditions drive tumor progression. This multifunctional activity could offer advantages in reducing tumor growth, overcoming resistance to single-target agents, and disrupting tumor microenvironment regulation.

#### 3.3.2. Cytotoxicity and safety.

The cytotoxicity data revealed key advantages of compound **14** over the reference compound, Acetazolamide, particularly in terms of selectivity for cancer cells. As Table 5 shows, while Acetazolamide exhibits strong cytotoxicity against the MDA-231 and MCF-7 cancer cell lines with IC₅₀ values of 7.64 ± 0.4 µM and 7.26 ± 0.3 µM, respectively, Compound **14** demonstrates promising cytotoxic activity with IC₅₀ values of 16.13 ± 1.2 µM and 22.57 ± 1.5 µM. This potency could be advantageous in reducing the risk of excessive cytotoxicity where precise targeting is essential. A significant advantage of compound **14** lies in its markedly lower cytotoxicity toward non-cancerous Vero cells, with an IC₅₀ of 148.32 ± 1.22 µM, compared to Acetazolamide’s IC₅₀ of 77.57 ± 0.57 µM. The higher IC₅₀ value for Vero cells suggests that compound **14** is more selective, potentially offering a better safety profile. This is crucial for minimizing adverse effects on healthy tissues during cancer treatment, a common limitation of many existing chemotherapeutic agents. Furthermore, the improved selectivity index of compound **14** indicates its potential to provide therapeutic benefits while reducing off-target toxicity.

**Table 5:** *In vitro* Cytotoxicity IC_50_ values of compound 14 and Acetazolamide.

Comp.	*In vitro* Cytotoxicity IC_50_ (µM) ^a^		
	MDA-231	MCF-7	Vero
Acetazolamide	7.64 ± 0.4	7.26 ± 0.3	77.57 ± 0.57
Cpmpound **14**	16.13 ± 1.2	22.57 ± 1.5	148.321.22

^**a**^ Values are given as mean ± SEM of three independent experiments.

#### 3.3.3. Real-time quantitative PCR.

The impact of compound **14** on the expression of critical apoptotic markers, such as BAX, Bcl-2, the BAX/Bcl-2 ratio, Caspase-8, and Caspase-9, was analyzed in MDA-MB-231 cells using real-time quantitative PCR as previously described [40] and presented in Table 6. In detail, compound **14** significantly upregulated the pro-apoptotic BAX gene (3.06 ± 0.18-fold) while simultaneously downregulating the anti-apoptotic Bcl-2 gene (0.55 ± 0.05-fold) compared to untreated control cells (set at 1.00-fold). This shift in the balance of BAX and Bcl-2 resulted in a marked increase in the BAX/Bcl-2 ratio (5.56 ± 0.69-fold), which is a critical determinant of mitochondrial-mediated apoptosis. These findings suggest that compound **14** strongly promotes pro-apoptotic signaling, favoring cell death pathways in cancer cells.

**Table 6:** Effect of compound 14 (16.13 µM) on Gene Expression and Apoptotic Markers in MDA-MB-231 Cells treated for 72 h.

Sample	Gene expression (Fold Change)^a^				
	BAX	Bcl-2	BAX/Bcl-2 ratio	Caspases-8	Caspases-9
MDA-231 cells	1.00 ± 0.03	1.00 ± 0.05	1.00 ± 0.08	1.00 ± 0..09	1.00 ± 0.13
Compound **14**	3.06 ± 0.18	0.55 ± 0.05	5.56 ± 0.69	2.37 ± 0.07	2.26 ± 0.15

^**a**^ Values are given as changes from the corresponding control (MDA-231 cells) group.

Data from three independent experiments represent the mean ± SEM, as the fold changes, with the control set to ‘1’.

Furthermore, compound **14** induced the activation of both Caspase-8 (2.37 ± 0.07-fold) and Caspase-9 (2.26 ± 0.15-fold), key initiator caspases involved in the extrinsic and intrinsic apoptotic pathways, respectively. This dual activation indicates that compound **14** may trigger apoptosis via both mitochondrial (intrinsic) and death receptor-mediated (extrinsic) pathways, amplifying its apoptotic effects.

The combined regulation of these apoptotic markers underscores the potency of compound **14** as an inducer of programmed cell death in MDA-MB-231 cells. Its ability to modulate both upstream signaling (BAX/Bcl-2 ratio) and downstream execution (Caspases-8 and −9) highlights its therapeutic potential, particularly for aggressive and apoptosis-resistant cancers such as triple-negative breast cancer.

#### 3.3.4. Apoptosis (flow cytometry).

Table 7 summarizes the effects of compound **14** on cell viability, apoptosis (early and late stages), and necrosis in MDA-MB-231 cells, compared to untreated control cells.

**Table 7:** Effect of compound 14 on stages of the cell death process in MDA-MB-231 cells after 48 h treatment.

Sample	Viable^a^	Apoptosis^a^		Necrosis^a^
		Early	Late	
MDA-MB-231	95.48	3.84	0.32	0.45
Compound **14**/ MDA-MB-231	19.20	22.50	58.27	0.03

^**a**^ Values represent stages of the cell death process in MDA-MB-231 cells treated with or without compound **14**.

In untreated MDA-MB-231 cells, the majority remained viable (95.48%), with minimal evidence of apoptosis (early: 3.84%, late: 0.32%) or necrosis (0.45%). This indicates that the basal apoptotic and necrotic activity in these cells is negligible under normal conditions.

**Fig 21:**
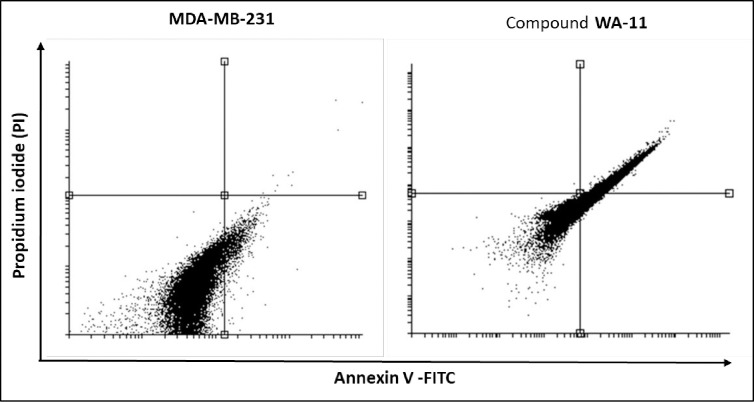
Impact of compound 14 (16.13 µM) on the progression of cell death stages in MDA-MB-231 cells following 48 hours of treatment: Viable (Left Bottom), Early Apoptosis (Right Bottom), Late (Right Top) and Necrosis (Left Top).

Treatment with compound **14** (Fig 21) resulted in a dramatic decrease in cell viability to 19.20%, reflecting its strong cytotoxic effect. Notably, this was accompanied by a significant increase in both early (22.50%) and late apoptosis (58.27%), suggesting that compound **14** predominantly induces programmed cell death in MDA-MB-231 cells. Interestingly, necrosis was almost absent (0.03%) following treatment, further supporting the specificity of compound **14** in promoting apoptotic pathways over uncontrolled cell death.

These findings highlight compound **14** as a potent inducer of apoptosis in MDA-MB-231 cells, favoring a regulated cell death mechanism. This is particularly advantageous in therapeutic contexts, as apoptosis minimizes inflammatory responses compared to necrosis, enhancing its potential for cancer treatment.

#### 3.3.5. Cell cycle (flow cytometry).

**Fig 22:**
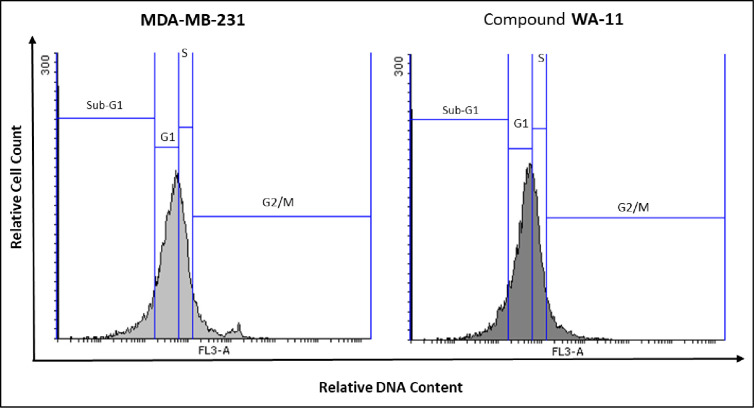
Impact of compound 14 (16.13 µM) on cell cycle progression in MDA-MB-231 cells following 48 hours of treatment.

The influence of compound **14** on the cell cycle distribution of MDA-MB-231 cells, in comparison to untreated controls, is presented in Table 8 and illustrated in Fig 22. In untreated MDA-MB-231 cells, the majority of cells were distributed in the G1 phase (44.98%), with smaller proportions in the S phase (24.95%) and G2/M phase (10.59%). A Sub-G1 population of 19.48% indicates a baseline level of apoptotic cells. Treatment with compound **14** resulted in a modest increase in the G1 phase population (49.10%) and a slight decrease in the proportions of cells in the S phase (23.78%) and G2/M phase (7.94%). This suggests that compound **14** may induce a mild G1 phase arrest, potentially interfering with the cell’s progression into DNA synthesis (S phase) and mitosis (G2/M phase). The Sub-G1 population (19.18%) remained comparable to the untreated control, indicating that while apoptosis occurs, the primary mechanism of compound **14**’s cytotoxicity may involve modulation of cell cycle progression.

**Table 8:** Effect of compound 14 on cell cycle progression in MDA-MB-231 cells after 48 h treatment.

Sample	Cell cycle distribution (%)^a^			
**%Sub-G1**	**%G1**	**%S**	**% G2/M**
MDA-MB-231	19.48	44.98	24.95	10.59
Compound **14**/ MDA-MB-231	19.18	49.10	23.78	7.94

^a^ Cell cycle distribution (%) of MDA-MB-231 cells were treated with or without compound **14**.

The observed G1 phase accumulation implies that compound **14** might exert its anti-proliferative effects by targeting proteins involved in G1/S transition, such as cyclins or cyclin-dependent kinases (CDKs). These effects may contribute to halting the uncontrolled proliferation characteristic of aggressive cancers like MDA-MB-231 cells.

## 4.Experimental

### 4.1.Computational studies

#### 4.1.1.DFT.

The structure and reactivity of compound **14** were optimized and investigated using Gaussian 09 (D.01) software employing the DFT method with the B3LYP functional and the 6–31 + G(d,p) basis set [41]. Detailed information and additional insights about this procedure can be found in the S1 Data.

#### 4.1.2.Molecular docking.

Molecular docking of compound **14** and the reference drugs against EGFR, hCA_IX, and hCA_XII was performed using MOE2014 and Discovery Studio 4.0 software [42]. Detailed information and additional insights about this procedure can be found in the S1 Data.

#### 4.1.3. MD simulations.

MD simulations were conducted for the hCA_IX–compound **14**, hCA_XII –compound **14**, and EGFR–compound **14** complexes using the CHARMM-GUI web server [32] and GROMACS 2021 [43,44]. The gmx_MMPBSA program applied the MM-GBSA approach to calculate the binding free energies of compound **14** [45,46]. PCA was performed to examine the dynamic motions of the alpha carbons in the selected amino acid sequences [47]. The free energy (Gα) of the protein in two specific states, defined by these coordinates, was linked to the likelihood of observing the protein’s free energy landscape (FEL) in those states through the exponential relationship [48]. Detailed information and additional insights about this procedure can be found in the S1 Data.

#### 4.1.4. ADMET and toxicity.

The pharmacokinetic and toxicity properties of Compound **14** were optimized using Discovery Studio 4.0 software [49]. Detailed information and additional insights about this procedure can be found in the S1 Data.

### 4.2. Chemistry

#### 4.2.1. General procedure for the synthesis of the 1,3,4-thiadiazole-sulfonamide derivative (14).

The 1,3,4-thiadiazole derivative **14** was synthesized by reacting intermediate **13** with ethyl (*Z*)-2-chloro-2-(2-(4-sulfamoyl phenyl)hydrazineylidene)acetate 5 in a 1:1 ratio, utilizing an alcoholic medium of 25 ml ethanol. The reaction was conducted under magnetic stirring and reflux, with a catalytic amount of TEA as a base, over a period of 6 hours. The progress of the reaction and the formation of the final product was closely monitored using thin-layer chromatography (TLC) with a solvent system of dichloromethane and methanol (9.5:0.5 to 5:5). Once the reaction was completed, heating was ceased, and the mixture was allowed to cool to room temperature. The resulting precipitate was then filtered, washed with absolute ethanol, air-dried, and recrystallized using a suitable solvent, yielding a pure product.

#### 4.2.2. Ethyl (*Z*)-5-(((*E*)-1-(4-acetamidophenyl)ethylidene)hydrazineylidene)-4-(4-sulfamoylphenyl)-4,5-dihydro-1,3,4-thiadiazole-2-carboxylate 14.

Yellow powder (Yield 78%), melting point 202−204ºC; IR (KBr, υ_max_/cm^-1^): FT-IR (v max, cm^-1^): 3260 cm^-1^ (NH), 3187, 3120 cm^-1^ (NH_2_), 3028 cm^-1^ (aromatic C-H), 2989 cm^-1^ (aliphatic C-H), 1686 cm^-1^ (ester, C = O), 1666 cm^-1^ (amide, C = O), 1619 cm^-1^ (C = N), and 1365, 1166 cm^-1^ (sym. and asym. SO_2_). ^1^H NMR (500 MHz, DMSO-*d*_6_) *δ* 10.10 (s, 1H, NH), 8.19 (d, *J* = 8.9 Hz, 2H, AB-Ar-H), 7.95 (d, *J* = 8.9 Hz, 2H, AB-Ar-H), 7.78 (d, *J* = 8.8 Hz, 2H, AB-Ar-H), 7.62 (d, *J* = 8.7 Hz, 2H, AB-Ar-H), 7.42 (s, 2H, NH_2_), 4.35 (q, *J* = 7.1 Hz, 2H, OC**H**_**2**_CH_3_), 2.36 (s, 3H, CH_3_), 2.03 (s, 3H, CH_3_), 1.29 (t, *J* = 7.1 Hz, 3H, OCH_2_C**H**_**3**_). ^13^C NMR (125 MHz, DMSO-*d*_6_) *δ* 169.12, 163.89, 161.15, 158.47, 144.31, 142.22, 141.70, 141.59, 132.03, 127.73, 127.35, 121.97, 119.05, 63.47, 24.63, 15.89, 14.50; MS (m/z)**=** 502 [%]:[M+, (17.28%)], Anal. Calcd for C_21_H_22_N_6_O_5_S2 (502.56): C, 50.19; H, 4.41; N, 16.72; Found: C, 50.32; H, 4.53; N, 16.86%.

### 4.3. *In vitro* studies

#### 4.3.1. Enzyme inhibition.

The *in vitro* inhibitory activity of compound **14** and references against EGFR, hCA_IX, and hCA_XII were evaluated using assay kits (BPS Bioscience, USA) at various concentrations: 1000, 300, 100, 30, 10, 3, 1, and 0.3 nM [50]. Detailed information and additional insights about this procedure can be found in the S1 Data.

#### 4.3.2. Cytotoxicity.

The MTT procedure [51,52] was utilized to evaluate the cytotoxicity and selectivity of compound **14** and the reference drug, Acetazolamide, against cancer cell lines (MDA-MB-231 and MCF-7) as well as non-cancerous Vero cell lines. Detailed information and additional insights about this procedure can be found in the S1 Data.

#### 4.3.3. RT-QPCR Studies.

The mRNA levels of BAX, Bcl-2, Caspase-8, and Caspase-9 in control MDA-MB-231 cells and those treated with compound **14** at the 16.13 µM concentration were measured using qRT-PCR, following previously described methods [40]. Detailed information and additional insights about this procedure can be found in the S1 Data.

#### 4.3.4. Flow cytometry.

The apoptotic properties of compound **14** (16.13 µM) and its effects on the cell cycle of MDA-MB-231 were assessed using flow cytometry analysis, following previously described methods [53]. Detailed information and additional insights about this procedure can be found in the S1 Data.

## 5. Conclusion

In conclusion, compound **14** is a promising dual inhibitor targeting EGFR and human carbonic anhydrases hCA_IX and hCA_XII, demonstrating potent anti-cancer activity and a favorable safety profile. The compound’s efficacy *in vitro*, as evidenced by its ability to significantly reduce cell viability and induce apoptosis in MDA-MB-231 breast cancer cells, supports its potential as a therapeutic agent for peripheral cancers. The computational studies, including DFT, molecular docking, and MD simulations, provide strong evidence for the compound’s stability, reactivity, and binding affinity, which correlate well with the observed biological activities. Although compound **14** shows moderate cytotoxicity with IC_50_ values of 16.13 µM in MDA-MB-231cells and 22.57 µM in MCF-7 cells, it exhibits selectivity against cancer cells over normal cells, as demonstrated by its higher IC_50_ in Vero cells. The favorable toxicological profile, including low carcinogenic risk and non-mutagenic properties, coupled with mild ocular irritancy and no skin irritancy, suggests that compound **14** has a promising therapeutic index. Further optimization of its pharmacokinetic properties, particularly improving solubility and absorption, will be key to enhancing its clinical applicability. These findings justify continued development and preclinical studies to explore the full therapeutic potential of compound **14** in cancer treatment.

## Supporting information

S1 Data The supporting information includes detailed synthetic procedures, in silico and in vitro studies, spectral characterization data, and a comprehensive toxicity report. (PDF)
