# Supplementary material for: A novel sulfamoylphenyl-dihydro-thiadiazole derivative as a dual EGFR and carbonic anhydrase inhibitor for cancer therapy
Source: PLoS One. 2025 Sep 4;20(9):e0328305. doi: 10.1371/journal.pone.0328305 (PMC12410758; doi:10.1371/journal.pone.0328305)
Supplement: S1 Data — (PDF) [file pone.0328305.s001.pdf]

A Novel Sulfamoylphenyl-Dihydro-Thiadiazole Derivative as a Dual EGFR and Carbonic Anhydrase Inhibitor for Cancer Therapy

Ibrahim H. Eissa[a*], Hazem Elkady[a], Walid E. Elgammal[b], Hazem A. Mahdy[a], Dalal Z. Husein[c], Fatma G.Amin[d], Asmaa A. A. Elsheshiny[e], Shimaa. S. Abdelfattah[f], Aisha A. Alsfouk[g], Eslam B. Elkaeed[h], Ahmed M. Metwaly[i*]

[a] Pharmaceutical Medicinal Chemistry & Drug Design Department, Faculty of Pharmacy (Boys), Al-Azhar University, Cairo, 11884, Egypt.

[b] Department of Chemistry, Faculty of Science, Al-Azhar University, Nasr City, Cairo, Egypt

[c] Chemistry Department, Faculty of Science, New Valley University, El-Kharja 72511, Egypt.

[d] Physics Department, Faculty of Science, Alexandria University, Alexandria, Egypt.

[e] Biophysics branch, Physics department, Faculty of science (girls), Al-azhar university, Nasr city, Cairo, Egypt.

[f] Physics Department, Faculty of Science, Al-Azhar University (Girl's Branch), Cairo, 11754, Egypt

[g] Department of Pharmaceutical Sciences, College of Pharmacy, Princess Nourah bint Abdulrahman University, P.O. Box 84428, Riyadh 11671, Saudi Arabia.

[h] Department of Pharmaceutical Sciences, College of Pharmacy, AlMaarefa University, Riyadh 13713, Saudi Arabia.

[i] Pharmacognosy and Medicinal Plants Department, Faculty of Pharmacy (Boys), Al-Azhar University, Cairo 11884, Egypt.

***Corresponding authors:**

Ibrahim H. Eissa

Medicinal Chemistry Department, Faculty of Pharmacy (Boys), Al-Azhar University, Cairo 11884, Egypt**.**

Email: Ibrahimeissa@azhar.edu.eg

Ahmed M. Metwaly

Pharmacognosy and Medicinal Plants Department, Faculty of Pharmacy (Boys), Al-Azhar University, Cairo 11884, Egypt**.** **Email:** ametwaly@azhar.edu.eg

Hazem Elkady: Medicinal Chemistry Department, Faculty of Pharmacy (Boys), Al-Azhar University, Cairo 11884, Egypt. **Email:** Hazemelkady@azhar.edu.eg

Content
**S1. Chemistry**
**S2. Biological testing**
**S.3. *In silico* studies**
**S.4. Spectral data**

S.1. Chemistry

The chemicals, reagents, and reaction solvents used in this study were gained from Sigma-Aldrich, Alpha Chem, Fluka, and Loba and were not purified further. The melting points (mp), which have not been corrected, were measured using the SMP50 Digital Melting Point App provided by Bibby Scientific in Staffordshire. A Thermo Fisher Nicolet IS10 spectrophotometer was used to detect infrared spectra as solids on the potassium bromide disc (ν_{max} in cm^{-1}) with a resolution of 4.0 cm^{-1}, covering 4000-400 cm^{-1}.^{1}H-NMR and ^{13}C-NMR spectra (400 and 101 MHz) were recorded at the JNM-ECA 500 II Made by JEOL-JAPAN instrument through a solution of deuterated dimethyl sulfoxide. Proton chemical shifts are labeled in part per million (ppm), downfield from tetramethyl silane (TMS, δ=0) as an internal standard, and the following abbreviations (or a combination thereof) are used to describe splitting patterns: s, singlet; d, doublet; t, triplet; q, quartet; m, multiplet, and br, broad. Mass spectra were measured with a Thermo Scientific GCMS model (Isq Lt) using the Thermo X-Calibur software (Shimadzu, Kyoto, Japan) at the Regional Center for Mycology and Biotechnology (RCMB), Al-Azhar University, Nasr City, Cairo, Egypt. Elemental studies were conducted at the Regional Center for Microbiology and Biotechnology, Al-Azhar University, Cairo, Egypt, with results accurate to within 0.4%. Thin-layer chromatography (TLC) was carried out on silica gel plates by using DCM: MeOH (95:5%), as the eluting system. The progress of the reaction and evaluation of product purity was determined using a UV indicator at 254 nm.

S2. Biological testing

S.2.1. *In vitro* anti-proliferative activity

<u>Materials and methods</u>

<u>Cell line</u>

Mammary gland breast cancer (MCF-7) and (MDA-MB-231). The cell line was obtained from ATCC via Holding company for biological products and vaccines (VACSERA), Cairo, Egypt.

Sorafenib was used as a standard anticancer drug for comparison.

<u>Chemical reagents</u>

The reagents RPMI-1640 medium , MTT and DMSO (sigma co., St. Louis, USA), Fetal Bovine serum (GIBCO, UK) .

<u>MTT assay</u>

The cell lines mentioned above were used to determine the inhibitory effects of compounds on cell growth using the MTT assay. This colorimetric assay is based on the conversion of the yellow tetrazolium bromide (MTT) to a purple formazan derivative by mitochondrial succinate dehydrogenase in viable cells. Cell lines were cultured in RPMI-1640 medium with 10% fetal bovine serum. Antibiotics added were 100 units/ml penicillin and 100µg/ml streptomycin at 37 C in a 5% Co_2 incubator. The cell lines were seedesin a 96-well plate at a density of $1.0x10^4$ cells/well. at 37 C for 48 h under 5% Co_2. After incubation the cells were treated with different concentration of compounds and incubated for 24 h. After 24 h of drug treatment, 20 µl of MTT solution at 5mg/ml was added and incubated for 4 h. Dimethyl sulfoxide (DMSO) in volume of 100 µl is added into each well to dissolve the purple formazan formed. The colorimetric assay is measured and recorded at absorbance of 570 nm using a plate reader (EXL 800 ,USA). The relative cell viability in percentage was calculated as (A570 of treated samples/A570 of untreated sample) X 100.

***Average of Relative viability of cells (%)**

Conc.(µM)	MDA-231	MCF-7
	ACZ	
100	6.9	8.1
50	16.7	15.2
25	24.3	23.0
12.5	32.1	34.8
6.25	55.9	51.6
3.125	70.2	68.9
1.56	88.5	87.5

Conc.(µM)	MDA-231	MCF-7
	WA-11	
100	18.5	24.4
50	26.1	35.1
25	38.6	46.5
12.5	51.0	57.6
6.25	69.9	71.7
3.125	86.3	90.3
1.56	100	100

S.2.2. Safety assay

Materials and methods

Cell line

The cell line (Vero) was obtained from ATCC via Holding company for biological products and vaccines (VACSERA) , Cairo, Egypt.

Chemical reagents

The reagents RPMI-1640 medium , MTT and DMSO (sigma co., St. Louis, USA), Fetal Bovine serum (GIBCO, UK) .

Sorafenib was used as a standard anticancer drug for comparison.

MTT assay

The different cell line mentioned above were used to determine the inhibitory effects of compounds on cell growth using the MTT assay. This colorimetric assay is based on the conversion of the yellow tetrazolium bromide (MTT) to a purple formazan derivative by mitochondrial succinate dehydrogenase in viable cells. The cells were cultured in RPMI-1640 medium with 10% fetal bovine serum. Antibiotics added were 100 units/ml penicillin and 100µg/ml streptomycin at 37 C in a 5% Co_2 incubator. The cellswere seedesin a 96-well plate at a density of $1.0x10^4$ cells/well.at 37 C for 48 h under 5% Co_2. After incubation the cells were treated with different concentration of compounds and incubated for 24 h. After 24 h of drug treatment, 20 µl of MTT solution at 5mg/ml was added and incubated for 4 h. Dimethyl sulfoxide(DMSO) in volume of 100µl

is added into each well to dissolve the purple formazan formed. The colorimetric assay is measured and recorded at absorbance of 570nmusing a plate reader (EXL 800, USA). The relative cell viability in percentage was calculated as (A570 of treated samples/A570 of untreated sample) X 100.

S.2.3. *In vitro* EGFR inhibition

• The in vitro inhibitory activity of the tested compounds against EGFR was accomplished using EGFR Kinase Assay Kit (BPS Bioscience, USA) at different dilutions of 1000, 300, 100, 30, 10, 3, 1, and 0.3 nM.

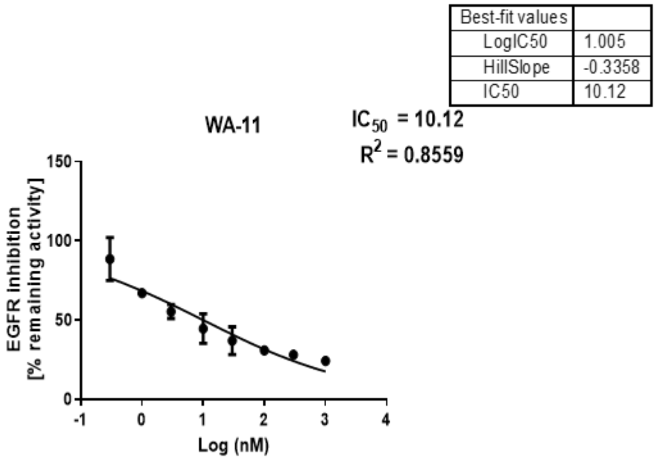

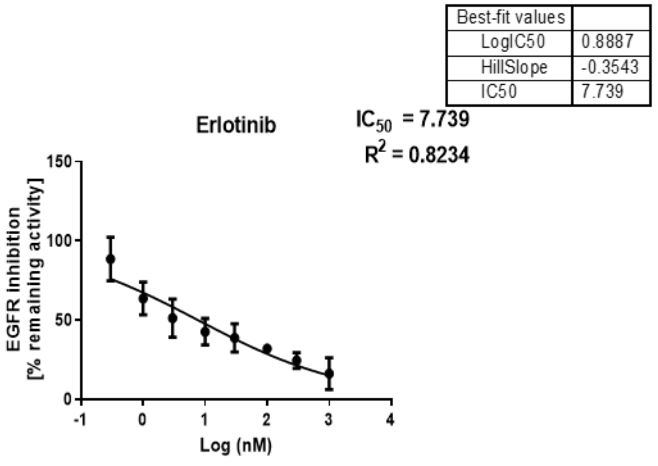

S.2.4. *In vitro* carbonic anhydrase inhibition

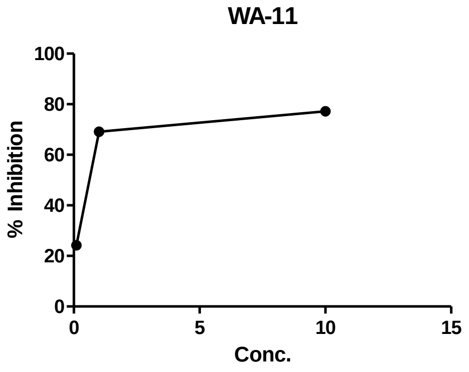

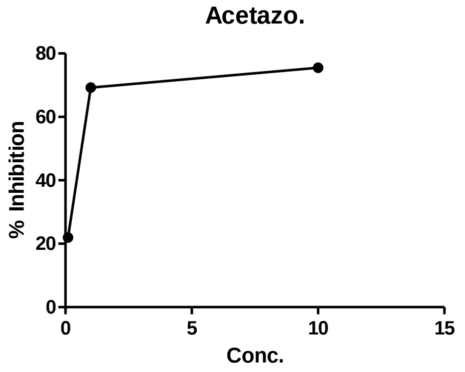

***In vitro* carbonic anhydrase (IX) inhibition**

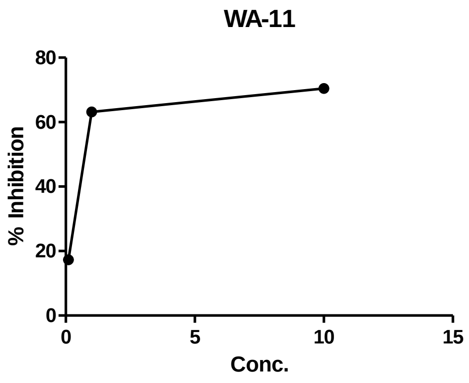

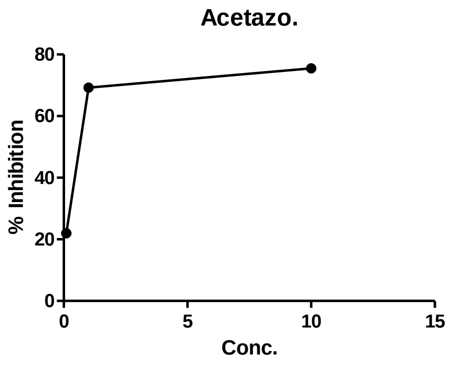

***In vitro* carbonic anhydrase (XII) inhibition**

S.2.4. Cell migration and wound healing assay

MDA-MB-231cells were grown to 95.0% confluency in a complete DMEM medium and then the wounds were formed using a plastic tip. After washing with pre-warmed PBS, the cells were incubated in the specific medium or compound **14** (16.13 µM) treatment. After incubation at 37°C and 5.0% CO_2 for 24h, the cells were washed with PBS and the wounds distance was determined as the scratch width of the treated and untreated groups using ImageJ software.

S.2.5. Cell cycle analysis

This assay was conducted according to the reported methods. In short, MDA-MB-231cells were seeded into six-well plates (2 × 105 cells per each well) and incubated for 24 h at 37 °C and 5% CO2. After that, they were treated with 16.13 µM of compound **14** dissolved in DMSO (1% v/v) for 48 h. After the cells were washed several times with cold phosphate buffered saline (PBS), fixed with ethyl alcohol (70%) and rinsed again with PBS, they were stained with the DNA fluorochrome propidium iodide and kept at 37 °C for 15 min in the dark. The cell distribution was analyzed using Epics XL-MCL™ Flow Cytometer (Beckman Coulter), and the data were analyzed using Flowing software (version 2.5.1, Turku Centre for Biotechnology, Turku, Finland).

S.2.6. Apoptosis analysis

Apoptosis detection was carried out using Annexin V fluorescein isothiocyanate (V-FITC)/PI kit according to the reported procedures. MDA-MB-231cells cells were seeded and incubated for 24 h, then treated with (16.13 µM) of compound **14** for 48 h. The cells were then collected, washed 3 times with PBS, fixed with ice-

cold absolute ethanol (70%) and stained with Annexin-V-FITC/propidium iodide (PI) using a double staining kit for 20 min in the dark. Epics XL-MCL™ Flow Cytometer was used to evaluate the apoptosis.

S.2.7. Quantitative Reverse Transcription-Poly¬merase Chain Reaction (qRT-PCR) Analysis

Cells were seeded in T-25 cm2 tissue culture flasks 24h before treatment with DMSO vehicle (0.01%) or compound **14 (**16.13 µM) for an additional 72 h. Total RNA was extracted, reverse transcribed, and assayed for BAX, Bcl-2, caspases-8, and caspases-9 genes expression by quantitative RT-PCR. ß-actin serves as the housekeeping gene. The quantity of BAX, Bcl-2, Caspase-8 and Caspase-9 mRNA in control and compound **14** (16.13 µM)-treated MDA-231 cells was assessed by qRT-PCR. Total RNA from vehicle-treated control (0.01% DMSO) and **14-**treated MDA-231 cells were extracted as-per the manufacturer instructi¬ons (RNeasy mini kit, Qiagen, Germany). After RNA extraction, cDNA was prepared using the Revert Aid First Strand cDNA Synthesis kit (Thermo Scientific, USA). Amplification of target cDNA for apoptosis markers and ß-actin [as a normalization (housekeeping) gene] was done using one-step RT-PCR SYBR® Green kit Master Mix (Bio-Rad Laboratories, USA) on Rotor-Gene Q real-time PCR thermal cycler instrument. cDNA (2 µl aliquots) was mixed with 1 µl of forward primer, 1 µl reverse primer (Table below), 10 µl master mixture, and the reaction volume was completed to 20 µl with nuclease-free water. All experiments were performed in triplicate.

Sequence of qRT-PCR primers, forward (F) and reverse (R), used in the current study.

**BAX**	F: 5′-CAAACTGGTGCTCAAGGC-3′<br>R: 5′-CACAAAGATGGTCACGGTC-3′
**BCL2**	F: 5′-GTACTTAAAAAATACAACATCACAG-3′<br>R: 5′-CTTGATTCTGGTGTTTCCC-3′
**Caspase 8**	F: 5'- AGAGTCTGTGCCCAAATCAAC -3 '<br>R: 5'- GCTGCTTCTCTCTTTGCTGAA -3'
**Caspase-9**	F: 5′-CATTTCATGGTGGAGGTGAAG-3′<br>R: 5′-GGGAACTGCAGGTGGCTG-3′
**Beta-actin**	F: 5′-CTTCCTTCCTGGGCATG-3′<br>R: 5′-GTCTTTGCGGATGTCCAC-3′

S3. *In silico* studies

S.3.1. Docking studies

Protein Preparation: The crystal structure of VEGFR-2 [PDB ID: 2OH4, resolution: 2.05 Å] was obtained from Protein Data Bank (https://www.rcsb.org). At first, the crystal structure of the VEGFR-2 complexed with the co-crystallized ligand was prepared by removing crystallographic water molecules. Only one chain was retained besides the co-crystallized ligand. The selected protein chain was protonated using the following setting. The used electrostatic functional form was GB/VI with a distance cut-off of 15 Å. The used value of the dielectric constant was 2 with an 80 dielectric constant of the used solvent. The used Van der Waals functional form was 800R3 with a distance cut-off of 10 Å. Then, the energy of the protein chain was minimized using Hamiltonian AM1 implanted in Molecular Operating Environment (MOE 2019 and MMFF94x (Merck molecular force field) for structural optimization. Next, the active site of the target protein was defined for ligand docking and redocking (in case of validation of docking protocol). The active site of the protein was identified as the residues that fall within the 5 Å distance from the perimeter of the co-crystallized ligand.

Ligand Preparation: 2D structures of the synthesized compounds and the standard compound, sorafenib were drawn using ChemBioDraw Ultra 14.0 and saved in MDL-SD file format. The 3D structures of the ligands were protonated, and the structures were optimized by energy minimization using MM2 force-field and 10000 iteration steps of 2 fs. The conformationally optimized ligands were used for docking studies.

Docking Setup and Validation of Docking Protocol: The protein-ligand docking studies were carried out using MOE version 2019. Validation of the docking protocol was carried out by redocking the co-crystallized reference ligand against the isolated pocket of VEGFR-2. The docking protocol was validated by comparing the heavy atoms RMSD value of the re-docked ligand pose with the corresponding co-crystallized reference ligand structure.

The docking setup for the tested compounds was established according to the protocol followed in the validation step. For each docking run, 30 docked solutions were generated using ASE for scoring function and rigid receptor for refinement. The pose with ideal binding mode was selected for further investigations. The docking results were visualized using Discovery Studio (DS) 4.0. Analysis of the docking results was carried out by comparing the interactions and docking score obtained for the docked ligands with that of the re-docked reference molecule.

S.3.2. MD simulations

Molecular Dynamic (MD) Simulation:

An unbiased molecular dynamics (MD) simulation was used to evaluate the binding affinity and stability of the VEGFR-2_**14** complex. A 200 ns trajectory was made for the system using GROMACS 2021. CHARMM-GUI solution builder was used to prepare starting files. The system was solvated using water molecules with a transferable intermolecular potential of three points (TIP3P) in a 10 nm cubic box with a buffer distance of 1 nm. Neutralization was achieved by adding NaCl ions at a concentration of 0.154 M. The CHARMM36m force field was used for the VEGFR-2 protein, TIP3P water, and ions.

All simulation dimensions employed periodic boundary conditions. Potential energy minimization with the steepest descent method was first performed to remove steric conflicts. When the highest force on any atom fell below 100 kJ/(mol·nm) or after 100,000 steps, convergence was reached. Thermodynamic equilibrium was the aim of the two-step equilibration procedure. The average temperature in the first stage (NVT ensemble) was maintained at 310 K by the Velocity Rescale thermostat. The second stage (NPT ensemble) used the Berendsen barostat to regulate pressure at 1 atm and the Velocity Rescale algorithm to maintain a temperature of 310 K. A Parrinello-Rahman barostat was used to regulate pressure at 1 atm, and a Nose-Hoover thermostat was used for temperature control in the production run (NPT ensemble). The hydrogen atom bond lengths were maintained with the LINear Constraint Solver (LINCS) method. Using a 1.2 nm cutoff, the Particle Mesh Ewald (PME) approach was used to calculate electrostatic interactions.

The leap-frog integrator propagated the Newtonian equations of motion using time steps of 1 fs for equilibration and 2 fs for production. Two thousand simulation frames were obtained at intervals of 0.1 ns.

After periodic boundary conditions were eliminated and protein integrity was restored, VMD TK scripts were used to analyse the trajectories. Many calculations were performed, such as the Root Mean Square Deviation (RMSD) of the VEGFR-2 backbone and the ligand system **14**. Other structural features that were evaluated included the number of hydrogen bonds between ligands and proteins, center of mass distances between ligands and proteins, radius of gyration (RoG), solvent accessible surface area (SASA), and root mean square fluctuation (RMSF). Protein-Ligand Interaction Fingerprints (ProLIF), a Python program, assesses interactions between ligands and amino acids quantified and characterized the frequency and type of interactions in each frame.

Binding free energy calculation using MM-GBSA:

The ligands' binding free energies were ascertained by applying the Molecular Mechanics/Generalized Born Surface Area (MM-GBSA) method through the gmx_MMPBSA program. To determine the contributing amino acids within a 1 nm radius of the ligand, a decomposition analysis was carried out. The ionic strength of 0.154 M and the solvation parameter (igb) of 5 were used in the simulations. The values of 1.0 and 78.5 were selected for the internal and external dielectric constants, respectively. Equation 1 describes in detail the basic free energy calculation approach.

$$\Delta G = \langle Gcomplex - (Greceptor + Gligand)\rangle \quad \text{Equation 1}$$

where < > is the mean of the enclosed free energy of the ligand, complex, and receptor throughout the computation frames. We employed the entire route (a total of 2000 frames) in our method. Equations 2 through 6 can be used to compute various energy terms in the following ways:

$$\Delta Gbinding = \Delta H - T\Delta S \quad \text{Equation 2}$$

$$\Delta H = \Delta Egas + \Delta Esol \quad \text{Equation 3}$$

$$\Delta Egas = \Delta Eele + \Delta EvdW \quad \text{Equation 4}$$

$$\Delta Esolv = EGB + ESA \quad \text{Equation 5}$$

$$ESA = \gamma.SASA \quad \text{Equation 6}$$

Where:

ΔH is the enthalpy which can be calculated from gas-phase energy (E_{gas}) and solvation-free energy (E_{sol}). -$T\Delta S$ is the entropy contribution to the free binding energy. E_{gas} is composed of electrostatic and van der Waals terms; E_{ele}, E_{vdW}, respectively. E_{sol} can be calculated from the polar solvation energy (E_{GB}) and nonpolar solvation energy (E_{SA}) which is estimated from the solvent-accessible surface area.

Principal Component Analysis:

The mobility of the α-carbon of VEGFR-2 was evaluated using Principal Component Analysis (PCA) and the mass-weighted covariance matrix (C). The final equilibrium frame of the VEGFR-2_**14** system for combined analysis (for the free energy landscape) and the final equilibrium frame of each trajectory for individual trajectories are referred to during the alignment process. Using gmx covar to diagonalize the covariance matrix in GROMACS, eigenvectors representing the dominating atomic motions were found. Eigenvalues indicated the amount of motion; the first principal component captured the largest variance, while

the contributions from the other components decreased. The gmx anaeig command was utilized for analysis with GROMACS. We computed the dimensions of the critical subspace using three parameters. 1) The variance gathered by each additional eigenvector was visualized, and the cumulative sum of the eigenvalues was calculated using extra eigenvectors. Furthermore, 2) a scree plot was generated, which displayed each eigenvalue in relation to its corresponding eigenvector index. The index with the largest slope reduction indicated the critical subspace size. Moreover, 3) the distribution of the eigenvectors was evaluated.

When a distribution other than a Gaussian distribution emerged, it indicated that the corresponding eigenvectors had meaningful dynamics.

We computed the cosine content (ci) of each eigenvector of the C matrix, which may take values ranging from 0 (no cosine) to 1 (perfect cosine). The following is the cosine content equation:

$$c_i = \frac{2}{T} \left(\int \cos(i\pi t\, p_i(t)\, dt\right)^2 \left(\int p_i^2\, (t)\, dt\right)^{-1}$$

Where T is the time of the simulation. Abnormally large ci values, which represent random motion, are related to insufficient sampling. When the cosine content of the first few PCs is near 1, the behavior of proteins on a large scale is analogous to diffusion. Accordingly, the first 10 PCs were used to calculate their cosine content.

Free Energy Landscape (FEL):

A protein's free-energy landscape (FEL) can be used to determine the different conformations it can adopt, which is necessary to understand the protein's dynamics and function. In this investigation, conformational sampling methods are often used, such as molecular dynamics (MD) simulations, which mimic the intrinsic temperature changes in proteins over time. Here, we have revealed the FEL of our protein of interest through molecular dynamics simulations. To improve its visual appeal, we projected this complex onto two chosen response coordinates. The free energy (Gα) of the protein in a state described by these coordinates is related to the probability of detecting the protein in that state via the exponential relationship e-Gα/kT. These computations can be used to determine the entire FEL:

$$G\alpha = -kT \ln\left(\frac{P(q_\alpha)}{P_{max}(q)}\right)$$

Where k is the Boltzmann constant, T is the temperature of simulation, $P(q_\alpha)$ is an estimate of the probability density function obtained from a histogram of the MD data and $P_{max}(q)$ is the probability of the most probable state. We derived two-dimensional representations of the free-energy landscapes by considering two separate reaction coordinates: qi and qj. These representations were obtained by analyzing the joint probability distributions, referred to as P(qi,qj), which describe the system. The GROMACS gmx sham command was utilized for this purpose.

S.3.4. Density Function Theory (DFT) calculations

DFT calculations

The Gaussian 9 package under B3LYP/6-311+G (d, p) level has been used to calculate density function theory (DFT). Several tools have been used, including Avogadro, GaussView 0.3, and Multiwfn. The following formulas were used to calculate the reactivity descriptors: ionization energy (I), chemical potential (μ), electron affinity (A), electrophilicity (χ), maximal charge acceptance (ΔNmax), softness (σ), and hardness (η):

$IP = -E_{HOMO}$

$EA = -E_{LUMO}$

$\mu = (IP+EA)/2$

$\eta = (IP-EA)$

$\chi = -\mu$

$\omega = \mu^2/(2\eta)$

$\sigma = 1/\eta$

$\Delta N = -(\mu/\eta)$

$\Delta E = -\omega$

$E_{gap} = E_{LUMO} - E_{HOMO}$

S.3.5. ADMET studies

ADMET descriptors (absorption, distribution, metabolism, excretion and toxicity) of the compounds were determined using Discovery studio 4.0. Sorafenib was used as a reference molecule. At first, the CHARMM force field was applied then the tested compounds were prepared and minimized according to the preparation of small molecule protocol.

- **Preparation of the tested compounds:**

In this protocol, the general-purpose panel was utilized with the activation of the Prepare ligand option. The change ionization was switched on the true option using the Rule based as an ionization method. In Rule based task, we used the carboxylate as an acid ionization. Additionally, the primary, secondary, and tertiary amines were selected as Base ionization. The ionization enumeration option was switched on the one protomer. Under the filter smart option, we selected all options. The false option was selected for tasks Generate tautomers, generate isomers, Fix bad valencies, and parallel processing. The generate coordinates task was switched on the 3D option. Finally, the duplicate structure task was activated on the remove option.

- **Running of ADMET protocol**

In this protocol, the small molecules panel was utilized with the activation of the ADMET descriptors option. Then, we selected the prepared compounds as the input ligands. Further, all the ADMET parameters (aqueous solubility, Blood brain barrier, intestinal absorption, CYP2D6, and plasma protein binding) were selected. Then, the output of the running protocol was visualized to give the ADMET chart.

S.3.6 Toxicity studies

The toxicity parameters of the synthesized compounds were calculated using Discovery studio 4.0. Sorafenib was used as a reference molecule. At first, the CHARMM force field was applied then the compounds were prepared and minimized according to the preparation of small molecule protocol.

- **Preparation of the tested compounds:**

In this protocol, the general-purpose panel was utilized with the activation of the Prepare ligand option. The change ionization was switched on the true option using the Rule based as an ionization method. In Rule based task, we used the carboxylate as an acid ionization. Additionally, the primary, secondary, and tertiary amines were selected as Base ionization. The ionization enumeration option was switched on the one protomer. Under the filter smart option, we selected all options. The false option was selected for tasks Generate tautomers, generate isomers, Fix bad valencies, and parallel processing. The generate coordinates task was switched on the 3D option. Finally, the duplicate structure task was activated on the remove option.

- **Running of Toxicity protocol**

In this protocol, the small molecules panel was utilized with the activation of the toxicity prediction (extensible) option. Then, we selected the prepared compounds as the input ligands. Further, the different toxicity models were selected from the model panel. The similarity search task was activated to be true. The detailed report task was switched on as a PDF file. Then, the output of the running protocol was visualized to give the toxicity PDF report.

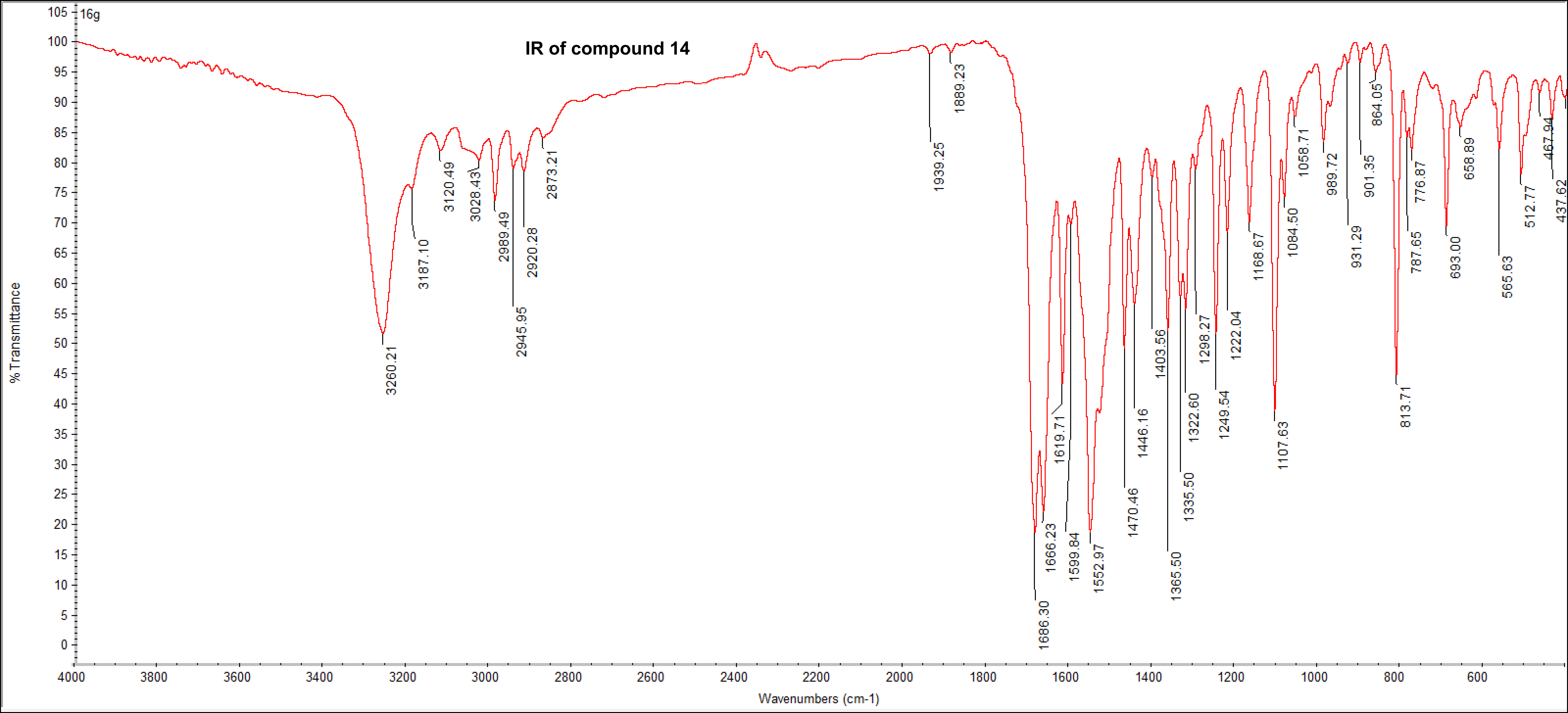
16g
IR of compound 14
%Transmittance
Wavenumbers (cm-1)
3260.21
3187.10
3120.49
3028.43
2989.49
2945.95
2920.28
2873.21
1939.25
1889.23
1686.30
1666.23
1619.71
1599.84
1552.97
1470.46
1446.16
1403.56
1365.50
1335.50
1322.60
1298.27
1249.54
1222.04
1168.67
1107.63
1084.50
1058.71
989.72
931.29
901.35
864.05
813.71
787.65
776.87
693.00
658.89
565.63
512.77
467.94
437.62

1H NMR of compound 14

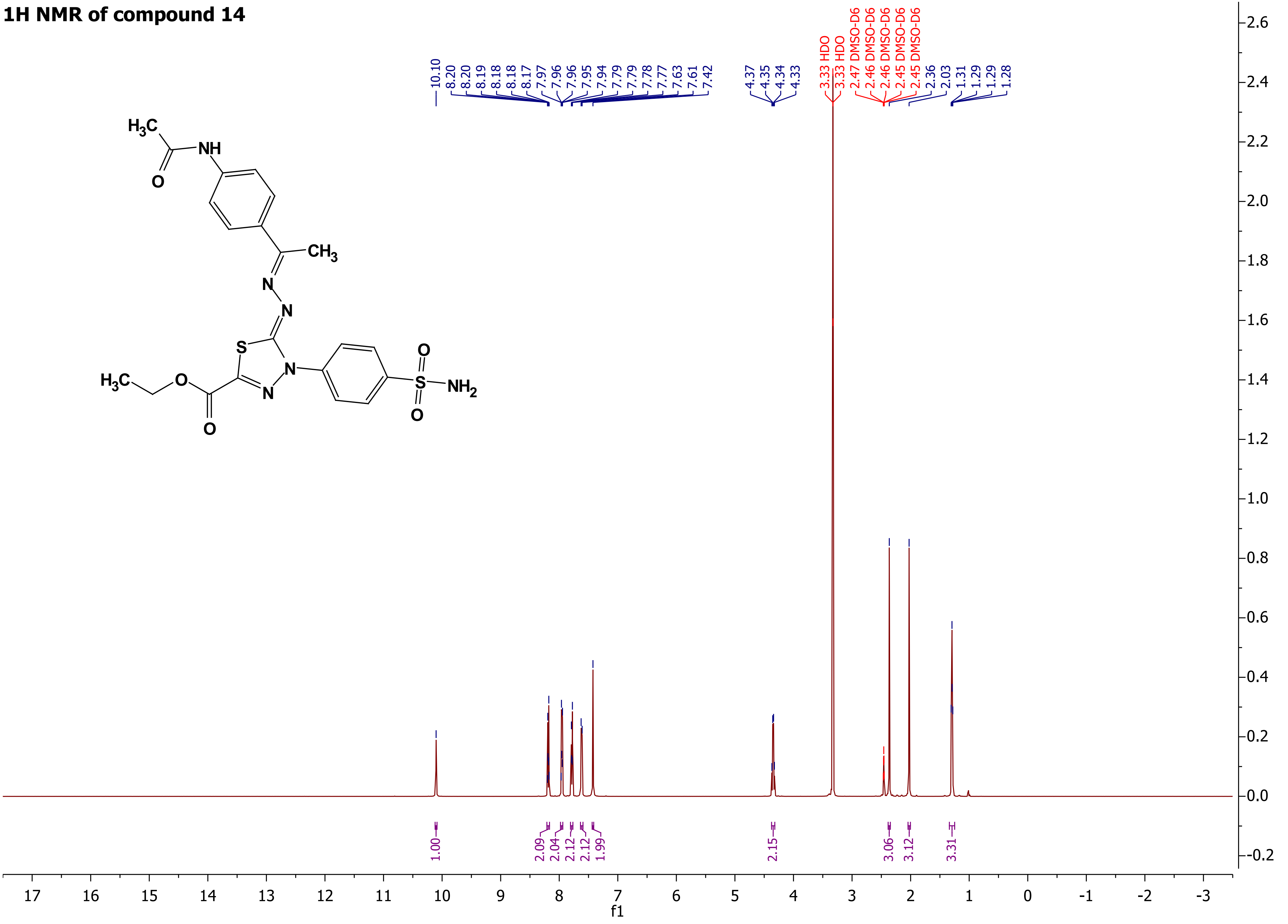

1H NMR of compound 14

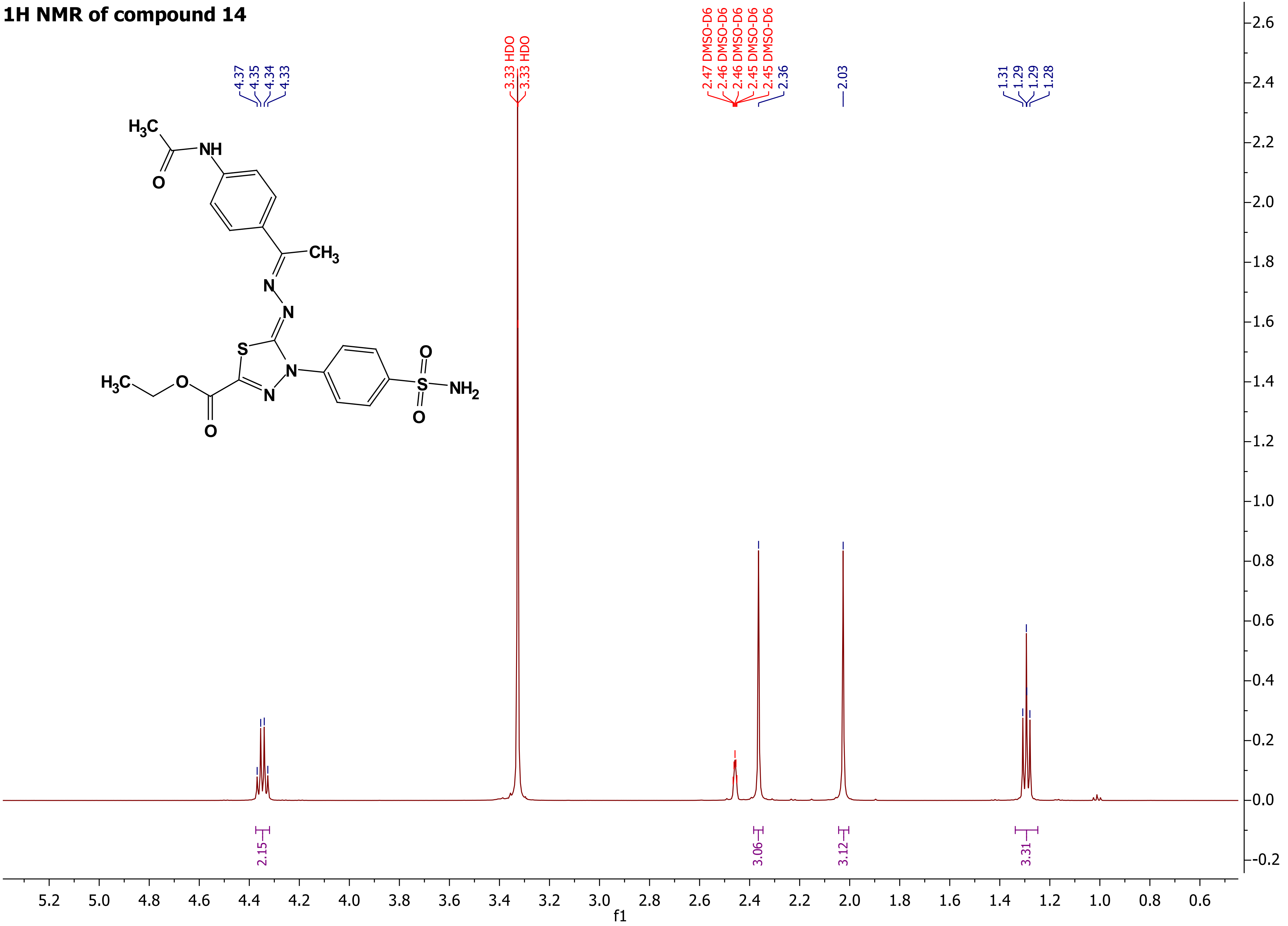

1H NMR of compound 14

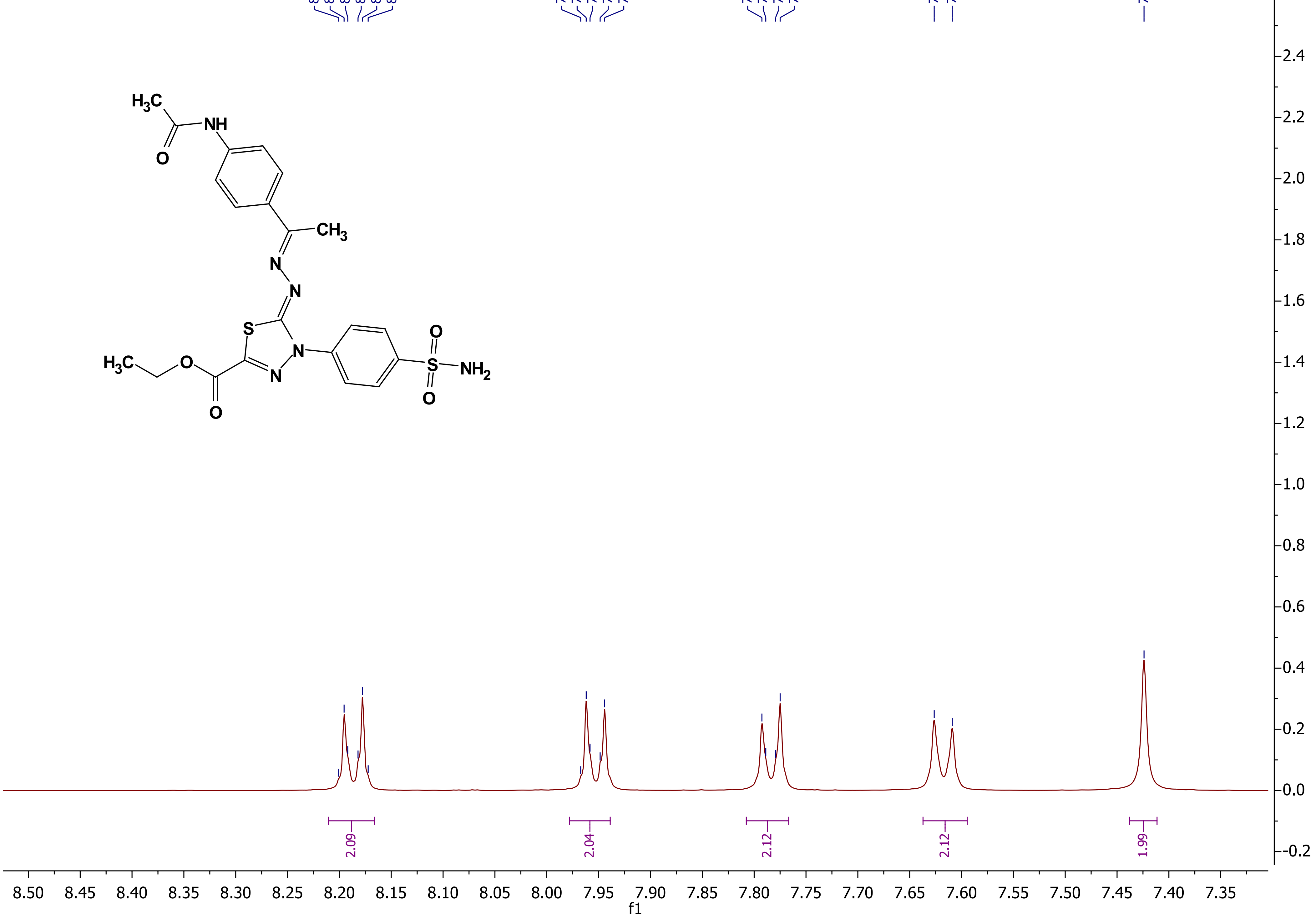

13C NMR of WA-11

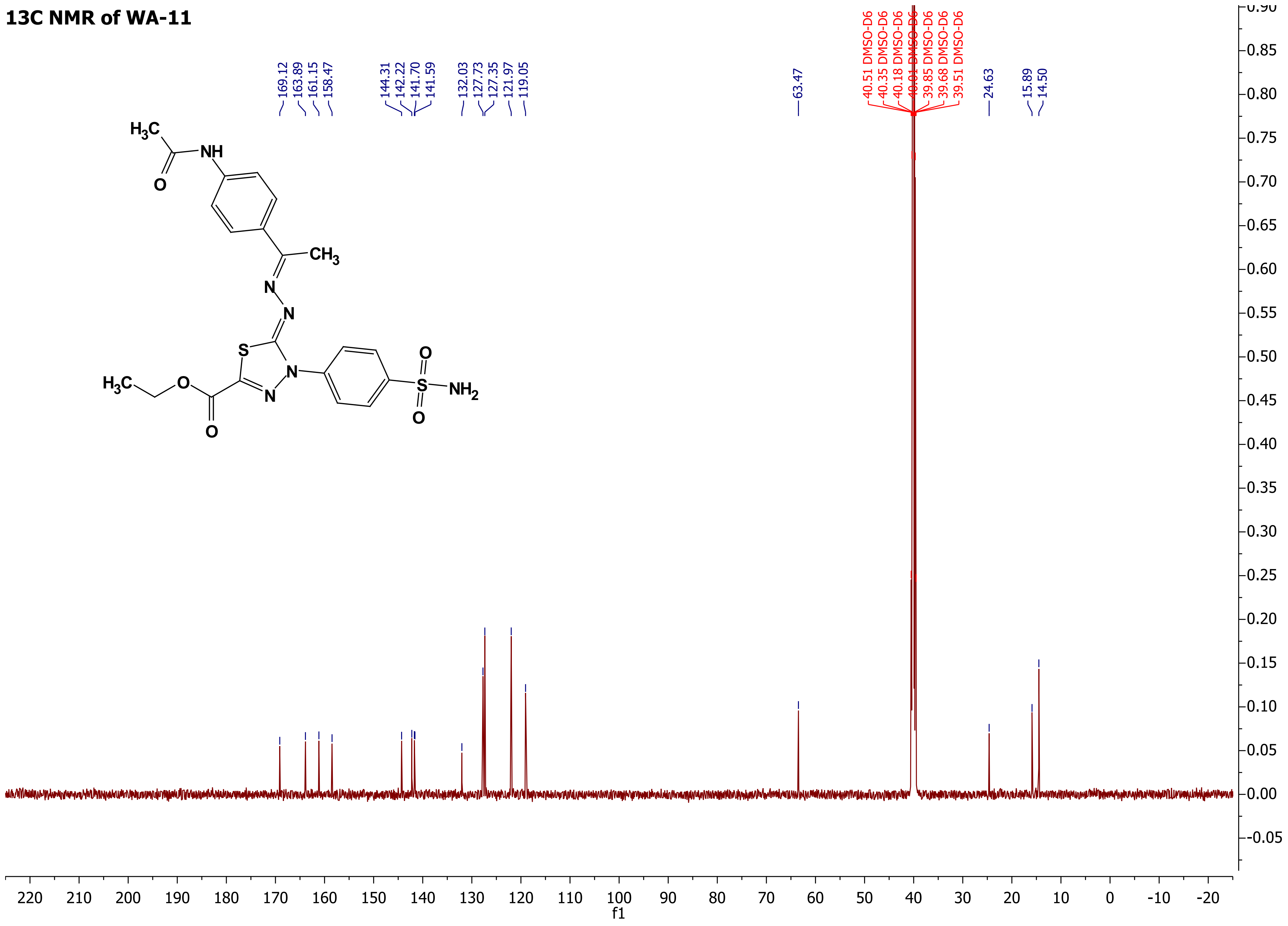

13C NMR of WA-11

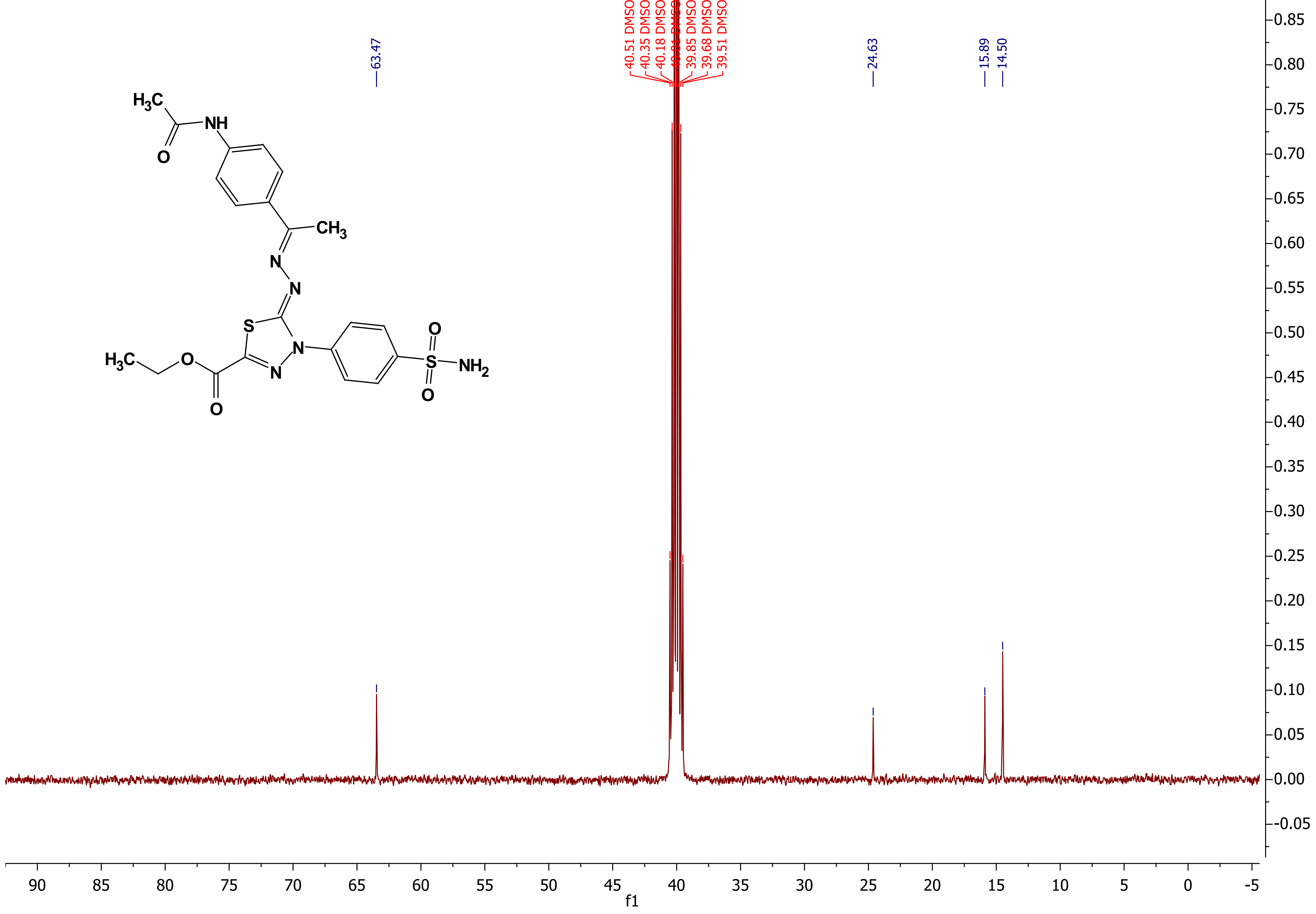

13C NMR of WA-11

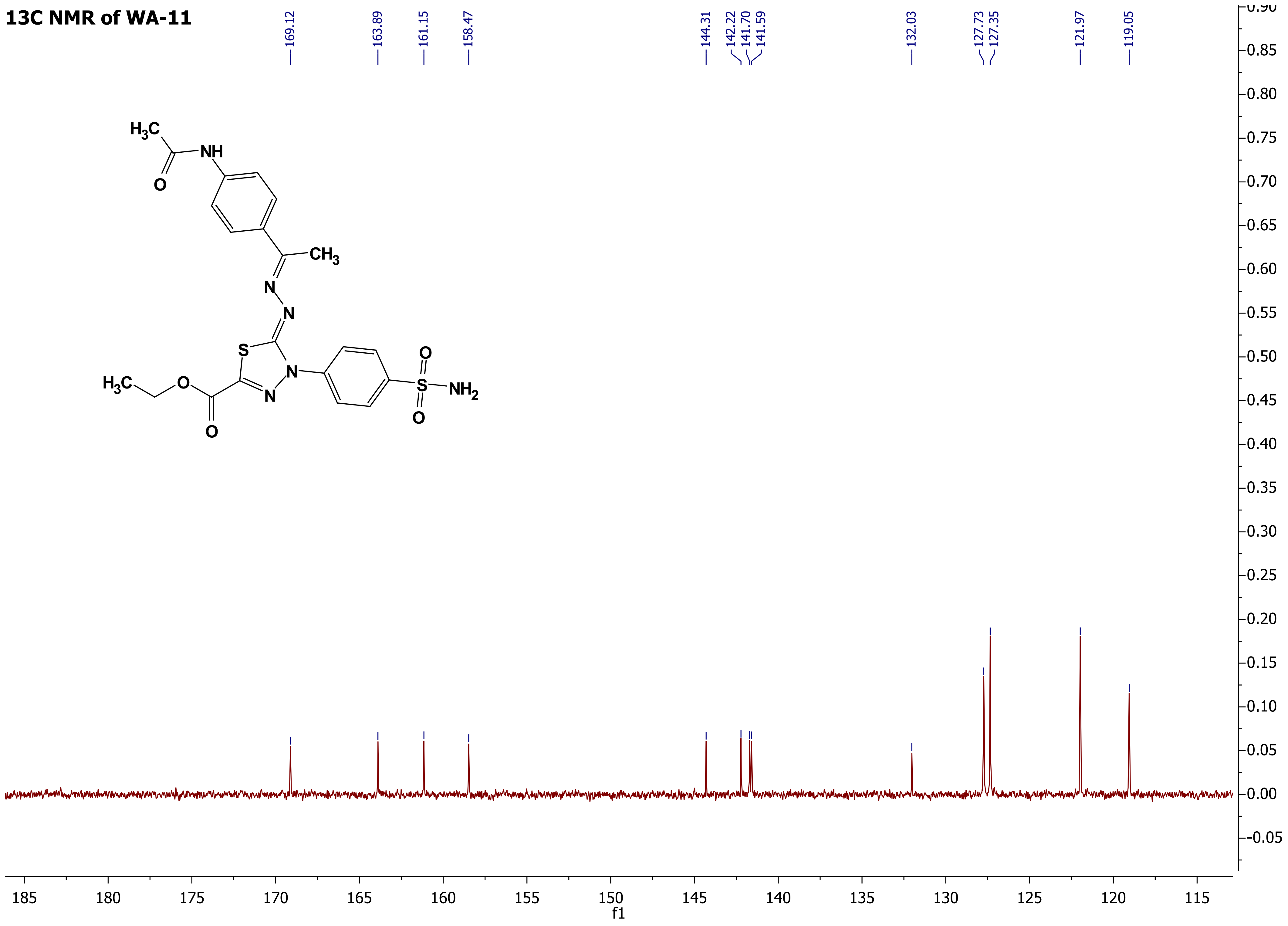

Mass spec. of compound 14

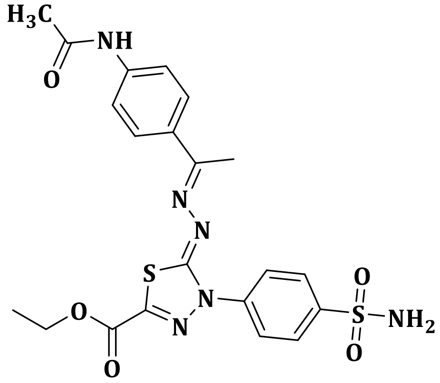

ibrahim-hassan-wa11 #300 RT: 5.04 AV: 1 SB: 2 3.82 , 3.53 NL: 2.00E3
T: {0,0} + c EI Full ms [40.00-1000.00]

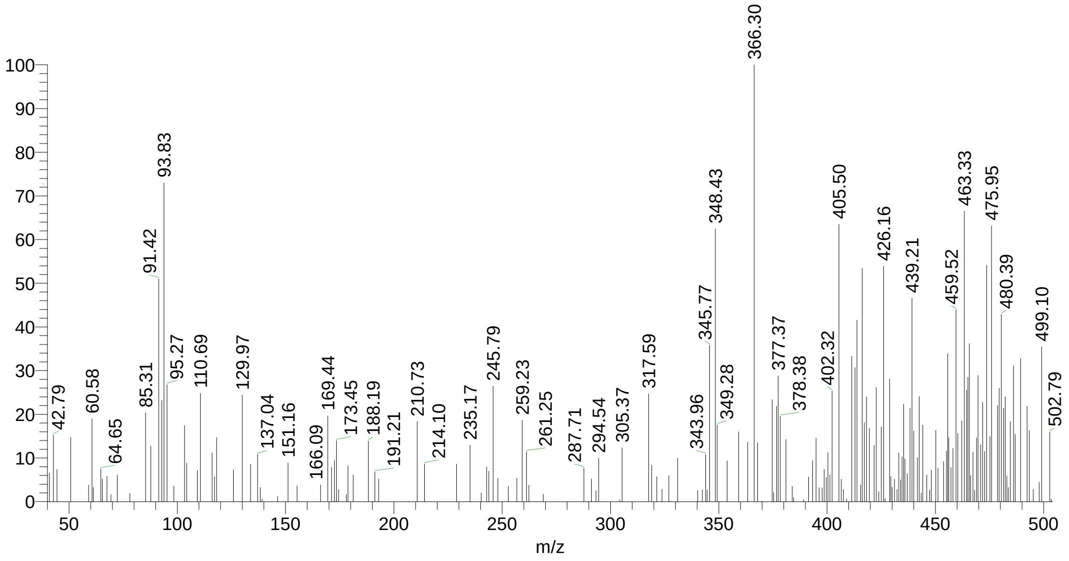

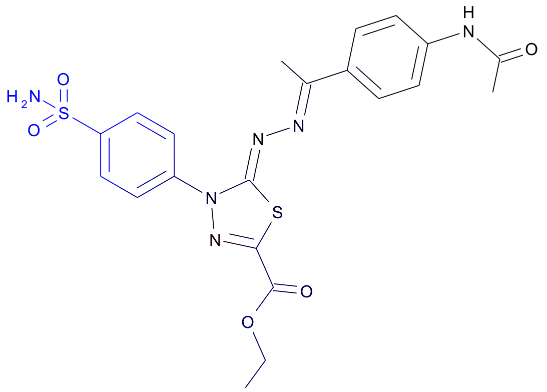

$C_{21}H_{22}N_6O_5S_2$
Molecular Weight: 502.56657
ALogP: 2.259
Rotatable Bonds: 8
Acceptors: 10
Donors: 2

Model Prediction

Prediction: Non-Mutagen
Probability: 0.236
Enrichment: 0.423
Bayesian Score: -13.3
Mahalanobis Distance: 12.9
Mahalanobis Distance p-value: 9.34e-006

Prediction: Positive if the Bayesian score is above the estimated best cutoff value from minimizing the false positive and false negative rate.
Probability: The esimated probability that the sample is in the positive category. This assumes that the Bayesian score follows a normal distribution and is different from the prediction using a cutoff.
Enrichment: An estimate of enrichment, that is, the increased likelihood (versus random) of this sample being in the category.
Bayesian Score: The standard Laplacian-modified Bayesian score.
Mahalanobis Distance: The Mahalanobis distance (MD) is the distance to the center of the training data. The larger the MD, the less trustworthy the prediction.
Mahalanobis Distance p-value: The p-value gives the fraction of training data with an MD greater than or equal to the one for the given sample, assuming normally distributed data. The smaller the p-value, the less trustworthy the prediciton. For highly non-normal X properties (e.g., fingerprints), the MD p-value is wildly inaccurate.

Structural Similar Compounds

Name	80734-02-7	15826-37-6	21794-01-4
Structure	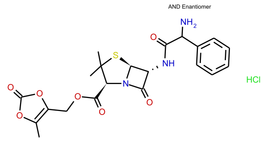	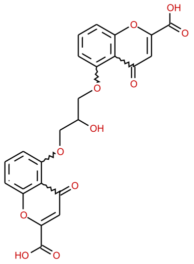	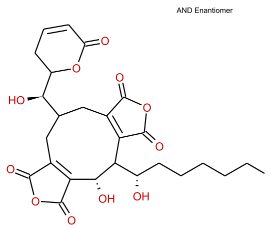
Actual Endpoint	Non-Mutagen	Non-Mutagen	Non-Mutagen
Predicted Endpoint	Non-Mutagen	Non-Mutagen	Non-Mutagen
Distance	0.612	0.614	0.616
Reference	Kazius et. al., J. Med. Chem. (2005) 48, 312-320	Kazius et. al., J. Med. Chem. (2005) 48, 312-320	Kazius et. al., J. Med. Chem. (2005) 48, 312-320

Model Applicability

Unknown features are fingerprint features in the query molecule, but not found or appearing too infrequently in the training set.

1. All properties and OPS components are within expected ranges.

Feature Contribution

Top features for positive contribution

Fingerprint	Bit/Smiles	Feature Structure	Score	Mutagen in training set
SCFP_12	-1325991669	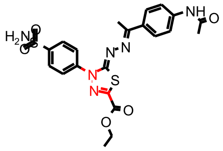 [*]N1[*][*]C(=N1)[*]	0.362	7 out of 8

SCFP_12	10	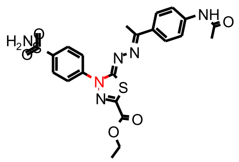 [*]N([*])[*]	0.306	1774 out of 2287
SCFP_12	-1380909229	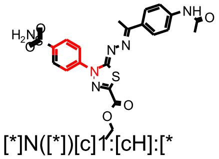 [*]N([*])[c]1:[cH]:[*]:[c]([*]):[cH]:[cH]:1	0.304	957 out of 1235

Top Features for negative contribution

Fingerprint	Bit/Smiles	Feature Structure	Score	Mutagen in training set
SCFP_12	1892918731	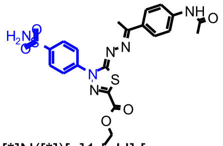 [*]N([*])[c]1:[cH]:[cH]:[c](:[cH]:[cH]:1)S(=O)(=O)N	-2.2	0 out of 14
SCFP_12	-1358544872	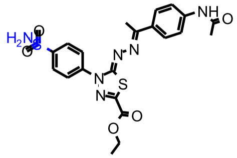 [*]S(=[*])(=[*])N	-1.57	1 out of 15
SCFP_12	-1463646519	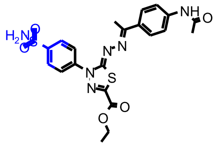 [*]:[cH]:[c](:[cH]:[*])S(=O)(=O)N	-1.29	1 out of 11

Erlotinib

TOPKAT_Ames_Mutagenicity

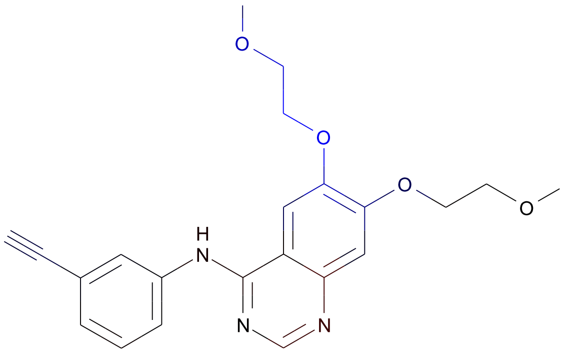

$C_{22}H_{23}N_3O_4$
Molecular Weight: 393.43572
ALogP: 4.309
Rotatable Bonds: 10
Acceptors: 7
Donors: 1

Structural Similar Compounds

Name	Carvedilol	99522-79-9	HYCANTHONE FUROATE
Structure	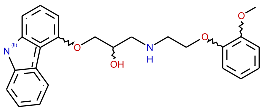	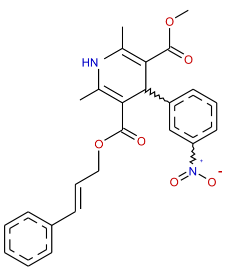	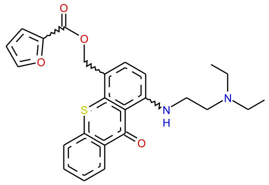
Actual Endpoint	Non-Mutagen	Non-Mutagen	Mutagen
Predicted Endpoint	Non-Mutagen	Non-Mutagen	Mutagen
Distance	0.594	0.598	0.606
Reference	Contrera, J.F., Matthews, E.J., Kruhlak, N.L., and Benz, R.D., Regulatory Toxicology and Pharmacology 2005, 313-323.	Kazius et. al., J. Med. Chem. (2005) 48, 312-320	EMIC

Model Prediction

Prediction: Non-Mutagen
Probability: 0.6
Enrichment: 1.07
Bayesian Score: -5.04
Mahalanobis Distance: 14.6
Mahalanobis Distance p-value: 6.74e-011

Prediction: Positive if the Bayesian score is above the estimated best cutoff value from minimizing the false positive and false negative rate.
Probability: The esimated probability that the sample is in the positive category. This assumes that the Bayesian score follows a normal distribution and is different from the prediction using a cutoff.
Enrichment: An estimate of enrichment, that is, the increased likelihood (versus random) of this sample being in the category.
Bayesian Score: The standard Laplacian-modified Bayesian score.
Mahalanobis Distance: The Mahalanobis distance (MD) is the distance to the center of the training data. The larger the MD, the less trustworthy the prediction.
Mahalanobis Distance p-value: The p-value gives the fraction of training data with an MD greater than or equal to the one for the given sample, assuming normally distributed data. The smaller the p-value, the less trustworthy the prediciton. For highly non-normal X properties (e.g., fingerprints), the MD p-value is wildly inaccurate.

Model Applicability

Unknown features are fingerprint features in the query molecule, but not found or appearing too infrequently in the training set.

1. All properties and OPS components are within expected ranges.

Feature Contribution

Top features for positive contribution

Fingerprint	Bit/Smiles	Feature Structure	Score	Mutagen in training set
SCFP_12	112346096	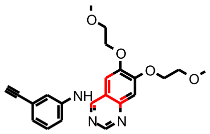 [*][c](:[*]):[c](:[cH]:[*]):[c](:[*]):[*]	0.36	1035 out of 1263

SCFP_12	10	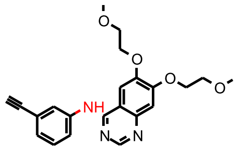 [*]N([*])[*]	0.306	1774 out of 2287
SCFP_12	-1380909229	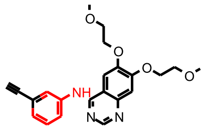 [*]N([*])[c]1:[cH]:[*]:[c]([*]):[cH]:[cH] :1	0.304	957 out of 1235
**Top Features for negative contribution**				
**Fingerprint**	**Bit/Smiles**	**Feature Structure**	**Score**	**Mutagen in training set**
SCFP_12	-1099149596	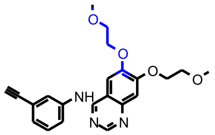 [*]OCCO[c](:[*]):[*]	-1.12	1 out of 9
SCFP_12	-677502852	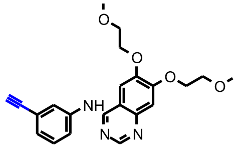 [*]C#C	-0.863	4 out of 19
SCFP_12	-417738003	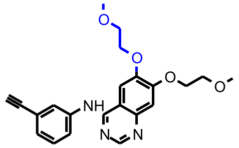 [*]OCCOC	-0.782	12 out of 48

acetazolamide.cdx — TOPKAT_Ames_Mutagenicity

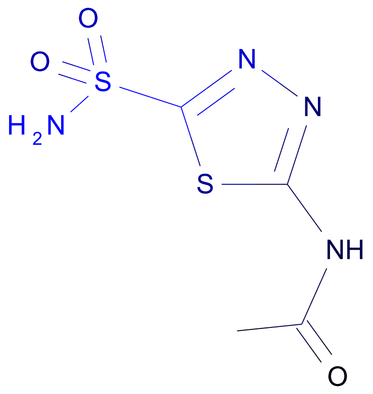

$C_4H_6N_4O_3S_2$
Molecular Weight: 222.24544
ALogP: -1.329
Rotatable Bonds: 2
Acceptors: 5
Donors: 2

Model Prediction

Prediction: Non-Mutagen
Probability: 0.533
Enrichment: 0.954
Bayesian Score: -6.75
Mahalanobis Distance: 10.4
Mahalanobis Distance p-value: 0.157

Prediction: Positive if the Bayesian score is above the estimated best cutoff value from minimizing the false positive and false negative rate.
Probability: The esimated probability that the sample is in the positive category. This assumes that the Bayesian score follows a normal distribution and is different from the prediction using a cutoff.
Enrichment: An estimate of enrichment, that is, the increased likelihood (versus random) of this sample being in the category.
Bayesian Score: The standard Laplacian-modified Bayesian score.
Mahalanobis Distance: The Mahalanobis distance (MD) is the distance to the center of the training data. The larger the MD, the less trustworthy the prediction.
Mahalanobis Distance p-value: The p-value gives the fraction of training data with an MD greater than or equal to the one for the given sample, assuming normally distributed data. The smaller the p-value, the less trustworthy the prediciton. For highly non-normal X properties (e.g., fingerprints), the MD p-value is wildly inaccurate.

Structural Similar Compounds

Name	ACETAZOLAMIDE	60391-92-6	2578-75-8
Structure	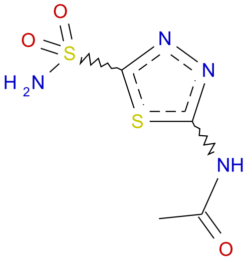	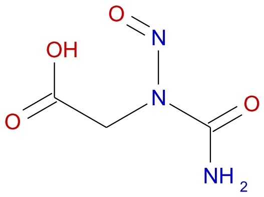	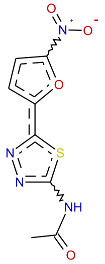
Actual Endpoint	Non-Mutagen	Non-Mutagen	Mutagen
Predicted Endpoint	Non-Mutagen	Mutagen	Mutagen
Distance	0.000	0.571	0.575
Reference	PDR 1994	Helma, C., Cramer, T., Kramer, S., and De Raedt, L., J. Chem. Inf. Comput. Sci., 2004, pp. 1402-1411	Helma, C., Cramer, T., Kramer, S., and De Raedt, L., J. Chem. Inf. Comput. Sci., 2004, pp. 1402-1411

Model Applicability

Unknown features are fingerprint features in the query molecule, but not found or appearing too infrequently in the training set.

1. All properties and OPS components are within expected ranges.

Feature Contribution

Top features for positive contribution				
Fingerprint	Bit/Smiles	Feature Structure	Score	Mutagen in training set
SCFP_12	-1607191420	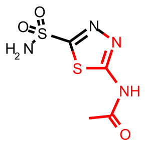 CC(=O)N[c]1:n:[*]:[*]:s:1	0.442	13 out of 14

SCFP_12	-100019659	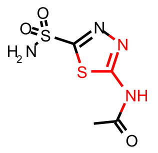 [*]N[c]1:n:[*]:[*]:s:1	0.413	29 out of 33
SCFP_12	1310748454	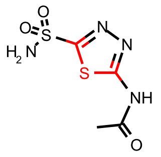 [*][c]1:[*]:[*]:[c]([*]):s:1	0.312	126 out of 161

Top Features for negative contribution

**Fingerprint**	**Bit/Smiles**	**Feature Structure**	**Score**	**Mutagen in training set**
SCFP_12	-1358544872	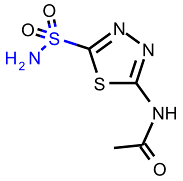 [*]S(=[*])(=[*])N	-1.57	1 out of 15
SCFP_12	182991870	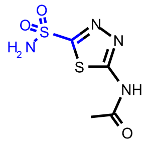 [*]:[c](:[*])S(=O)(=O)N	-1.15	8 out of 48
SCFP_12	1184199839	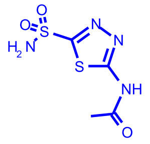 CC(=O)N[c]1:n:n:[c](:s:1)S(=O)(=O)N	-0.452	0 out of 1

Comp. 14

TOPKAT_Developmental_Toxicity_Potential

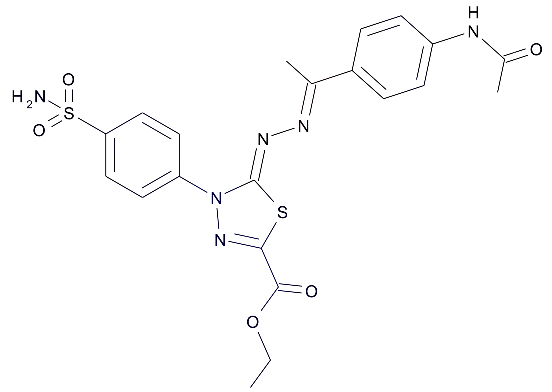

$C_{21}H_{22}N_6O_5S_2$

Molecular Weight: 502.56657

ALogP: 2.259

Rotatable Bonds: 8

Acceptors: 10

Donors: 2

Model Prediction

Prediction: Non-Toxic

Probability: 0.453

Enrichment: 0.861

Bayesian Score: -2.68

Mahalanobis Distance: 11.7

Mahalanobis Distance p-value: 8.21e-005

Prediction: Positive if the Bayesian score is above the estimated best cutoff value from minimizing the false positive and false negative rate.
Probability: The esimated probability that the sample is in the positive category. This assumes that the Bayesian score follows a normal distribution and is different from the prediction using a cutoff.
Enrichment: An estimate of enrichment, that is, the increased likelihood (versus random) of this sample being in the category.
Bayesian Score: The standard Laplacian-modified Bayesian score.
Mahalanobis Distance: The Mahalanobis distance (MD) is the distance to the center of the training data. The larger the MD, the less trustworthy the prediction.
Mahalanobis Distance p-value: The p-value gives the fraction of training data with an MD greater than or equal to the one for the given sample, assuming normally distributed data. The smaller the p-value, the less trustworthy the prediciton. For highly non-normal X properties (e.g., fingerprints), the MD p-value is wildly inaccurate.

Structural Similar Compounds

Name	Cyclic AMP Bucladesine	Bacampicillin .HCl (Free base form)	Lenampicillin .HCl (Free base form)
Structure	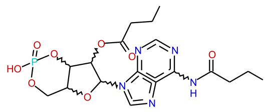	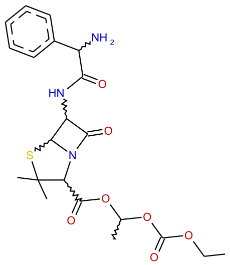	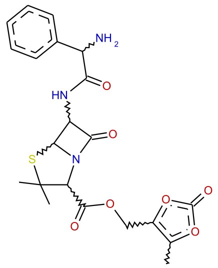
Actual Endpoint	Non-Toxic	Toxic	Non-Toxic
Predicted Endpoint	Non-Toxic	Non-Toxic	Non-Toxic
Distance	0.602	0.618	0.622
Reference	Oyo Yakuri 27(3):585-597; 1984	Chemotherapy 27:30-35; 1979	Chemotherapy 32:130-145; 1984

Model Applicability

Unknown features are fingerprint features in the query molecule, but not found or appearing too infrequently in the training set.

1. OPS PC19 out of range. Value: -3.0221. Training min, max, SD, explained variance: -2.7817, 3.2747, 1.037, 0.0157.

Feature Contribution

Top features for positive contribution				
**Fingerprint**	**Bit/Smiles**	**Feature Structure**	**Score**	**Toxic in training set**
SCFP_6	-331724199	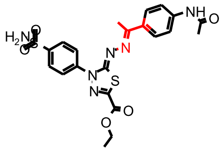 [*]\N=C(/C)\[c](:[*]):[*]	0.271	1 out of 1

SCFP_6	1626825020	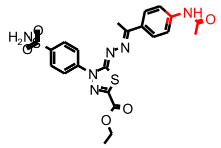 [*]:[c](:[*])NC(=O)C	0.271	1 out of 1
SCFP_6	-1247518081	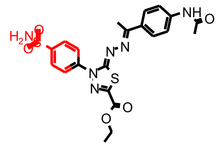 NS(=O)(=O)[c]1:[cH]:[cH]:[*]:[cH]:[cH]:1	0.271	1 out of 1

Top Features for negative contribution

Fingerprint	Bit/Smiles	Feature Structure	Score	Toxic in training set
SCFP_6	-1463646519	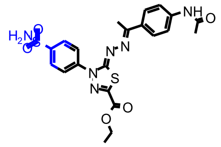 [*]:[cH]:[c](:[cH]:[*])S(=O)(=O)N	-0.718	0 out of 2
SCFP_6	-1380909229	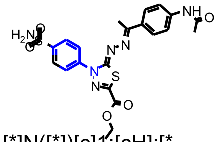 [*]N([*])[c]1:[cH]:[*]:[c]([*]):[cH]:[cH] :1	-0.449	6 out of 19
SCFP_6	1586247563	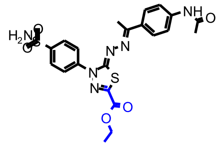 [*]C(=[*])C(=O)OCC	-0.438	1 out of 4

Erlotinib

TOPKAT_Developmental_Toxicity_Potential

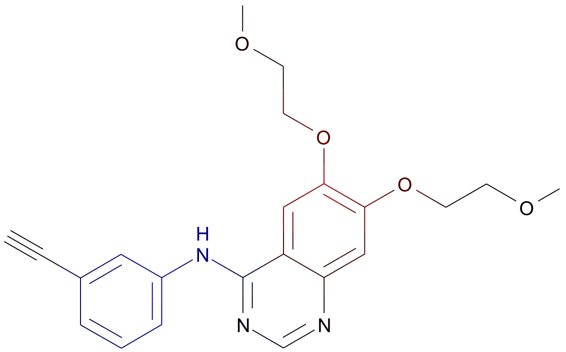

$C_{22}H_{23}N_3O_4$
Molecular Weight: 393.43572
ALogP: 4.309
Rotatable Bonds: 10
Acceptors: 7
Donors: 1

Model Prediction

Prediction: Non-Toxic
Probability: 0.504
Enrichment: 0.959
Bayesian Score: -1.17
Mahalanobis Distance: 8.76
Mahalanobis Distance p-value: 0.258

Prediction: Positive if the Bayesian score is above the estimated best cutoff value from minimizing the false positive and false negative rate.
Probability: The esimated probability that the sample is in the positive category. This assumes that the Bayesian score follows a normal distribution and is different from the prediction using a cutoff.
Enrichment: An estimate of enrichment, that is, the increased likelihood (versus random) of this sample being in the category.
Bayesian Score: The standard Laplacian-modified Bayesian score.
Mahalanobis Distance: The Mahalanobis distance (MD) is the distance to the center of the training data. The larger the MD, the less trustworthy the prediction.
Mahalanobis Distance p-value: The p-value gives the fraction of training data with an MD greater than or equal to the one for the given sample, assuming normally distributed data. The smaller the p-value, the less trustworthy the prediciton. For highly non-normal X properties (e.g., fingerprints), the MD p-value is wildly inaccurate.

Structural Similar Compounds

Name	Nicardipine	Suxibuzone	Etofenamate
Structure	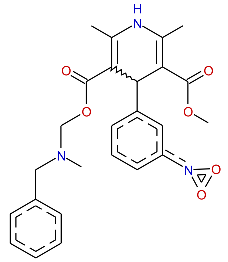	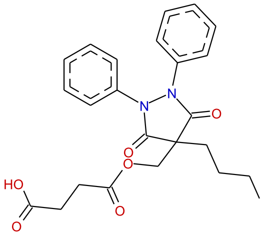	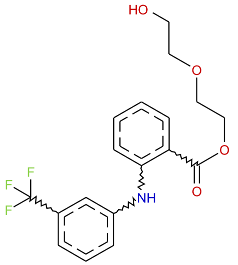
Actual Endpoint	Non-Toxic	Toxic	Non-Toxic
Predicted Endpoint	Non-Toxic	Toxic	Non-Toxic
Distance	0.608	0.620	0.635
Reference	Kiso to Rinsho 13:1149-1159; 1979	Oyo Yakuri 20:377-386; 1980	Iyakuhin Kenkyu 13(4):896-909; 1982

Model Applicability

Unknown features are fingerprint features in the query molecule, but not found or appearing too infrequently in the training set.

1. All properties and OPS components are within expected ranges.

Feature Contribution

Top features for positive contribution

Fingerprint	Bit/Smiles	Feature Structure	Score	Toxic in training set
SCFP_6	123285475	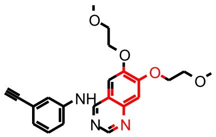 [*]O[c]1:[cH]:[c](:n:[*]):[c](:[*]):[*]:[c]:1[*]	0.478	4 out of 4

SCFP_6	446954673	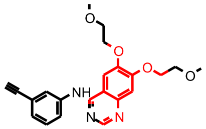 [*]CO[c]1:[cH]:[c]2:n:[cH]:[*]:[c]([*]):[c]:2:[cH]:[c]:1O[*]	0.381	2 out of 2
SCFP_6	-1814968949	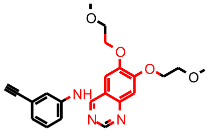 [*]CO[c]1:[cH]:[c]2:[c](N[*]):n:[*]:n:[c]:2:[cH]:[c]:1O[*]	0.381	2 out of 2

Top Features for negative contribution

Fingerprint	Bit/Smiles	Feature Structure	Score	Toxic in training set
SCFP_6	2142015375	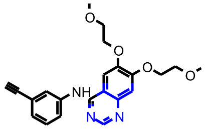 [*]:[cH]:[c]1:n:[cH]:n:[*]:[c]:1:[*]	-0.718	0 out of 2
SCFP_6	-2020651081	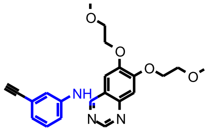 [*][c]1:[cH]:[cH]:[cH]:[c](N[c](:[*]):[*]):[cH]:1	-0.718	0 out of 2
SCFP_6	-300914917	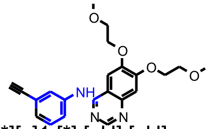 [*][c]1:[*]:[cH]:[cH]:[c](N[c](:[*]):[*]):[cH]:1	-0.718	0 out of 2

acetazolamide.cdx

TOPKAT_Developmental_Toxicity_Potential

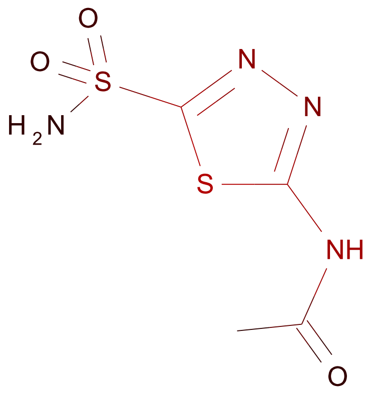

$C_4H_6N_4O_3S_2$
Molecular Weight: 222.24544
ALogP: -1.329
Rotatable Bonds: 2
Acceptors: 5
Donors: 2

Model Prediction

Prediction: Toxic
Probability: 0.684
Enrichment: 1.3
Bayesian Score: 3.49
Mahalanobis Distance: 8.25
Mahalanobis Distance p-value: 0.483

Prediction: Positive if the Bayesian score is above the estimated best cutoff value from minimizing the false positive and false negative rate.
Probability: The esimated probability that the sample is in the positive category. This assumes that the Bayesian score follows a normal distribution and is different from the prediction using a cutoff.
Enrichment: An estimate of enrichment, that is, the increased likelihood (versus random) of this sample being in the category.
Bayesian Score: The standard Laplacian-modified Bayesian score.
Mahalanobis Distance: The Mahalanobis distance (MD) is the distance to the center of the training data. The larger the MD, the less trustworthy the prediction.
Mahalanobis Distance p-value: The p-value gives the fraction of training data with an MD greater than or equal to the one for the given sample, assuming normally distributed data. The smaller the p-value, the less trustworthy the prediciton. For highly non-normal X properties (e.g., fingerprints), the MD p-value is wildly inaccurate.

Structural Similar Compounds

Name	Acetazolamide	Chlorothiazide	6-Aminonicotinamide
Structure	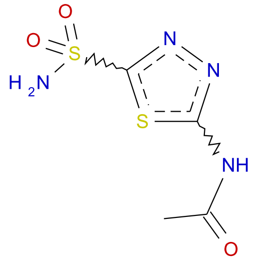	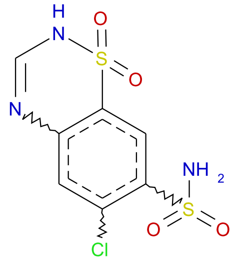	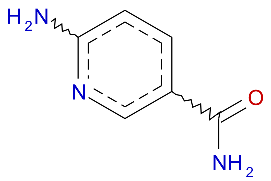
Actual Endpoint	Toxic	Non-Toxic	Toxic
Predicted Endpoint	Toxic	Non-Toxic	Toxic
Distance	0.000	0.713	0.767
Reference	Br J Exp Pathol 53(1):5-21; 1972	John Hopkins Med J 130:95-104; 1972	Teratology 1(1):103-8; 1968

Model Applicability

Unknown features are fingerprint features in the query molecule, but not found or appearing too infrequently in the training set.

1. All properties and OPS components are within expected ranges.

Feature Contribution

Top features for positive contribution

Fingerprint	Bit/Smiles	Feature Structure	Score	Toxic in training set
SCFP_6	1626825020	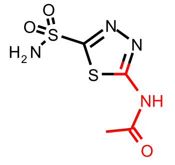 [*]:[c](:[*])NC(=O)C	0.271	1 out of 1

SCFP_6	-1649012287	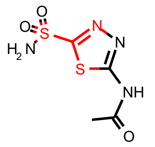 [*]S(=[*])(=[*])[c]1:n:[*]:[*]:s:1	0.271	1 out of 1
SCFP_6	-100019659	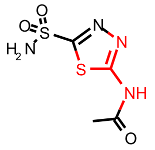 [*]N[c]1:n:[*]:[*]:s:1	0.271	1 out of 1
**Top Features for negative contribution**				
**Fingerprint**	**Bit/Smiles**	**Feature Structure**	**Score**	**Toxic in training set**
SCFP_6	149212520	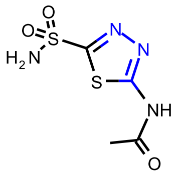 [*][c]1:[*]:[*]:n:n:1	-0.448	5 out of 16
SCFP_6	8	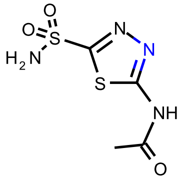 [*]:n:[*]	-0.278	24 out of 61
SCFP_6	-1358544872	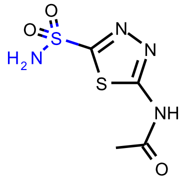 [*]S(=[*])(=[*])N	-0.252	1 out of 3

Comp. 14

TOPKAT_Mouse_Female_FDA_None_vs_Carcinogen

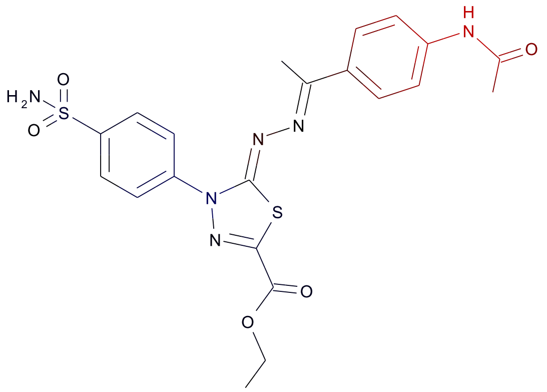

$C_{21}H_{22}N_6O_5S_2$

Molecular Weight: 502.56657

ALogP: 2.259

Rotatable Bonds: 8

Acceptors: 10

Donors: 2

Model Prediction

Prediction: Non-Carcinogen

Probability: 0.23

Enrichment: 0.718

Bayesian Score: -2.05

Mahalanobis Distance: 13.1

Mahalanobis Distance p-value: 0.00034

Prediction: Positive if the Bayesian score is above the estimated best cutoff value from minimizing the false positive and false negative rate.
Probability: The esimated probability that the sample is in the positive category. This assumes that the Bayesian score follows a normal distribution and is different from the prediction using a cutoff.
Enrichment: An estimate of enrichment, that is, the increased likelihood (versus random) of this sample being in the category.
Bayesian Score: The standard Laplacian-modified Bayesian score.
Mahalanobis Distance: The Mahalanobis distance (MD) is the distance to the center of the training data. The larger the MD, the less trustworthy the prediction.
Mahalanobis Distance p-value: The p-value gives the fraction of training data with an MD greater than or equal to the one for the given sample, assuming normally distributed data. The smaller the p-value, the less trustworthy the prediciton. For highly non-normal X properties (e.g., fingerprints), the MD p-value is wildly inaccurate.

Structural Similar Compounds

Name	Bacampicillin	Sulfasalazine	Nimodipine
Structure	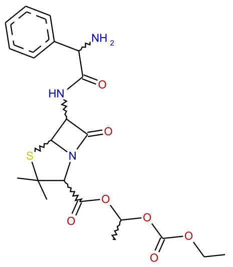	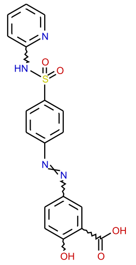	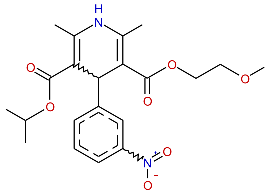
Actual Endpoint	Non-Carcinogen	Carcinogen	Non-Carcinogen
Predicted Endpoint	Non-Carcinogen	Carcinogen	Non-Carcinogen
Distance	0.629	0.778	0.788
Reference	US FDA (Centre for Drug Eval.& Res./Off. Testing & Res.) Sept. 1997	US FDA (Centre for Drug Eval.& Res./Off. Testing & Res.) Sept. 1997	US FDA (Centre for Drug Eval.& Res./Off. Testing & Res.) Sept. 1997

Model Applicability

Unknown features are fingerprint features in the query molecule, but not found or appearing too infrequently in the training set.

1. OPS PC4 out of range. Value: 5.6869. Training min, max, SD, explained variance: -4.7116, 4.7287, 2.103, 0.0450.
2. Unknown ECFP_2 feature: -934225701: [*]C(=[*])C1=N[*][*]S1
3. Unknown ECFP_2 feature: -1110911409: [*]=C1[*][*]=NN1[c](:[*]):[*]
4. Unknown ECFP_2 feature: 189949281: [*]\N=C\1/S[*]=[*]N1[*]
5. Unknown ECFP_2 feature: -819426257: [*]C(=NN=[*])[*]
6. Unknown ECFP_2 feature: 562081661: [*]C(=NN=[*])[*]
7. Unknown ECFP_2 feature: 128986386: [*]\N=C(/C)\[c](:[*]):[*]

Feature Contribution

Top features for positive contribution				
**Fingerprint**	**Bit/Smiles**	**Feature Structure**	**Score**	**Carcinogen in training set**

ECFP_6	-1087070950	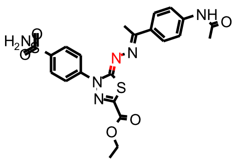 [*]N=[*]	0.724	10 out of 14
ECFP_6	-847011520	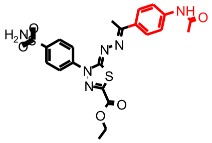 [*][c]1:[cH]:[cH]:[c](NC(=O)C):[cH]:[cH]:1	0.617	2 out of 2
ECFP_6	1776488	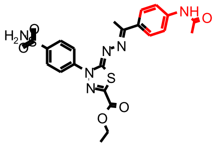 CC(=O)N[c]1:[cH]:[cH]:[*]:[cH]:[cH]:1	0.617	2 out of 2
**Top Features for negative contribution**				
**Fingerprint**	**Bit/Smiles**	**Feature Structure**	**Score**	**Carcinogen in training set**
ECFP_6	-175021654	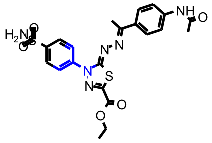 [*]N([*])[c](:[cH]:[*]):[cH]:[*]	-0.805	0 out of 4
ECFP_6	912478223	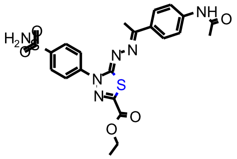 [*]S[*]	-0.638	1 out of 9

ECFP_6	-2137232509	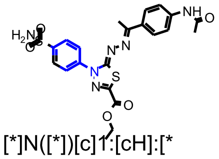 [*]N([*])[c]1:[cH]:[*]:[c]([*]):[cH]:[cH]:1	-0.482	0 out of 2

Erlotinib

TOPKAT_Mouse_Female_FDA_None_vs_Carcinogen

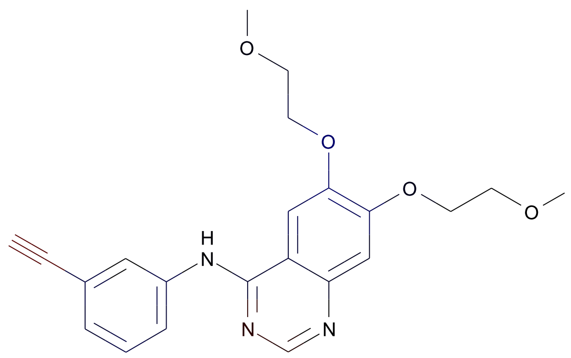

$C_{22}H_{23}N_3O_4$
Molecular Weight: 393.43572
ALogP: 4.309
Rotatable Bonds: 10
Acceptors: 7
Donors: 1

Model Prediction

Prediction: Non-Carcinogen
Probability: 0.235
Enrichment: 0.733
Bayesian Score: -1.68
Mahalanobis Distance: 15.3
Mahalanobis Distance p-value: 7.99e-008

Prediction: Positive if the Bayesian score is above the estimated best cutoff value from minimizing the false positive and false negative rate.
Probability: The esimated probability that the sample is in the positive category. This assumes that the Bayesian score follows a normal distribution and is different from the prediction using a cutoff.
Enrichment: An estimate of enrichment, that is, the increased likelihood (versus random) of this sample being in the category.
Bayesian Score: The standard Laplacian-modified Bayesian score.
Mahalanobis Distance: The Mahalanobis distance (MD) is the distance to the center of the training data. The larger the MD, the less trustworthy the prediction.
Mahalanobis Distance p-value: The p-value gives the fraction of training data with an MD greater than or equal to the one for the given sample, assuming normally distributed data. The smaller the p-value, the less trustworthy the prediciton. For highly non-normal X properties (e.g., fingerprints), the MD p-value is wildly inaccurate.

Structural Similar Compounds

Name	Mycophenolate	Nicardipine	Nimodipine
Structure	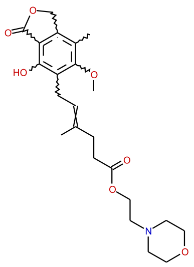	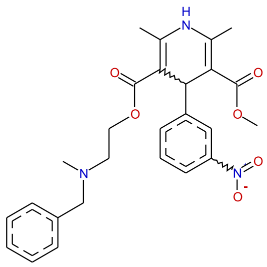	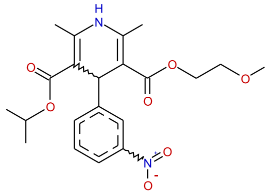
Actual Endpoint	Non-Carcinogen	Non-Carcinogen	Non-Carcinogen
Predicted Endpoint	Non-Carcinogen	Non-Carcinogen	Non-Carcinogen
Distance	0.632	0.664	0.675
Reference	US FDA (Centre for Drug Eval.& Res./Off. Testing & Res.) Sept. 1997	US FDA (Centre for Drug Eval.& Res./Off. Testing & Res.) Sept. 1997	US FDA (Centre for Drug Eval.& Res./Off. Testing & Res.) Sept. 1997

Model Applicability

Unknown features are fingerprint features in the query molecule, but not found or appearing too infrequently in the training set.

1. All properties and OPS components are within expected ranges.
2. Unknown ECFP_2 feature: -182178874: [*]#C[c](:c:[*]):c:[*]
3. Unknown ECFP_2 feature: 1139738044: [*]:[c](:[*])C#C

Feature Contribution

Top features for positive contribution

Fingerprint	Bit/Smiles	Feature Structure	Score	Carcinogen in training set
ECFP_6	-1939823063	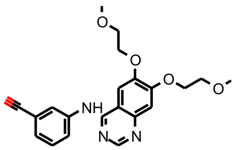 [*]#C	0.866	8 out of 9

ECFP_6	-1545539812	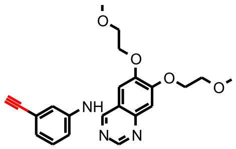<br>[*]C#C	0.866	8 out of 9
ECFP_6	-1114776580	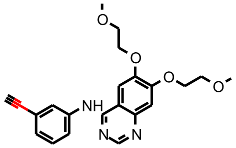<br>[*]C#[*]	0.755	11 out of 15
**Top Features for negative contribution**				
**Fingerprint**	**Bit/Smiles**	**Feature Structure**	**Score**	**Carcinogen in training set**
ECFP_6	2007300961	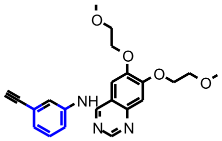<br>[*][c]1:[*]:[c]([*]):<br>[cH]:[cH]:[cH]:1	-0.652	5 out of 34
ECFP_6	-2063202154	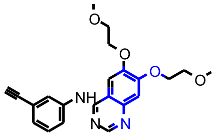<br>[*]O[c]1:[cH]:[c](:n:<br>[*]):[c](:[*]):[*]:[<br>c]:1[*]	-0.482	0 out of 2
ECFP_6	-2063600634	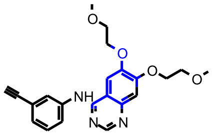<br>[*]O[c]1:[cH]:[c](:[c<br>]([*]):[*]):[c](:[*]<br>):[*]:[c]:1[*]	-0.482	0 out of 2

acetazolamide.cdx

TOPKAT_Mouse_Female_FDA_None_vs_Carcinogen

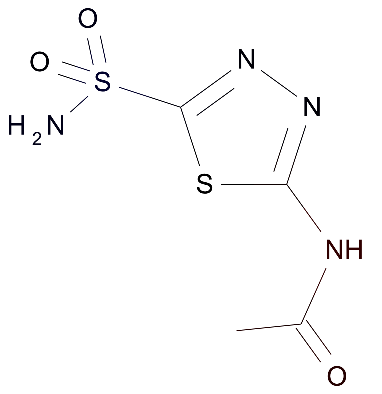

$C_4H_6N_4O_3S_2$
Molecular Weight: 222.24544
ALogP: -1.329
Rotatable Bonds: 2
Acceptors: 5
Donors: 2

Model Prediction

Prediction: Carcinogen
Probability: 0.251
Enrichment: 0.785
Bayesian Score: -0.612
Mahalanobis Distance: 12.9
Mahalanobis Distance p-value: 0.000784

Prediction: Positive if the Bayesian score is above the estimated best cutoff value from minimizing the false positive and false negative rate.
Probability: The esimated probability that the sample is in the positive category. This assumes that the Bayesian score follows a normal distribution and is different from the prediction using a cutoff.
Enrichment: An estimate of enrichment, that is, the increased likelihood (versus random) of this sample being in the category.
Bayesian Score: The standard Laplacian-modified Bayesian score.
Mahalanobis Distance: The Mahalanobis distance (MD) is the distance to the center of the training data. The larger the MD, the less trustworthy the prediction.
Mahalanobis Distance p-value: The p-value gives the fraction of training data with an MD greater than or equal to the one for the given sample, assuming normally distributed data. The smaller the p-value, the less trustworthy the prediciton. For highly non-normal X properties (e.g., fingerprints), the MD p-value is wildly inaccurate.

Structural Similar Compounds

Name	Nitrofurazone	Aminonitrothiazole	Azaserine
Structure	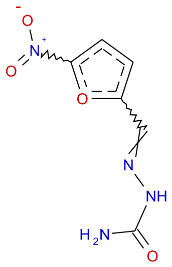	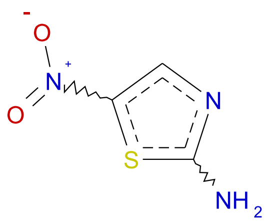	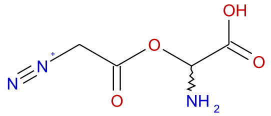
Actual Endpoint	Carcinogen	Non-Carcinogen	Non-Carcinogen
Predicted Endpoint	Carcinogen	Carcinogen	Non-Carcinogen
Distance	0.681	0.702	0.726
Reference	US FDA (Centre for Drug Eval.& Res./Off. Testing & Res.) Sept. 1997	US FDA (Centre for Drug Eval.& Res./Off. Testing & Res.) Sept. 1997	US FDA (Centre for Drug Eval.& Res./Off. Testing & Res.) Sept. 1997

Model Applicability

Unknown features are fingerprint features in the query molecule, but not found or appearing too infrequently in the training set.

1. All properties and OPS components are within expected ranges.
2. Unknown ECFP_2 feature: -1596132236: [*]N[c]1:n:[*]:[*]:s:1
3. Unknown ECFP_2 feature: 1221843808: [*]S(=[*])(=[*])[c]1:n:[*]:[*]:s:1

Feature Contribution

Top features for positive contribution				
Fingerprint	Bit/Smiles	Feature Structure	Score	Carcinogen in training set
ECFP_6	-1923054811	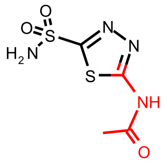 [*]:[c](:[*])NC(=O)C	0.442	2 out of 3

ECFP_6	-474544785	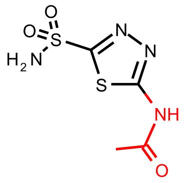 [*]NC(=O)C	0.442	2 out of 3
ECFP_6	914325265	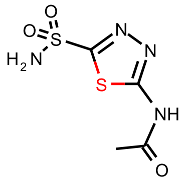 [*]:s:[*]	0.127	4 out of 11
**Top Features for negative contribution**				
**Fingerprint**	**Bit/Smiles**	**Feature Structure**	**Score**	**Carcinogen in training set**
ECFP_6	2102150379	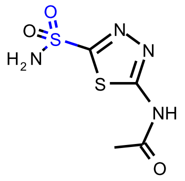 [*]S(=[*])(=O)[*]	-0.263	5 out of 22
ECFP_6	-797085356	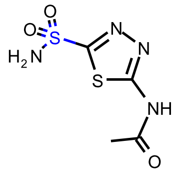 [*]S(=[*])(=[*])[*]	-0.263	5 out of 22
ECFP_6	866218936	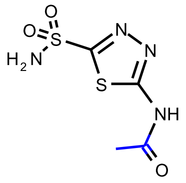 [*]C(=[*])C	-0.256	6 out of 26

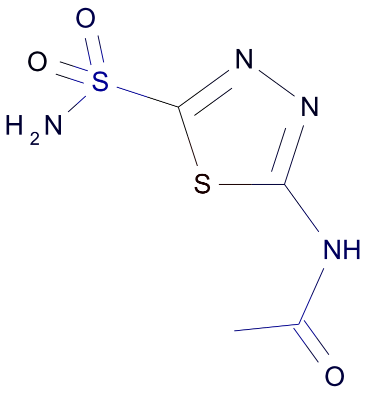

$C_4H_6N_4O_3S_2$
Molecular Weight: 222.24544
ALogP: -1.329
Rotatable Bonds: 2
Acceptors: 5
Donors: 2

Model Prediction

Prediction: Single-Carcinogen
Probability: 0.23
Enrichment: 0.562
Bayesian Score: -7.54
Mahalanobis Distance: 13.8
Mahalanobis Distance p-value: 1e-005

Prediction: Positive if the Bayesian score is above the estimated best cutoff value from minimizing the false positive and false negative rate.
Probability: The esimated probability that the sample is in the positive category. This assumes that the Bayesian score follows a normal distribution and is different from the prediction using a cutoff.
Enrichment: An estimate of enrichment, that is, the increased likelihood (versus random) of this sample being in the category.
Bayesian Score: The standard Laplacian-modified Bayesian score.
Mahalanobis Distance: The Mahalanobis distance (MD) is the distance to the center of the training data. The larger the MD, the less trustworthy the prediction.
Mahalanobis Distance p-value: The p-value gives the fraction of training data with an MD greater than or equal to the one for the given sample, assuming normally distributed data. The smaller the p-value, the less trustworthy the prediciton. For highly non-normal X properties (e.g., fingerprints), the MD p-value is wildly inaccurate.

Structural Similar Compounds

Name	Nitrofurazone	Niridazole	Dacarbazine
Structure	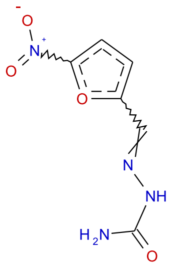	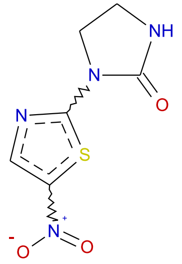	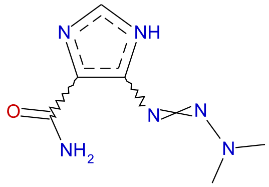
Actual Endpoint	Single-Carcinogen	Multiple-Carcinogen	Single-Carcinogen
Predicted Endpoint	Single-Carcinogen	Multiple-Carcinogen	Single-Carcinogen
Distance	0.675	0.763	0.804
Reference	US FDA (Centre for Drug Eval.& Res./Off. Testing & Res.) Sept. 1997	US FDA (Centre for Drug Eval.& Res./Off. Testing & Res.) Sept. 1997	US FDA (Centre for Drug Eval.& Res./Off. Testing & Res.) Sept. 1997

Model Applicability

Unknown features are fingerprint features in the query molecule, but not found or appearing too infrequently in the training set.

1. Molecular_FractionalPolarSurfaceArea out of range. Value: 0.738. Training min, max, mean, SD: 0, 0.632, 0.25695, 0.1555.
2. Unknown ECFP_2 feature: -1596132236: [*]N[c]1:n:[*]:[*]:s:1
3. Unknown ECFP_2 feature: 1221843808: [*]S(=[*])(=[*])[c]1:n:[*]:[*]:s:1

Feature Contribution

Top features for positive contribution

Fingerprint	Bit/Smiles	Feature Structure	Score	Multiple-Carcinogen in training set
ECFP_4	85262808	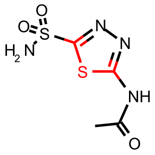 [*][c]1:[*]:[*]:[c]([*]):s:1	0.351	1 out of 1

ECFP_4	914325265	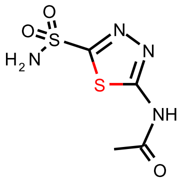 [*]:s:[*]	0.13	2 out of 4
**Top Features for negative contribution**				
**Fingerprint**	**Bit/Smiles**	**Feature Structure**	**Score**	**Multiple-Carcinogen in training set**
ECFP_4	866218936	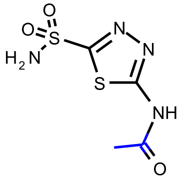 [*]C(=[*])C	-1.24	0 out of 6
ECFP_4	2102150379	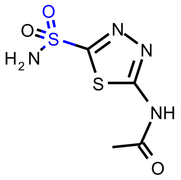 [*]S(=[*])(=O)[*]	-1.11	0 out of 5
ECFP_4	-797085356	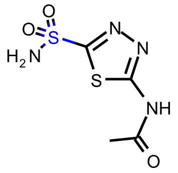 [*]S(=[*])(=[*])[*]	-1.11	0 out of 5

Comp. 14

TOPKAT_Mouse_Male_FDA_None_vs_Carcinogen

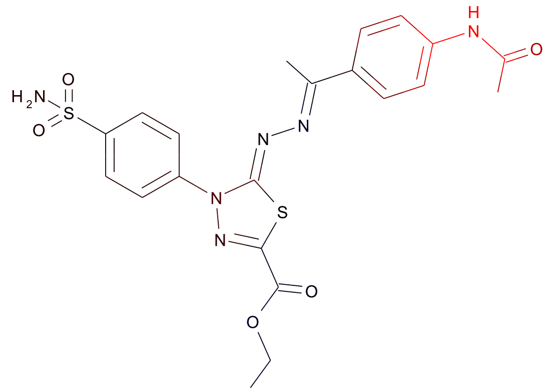

$C_{21}H_{22}N_6O_5S_2$

Molecular Weight: 502.56657

ALogP: 2.259

Rotatable Bonds: 8

Acceptors: 10

Donors: 2

Model Prediction

Prediction: Carcinogen

Probability: 0.448

Enrichment: 1.52

Bayesian Score: 4.33

Mahalanobis Distance: 13.3

Mahalanobis Distance p-value: 8.05e-005

Prediction: Positive if the Bayesian score is above the estimated best cutoff value from minimizing the false positive and false negative rate.
Probability: The esimated probability that the sample is in the positive category. This assumes that the Bayesian score follows a normal distribution and is different from the prediction using a cutoff.
Enrichment: An estimate of enrichment, that is, the increased likelihood (versus random) of this sample being in the category.
Bayesian Score: The standard Laplacian-modified Bayesian score.
Mahalanobis Distance: The Mahalanobis distance (MD) is the distance to the center of the training data. The larger the MD, the less trustworthy the prediction.
Mahalanobis Distance p-value: The p-value gives the fraction of training data with an MD greater than or equal to the one for the given sample, assuming normally distributed data. The smaller the p-value, the less trustworthy the prediciton. For highly non-normal X properties (e.g., fingerprints), the MD p-value is wildly inaccurate.

Structural Similar Compounds

Name	Bacampicillin	Sulfasalazine	Nimodipine
Structure	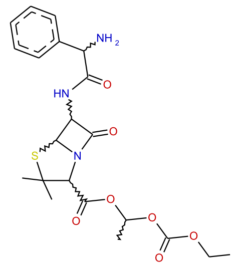	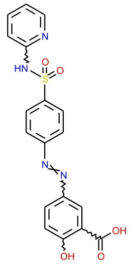	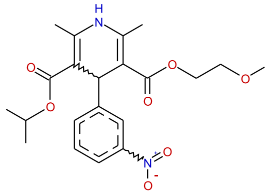
Actual Endpoint	Non-Carcinogen	Carcinogen	Non-Carcinogen
Predicted Endpoint	Non-Carcinogen	Carcinogen	Non-Carcinogen
Distance	0.613	0.760	0.762
Reference	US FDA (Centre for Drug Eval.& Res./Off. Testing & Res.) Sept. 1997	US FDA (Centre for Drug Eval.& Res./Off. Testing & Res.) Sept. 1997	US FDA (Centre for Drug Eval.& Res./Off. Testing & Res.) Sept. 1997

Model Applicability

Unknown features are fingerprint features in the query molecule, but not found or appearing too infrequently in the training set.

1. All properties and OPS components are within expected ranges.

Feature Contribution

Top features for positive contribution				
**Fingerprint**	**Bit/Smiles**	**Feature Structure**	**Score**	**Carcinogen in training set**
FCFP_6	-1944671191	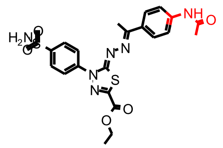 [*]:[c](:[*])NC(=O)C	0.891	4 out of 4

FCFP_6	1907952166	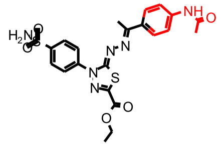 CC(=O)N[c]1:[cH]:[cH]:[*]:[cH]:[cH]:1	0.805	3 out of 3
FCFP_6	-451043714	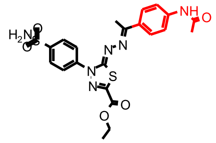 [*][c]1:[cH]:[cH]:[c](NC(=O)C):[cH]:[cH]:1	0.676	2 out of 2

Top Features for negative contribution

Fingerprint	Bit/Smiles	Feature Structure	Score	Carcinogen in training set
FCFP_6	-432846198	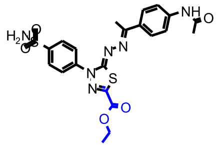 [*]C(=[*])C(=O)OCC	-0.551	2 out of 16
FCFP_6	-1549192822	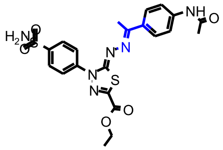 [*]\N=C(/C)\[c](:[*]):[*]	-0.489	3 out of 21
FCFP_6	-1272768868	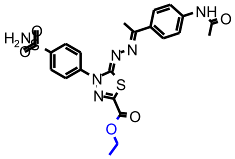 [*]OCC	-0.218	22 out of 105

Erlotinib

TOPKAT_Mouse_Male_FDA_None_vs_Carcinogen

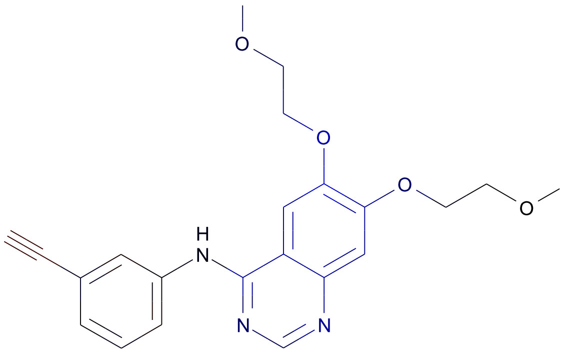

$C_{22}H_{23}N_3O_4$
Molecular Weight: 393.43572
ALogP: 4.309
Rotatable Bonds: 10
Acceptors: 7
Donors: 1

Model Prediction

Prediction: Non-Carcinogen
Probability: 0.181
Enrichment: 0.614
Bayesian Score: -6.29
Mahalanobis Distance: 16
Mahalanobis Distance p-value: 1.07e-009

Prediction: Positive if the Bayesian score is above the estimated best cutoff value from minimizing the false positive and false negative rate.
Probability: The esimated probability that the sample is in the positive category. This assumes that the Bayesian score follows a normal distribution and is different from the prediction using a cutoff.
Enrichment: An estimate of enrichment, that is, the increased likelihood (versus random) of this sample being in the category.
Bayesian Score: The standard Laplacian-modified Bayesian score.
Mahalanobis Distance: The Mahalanobis distance (MD) is the distance to the center of the training data. The larger the MD, the less trustworthy the prediction.
Mahalanobis Distance p-value: The p-value gives the fraction of training data with an MD greater than or equal to the one for the given sample, assuming normally distributed data. The smaller the p-value, the less trustworthy the prediciton. For highly non-normal X properties (e.g., fingerprints), the MD p-value is wildly inaccurate.

Structural Similar Compounds

Name	Mycophenolate	Nicardipine	Nimodipine
Structure	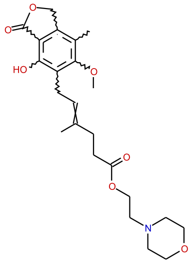	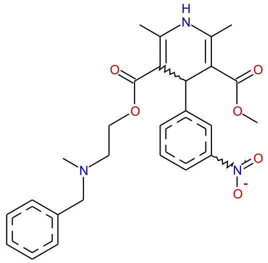	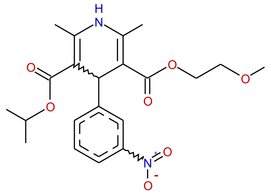
Actual Endpoint	Non-Carcinogen	Non-Carcinogen	Non-Carcinogen
Predicted Endpoint	Non-Carcinogen	Non-Carcinogen	Non-Carcinogen
Distance	0.615	0.655	0.668
Reference	US FDA (Centre for Drug Eval.& Res./Off. Testing & Res.) Sept. 1997	US FDA (Centre for Drug Eval.& Res./Off. Testing & Res.) Sept. 1997	US FDA (Centre for Drug Eval.& Res./Off. Testing & Res.) Sept. 1997

Model Applicability

Unknown features are fingerprint features in the query molecule, but not found or appearing too infrequently in the training set.

1. All properties and OPS components are within expected ranges.
2. Unknown FCFP_2 feature: 902193919: [*]:[c](:[*])C#C

Feature Contribution

Top features for positive contribution				
**Fingerprint**	**Bit/Smiles**	**Feature Structure**	**Score**	**Carcinogen in training set**
FCFP_6	131784192	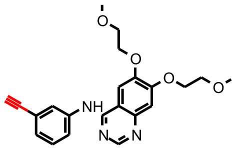 [*]C#C	0.983	8 out of 9

FCFP_6	-773983804	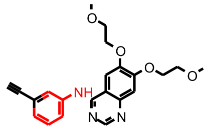 [*]N[c]1:[cH]:[*]:[c]([*]):[cH]:[cH]:1	0.409	10 out of 24
FCFP_6	-771557733	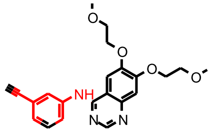 [*]N[c]1:[cH]:[*]:[cH]:[c](:[cH]:1)C#[*]	0.38	2 out of 4
**Top Features for negative contribution**				
**Fingerprint**	**Bit/Smiles**	**Feature Structure**	**Score**	**Carcinogen in training set**
FCFP_6	-124685461	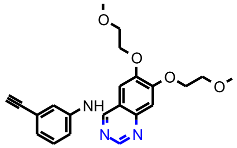 [*]:n:[cH]:n:[*]	-0.731	1 out of 12
FCFP_6	1293778554	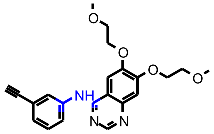 [*]:[c](:[*])N[c](:[*]):[*]	-0.719	0 out of 4
FCFP_6	1674955425	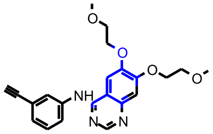 [*]O[c]1:[cH]:[c](:[c]([*]):[*]):[c](:[*]):[*]:[c]:1[*]	-0.719	0 out of 4

acetazolamide.cdx

TOPKAT_Mouse_Male_FDA_None_vs_Carcinogen

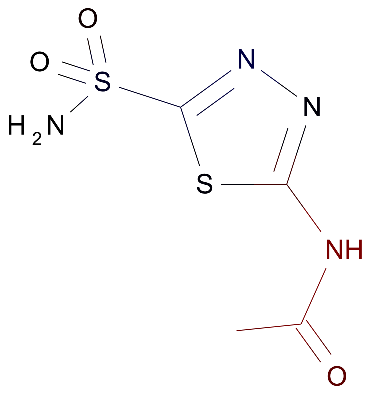

$C_4H_6N_4O_3S_2$
Molecular Weight: 222.24544
ALogP: -1.329
Rotatable Bonds: 2
Acceptors: 5
Donors: 2

Model Prediction

Prediction: Carcinogen
Probability: 0.406
Enrichment: 1.38
Bayesian Score: 3.18
Mahalanobis Distance: 11.5
Mahalanobis Distance p-value: 0.0218

Prediction: Positive if the Bayesian score is above the estimated best cutoff value from minimizing the false positive and false negative rate.
Probability: The esimated probability that the sample is in the positive category. This assumes that the Bayesian score follows a normal distribution and is different from the prediction using a cutoff.
Enrichment: An estimate of enrichment, that is, the increased likelihood (versus random) of this sample being in the category.
Bayesian Score: The standard Laplacian-modified Bayesian score.
Mahalanobis Distance: The Mahalanobis distance (MD) is the distance to the center of the training data. The larger the MD, the less trustworthy the prediction.
Mahalanobis Distance p-value: The p-value gives the fraction of training data with an MD greater than or equal to the one for the given sample, assuming normally distributed data. The smaller the p-value, the less trustworthy the prediciton. For highly non-normal X properties (e.g., fingerprints), the MD p-value is wildly inaccurate.

Structural Similar Compounds

Name	Nitrofurazone	Aminonitrothiazole	Azaserine
Structure	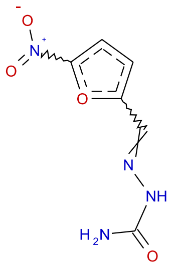	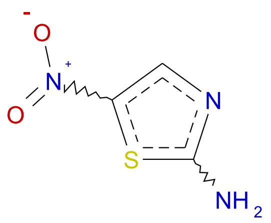	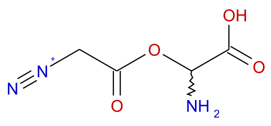
Actual Endpoint	Non-Carcinogen	Non-Carcinogen	Carcinogen
Predicted Endpoint	Carcinogen	Carcinogen	Carcinogen
Distance	0.649	0.663	0.710
Reference	US FDA (Centre for Drug Eval.& Res./Off. Testing & Res.) Sept. 1997	US FDA (Centre for Drug Eval.& Res./Off. Testing & Res.) Sept. 1997	US FDA (Centre for Drug Eval.& Res./Off. Testing & Res.) Sept. 1997

Model Applicability

Unknown features are fingerprint features in the query molecule, but not found or appearing too infrequently in the training set.

1. All properties and OPS components are within expected ranges.

Feature Contribution

Top features for positive contribution

Fingerprint	Bit/Smiles	Feature Structure	Score	Carcinogen in training set
FCFP_6	-1944671191	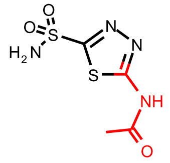 [*]:[c](:[*])NC(=O)C	0.891	4 out of 4

FCFP_6	1404307551	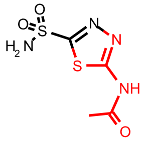 CC(=O)N[c]1:n:[*]:[*]:s:1	0.46	1 out of 1
FCFP_6	566058135	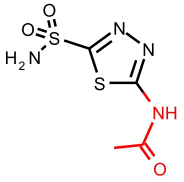 [*]NC(=O)C	0.447	17 out of 40
**Top Features for negative contribution**				
**Fingerprint**	**Bit/Smiles**	**Feature Structure**	**Score**	**Carcinogen in training set**
FCFP_6	86586436	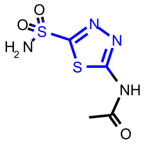 [*][c]1:n:n:[c](:s:1)S(=[*])(=[*])[*]	-0.719	0 out of 4
FCFP_6	-1055311551	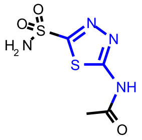 [*]N[c]1:n:n:[c]([*]):s:1	-0.233	0 out of 1
FCFP_6	-1539162406	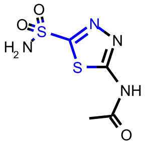 [*]S(=[*])(=[*])[c]1:n:[*]:[*]:s:1	-0.19	2 out of 10

Comp. 14

TOPKAT_Mouse_Male_FDA_Single_vs_Multiple

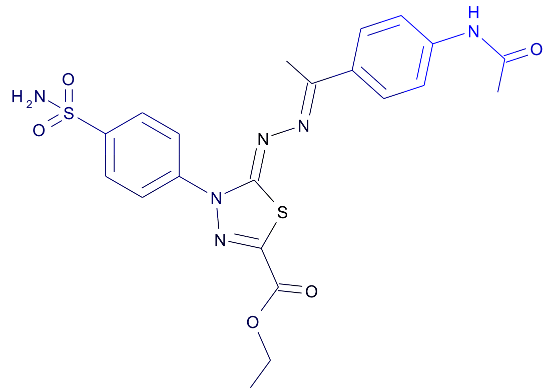

$C_{21}H_{22}N_6O_5S_2$

Molecular Weight: 502.56657

ALogP: 2.259

Rotatable Bonds: 8

Acceptors: 10

Donors: 2

Model Prediction

Prediction: Single-Carcinogen

Probability: 0.125

Enrichment: 0.414

Bayesian Score: -16.4

Mahalanobis Distance: 16.2

Mahalanobis Distance p-value: 2.78e-007

Prediction: Positive if the Bayesian score is above the estimated best cutoff value from minimizing the false positive and false negative rate.
Probability: The esimated probability that the sample is in the positive category. This assumes that the Bayesian score follows a normal distribution and is different from the prediction using a cutoff.
Enrichment: An estimate of enrichment, that is, the increased likelihood (versus random) of this sample being in the category.
Bayesian Score: The standard Laplacian-modified Bayesian score.
Mahalanobis Distance: The Mahalanobis distance (MD) is the distance to the center of the training data. The larger the MD, the less trustworthy the prediction.
Mahalanobis Distance p-value: The p-value gives the fraction of training data with an MD greater than or equal to the one for the given sample, assuming normally distributed data. The smaller the p-value, the less trustworthy the prediciton. For highly non-normal X properties (e.g., fingerprints), the MD p-value is wildly inaccurate.

Structural Similar Compounds

Name	Sulfasalazine	Dihydroxymethylfuratrizine	Reserpine
Structure	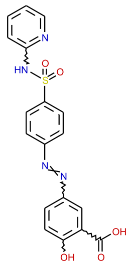	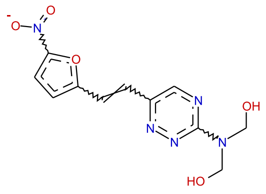	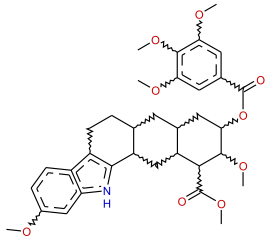
Actual Endpoint	Single-Carcinogen	Multiple-Carcinogen	Multiple-Carcinogen
Predicted Endpoint	Single-Carcinogen	Multiple-Carcinogen	Multiple-Carcinogen
Distance	0.833	0.948	0.962
Reference	US FDA (Centre for Drug Eval.& Res./Off. Testing & Res.) Sept. 1997	US FDA (Centre for Drug Eval.& Res./Off. Testing & Res.) Sept. 1997	US FDA (Centre for Drug Eval.& Res./Off. Testing & Res.) Sept. 1997

Model Applicability

Unknown features are fingerprint features in the query molecule, but not found or appearing too infrequently in the training set.

1. OPS PC12 out of range. Value: 2.4039. Training min, max, SD, explained variance: -3.4599, 2.3291, 1.246, 0.0290.

Feature Contribution

Top features for positive contribution

Fingerprint	Bit/Smiles	Feature Structure	Score	Multiple-Carcinogen in training set
FCFP_12	565998553	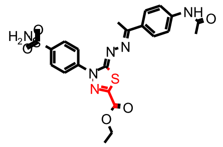 [*]C(=[*])C1=N[*][*]S 1	0.194	6 out of 14

FCFP_12	1070061035	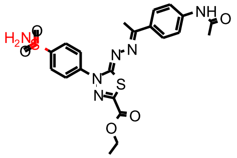 [*]S(=[*])(=[*])N	0.135	13 out of 33
FCFP_12	136597326	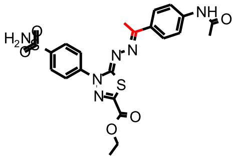 [*]C(=[*])C	0.0722	18 out of 49
**Top Features for negative contribution**				
**Fingerprint**	**Bit/Smiles**	**Feature Structure**	**Score**	**Multiple-Carcinogen in training set**
FCFP_12	1294255210	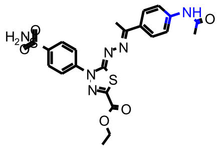 [*]C(=[*])N[c](:[*]):[*]	-1.63	0 out of 12
FCFP_12	1175665944	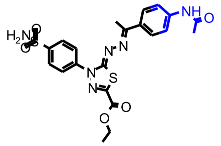 [*]:[cH]:[c](NC(=O)C):[cH]:[*]	-1.22	0 out of 7
FCFP_12	590925877	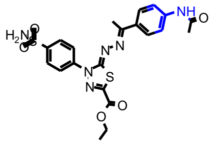 [*]N[c](:[cH]:[*]):[cH]:[*]	-0.998	1 out of 13

acetazolamide.cdx

TOPKAT_Mouse_Male_FDA_Single_vs_Multiple

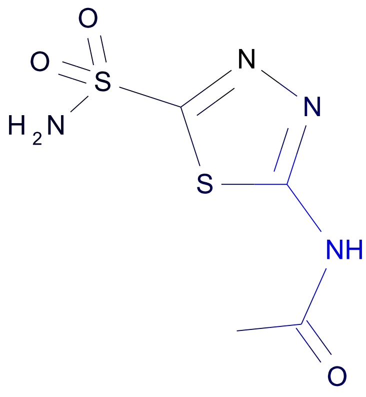

$C_4H_6N_4O_3S_2$
Molecular Weight: 222.24544
ALogP: -1.329
Rotatable Bonds: 2
Acceptors: 5
Donors: 2

Structural Similar Compounds

Name	Azaserine	Niridazole	Hydrochlorothiazide
Structure	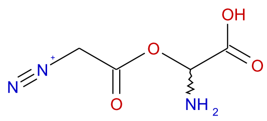	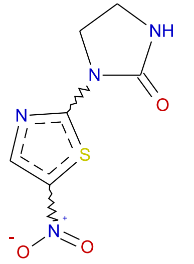	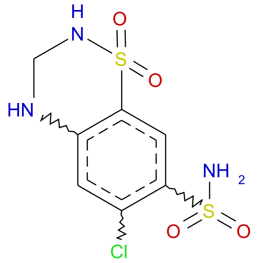
Actual Endpoint	Single-Carcinogen	Multiple-Carcinogen	Single-Carcinogen
Predicted Endpoint	Single-Carcinogen	Multiple-Carcinogen	Single-Carcinogen
Distance	0.714	0.745	0.772
Reference	US FDA (Centre for Drug Eval.& Res./Off. Testing & Res.) Sept. 1997	US FDA (Centre for Drug Eval.& Res./Off. Testing & Res.) Sept. 1997	US FDA (Centre for Drug Eval.& Res./Off. Testing & Res.) Sept. 1997

Model Prediction

Prediction: Single-Carcinogen
Probability: 0.15
Enrichment: 0.498
Bayesian Score: -7.39
Mahalanobis Distance: 13.8
Mahalanobis Distance p-value: 2.31e-005

Prediction: Positive if the Bayesian score is above the estimated best cutoff value from minimizing the false positive and false negative rate.
Probability: The esimated probability that the sample is in the positive category. This assumes that the Bayesian score follows a normal distribution and is different from the prediction using a cutoff.
Enrichment: An estimate of enrichment, that is, the increased likelihood (versus random) of this sample being in the category.
Bayesian Score: The standard Laplacian-modified Bayesian score.
Mahalanobis Distance: The Mahalanobis distance (MD) is the distance to the center of the training data. The larger the MD, the less trustworthy the prediction.
Mahalanobis Distance p-value: The p-value gives the fraction of training data with an MD greater than or equal to the one for the given sample, assuming normally distributed data. The smaller the p-value, the less trustworthy the prediciton. For highly non-normal X properties (e.g., fingerprints), the MD p-value is wildly inaccurate.

Model Applicability

Unknown features are fingerprint features in the query molecule, but not found or appearing too infrequently in the training set.

1. Molecular_FractionalPolarSurfaceArea out of range. Value: 0.738. Training min, max, mean, SD: 0, 0.723, 0.26638, 0.1689.

Feature Contribution

Top features for positive contribution

Fingerprint	Bit/Smiles	Feature Structure	Score	Multiple-Carcinogen in training set
FCFP_12	-1539162406	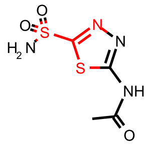 [*]S(=[*])(=[*])[c]1:n:[*]:[*]:s:1	0.174	1 out of 2

FCFP_12	1070061035	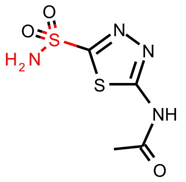 [*]S(=[*])(=[*])N	0.135	13 out of 33
FCFP_12	136597326	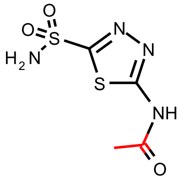 [*]C(=[*])C	0.0722	18 out of 49
**Top Features for negative contribution**				
**Fingerprint**	**Bit/Smiles**	**Feature Structure**	**Score**	**Multiple-Carcinogen in training set**
FCFP_12	1294255210	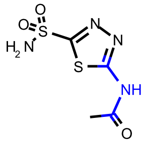 [*]C(=[*])N[c](:[*]):[*]	-1.63	0 out of 12
FCFP_12	-1151914249	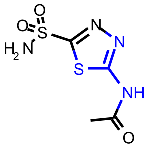 [*]N[c]1:n:[*]:[*]:s:1	-0.859	0 out of 4
FCFP_12	-1096219292	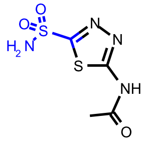 [*]:[c](:[*])S(=O)(=O)N	-0.859	0 out of 4

Comp. 14

TOPKAT_Ocular_Irritancy_Mild_vs_Moderate_Severe

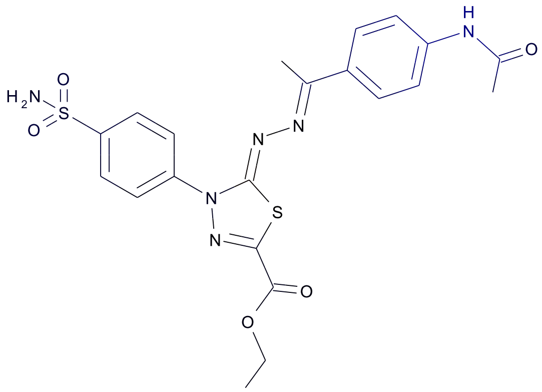

$C_{21}H_{22}N_6O_5S_2$

Molecular Weight: 502.56657

ALogP: 2.259

Rotatable Bonds: 8

Acceptors: 10

Donors: 2

Model Prediction

Prediction: Mild

Probability: 0.635

Enrichment: 0.921

Bayesian Score: -4.55

Mahalanobis Distance: 9.22

Mahalanobis Distance p-value: 0.392

Prediction: Positive if the Bayesian score is above the estimated best cutoff value from minimizing the false positive and false negative rate.
Probability: The esimated probability that the sample is in the positive category. This assumes that the Bayesian score follows a normal distribution and is different from the prediction using a cutoff.
Enrichment: An estimate of enrichment, that is, the increased likelihood (versus random) of this sample being in the category.
Bayesian Score: The standard Laplacian-modified Bayesian score.
Mahalanobis Distance: The Mahalanobis distance (MD) is the distance to the center of the training data. The larger the MD, the less trustworthy the prediction.
Mahalanobis Distance p-value: The p-value gives the fraction of training data with an MD greater than or equal to the one for the given sample, assuming normally distributed data. The smaller the p-value, the less trustworthy the prediciton. For highly non-normal X properties (e.g., fingerprints), the MD p-value is wildly inaccurate.

Structural Similar Compounds

Name	2;2'-Stilbenedisulfonic acid; 4;4'-dinitro-	2;7-NAPHTHALENE DISULFONIC ACID;4-AMINO-5-HYDROXY-;P-TOLUENE SULFONATE (ESTER)	2;7-Naphthalenedisulfonic acid;
Structure	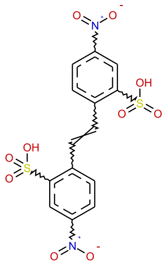	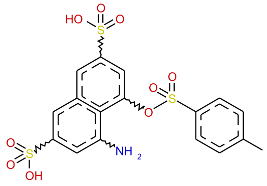	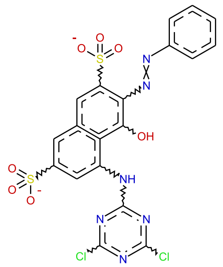
Actual Endpoint	Mild	Moderate_Severe	Moderate_Severe
Predicted Endpoint	Mild	Moderate_Severe	Moderate_Severe
Distance	0.744	0.747	0.823
Reference	Prehled Prumyslove Toxikologie; Organicke Latky; Marhold; J. pp 1062;86	28ZPAK-;194;72	Prehled Prumyslove Toxikologie; Organicke Latky; Marhold; J. - ;1300;86

Model Applicability

Unknown features are fingerprint features in the query molecule, but not found or appearing too infrequently in the training set.

1. All properties and OPS components are within expected ranges.

Feature Contribution

Top features for positive contribution

Fingerprint	Bit/Smiles	Feature Structure	Score	Moderate_Severe in training set
FCFP_10	-1036166322	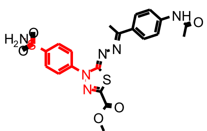 [*]S(=[*])(=[*])[c]1:[cH]:[cH]:[c](:[cH]:[cH]:1)N2N=[*][*]C2=[*]	0.256	2 out of 2

FCFP_10	1070061035	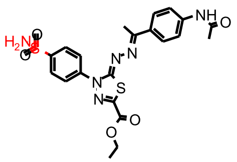 [*]S(=[*])(=[*])N	0.239	284 out of 338
FCFP_10	3	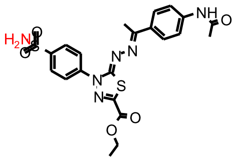 [*]N	0.165	383 out of 491
**Top Features for negative contribution**				
**Fingerprint**	**Bit/Smiles**	**Feature Structure**	**Score**	**Moderate_Severe in training set**
FCFP_10	-790336137	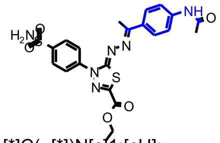 [*]C(=[*])N[c]1:[cH]:[cH]:[c](:[cH]:[cH]:1)C(=[*])[*]	-0.507	0 out of 1
FCFP_10	-1085821960	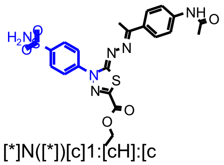 [*]N([*])[c]1:[cH]:[cH]:[c](:[cH]:[cH]:1)S(=O)(=O)N	-0.507	0 out of 1
FCFP_10	-178394671	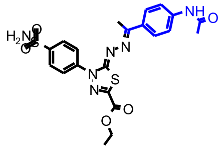 [*]C(=[*])[c]1:[cH]:[cH]:[c](NC(=O)C):[cH]:[cH]:1	-0.507	0 out of 1

Erlotinib

TOPKAT_Ocular_Irritancy_Mild_vs_Moderate_Severe

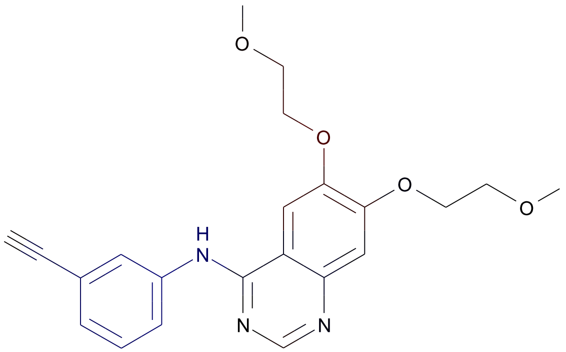

$C_{22}H_{23}N_3O_4$
Molecular Weight: 393.43572
ALogP: 4.309
Rotatable Bonds: 10
Acceptors: 7
Donors: 1

Structural Similar Compounds

Name	Benzoic acid; p-(N-butyl-2-(butylamino)acetamido)-; butyl ester;	COLCHICINE	Benzoic acid; p-(N-butyl-2-(piperidino)acetamido)-; butyl ester;
Structure	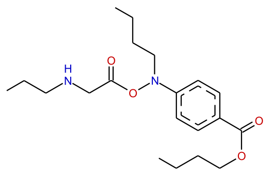	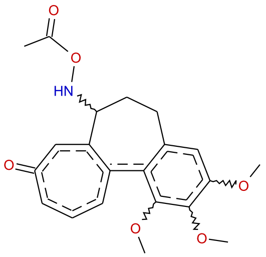	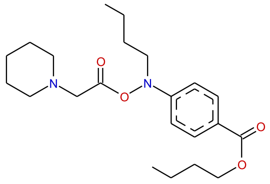
Actual Endpoint	Moderate_Severe	Moderate_Severe	Moderate_Severe
Predicted Endpoint	Moderate_Severe	Moderate_Severe	Moderate_Severe
Distance	0.648	0.707	0.750
Reference	Arzneimittel-Forschung 8;609;58	AJOPAA 31;837;48	Arzneimittel-Forschung 8;609;58

Model Prediction

Prediction: Mild
Probability: 0.766
Enrichment: 1.11
Bayesian Score: -2.1
Mahalanobis Distance: 10.4
Mahalanobis Distance p-value: 0.0368

Prediction: Positive if the Bayesian score is above the estimated best cutoff value from minimizing the false positive and false negative rate.
Probability: The esimated probability that the sample is in the positive category. This assumes that the Bayesian score follows a normal distribution and is different from the prediction using a cutoff.
Enrichment: An estimate of enrichment, that is, the increased likelihood (versus random) of this sample being in the category.
Bayesian Score: The standard Laplacian-modified Bayesian score.
Mahalanobis Distance: The Mahalanobis distance (MD) is the distance to the center of the training data. The larger the MD, the less trustworthy the prediction.
Mahalanobis Distance p-value: The p-value gives the fraction of training data with an MD greater than or equal to the one for the given sample, assuming normally distributed data. The smaller the p-value, the less trustworthy the prediciton. For highly non-normal X properties (e.g., fingerprints), the MD p-value is wildly inaccurate.

Model Applicability

Unknown features are fingerprint features in the query molecule, but not found or appearing too infrequently in the training set.

1. All properties and OPS components are within expected ranges.
2. Unknown FCFP_2 feature: -1151884458: [*]N[c](:n:[*]):[c](:[*]):[*]
3. Unknown FCFP_2 feature: -124685461: [*]:n:c:n:[*]
4. Unknown FCFP_2 feature: 902193919: [*]:[c](:[*])C#C

Feature Contribution

Top features for positive contribution				
**Fingerprint**	**Bit/Smiles**	**Feature Structure**	**Score**	**Moderate_Severe in training set**
FCFP_10	-1059904848	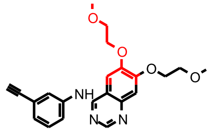 [*][c](:[*]):[c](OCCO C):[cH]:[*]	0.386	17 out of 17

FCFP_10	365650923	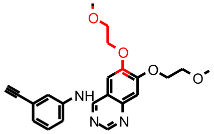<br>[*]OCCO[c](:[*]):[*]	0.386	17 out of 17
FCFP_10	-1716224640	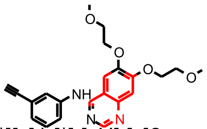<br>[*][c]1:[*]:[cH]:[c]2<br>:[c]([*]):[*]:[cH]:n<br>:[c]:2:[cH]:1	0.294	3 out of 3

Top Features for negative contribution

Fingerprint	Bit/Smiles	Feature Structure	Score	Moderate_Severe in training set
FCFP_10	-1699003333	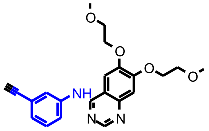<br>[*]N[c]1:[cH]:[cH]:[c<br>H]:[c](:[cH]:1)C#[*]	-1.09	2 out of 12
FCFP_10	1679603620	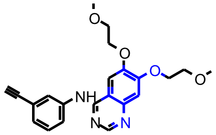<br>[*]O[c]1:[cH]:[c](:n:<br>[*]):[c](:[*]):[*]:[<br>c]:1[*]	-0.507	0 out of 1
FCFP_10	341504799	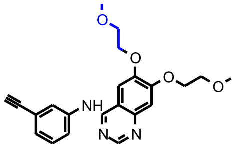<br>[*]CCOC	-0.425	7 out of 17

acetazolamide.cdx

TOPKAT_Ocular_Irritancy_Mild_vs_Moderate_Severe

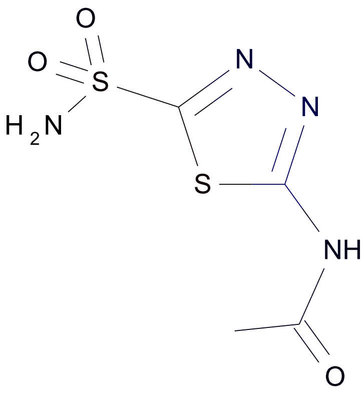

$C_4H_6N_4O_3S_2$
Molecular Weight: 222.24544
ALogP: -1.329
Rotatable Bonds: 2
Acceptors: 5
Donors: 2

Structural Similar Compounds

Name	SULFOACETIC ACID	ALLOXAN	Benzenesulfonic acid; 3;5-dicarboxy-; sodium salt
Structure	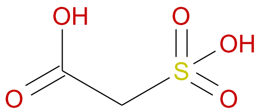	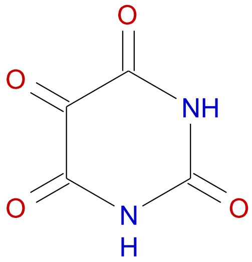	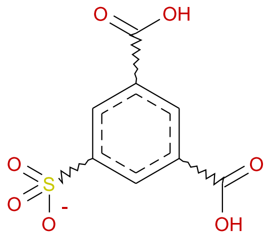
Actual Endpoint	Moderate_Severe	Mild	Moderate_Severe
Predicted Endpoint	Moderate_Severe	Mild	Moderate_Severe
Distance	0.594	0.614	0.700
Reference	JIHTAB 31;60;49	28ZPAK-;150;72	Prehled Prumyslove Toxikologie; Organicke Latky; Marhold; J. -;1056;86

Model Prediction

Prediction: Mild
Probability: 0.781
Enrichment: 1.13
Bayesian Score: -1.65
Mahalanobis Distance: 6.51
Mahalanobis Distance p-value: 1

Prediction: Positive if the Bayesian score is above the estimated best cutoff value from minimizing the false positive and false negative rate.
Probability: The esimated probability that the sample is in the positive category. This assumes that the Bayesian score follows a normal distribution and is different from the prediction using a cutoff.
Enrichment: An estimate of enrichment, that is, the increased likelihood (versus random) of this sample being in the category.
Bayesian Score: The standard Laplacian-modified Bayesian score.
Mahalanobis Distance: The Mahalanobis distance (MD) is the distance to the center of the training data. The larger the MD, the less trustworthy the prediction.
Mahalanobis Distance p-value: The p-value gives the fraction of training data with an MD greater than or equal to the one for the given sample, assuming normally distributed data. The smaller the p-value, the less trustworthy the prediciton. For highly non-normal X properties (e.g., fingerprints), the MD p-value is wildly inaccurate.

Model Applicability

Unknown features are fingerprint features in the query molecule, but not found or appearing too infrequently in the training set.

1. All properties and OPS components are within expected ranges.

Feature Contribution

Top features for positive contribution

Fingerprint	Bit/Smiles	Feature Structure	Score	Moderate_Severe in training set
FCFP_10	-1539162406	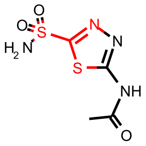 [*]S(=[*])(=[*])[c]1:n:[*]:[*]:s:1	0.294	3 out of 3

FCFP_10	1070061035	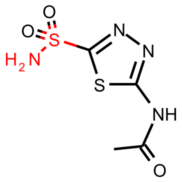 [*]S(=[*])(=[*])N	0.239	284 out of 338
FCFP_10	3	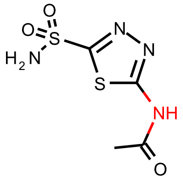 [*]N	0.165	383 out of 491

Top Features for negative contribution

**Fingerprint**	**Bit/Smiles**	**Feature Structure**	**Score**	**Moderate_Severe in training set**
FCFP_10	4427049	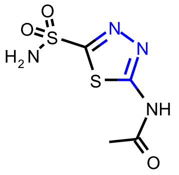 [*][c]1:[*]:[*]:n:n:1	-1.29	0 out of 4
FCFP_10	-1944671191	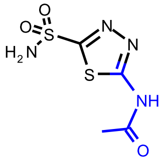 [*]:[c](:[*])NC(=O)C	-0.341	3 out of 7
FCFP_10	-1096219292	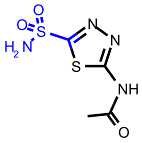 [*]:[c](:[*])S(=O)(=O<br>)N	-0.267	19 out of 38

Comp. 14

TOPKAT_Ocular_Irritancy_None_vs_Irritant

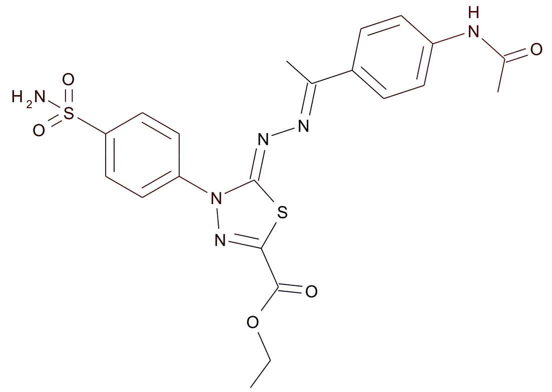

$C_{21}H_{22}N_6O_5S_2$
Molecular Weight: 502.56657
ALogP: 2.259
Rotatable Bonds: 8
Acceptors: 10
Donors: 2

Model Prediction

Prediction: Irritant
Probability: 1
Enrichment: 1.18
Bayesian Score: 2.66
Mahalanobis Distance: 9.42
Mahalanobis Distance p-value: 0.295

Prediction: Positive if the Bayesian score is above the estimated best cutoff value from minimizing the false positive and false negative rate.
Probability: The esimated probability that the sample is in the positive category. This assumes that the Bayesian score follows a normal distribution and is different from the prediction using a cutoff.
Enrichment: An estimate of enrichment, that is, the increased likelihood (versus random) of this sample being in the category.
Bayesian Score: The standard Laplacian-modified Bayesian score.
Mahalanobis Distance: The Mahalanobis distance (MD) is the distance to the center of the training data. The larger the MD, the less trustworthy the prediction.
Mahalanobis Distance p-value: The p-value gives the fraction of training data with an MD greater than or equal to the one for the given sample, assuming normally distributed data. The smaller the p-value, the less trustworthy the prediciton. For highly non-normal X properties (e.g., fingerprints), the MD p-value is wildly inaccurate.

Structural Similar Compounds

Name	2;2'-Stilbenedisulfonic acid; 4;4'-dinitro-	2;7-NAPHTHALENE DISULFONIC ACID;4-AMINO-5-HYDROXY-;P-TOLUENE SULFONATE (ESTER)	2;7-Naphthalenedisulfonic acid;
Structure	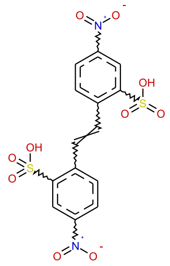	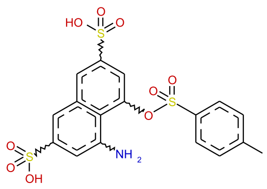	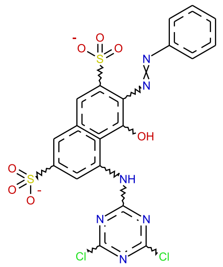
Actual Endpoint	Irritant	Irritant	Irritant
Predicted Endpoint	Irritant	Irritant	Irritant
Distance	0.748	0.750	0.809
Reference	Prehled Prumyslove Toxikologie; Organicke Latky; Marhold; J. pp 1062;86	28ZPAK-;194;72	Prehled Prumyslove Toxikologie; Organicke Latky; Marhold; J. - ;1300;86

Model Applicability

Unknown features are fingerprint features in the query molecule, but not found or appearing too infrequently in the training set.

1. All properties and OPS components are within expected ranges.

Feature Contribution

Top features for positive contribution

Fingerprint	Bit/Smiles	Feature Structure	Score	Irritant in training set
FCFP_12	1175665944	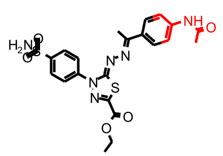 [*]:[cH]:[c](NC(=O)C):[cH]:[*]	0.198	14 out of 14

FCFP_12	-1944671191	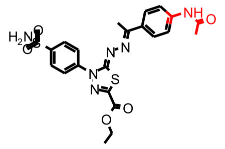 [*]:[c](:[*])NC(=O)C	0.184	7 out of 7
FCFP_12	675799546	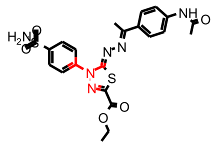 [*]=C1[*][*]=NN1[c](:[*]):[*]	0.184	7 out of 7

Top Features for negative contribution

Fingerprint	Bit/Smiles	Feature Structure	Score	Irritant in training set
FCFP_12	-432846198	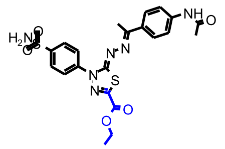 [*]C(=[*])C(=O)OCC	-0.229	91 out of 142
FCFP_12	565998553	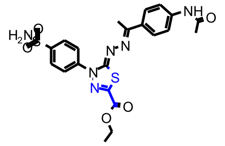 [*]C(=[*])C1=N[*][*]S1	-0.0662	198 out of 262
FCFP_12	65948508	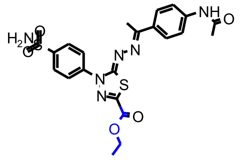 [*]C(=[*])OCC	-0.0658	68 out of 90

Erlotinib

TOPKAT_Ocular_Irritancy_None_vs_Irritant

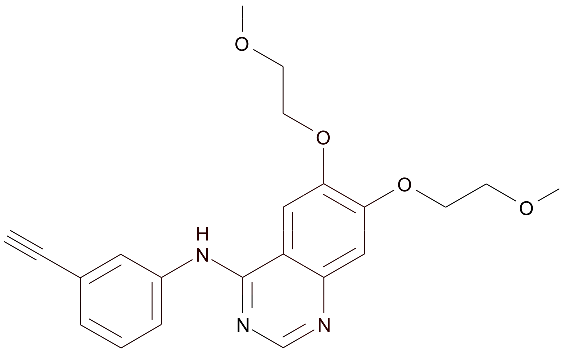

$C_{22}H_{23}N_3O_4$
Molecular Weight: 393.43572
ALogP: 4.309
Rotatable Bonds: 10
Acceptors: 7
Donors: 1

Structural Similar Compounds

Name	Benzoic acid; p-(N-butyl-2-(butylamino)acetamido)-; butyl ester;	COLCHICINE	Cinchoninamide; 2-butoxy-N-(2-(diethylamino)ethyl)-; monohydrochloride
Structure	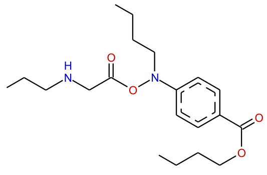	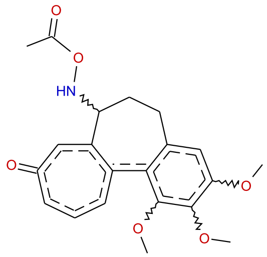	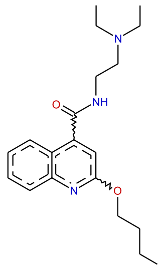
Actual Endpoint	Irritant	Irritant	Irritant
Predicted Endpoint	Non-Irritant	Irritant	Irritant
Distance	0.637	0.682	0.747
Reference	Arzneimittel-Forschung 8;609;58	AJOPAA 31;837;48	Arzneimittel-Forschung 8;181;58

Model Prediction

Prediction: Irritant
Probability: 1
Enrichment: 1.18
Bayesian Score: 2.13
Mahalanobis Distance: 9.75
Mahalanobis Distance p-value: 0.161

Prediction: Positive if the Bayesian score is above the estimated best cutoff value from minimizing the false positive and false negative rate.
Probability: The esimated probability that the sample is in the positive category. This assumes that the Bayesian score follows a normal distribution and is different from the prediction using a cutoff.
Enrichment: An estimate of enrichment, that is, the increased likelihood (versus random) of this sample being in the category.
Bayesian Score: The standard Laplacian-modified Bayesian score.
Mahalanobis Distance: The Mahalanobis distance (MD) is the distance to the center of the training data. The larger the MD, the less trustworthy the prediction.
Mahalanobis Distance p-value: The p-value gives the fraction of training data with an MD greater than or equal to the one for the given sample, assuming normally distributed data. The smaller the p-value, the less trustworthy the prediciton. For highly non-normal X properties (e.g., fingerprints), the MD p-value is wildly inaccurate.

Model Applicability

Unknown features are fingerprint features in the query molecule, but not found or appearing too infrequently in the training set.

1. All properties and OPS components are within expected ranges.
2. Unknown FCFP_2 feature: -1151884458: [*]N[c](:n:[*]):[c](:[*]):[*]
3. Unknown FCFP_2 feature: -124685461: [*]:n:c:n:[*]
4. Unknown FCFP_2 feature: 902193919: [*]:[c](:[*])C#C

Feature Contribution

Top features for positive contribution

Fingerprint	Bit/Smiles	Feature Structure	Score	Irritant in training set
FCFP_12	1747237384	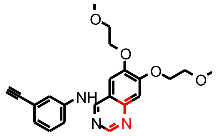 [*]:[cH]:n:[c](:[*]):[*]	0.208	44 out of 44

FCFP_12	178336375	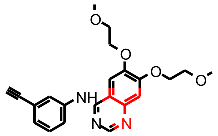 [*]:[cH]:[c](:n:[*]):[c](:[*]):[*]	0.202	19 out of 19
FCFP_12	17	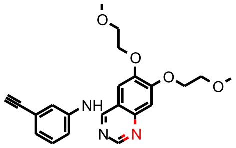 [*]:n:[*]	0.189	48 out of 49

Top Features for negative contribution

**Fingerprint**	**Bit/Smiles**	**Feature Structure**	**Score**	**Irritant in training set**
FCFP_12	-453677277	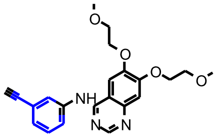 [*][c]1:[*]:[cH]:[c](:[cH]:[cH]:1)S(=[*])(=[*])[*]	0	264 out of 323
FCFP_12	991735244	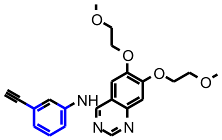 [*][c]1:[*]:[c]([*]):[cH]:[cH]:[cH]:1	0	237 out of 291
FCFP_12	-773983804	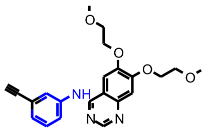 [*]N[c]1:[cH]:[*]:[c]([*]):[cH]:[cH]:1	0	102 out of 121

acetazolamide.cdx

TOPKAT_Ocular_Irritancy_None_vs_Irritant

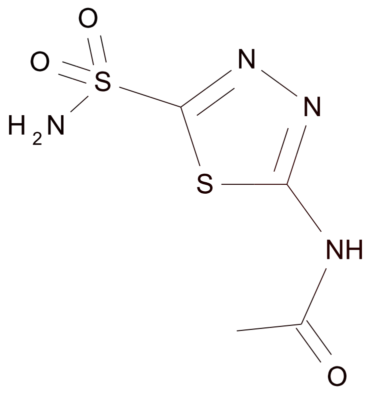

$C_4H_6N_4O_3S_2$
Molecular Weight: 222.24544
ALogP: -1.329
Rotatable Bonds: 2
Acceptors: 5
Donors: 2

Structural Similar Compounds

Name	SULFOACETIC ACID	ALLOXAN	SULFAMIC ACID AMINO ETHYL ESTER
Structure	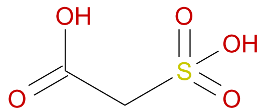	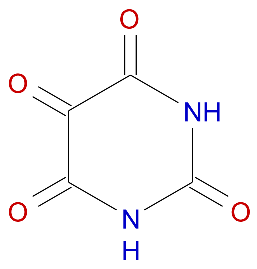	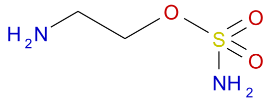
Actual Endpoint	Irritant	Irritant	Non-Irritant
Predicted Endpoint	Irritant	Irritant	Non-Irritant
Distance	0.587	0.605	0.610
Reference	JIHTAB 31;60;49	28ZPAK-;150;72	AIHAAP 23;95;62

Model Prediction

Prediction: Irritant
Probability: 1
Enrichment: 1.18
Bayesian Score: 1.72
Mahalanobis Distance: 6.52
Mahalanobis Distance p-value: 1

Prediction: Positive if the Bayesian score is above the estimated best cutoff value from minimizing the false positive and false negative rate.
Probability: The esimated probability that the sample is in the positive category. This assumes that the Bayesian score follows a normal distribution and is different from the prediction using a cutoff.
Enrichment: An estimate of enrichment, that is, the increased likelihood (versus random) of this sample being in the category.
Bayesian Score: The standard Laplacian-modified Bayesian score.
Mahalanobis Distance: The Mahalanobis distance (MD) is the distance to the center of the training data. The larger the MD, the less trustworthy the prediction.
Mahalanobis Distance p-value: The p-value gives the fraction of training data with an MD greater than or equal to the one for the given sample, assuming normally distributed data. The smaller the p-value, the less trustworthy the prediciton. For highly non-normal X properties (e.g., fingerprints), the MD p-value is wildly inaccurate.

Model Applicability

Unknown features are fingerprint features in the query molecule, but not found or appearing too infrequently in the training set.

1. All properties and OPS components are within expected ranges.

Feature Contribution

Top features for positive contribution

Fingerprint	Bit/Smiles	Feature Structure	Score	Irritant in training set
FCFP_12	1747237384	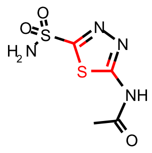 [*]:[cH]:n:[c](:[*]):[*]	0.208	44 out of 44

FCFP_12	-1151914249	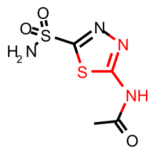 [*]N[c]1:n:[*]:[*]:s:1	0.19	9 out of 9
FCFP_12	17	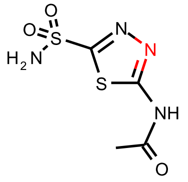 [*]:n:[*]	0.189	48 out of 49
**Top Features for negative contribution**				
**Fingerprint**	**Bit/Smiles**	**Feature Structure**	**Score**	**Irritant in training set**
FCFP_12	0	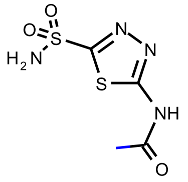 [*]C(=[*])[*]	0	1184 out of 1397
FCFP_12	1872154524	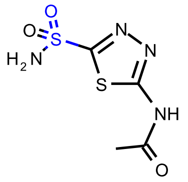 [*]C(=O)[*]	0	563 out of 690
FCFP_12	1	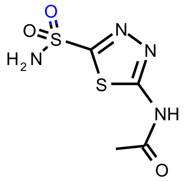 [*]S[*]	0	872 out of 1051

Comp. 14 **TOPKAT_Rat_Female_FDA_None_vs_Carcinogen**

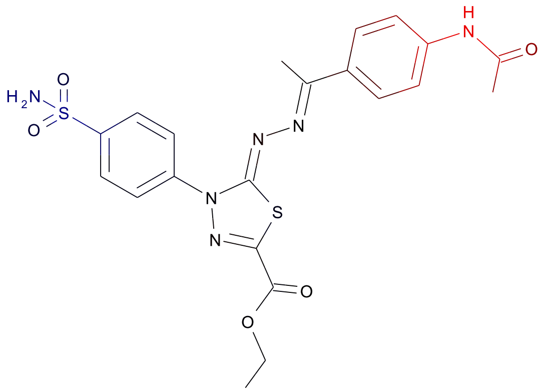

$C_{21}H_{22}N_6O_5S_2$
Molecular Weight: 502.56657
ALogP: 2.259
Rotatable Bonds: 8
Acceptors: 10
Donors: 2

Model Prediction

Prediction: Carcinogen
Probability: 0.3
Enrichment: 0.933
Bayesian Score: 0.0857
Mahalanobis Distance: 12.4
Mahalanobis Distance p-value: 0.0013

Prediction: Positive if the Bayesian score is above the estimated best cutoff value from minimizing the false positive and false negative rate.
Probability: The esimated probability that the sample is in the positive category. This assumes that the Bayesian score follows a normal distribution and is different from the prediction using a cutoff.
Enrichment: An estimate of enrichment, that is, the increased likelihood (versus random) of this sample being in the category.
Bayesian Score: The standard Laplacian-modified Bayesian score.
Mahalanobis Distance: The Mahalanobis distance (MD) is the distance to the center of the training data. The larger the MD, the less trustworthy the prediction.
Mahalanobis Distance p-value: The p-value gives the fraction of training data with an MD greater than or equal to the one for the given sample, assuming normally distributed data. The smaller the p-value, the less trustworthy the prediciton. For highly non-normal X properties (e.g., fingerprints), the MD p-value is wildly inaccurate.

Structural Similar Compounds

Name	Bacampicillin	Nimodipine	Carbenicillin
Structure	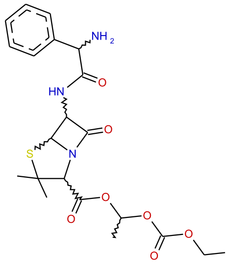	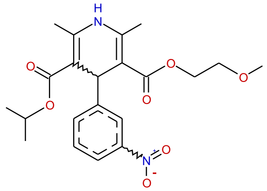	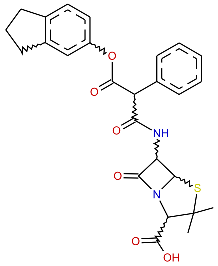
Actual Endpoint	Non-Carcinogen	Non-Carcinogen	Non-Carcinogen
Predicted Endpoint	Non-Carcinogen	Non-Carcinogen	Non-Carcinogen
Distance	0.653	0.790	0.799
Reference	US FDA (Centre for Drug Eval.& Res./Off. Testing & Res.) Sept. 1997	US FDA (Centre for Drug Eval.& Res./Off. Testing & Res.) Sept. 1997	US FDA (Centre for Drug Eval.& Res./Off. Testing & Res.) Sept. 1997

Model Applicability

Unknown features are fingerprint features in the query molecule, but not found or appearing too infrequently in the training set.

1. OPS PC4 out of range. Value: 5.1776. Training min, max, SD, explained variance: -5.3541, 4.9503, 2.055, 0.0443.
2. Unknown ECFP_2 feature: -934225701: [*]C(=[*])C1=N[*][*]S1
3. Unknown ECFP_2 feature: -1110911409: [*]=C1[*][*]=NN1[c](:[*]):[*]
4. Unknown ECFP_2 feature: 189949281: [*]\N=C\1/S[*]=[*]N1[*]
5. Unknown ECFP_2 feature: -819426257: [*]C(=NN=[*])[*]
6. Unknown ECFP_2 feature: 562081661: [*]C(=NN=[*])[*]
7. Unknown ECFP_2 feature: 128986386: [*]\N=C(/C)\[c](:[*]):[*]

Feature Contribution

Top features for positive contribution				
**Fingerprint**	**Bit/Smiles**	**Feature Structure**	**Score**	**Carcinogen in training set**

ECFP_12	1776488	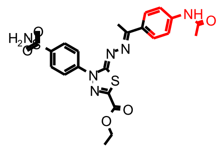 CC(=O)N[c]1:[cH]:[cH]:[*]:[cH]:[cH]:1	0.613	2 out of 2
ECFP_12	-847011520	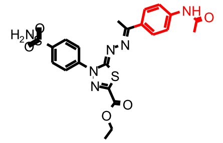 [*][c]1:[cH]:[cH]:[c](NC(=O)C):[cH]:[cH]:1	0.613	2 out of 2
ECFP_12	-474544785	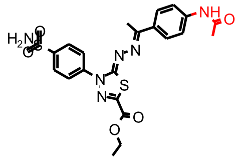 [*]NC(=O)C	0.575	3 out of 4
**Top Features for negative contribution**				
**Fingerprint**	**Bit/Smiles**	**Feature Structure**	**Score**	**Carcinogen in training set**
ECFP_12	-934226723	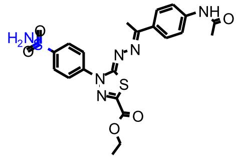 [*]S(=[*])(=[*])N	-1.16	0 out of 7
ECFP_12	-2121766239	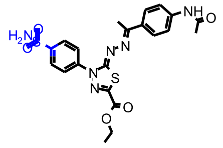 [*]:[c](:[*])S(=O)(=O)N	-1.06	0 out of 6

ECFP_12	-2137232509	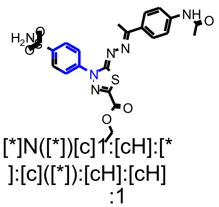 [*]N([*])[c]1:[cH]:[*]:[c]([*]):[cH]:[cH]:1	-0.485	0 out of 2

Erlotinib

TOPKAT_Rat_Female_FDA_None_vs_Carcinogen

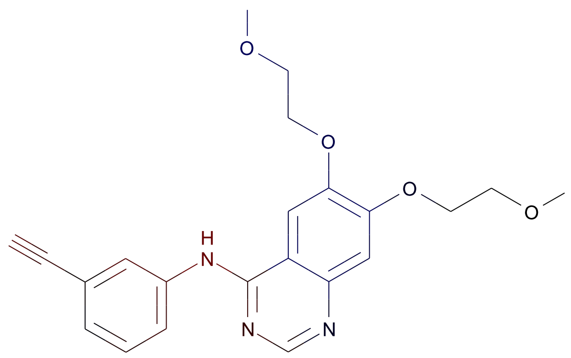

$C_{22}H_{23}N_3O_4$
Molecular Weight: 393.43572
ALogP: 4.309
Rotatable Bonds: 10
Acceptors: 7
Donors: 1

Model Prediction

Prediction: Carcinogen
Probability: 0.285
Enrichment: 0.885
Bayesian Score: -0.692
Mahalanobis Distance: 16.3
Mahalanobis Distance p-value: 1.65e-011

Prediction: Positive if the Bayesian score is above the estimated best cutoff value from minimizing the false positive and false negative rate.
Probability: The esimated probability that the sample is in the positive category. This assumes that the Bayesian score follows a normal distribution and is different from the prediction using a cutoff.
Enrichment: An estimate of enrichment, that is, the increased likelihood (versus random) of this sample being in the category.
Bayesian Score: The standard Laplacian-modified Bayesian score.
Mahalanobis Distance: The Mahalanobis distance (MD) is the distance to the center of the training data. The larger the MD, the less trustworthy the prediction.
Mahalanobis Distance p-value: The p-value gives the fraction of training data with an MD greater than or equal to the one for the given sample, assuming normally distributed data. The smaller the p-value, the less trustworthy the prediciton. For highly non-normal X properties (e.g., fingerprints), the MD p-value is wildly inaccurate.

Structural Similar Compounds

Name	Mycophenolate	Nicardipine	Nimodipine
Structure	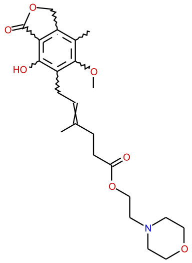	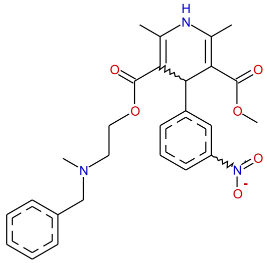	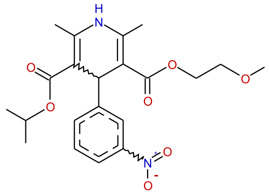
Actual Endpoint	Non-Carcinogen	Carcinogen	Non-Carcinogen
Predicted Endpoint	Non-Carcinogen	Carcinogen	Non-Carcinogen
Distance	0.644	0.660	0.675
Reference	US FDA (Centre for Drug Eval.& Res./Off. Testing & Res.) Sept. 1997	US FDA (Centre for Drug Eval.& Res./Off. Testing & Res.) Sept. 1997	US FDA (Centre for Drug Eval.& Res./Off. Testing & Res.) Sept. 1997

Model Applicability

Unknown features are fingerprint features in the query molecule, but not found or appearing too infrequently in the training set.

1. OPS PC28 out of range. Value: 3.6564. Training min, max, SD, explained variance: -2.8936, 3.5771, 1.028, 0.0111.
2. Unknown ECFP_2 feature: 1139738044: [*]:[c](:[*])C#C

Feature Contribution

Top features for positive contribution

Fingerprint	Bit/Smiles	Feature Structure	Score	Carcinogen in training set
ECFP_12	-1545539812	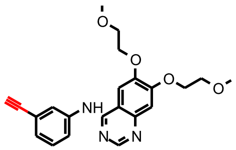 [*]C#C	0.78	8 out of 10

ECFP_12	-1939823063	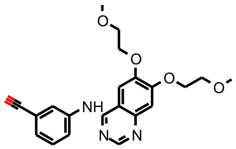 [*]#C	0.78	8 out of 10
ECFP_12	-177077903	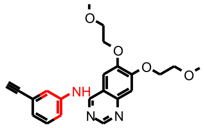 [*]N[c](:[cH]:[*]):[cH]:[*]	0.529	6 out of 10

Top Features for negative contribution

Fingerprint	Bit/Smiles	Feature Structure	Score	Carcinogen in training set
ECFP_12	301015111	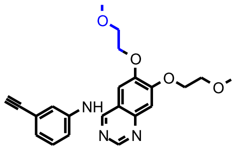 [*]CCOC	-0.661	0 out of 3
ECFP_12	-2063202154	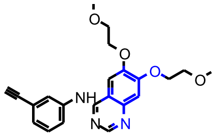 [*]O[c]1:[cH]:[c](:n:[*]):[c](:[*]):[*]:[c]:1[*]	-0.661	0 out of 3
ECFP_12	-2063600634	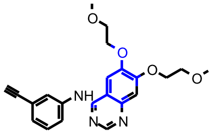 [*]O[c]1:[cH]:[c](:[c]([*]):[*]):[c](:[*]):[*]:[c]:1[*]	-0.661	0 out of 3

acetazolamide.cdx

TOPKAT_Rat_Female_FDA_None_vs_Carcinogen

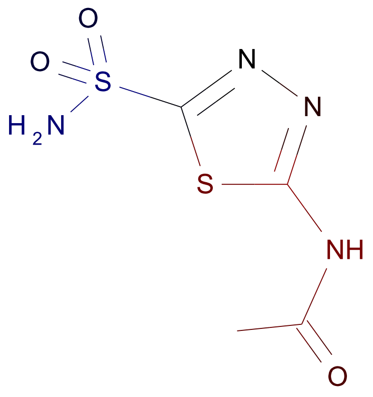

$C_4H_6N_4O_3S_2$
Molecular Weight: 222.24544
ALogP: -1.329
Rotatable Bonds: 2
Acceptors: 5
Donors: 2

Model Prediction

Prediction: Carcinogen
Probability: 0.349
Enrichment: 1.09
Bayesian Score: 2.25
Mahalanobis Distance: 12.6
Mahalanobis Distance p-value: 0.000672

Prediction: Positive if the Bayesian score is above the estimated best cutoff value from minimizing the false positive and false negative rate.
Probability: The esimated probability that the sample is in the positive category. This assumes that the Bayesian score follows a normal distribution and is different from the prediction using a cutoff.
Enrichment: An estimate of enrichment, that is, the increased likelihood (versus random) of this sample being in the category.
Bayesian Score: The standard Laplacian-modified Bayesian score.
Mahalanobis Distance: The Mahalanobis distance (MD) is the distance to the center of the training data. The larger the MD, the less trustworthy the prediction.
Mahalanobis Distance p-value: The p-value gives the fraction of training data with an MD greater than or equal to the one for the given sample, assuming normally distributed data. The smaller the p-value, the less trustworthy the prediciton. For highly non-normal X properties (e.g., fingerprints), the MD p-value is wildly inaccurate.

Structural Similar Compounds

Name	Nitrofurazone	Furidazina	Aminonitrothiazole
Structure	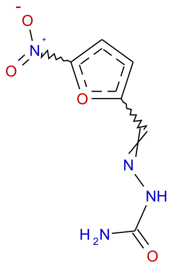	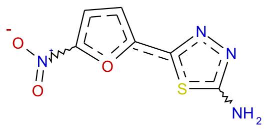	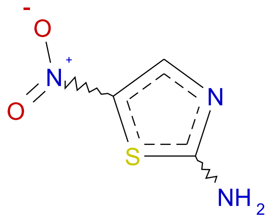
Actual Endpoint	Carcinogen	Carcinogen	Carcinogen
Predicted Endpoint	Carcinogen	Carcinogen	Carcinogen
Distance	0.689	0.708	0.709
Reference	US FDA (Centre for Drug Eval.& Res./Off. Testing & Res.) Sept. 1997	US FDA (Centre for Drug Eval.& Res./Off. Testing & Res.) Sept. 1997	US FDA (Centre for Drug Eval.& Res./Off. Testing & Res.) Sept. 1997

Model Applicability

Unknown features are fingerprint features in the query molecule, but not found or appearing too infrequently in the training set.

1. All properties and OPS components are within expected ranges.
2. Unknown ECFP_2 feature: 1221843808: [*]S(=[*])(=[*])[c]1:n:[*]:[*]:s:1

Feature Contribution

Top features for positive contribution

Fingerprint	Bit/Smiles	Feature Structure	Score	Carcinogen in training set
ECFP_12	85262808	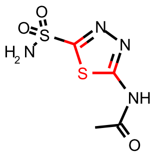 [*][c]1:[*]:[*]:[c]([*]):s:1	0.851	5 out of 5

ECFP_12	-474544785	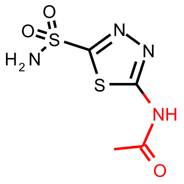 [*]NC(=O)C	0.575	3 out of 4
ECFP_12	-1923054811	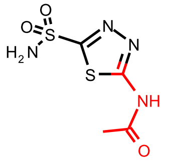 [*]:[c](:[*])NC(=O)C	0.575	3 out of 4

Top Features for negative contribution

Fingerprint	Bit/Smiles	Feature Structure	Score	Carcinogen in training set
ECFP_12	-934226723	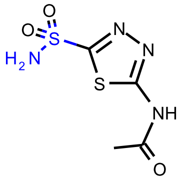 [*]S(=[*])(=[*])N	-1.16	0 out of 7
ECFP_12	-2121766239	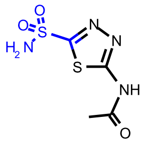 [*]:[c](:[*])S(=O)(=O)N	-1.06	0 out of 6
ECFP_12	-1897341097	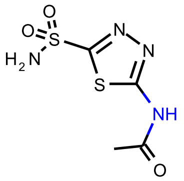 [*]N[*]	-0.096	30 out of 106

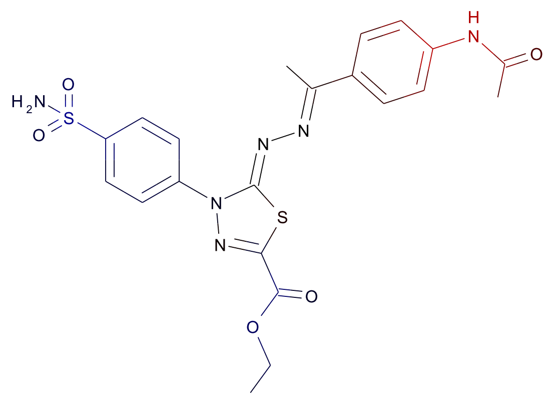

$C_{21}H_{22}N_6O_5S_2$
Molecular Weight: 502.56657
ALogP: 2.259
Rotatable Bonds: 8
Acceptors: 10
Donors: 2

Model Prediction

Prediction: Multiple-Carcinogen
Probability: 0.515
Enrichment: 1.38
Bayesian Score: 0.291
Mahalanobis Distance: 13.8
Mahalanobis Distance p-value: 3.91e-005

Prediction: Positive if the Bayesian score is above the estimated best cutoff value from minimizing the false positive and false negative rate.
Probability: The esimated probability that the sample is in the positive category. This assumes that the Bayesian score follows a normal distribution and is different from the prediction using a cutoff.
Enrichment: An estimate of enrichment, that is, the increased likelihood (versus random) of this sample being in the category.
Bayesian Score: The standard Laplacian-modified Bayesian score.
Mahalanobis Distance: The Mahalanobis distance (MD) is the distance to the center of the training data. The larger the MD, the less trustworthy the prediction.
Mahalanobis Distance p-value: The p-value gives the fraction of training data with an MD greater than or equal to the one for the given sample, assuming normally distributed data. The smaller the p-value, the less trustworthy the prediciton. For highly non-normal X properties (e.g., fingerprints), the MD p-value is wildly inaccurate.

Structural Similar Compounds

Name	Lasiocarpine	Nicardipine	Symphylline
Structure	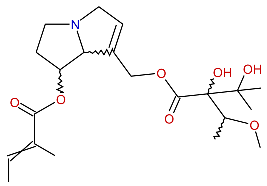	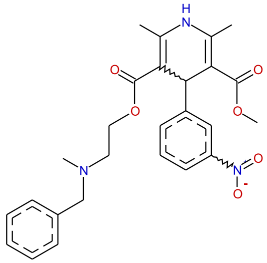	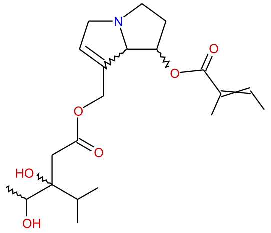
Actual Endpoint	Single-Carcinogen	Single-Carcinogen	Single-Carcinogen
Predicted Endpoint	Single-Carcinogen	Single-Carcinogen	Single-Carcinogen
Distance	0.799	0.799	0.874
Reference	US FDA (Centre for Drug Eval.& Res./Off. Testing & Res.) Sept. 1997	US FDA (Centre for Drug Eval.& Res./Off. Testing & Res.) Sept. 1997	US FDA (Centre for Drug Eval.& Res./Off. Testing & Res.) Sept. 1997

Model Applicability

Unknown features are fingerprint features in the query molecule, but not found or appearing too infrequently in the training set.

1. Num_H_Acceptors out of range. Value: 10. Training min, max, mean, SD: 0, 9, 3.9512, 2.048.
2. OPS PC13 out of range. Value: -3.944. Training min, max, SD, explained variance: -2.7175, 3.5682, 1.245, 0.0229.
3. OPS PC15 out of range. Value: 3.4113. Training min, max, SD, explained variance: -2.9572, 2.6953, 1.089, 0.0176.

Feature Contribution

Top features for positive contribution

Fingerprint	Bit/Smiles	Feature Structure	Score	Multiple-Carcinogen in training set
SCFP_4	2097618059	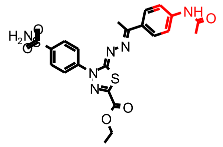 [*]:[cH]:[c](NC(=O)C):[cH]:[*]	0.73	5 out of 6

SCFP_4	1631845520	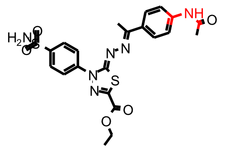 [*]C(=[*])N[c](:[*]):[*]	0.601	6 out of 9
SCFP_4	17	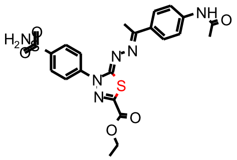 [*]S[*]	0.548	10 out of 17

Top Features for negative contribution

**Fingerprint**	**Bit/Smiles**	**Feature Structure**	**Score**	**Multiple-Carcinogen in training set**
SCFP_4	798581009	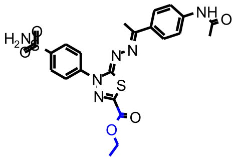 [*]C(=[*])OCC	-0.666	0 out of 3
SCFP_4	182991870	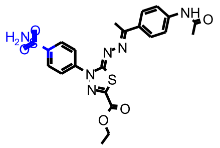 [*]:[c](:[*])S(=O)(=O)N	-0.666	0 out of 3
SCFP_4	1132907712	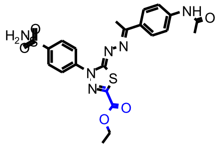 [*]OC(=O)C(=[*])[*]	-0.651	1 out of 9

Erlotinib

TOPKAT_Rat_Female_FDA_Single_vs_Multiple

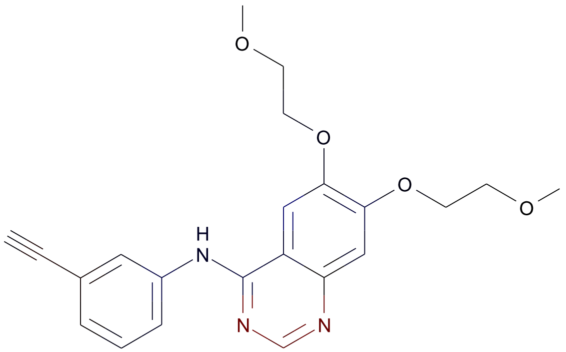

$C_{22}H_{23}N_3O_4$

Molecular Weight: 393.43572

ALogP: 4.309

Rotatable Bonds: 10

Acceptors: 7

Donors: 1

Structural Similar Compounds

Name	Nicardipine	Diltiazem	Moricizine
Structure	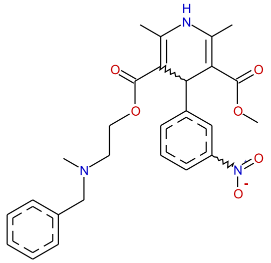	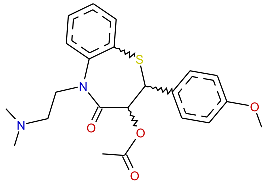	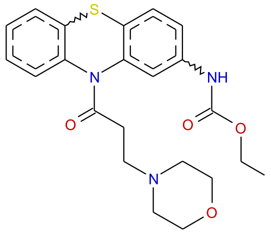
Actual Endpoint	Single-Carcinogen	Multiple-Carcinogen	Single-Carcinogen
Predicted Endpoint	Single-Carcinogen	Multiple-Carcinogen	Single-Carcinogen
Distance	0.632	0.748	0.749
Reference	US FDA (Centre for Drug Eval.& Res./Off. Testing & Res.) Sept. 1997	US FDA (Centre for Drug Eval.& Res./Off. Testing & Res.) Sept. 1997	US FDA (Centre for Drug Eval.& Res./Off. Testing & Res.) Sept. 1997

Model Prediction

Prediction: Single-Carcinogen

Probability: 0.423

Enrichment: 1.13

Bayesian Score: -2.63

Mahalanobis Distance: 15.2

Mahalanobis Distance p-value: 2.08e-006

Prediction: Positive if the Bayesian score is above the estimated best cutoff value from minimizing the false positive and false negative rate.
Probability: The esimated probability that the sample is in the positive category. This assumes that the Bayesian score follows a normal distribution and is different from the prediction using a cutoff.
Enrichment: An estimate of enrichment, that is, the increased likelihood (versus random) of this sample being in the category.
Bayesian Score: The standard Laplacian-modified Bayesian score.
Mahalanobis Distance: The Mahalanobis distance (MD) is the distance to the center of the training data. The larger the MD, the less trustworthy the prediction.
Mahalanobis Distance p-value: The p-value gives the fraction of training data with an MD greater than or equal to the one for the given sample, assuming normally distributed data. The smaller the p-value, the less trustworthy the prediciton. For highly non-normal X properties (e.g., fingerprints), the MD p-value is wildly inaccurate.

Model Applicability

Unknown features are fingerprint features in the query molecule, but not found or appearing too infrequently in the training set.

1. All properties and OPS components are within expected ranges.

Feature Contribution

Top features for positive contribution

Fingerprint	Bit/Smiles	Feature Structure	Score	Multiple-Carcinogen in training set
SCFP_4	-1065373877	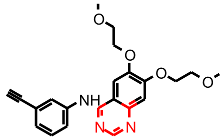 [*][c]1:[*]:[c](:[*]):n:[cH]:n:1	0.721	3 out of 3

SCFP_4	-1181430618	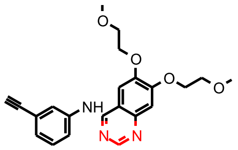<br>[*]:n:[cH]:n:[*]	0.663	4 out of 5
SCFP_4	2142015375	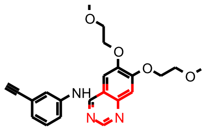<br>[*]:[cH]:[c]1:n:[cH]:<br>n:[*]:[c]:1:[*]	0.433	2 out of 3
**Top Features for negative contribution**				
**Fingerprint**	**Bit/Smiles**	**Feature Structure**	**Score**	**Multiple-Carcinogen in training set**
SCFP_4	622342378	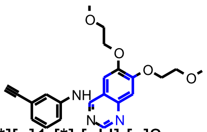<br>[*][c]1:[*]:[cH]:[c]2<br>:[c]([*]):[*]:[cH]:n<br>:[c]:2:[cH]:1	-0.816	0 out of 4
SCFP_4	112346096	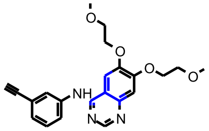<br>[*][c](:[*]):[c](:[cH<br>]:[*]):[c](:[*]):[*]	-0.73	1 out of 10
SCFP_4	1242547645	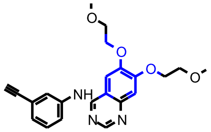<br>[*]CO[c]1:[cH]:[c](:[<br>*]):[*]:[cH]:[c]:1O[<br>*]	-0.489	0 out of 2

acetazolamide.cdx

TOPKAT_Rat_Female_FDA_Single_vs_Multiple

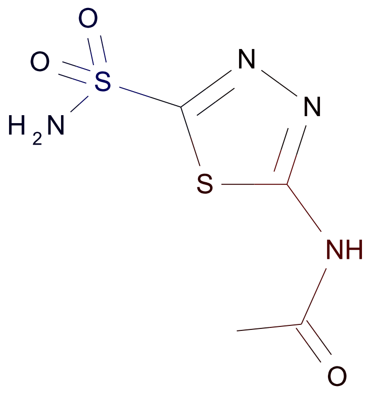

$C_4H_6N_4O_3S_2$

Molecular Weight: 222.24544

ALogP: -1.329

Rotatable Bonds: 2

Acceptors: 5

Donors: 2

Model Prediction

Prediction: Multiple-Carcinogen

Probability: 0.55

Enrichment: 1.47

Bayesian Score: 2.16

Mahalanobis Distance: 9.91

Mahalanobis Distance p-value: 0.0701

Prediction: Positive if the Bayesian score is above the estimated best cutoff value from minimizing the false positive and false negative rate.
Probability: The esimated probability that the sample is in the positive category. This assumes that the Bayesian score follows a normal distribution and is different from the prediction using a cutoff.
Enrichment: An estimate of enrichment, that is, the increased likelihood (versus random) of this sample being in the category.
Bayesian Score: The standard Laplacian-modified Bayesian score.
Mahalanobis Distance: The Mahalanobis distance (MD) is the distance to the center of the training data. The larger the MD, the less trustworthy the prediction.
Mahalanobis Distance p-value: The p-value gives the fraction of training data with an MD greater than or equal to the one for the given sample, assuming normally distributed data. The smaller the p-value, the less trustworthy the prediciton. For highly non-normal X properties (e.g., fingerprints), the MD p-value is wildly inaccurate.

Structural Similar Compounds

Name	Nitrofurazone	Niridazole	Furidazina
Structure	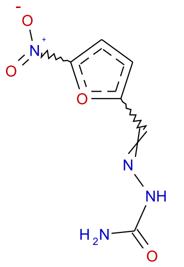	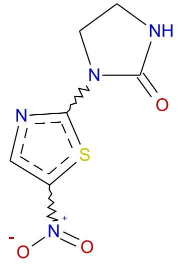	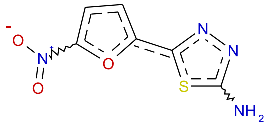
Actual Endpoint	Multiple-Carcinogen	Multiple-Carcinogen	Single-Carcinogen
Predicted Endpoint	Multiple-Carcinogen	Multiple-Carcinogen	Multiple-Carcinogen
Distance	0.676	0.696	0.713
Reference	US FDA (Centre for Drug Eval.& Res./Off. Testing & Res.) Sept. 1997	US FDA (Centre for Drug Eval.& Res./Off. Testing & Res.) Sept. 1997	US FDA (Centre for Drug Eval.& Res./Off. Testing & Res.) Sept. 1997

Model Applicability

Unknown features are fingerprint features in the query molecule, but not found or appearing too infrequently in the training set.

1. All properties and OPS components are within expected ranges.

Feature Contribution

Top features for positive contribution

Fingerprint	Bit/Smiles	Feature Structure	Score	Multiple-Carcinogen in training set
SCFP_4	1631845520	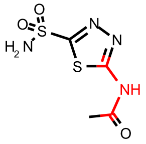 [*]C(=[*])N[c](:[*]):[*]	0.601	6 out of 9

SCFP_4	17	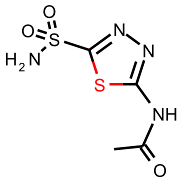 [*]S[*]	0.548	10 out of 17
SCFP_4	1310748454	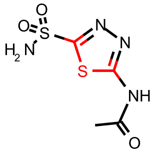 [*][c]1:[*]:[*]:[c]([*]):s:1	0.444	4 out of 7
**Top Features for negative contribution**				
**Fingerprint**	**Bit/Smiles**	**Feature Structure**	**Score**	**Multiple-Carcinogen in training set**
SCFP_4	182991870	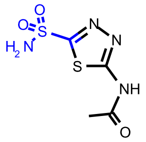 [*]:[c](:[*])S(=O)(=O)N	-0.666	0 out of 3
SCFP_4	1311429347	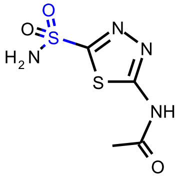 [*]S(=[*])(=O)[*]	-0.566	1 out of 8
SCFP_4	21	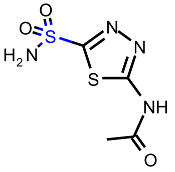 [*]S(=[*])(=[*])[*]	-0.566	1 out of 8

Comp. 14

TOPKAT_Rat_Male_FDA_None_vs_Carcinogen

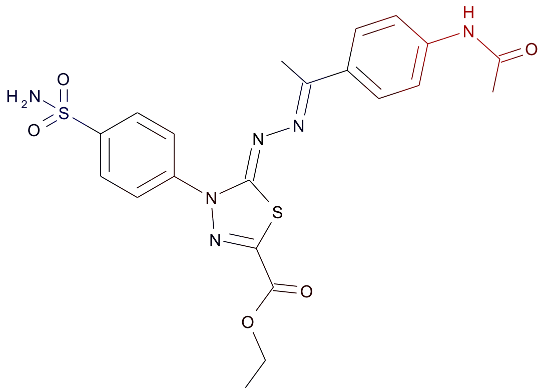

$C_{21}H_{22}N_6O_5S_2$

Molecular Weight: 502.56657

ALogP: 2.259

Rotatable Bonds: 8

Acceptors: 10

Donors: 2

Model Prediction

Prediction: Carcinogen

Probability: 0.381

Enrichment: 1.14

Bayesian Score: 0.825

Mahalanobis Distance: 16

Mahalanobis Distance p-value: 4.91e-009

Prediction: Positive if the Bayesian score is above the estimated best cutoff value from minimizing the false positive and false negative rate.
Probability: The esimated probability that the sample is in the positive category. This assumes that the Bayesian score follows a normal distribution and is different from the prediction using a cutoff.
Enrichment: An estimate of enrichment, that is, the increased likelihood (versus random) of this sample being in the category.
Bayesian Score: The standard Laplacian-modified Bayesian score.
Mahalanobis Distance: The Mahalanobis distance (MD) is the distance to the center of the training data. The larger the MD, the less trustworthy the prediction.
Mahalanobis Distance p-value: The p-value gives the fraction of training data with an MD greater than or equal to the one for the given sample, assuming normally distributed data. The smaller the p-value, the less trustworthy the prediciton. For highly non-normal X properties (e.g., fingerprints), the MD p-value is wildly inaccurate.

Structural Similar Compounds

Name	Bacampicillin	Nimodipine	Sulfasalazine
Structure	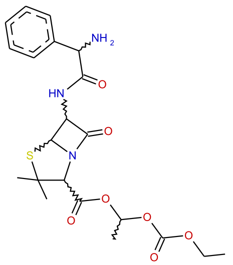	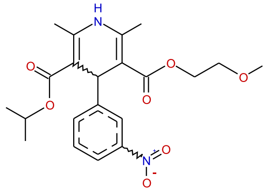	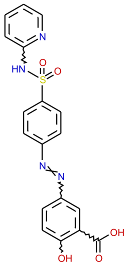
Actual Endpoint	Non-Carcinogen	Carcinogen	Carcinogen
Predicted Endpoint	Non-Carcinogen	Carcinogen	Carcinogen
Distance	0.635	0.764	0.771
Reference	US FDA (Centre for Drug Eval.& Res./Off. Testing & Res.) Sept. 1997	US FDA (Centre for Drug Eval.& Res./Off. Testing & Res.) Sept. 1997	US FDA (Centre for Drug Eval.& Res./Off. Testing & Res.) Sept. 1997

Model Applicability

Unknown features are fingerprint features in the query molecule, but not found or appearing too infrequently in the training set.

1. OPS PC4 out of range. Value: 6.2948. Training min, max, SD, explained variance: -5.6236, 6.17, 2.327, 0.0505.
2. OPS PC12 out of range. Value: -4.4307. Training min, max, SD, explained variance: -3.9196, 6.4101, 1.581, 0.0233.

Feature Contribution

Top features for positive contribution

Fingerprint	Bit/Smiles	Feature Structure	Score	Carcinogen in training set
SCFP_6	-347048986	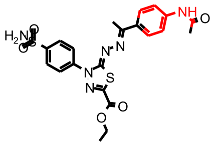 [*]C(=[*])N[c]1:[cH]:[cH]:[*]:[cH]:[cH]:1	0.615	5 out of 7

SCFP_6	814408713	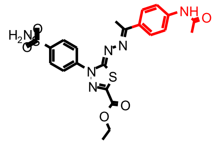 [*][c]1:[cH]:[cH]:[c](NC(=O)C):[cH]:[cH]:1	0.603	2 out of 2
SCFP_6	1626825020	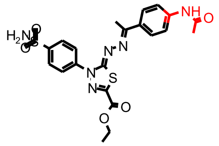 [*]:[c](:[*])NC(=O)C	0.561	3 out of 4

Top Features for negative contribution

Fingerprint	Bit/Smiles	Feature Structure	Score	Carcinogen in training set
SCFP_6	-1358544872	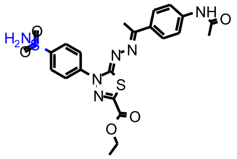 [*]S(=[*])(=[*])N	-0.484	1 out of 7
SCFP_6	798581009	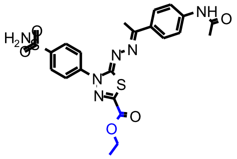 [*]C(=[*])OCC	-0.412	2 out of 11
SCFP_6	-1463646519	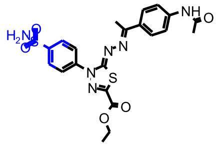 [*]:[cH]:[c](:[cH]:[*])S(=O)(=O)N	-0.38	1 out of 6

Erlotinib

TOPKAT_Rat_Male_FDA_None_vs_Carcinogen

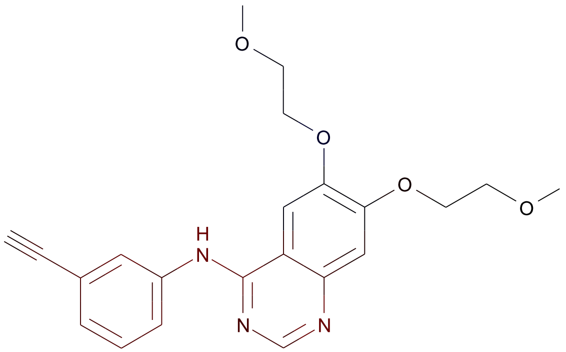

$C_{22}H_{23}N_3O_4$
Molecular Weight: 393.43572
ALogP: 4.309
Rotatable Bonds: 10
Acceptors: 7
Donors: 1

Model Prediction

Prediction: Carcinogen
Probability: 0.52
Enrichment: 1.56
Bayesian Score: 4.91
Mahalanobis Distance: 17.4
Mahalanobis Distance p-value: 2.91e-012

Prediction: Positive if the Bayesian score is above the estimated best cutoff value from minimizing the false positive and false negative rate.
Probability: The esimated probability that the sample is in the positive category. This assumes that the Bayesian score follows a normal distribution and is different from the prediction using a cutoff.
Enrichment: An estimate of enrichment, that is, the increased likelihood (versus random) of this sample being in the category.
Bayesian Score: The standard Laplacian-modified Bayesian score.
Mahalanobis Distance: The Mahalanobis distance (MD) is the distance to the center of the training data. The larger the MD, the less trustworthy the prediction.
Mahalanobis Distance p-value: The p-value gives the fraction of training data with an MD greater than or equal to the one for the given sample, assuming normally distributed data. The smaller the p-value, the less trustworthy the prediciton. For highly non-normal X properties (e.g., fingerprints), the MD p-value is wildly inaccurate.

Structural Similar Compounds

Name	Mycophenolate	Nicardipine	Nimodipine
Structure	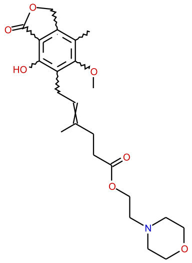	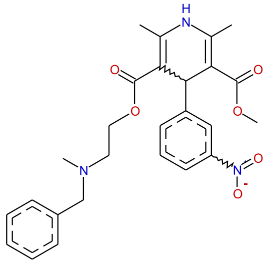	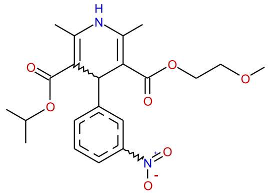
Actual Endpoint	Non-Carcinogen	Carcinogen	Carcinogen
Predicted Endpoint	Non-Carcinogen	Carcinogen	Carcinogen
Distance	0.624	0.667	0.676
Reference	US FDA (Centre for Drug Eval.& Res./Off. Testing & Res.) Sept. 1997	US FDA (Centre for Drug Eval.& Res./Off. Testing & Res.) Sept. 1997	US FDA (Centre for Drug Eval.& Res./Off. Testing & Res.) Sept. 1997

Model Applicability

Unknown features are fingerprint features in the query molecule, but not found or appearing too infrequently in the training set.

1. All properties and OPS components are within expected ranges.

Feature Contribution

Top features for positive contribution

Fingerprint	Bit/Smiles	Feature Structure	Score	Carcinogen in training set
SCFP_6	-1065373877	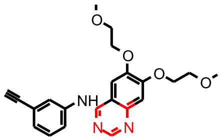 [*][c]1:[*]:[c](:[*]):n:[cH]:n:1	0.429	3 out of 5

SCFP_6	123285475	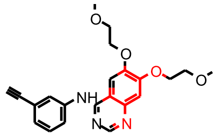 [*]O[c]1:[cH]:[c](:n:[*]):[c](:[*]):[*]:[c]:1[*]	0.429	3 out of 5
SCFP_6	-2147171373	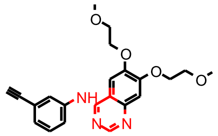 [*]N[c]1:n:[cH]:n:[*]:[c]:1:[*]	0.415	1 out of 1
**Top Features for negative contribution**				
**Fingerprint**	**Bit/Smiles**	**Feature Structure**	**Score**	**Carcinogen in training set**
SCFP_6	125474664	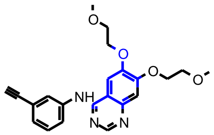 [*]O[c]1:[cH]:[c](:[c]([*]):[*]):[c](:[*]):[*]:[c]:1[*]	-0.484	1 out of 7
SCFP_6	-417738003	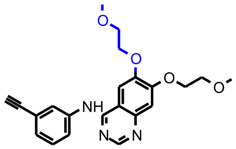 [*]OCCOC	-0.264	1 out of 5
SCFP_6	951581613	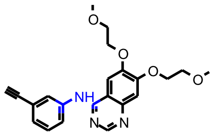 [*]:[c](:[*])N[c](:[*]):[*]	-0.132	1 out of 4

acetazolamide.cdx

TOPKAT_Rat_Male_FDA_None_vs_Carcinogen

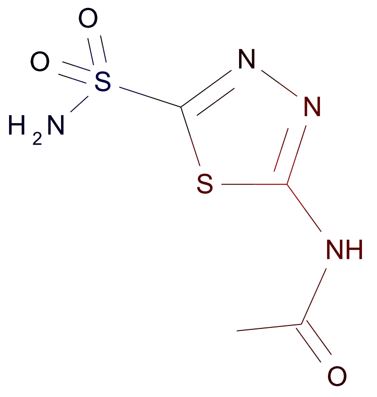

$C_4H_6N_4O_3S_2$
Molecular Weight: 222.24544
ALogP: -1.329
Rotatable Bonds: 2
Acceptors: 5
Donors: 2

Structural Similar Compounds

Name	Nitrofurazone	Niridazole	Furidazina
Structure	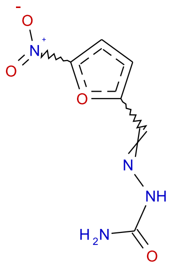	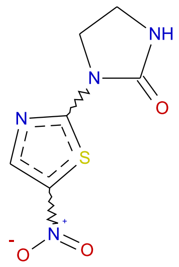	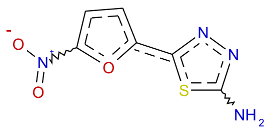
Actual Endpoint	Carcinogen	Carcinogen	Carcinogen
Predicted Endpoint	Carcinogen	Carcinogen	Carcinogen
Distance	0.689	0.700	0.707
Reference	US FDA (Centre for Drug Eval.& Res./Off. Testing & Res.) Sept. 1997	US FDA (Centre for Drug Eval.& Res./Off. Testing & Res.) Sept. 1997	US FDA (Centre for Drug Eval.& Res./Off. Testing & Res.) Sept. 1997

Model Prediction

Prediction: Carcinogen
Probability: 0.419
Enrichment: 1.25
Bayesian Score: 2.04
Mahalanobis Distance: 22.3
Mahalanobis Distance p-value: 7.97e-025

Prediction: Positive if the Bayesian score is above the estimated best cutoff value from minimizing the false positive and false negative rate.
Probability: The esimated probability that the sample is in the positive category. This assumes that the Bayesian score follows a normal distribution and is different from the prediction using a cutoff.
Enrichment: An estimate of enrichment, that is, the increased likelihood (versus random) of this sample being in the category.
Bayesian Score: The standard Laplacian-modified Bayesian score.
Mahalanobis Distance: The Mahalanobis distance (MD) is the distance to the center of the training data. The larger the MD, the less trustworthy the prediction.
Mahalanobis Distance p-value: The p-value gives the fraction of training data with an MD greater than or equal to the one for the given sample, assuming normally distributed data. The smaller the p-value, the less trustworthy the prediciton. For highly non-normal X properties (e.g., fingerprints), the MD p-value is wildly inaccurate.

Model Applicability

Unknown features are fingerprint features in the query molecule, but not found or appearing too infrequently in the training set.

1. All properties and OPS components are within expected ranges.

Feature Contribution

Top features for positive contribution

Fingerprint	Bit/Smiles	Feature Structure	Score	Carcinogen in training set
SCFP_6	-100019659	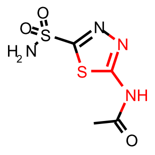 [*]N[c]1:n:[*]:[*]:s:1	0.603	2 out of 2

SCFP_6	1626825020	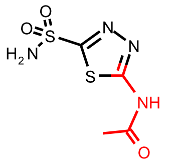 [*]:[c](:[*])NC(=O)C	0.561	3 out of 4
SCFP_6	149212520	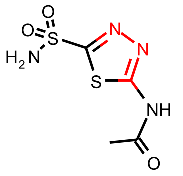 [*][c]1:[*]:[*]:n:n:1	0.543	9 out of 15
**Top Features for negative contribution**				
**Fingerprint**	**Bit/Smiles**	**Feature Structure**	**Score**	**Carcinogen in training set**
SCFP_6	-1358544872	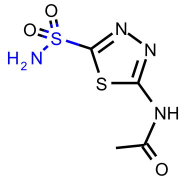 [*]S(=[*])(=[*])N	-0.484	1 out of 7
SCFP_6	21	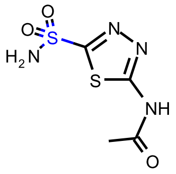 [*]S(=[*])(=[*])[*]	-0.283	7 out of 30
SCFP_6	1311429347	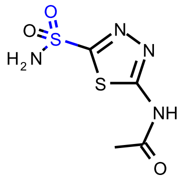 [*]S(=[*])(=O)[*]	-0.283	7 out of 30

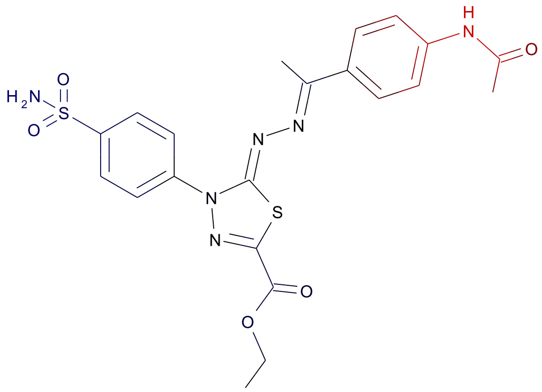

$C_{21}H_{22}N_6O_5S_2$
Molecular Weight: 502.56657
ALogP: 2.259
Rotatable Bonds: 8
Acceptors: 10
Donors: 2

Model Prediction

Prediction: Single-Carcinogen
Probability: 0.565
Enrichment: 1.36
Bayesian Score: 0.149
Mahalanobis Distance: 17.5
Mahalanobis Distance p-value: 5.2e-008

Prediction: Positive if the Bayesian score is above the estimated best cutoff value from minimizing the false positive and false negative rate.
Probability: The esimated probability that the sample is in the positive category. This assumes that the Bayesian score follows a normal distribution and is different from the prediction using a cutoff.
Enrichment: An estimate of enrichment, that is, the increased likelihood (versus random) of this sample being in the category.
Bayesian Score: The standard Laplacian-modified Bayesian score.
Mahalanobis Distance: The Mahalanobis distance (MD) is the distance to the center of the training data. The larger the MD, the less trustworthy the prediction.
Mahalanobis Distance p-value: The p-value gives the fraction of training data with an MD greater than or equal to the one for the given sample, assuming normally distributed data. The smaller the p-value, the less trustworthy the prediciton. For highly non-normal X properties (e.g., fingerprints), the MD p-value is wildly inaccurate.

Structural Similar Compounds

Name	Nimodipine	Sulfasalazine	Lasiocarpine
Structure	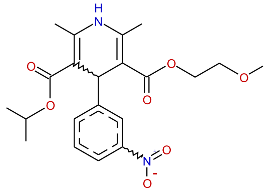	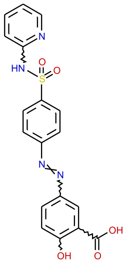	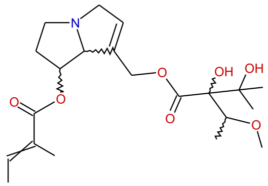
Actual Endpoint	Single-Carcinogen	Single-Carcinogen	Single-Carcinogen
Predicted Endpoint	Single-Carcinogen	Single-Carcinogen	Single-Carcinogen
Distance	0.806	0.819	0.824
Reference	US FDA (Centre for Drug Eval.& Res./Off. Testing & Res.) Sept. 1997	US FDA (Centre for Drug Eval.& Res./Off. Testing & Res.) Sept. 1997	US FDA (Centre for Drug Eval.& Res./Off. Testing & Res.) Sept. 1997

Model Applicability

Unknown features are fingerprint features in the query molecule, but not found or appearing too infrequently in the training set.

1. Num_H_Acceptors out of range. Value: 10. Training min, max, mean, SD: 0, 9, 3.8906, 2.196.
2. OPS PC5 out of range. Value: 6.3854. Training min, max, SD, explained variance: -4.8296, 6.2055, 2.09, 0.0593.

Feature Contribution

Top features for positive contribution

Fingerprint	Bit/Smiles	Feature Structure	Score	Multiple-Carcinogen in training set
SCFP_8	2097618059	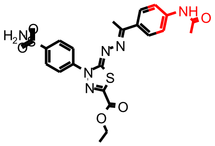 [*]:[cH]:[c](NC(=O)C):[cH]:[*]	0.681	6 out of 7

SCFP_8	-347048986	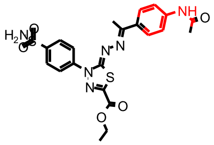<br>[*]C(=[*])N[c]1:[cH]:<br>[cH]:[*]:[cH]:[cH]:1	0.574	4 out of 5
SCFP_8	814408713	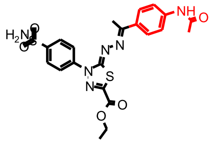<br>[*][c]1:[cH]:[cH]:[c]<br>(NC(=O)C):[cH]:[cH]:<br>1	0.553	2 out of 2

Top Features for negative contribution

**Fingerprint**	**Bit/Smiles**	**Feature Structure**	**Score**	**Multiple-Carcinogen in training set**
SCFP_8	-1247518081	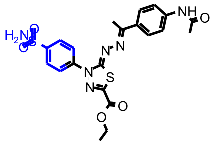<br>NS(=O)(=O)[c]1:[cH]:[<br>cH]:[*]:[cH]:[cH]:1	-0.737	0 out of 3
SCFP_8	1132907712	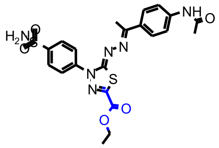<br>[*]OC(=O)C(=[*])[*]	-0.58	2 out of 12
SCFP_8	1892918731	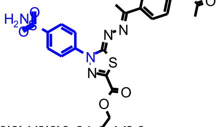<br>[*]N([*])[c]1:[cH]:[c<br>H]:[c](:[cH]:[cH]:1)<br>S(=O)(=O)N	-0.546	0 out of 2

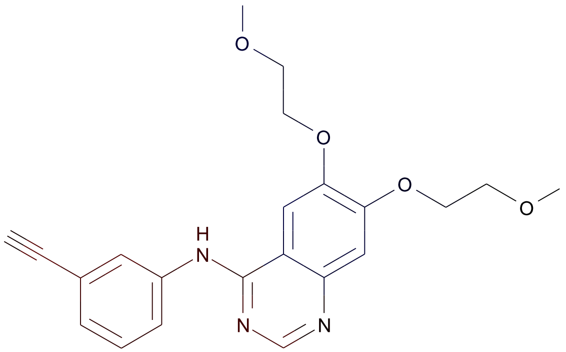

$C_{22}H_{23}N_3O_4$
Molecular Weight: 393.43572
ALogP: 4.309
Rotatable Bonds: 10
Acceptors: 7
Donors: 1

Model Prediction

Prediction: Multiple-Carcinogen
Probability: 0.568
Enrichment: 1.37
Bayesian Score: 0.821
Mahalanobis Distance: 16.5
Mahalanobis Distance p-value: 4.64e-007

Prediction: Positive if the Bayesian score is above the estimated best cutoff value from minimizing the false positive and false negative rate.
Probability: The esimated probability that the sample is in the positive category. This assumes that the Bayesian score follows a normal distribution and is different from the prediction using a cutoff.
Enrichment: An estimate of enrichment, that is, the increased likelihood (versus random) of this sample being in the category.
Bayesian Score: The standard Laplacian-modified Bayesian score.
Mahalanobis Distance: The Mahalanobis distance (MD) is the distance to the center of the training data. The larger the MD, the less trustworthy the prediction.
Mahalanobis Distance p-value: The p-value gives the fraction of training data with an MD greater than or equal to the one for the given sample, assuming normally distributed data. The smaller the p-value, the less trustworthy the prediciton. For highly non-normal X properties (e.g., fingerprints), the MD p-value is wildly inaccurate.

Structural Similar Compounds

Name	Nicardipine	Nimodipine	Felodipine
Structure	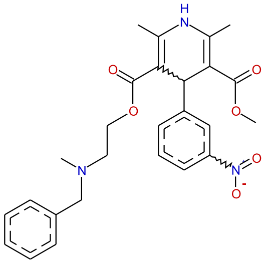	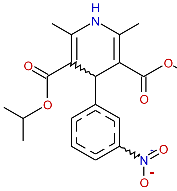	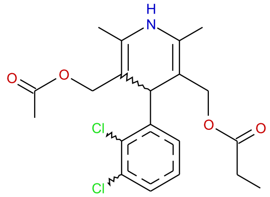
Actual Endpoint	Single-Carcinogen	Single-Carcinogen	Single-Carcinogen
Predicted Endpoint	Single-Carcinogen	Single-Carcinogen	Single-Carcinogen
Distance	0.685	0.700	0.721
Reference	US FDA (Centre for Drug Eval.& Res./Off. Testing & Res.) Sept. 1997	US FDA (Centre for Drug Eval.& Res./Off. Testing & Res.) Sept. 1997	US FDA (Centre for Drug Eval.& Res./Off. Testing & Res.) Sept. 1997

Model Applicability

Unknown features are fingerprint features in the query molecule, but not found or appearing too infrequently in the training set.

1. All properties and OPS components are within expected ranges.

Feature Contribution

Top features for positive contribution

Fingerprint	Bit/Smiles	Feature Structure	Score	Multiple-Carcinogen in training set
SCFP_8	-1065373877	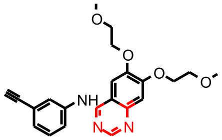 [*][c]1:[*]:[c](:[*]):n:[cH]:n:1	0.649	3 out of 3

SCFP_8	2	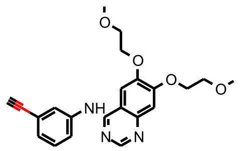 [*]C#[*]	0.584	6 out of 8
SCFP_8	-1181430618	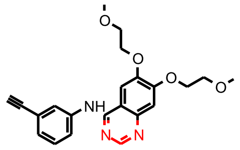 [*]:n:[cH]:n:[*]	0.453	4 out of 6
**Top Features for negative contribution**				
**Fingerprint**	**Bit/Smiles**	**Feature Structure**	**Score**	**Multiple-Carcinogen in training set**
SCFP_8	136239834	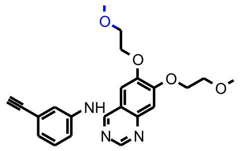 [*]OC	-0.358	3 out of 13
SCFP_8	129215346	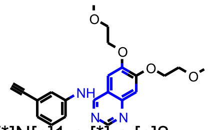 [*]N[c]1:n:[*]:n:[c]2:[cH]:[*]:[c]([*]):[cH]:[c]:1:2	-0.31	0 out of 1
SCFP_8	125474664	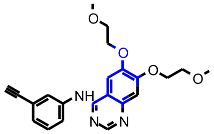 [*]O[c]1:[cH]:[c](:[c]([*]):[*]):[c](:[*]):[*]:[c]:1[*]	-0.31	0 out of 1

acetazolamide.cdx

TOPKAT_Rat_Male_FDA_Single_vs_Multiple

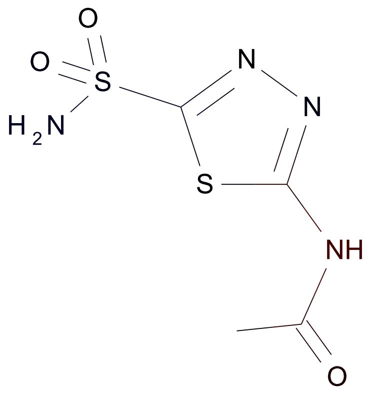

$C_4H_6N_4O_3S_2$
Molecular Weight: 222.24544
ALogP: -1.329
Rotatable Bonds: 2
Acceptors: 5
Donors: 2

Structural Similar Compounds

Name	Nitrofurazone	Niridazole	Azaserine
Structure	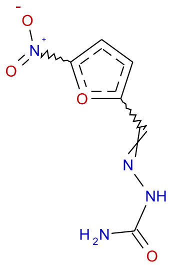	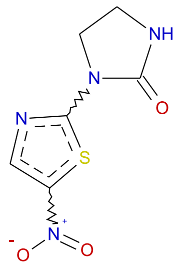	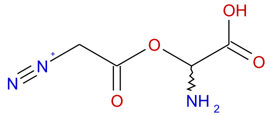
Actual Endpoint	Multiple-Carcinogen	Multiple-Carcinogen	Single-Carcinogen
Predicted Endpoint	Multiple-Carcinogen	Multiple-Carcinogen	Single-Carcinogen
Distance	0.700	0.730	0.748
Reference	US FDA (Centre for Drug Eval.& Res./Off. Testing & Res.) Sept. 1997	US FDA (Centre for Drug Eval.& Res./Off. Testing & Res.) Sept. 1997	US FDA (Centre for Drug Eval.& Res./Off. Testing & Res.) Sept. 1997

Model Prediction

Prediction: Multiple-Carcinogen
Probability: 0.569
Enrichment: 1.37
Bayesian Score: 1.44
Mahalanobis Distance: 10.5
Mahalanobis Distance p-value: 0.052

Prediction: Positive if the Bayesian score is above the estimated best cutoff value from minimizing the false positive and false negative rate.
Probability: The esimated probability that the sample is in the positive category. This assumes that the Bayesian score follows a normal distribution and is different from the prediction using a cutoff.
Enrichment: An estimate of enrichment, that is, the increased likelihood (versus random) of this sample being in the category.
Bayesian Score: The standard Laplacian-modified Bayesian score.
Mahalanobis Distance: The Mahalanobis distance (MD) is the distance to the center of the training data. The larger the MD, the less trustworthy the prediction.
Mahalanobis Distance p-value: The p-value gives the fraction of training data with an MD greater than or equal to the one for the given sample, assuming normally distributed data. The smaller the p-value, the less trustworthy the prediciton. For highly non-normal X properties (e.g., fingerprints), the MD p-value is wildly inaccurate.

Model Applicability

Unknown features are fingerprint features in the query molecule, but not found or appearing too infrequently in the training set.

1. OPS PC20 out of range. Value: 3.0465. Training min, max, SD, explained variance: -2.6601, 2.9766, 1.03, 0.0144.

Feature Contribution

Top features for positive contribution

Fingerprint	Bit/Smiles	Feature Structure	Score	Multiple-Carcinogen in training set
SCFP_8	1631845520	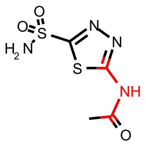 [*]C(=[*])N[c](:[*]):[*]	0.495	6 out of 9

SCFP_8	1626825020	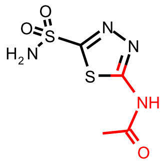 [*]:[c](:[*])NC(=O)C	0.362	2 out of 3
SCFP_8	1256995004	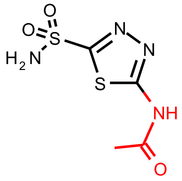 [*]NC(=O)C	0.331	10 out of 19
**Top Features for negative contribution**				
**Fingerprint**	**Bit/Smiles**	**Feature Structure**	**Score**	**Multiple-Carcinogen in training set**
SCFP_8	136627117	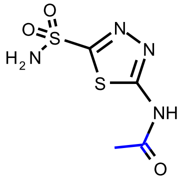 [*]C(=[*])C	-0.41	4 out of 18
SCFP_8	182991870	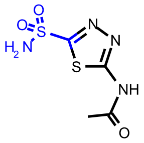 [*]:[c](:[*])S(=O)(=O)N	-0.342	1 out of 5
SCFP_8	-1607191420	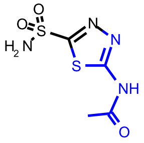 CC(=O)N[c]1:n:[*]:[*] :s:1	-0.31	0 out of 1

Comp. 14

TOPKAT_Skin_Irritancy_None_vs_Irritant

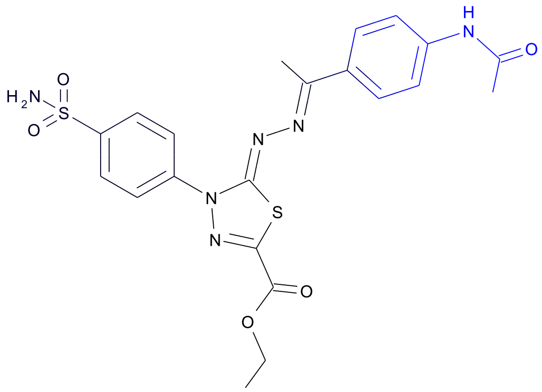

$C_{21}H_{22}N_6O_5S_2$

Molecular Weight: 502.56657

ALogP: 2.259

Rotatable Bonds: 8

Acceptors: 10

Donors: 2

Structural Similar Compounds

Name	Benzenesulfonic acid, 2,2'-(1,4-anthraquinonylenediimino)bis(5-meth yl-, disodium salt	2,2'-Stilbenedisulfonic acid, 4,4'-dinitro-	2-Anthracenesulfonic acid, 1-amino-9,10-dihydro-9,10-dioxo-4-(2,4,6 -trimethylanilino)-, monosodium salt
Structure	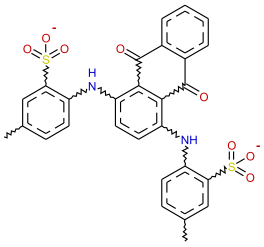	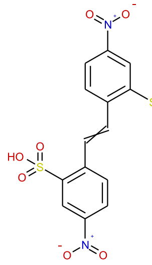	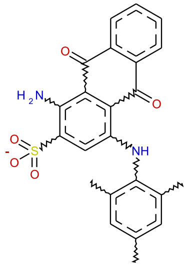
Actual Endpoint	Irritant	Irritant	Irritant
Predicted Endpoint	Non-Irritant	Irritant	Non-Irritant
Distance	0.678	0.837	0.958
Reference	85JCAE "Prehled Prumyslove Toxikologie; Organicke Latky," Marhold, J., Prague , Czechoslovakia, Avicenum, 1986 Volume(issue)/page/year: -,1330,1986	85JCAE "Prehled Prumyslove Toxikologie; Organicke Latky," Marhold, J., Prague , Czechoslovakia, Avicenum, 1986 Volume(issue)/page/year: -,1062,1986	85JCAE "Prehled Prumyslove Toxikologie; Organicke Latky," Marhold, J., Prague , Czechoslovakia, Avicenum, 1986 Volume(issue)/page/year: -,1327,1986

Model Prediction

Prediction: Non-Irritant

Probability: 2.36e-006

Enrichment: 2.56e-006

Bayesian Score: -10.3

Mahalanobis Distance: 9.98

Mahalanobis Distance p-value: 0.0665

Prediction: Positive if the Bayesian score is above the estimated best cutoff value from minimizing the false positive and false negative rate.
Probability: The esimated probability that the sample is in the positive category. This assumes that the Bayesian score follows a normal distribution and is different from the prediction using a cutoff.
Enrichment: An estimate of enrichment, that is, the increased likelihood (versus random) of this sample being in the category.
Bayesian Score: The standard Laplacian-modified Bayesian score.
Mahalanobis Distance: The Mahalanobis distance (MD) is the distance to the center of the training data. The larger the MD, the less trustworthy the prediction.
Mahalanobis Distance p-value: The p-value gives the fraction of training data with an MD greater than or equal to the one for the given sample, assuming normally distributed data. The smaller the p-value, the less trustworthy the prediciton. For highly non-normal X properties (e.g., fingerprints), the MD p-value is wildly inaccurate.

Model Applicability

Unknown features are fingerprint features in the query molecule, but not found or appearing too infrequently in the training set.

1. All properties and OPS components are within expected ranges.

Feature Contribution

Top features for positive contribution				
**Fingerprint**	**Bit/Smiles**	**Feature Structure**	**Score**	**Irritant in training set**

FCFP_12	-1986158408	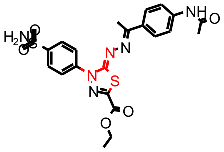 [*]\N=C\1/S[*]=[*]N1[*]	0.0821	13 out of 13
FCFP_12	-432846198	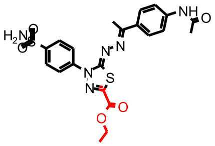 [*]C(=[*])C(=O)OCC	0.0697	160 out of 163
FCFP_12	580960234	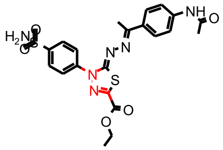 [*]N1[*][*]C(=N1)[*]	0.0583	2 out of 2

Top Features for negative contribution

Fingerprint	Bit/Smiles	Feature Structure	Score	Irritant in training set
FCFP_12	-1944671191	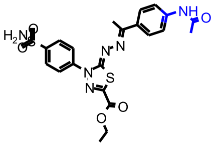 [*]:[c](:[*])NC(=O)C	-1.87	0 out of 6
FCFP_12	1907952166	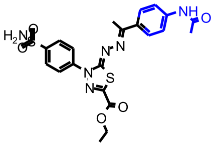 CC(=O)N[c]1:[cH]:[cH]:[*]:[cH]:[cH]:1	-1.72	0 out of 5

FCFP_12	1175665944	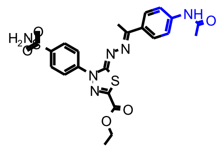 [*]:[cH]:[c](NC(=O)C):[cH]:[*]	-1.02	2 out of 8

Erlotinib

TOPKAT_Skin_Irritancy_None_vs_Irritant

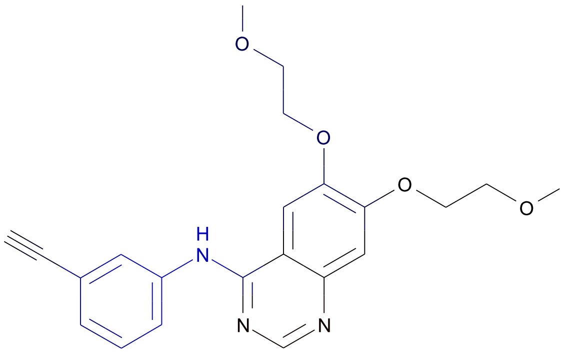

$C_{22}H_{23}N_3O_4$

Molecular Weight: 393.43572

ALogP: 4.309

Rotatable Bonds: 10

Acceptors: 7

Donors: 1

Model Prediction

Prediction: Non-Irritant

Probability: 0.119

Enrichment: 0.13

Bayesian Score: -5.76

Mahalanobis Distance: 10.2

Mahalanobis Distance p-value: 0.0333

Prediction: Positive if the Bayesian score is above the estimated best cutoff value from minimizing the false positive and false negative rate.
Probability: The esimated probability that the sample is in the positive category. This assumes that the Bayesian score follows a normal distribution and is different from the prediction using a cutoff.
Enrichment: An estimate of enrichment, that is, the increased likelihood (versus random) of this sample being in the category.
Bayesian Score: The standard Laplacian-modified Bayesian score.
Mahalanobis Distance: The Mahalanobis distance (MD) is the distance to the center of the training data. The larger the MD, the less trustworthy the prediction.
Mahalanobis Distance p-value: The p-value gives the fraction of training data with an MD greater than or equal to the one for the given sample, assuming normally distributed data. The smaller the p-value, the less trustworthy the prediciton. For highly non-normal X properties (e.g., fingerprints), the MD p-value is wildly inaccurate.

Structural Similar Compounds

Name	Propanoic acid, 2-(4-((5-(trifluoromethyl)-2-pyridinyl)oxy)phenoxy) -, butyl ester	Carbamic acid, ((dibutylamino)thio)methyl-, 2,2-dimethyl-2,3-dihydro-7-benzofuranyl ester	1,4-Pentadien-3-one, 1,5-bis(p-azidophenyl)-
Structure	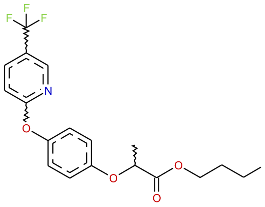	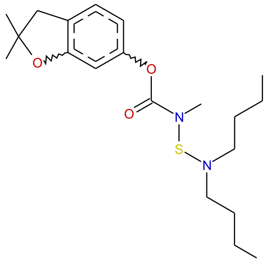	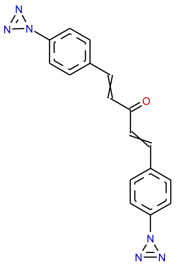
Actual Endpoint	Irritant	Irritant	Irritant
Predicted Endpoint	Irritant	Irritant	Irritant
Distance	0.776	0.777	0.803
Reference	NNGADV Nippon Noyaku Gakkaishi. Journal of the Pesticide Science Society of Japan. (Nippon Noyaku Gakkai, 1-43-11, Komagome, Toshima-ku, Tokyo 170, Japan) V.1-1976- Volume(issue)/page/year: 15,305,1990	NTIS** National Technical Information Service. (Springfield, VA 22161) Forme rly U.S. Clearinghouse for Scientific & Technical Information. Volume(issue)/pag e/year: OTS0539690	85JCAE "Prehled Prumyslove Toxikologie; Organicke Latky," Marhold, J., Prague , Czechoslovakia, Avicenum, 1986 Volume(issue)/page/year: -,733,1986

Model Applicability

Unknown features are fingerprint features in the query molecule, but not found or appearing too infrequently in the training set.

1. All properties and OPS components are within expected ranges.
2. Unknown FCFP_2 feature: 902193919: [*]:[c](:[*])C#C

Feature Contribution

Top features for positive contribution				
Fingerprint	Bit/Smiles	Feature Structure	Score	Irritant in training set

FCFP_12	-124685461	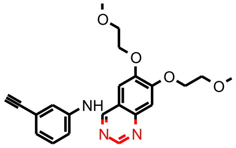 [*]:n:[cH]:n:[*]	0.0734	5 out of 5
FCFP_12	-475316933	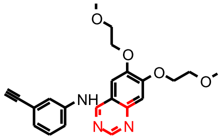 [*][c]1:[*]:[c](:[*]):n:[cH]:n:1	0.0703	4 out of 4
FCFP_12	131784192	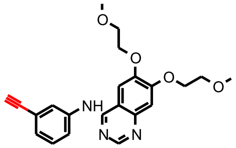 [*]C#C	0.0658	3 out of 3
**Top Features for negative contribution**				
**Fingerprint**	**Bit/Smiles**	**Feature Structure**	**Score**	**Irritant in training set**
FCFP_12	411414971	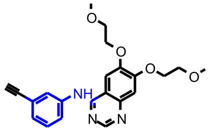 [*][c]1:[cH]:[cH]:[cH]:[c](N[c](:[*]):[*]):[cH]:1	-1.31	1 out of 7
FCFP_12	-1059904848	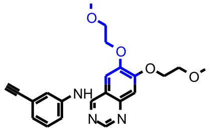 [*][c](:[*]):[c](OCCOC):[cH]:[*]	-1.04	0 out of 2

FCFP_12	839741273	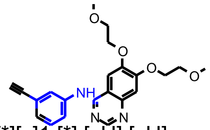 [*][c]1:[*]:[cH]:[cH]:[c](N[c](:[*]):[*]):[cH]:1	-0.708	4 out of 10

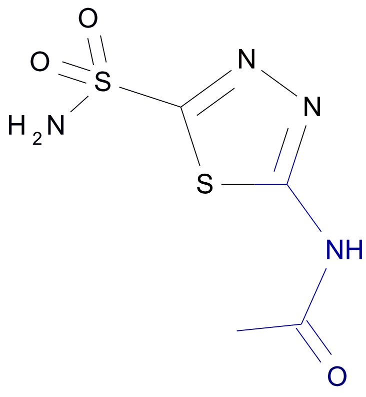

$C_4H_6N_4O_3S_2$
Molecular Weight: 222.24544
ALogP: -1.329
Rotatable Bonds: 2
Acceptors: 5
Donors: 2

Model Prediction

Prediction: Non-Irritant
Probability: 0.743
Enrichment: 0.807
Bayesian Score: -3.77
Mahalanobis Distance: 7.13
Mahalanobis Distance p-value: 0.993

Prediction: Positive if the Bayesian score is above the estimated best cutoff value from minimizing the false positive and false negative rate.
Probability: The esimated probability that the sample is in the positive category. This assumes that the Bayesian score follows a normal distribution and is different from the prediction using a cutoff.
Enrichment: An estimate of enrichment, that is, the increased likelihood (versus random) of this sample being in the category.
Bayesian Score: The standard Laplacian-modified Bayesian score.
Mahalanobis Distance: The Mahalanobis distance (MD) is the distance to the center of the training data. The larger the MD, the less trustworthy the prediction.
Mahalanobis Distance p-value: The p-value gives the fraction of training data with an MD greater than or equal to the one for the given sample, assuming normally distributed data. The smaller the p-value, the less trustworthy the prediciton. For highly non-normal X properties (e.g., fingerprints), the MD p-value is wildly inaccurate.

Structural Similar Compounds

Name	p-Benzenedisulfonic acid, 2-amino-	Hydrazine, 2,4-dinitrophenyl-	Benzenesulfonic acid, 3-amino-4-hydroxy-5-nitro-
Structure	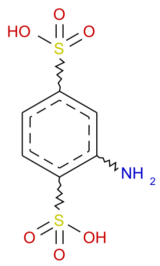	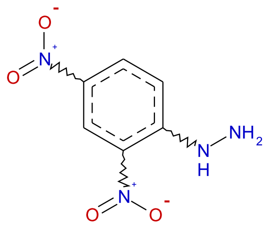	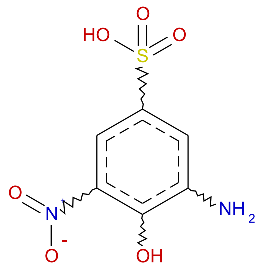
Actual Endpoint	Irritant	Non-Irritant	Irritant
Predicted Endpoint	Non-Irritant	Non-Irritant	Non-Irritant
Distance	0.730	0.734	0.745
Reference	85JCAE "Prehled Prumyslove Toxikologie; Organicke Latky," Marhold, J., Prague , Czechoslovakia, Avicenum, 1986 Volume(issue)/page/year: -,1052,1986	28ZPAK -,132,72	85JCAE "Prehled Prumyslove Toxikologie; Organicke Latky," Marhold, J., Prague , Czechoslovakia, Avicenum, 1986 Volume(issue)/page/year: -,1053,1986

Model Applicability

Unknown features are fingerprint features in the query molecule, but not found or appearing too infrequently in the training set.

1. All properties and OPS components are within expected ranges.

Feature Contribution

Top features for positive contribution				
**Fingerprint**	**Bit/Smiles**	**Feature Structure**	**Score**	**Irritant in training set**
FCFP_12	4427049	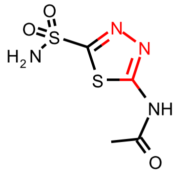 [*][c]1:[*]:[*]:n:n:1	0.0734	5 out of 5

FCFP_12	-1539162406	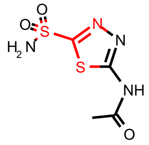 [*]S(=[*])(=[*])[c]1:n:[*]:[*]:s:1	0.0583	2 out of 2

Top Features for negative contribution

**Fingerprint**	**Bit/Smiles**	**Feature Structure**	**Score**	**Irritant in training set**
FCFP_12	-1944671191	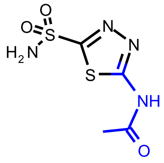 [*]:[c](:[*])NC(=O)C	-1.87	0 out of 6
FCFP_12	1294255210	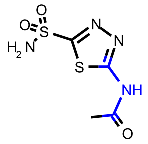 [*]C(=[*])N[c](:[*]):[*]	-0.486	12 out of 22
FCFP_12	566058135	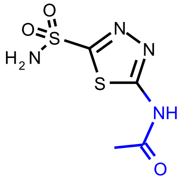 [*]NC(=O)C	-0.367	13 out of 21

Comp. 14

TOPKAT_Carcinogenic_Potency_TD50_Mouse

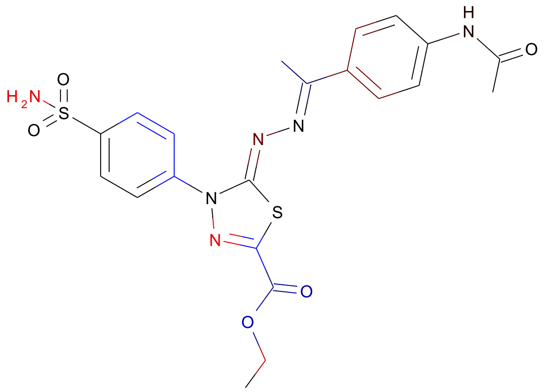

$C_{21}H_{22}N_6O_5S_2$
Molecular Weight: 502.56657
ALogP: 2.259
Rotatable Bonds: 8
Acceptors: 10
Donors: 2

Structural Similar Compounds

Name	Salicylazosulfapyridine	420	D & C red no. 5
Structure	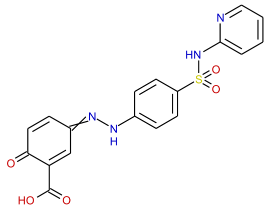	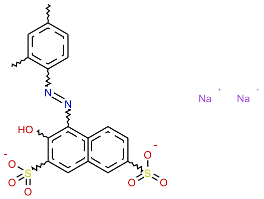	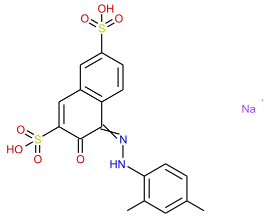
Actual Endpoint (-log C)	2.5034	2.78302	2.80732
Predicted Endpoint (-log C)	3.54214	3.31546	3.78615
Distance	0.730	0.799	0.839
Reference	CPDB	CPDB	CPDB

Model Prediction

Prediction: 14.2
Unit: mg/kg_body_weight/day
Mahalanobis Distance: 13.5
Mahalanobis Distance p-value: 5.19e-009

Mahalanobis Distance: The Mahalanobis distance (MD) is a generalization of the Euclidean distance that accounts for correlations among the X properties. It is calculated as the distance to the center of the training data. The larger the MD, the less trustworthy the prediction.
Mahalanobis Distance p-value: The p-value gives the fraction of training data with an MD greater than or equal to the one for the given sample, assuming normally distributed data. The smaller the p-value, the less trustworthy the prediciton. For highly non-normal X properties (e.g., fingerprints), the MD p-value is wildly inaccurate.

Model Applicability

Unknown features are fingerprint features in the query molecule, but not found or appearing too infrequently in the training set.

1. All properties and OPS components are within expected ranges.
2. Unknown ECFP_2 feature: -934225701: [*]C(=[*])C1=N[*][*]S1
3. Unknown ECFP_2 feature: -1110911409: [*]=C1[*][*]=NN1[c](:[*]):[*]
4. Unknown ECFP_2 feature: 189949281: [*]\N=C\1/S[*]=[*]N1[*]
5. Unknown ECFP_2 feature: -819426257: [*]C(=NN=[*])[*]
6. Unknown ECFP_2 feature: 562081661: [*]C(=NN=[*])[*]
7. Unknown ECFP_2 feature: 128986386: [*]\N=C(/C)\[c](:[*]):[*]

Feature Contribution

Top features for positive contribution			
**Fingerprint**	**Bit/Smiles**	**Feature Structure**	**Score**

ECFP_6	655739385	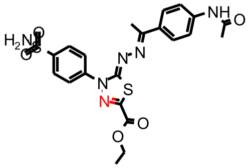 [*]N=[*]	0.229
ECFP_6	1572579716	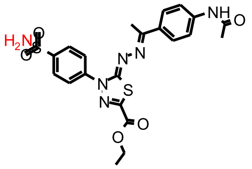 [*]N	0.225
ECFP_6	1559650422	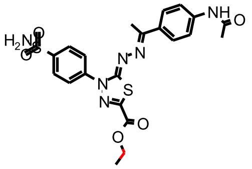 [*]C[*]	0.203
**Top Features for negative contribution**			
**Fingerprint**	**Bit/Smiles**	**Feature Structure**	**Score**
ECFP_6	1996767644	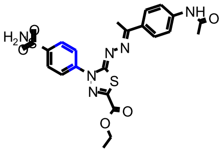 [*][c](:[*]):[cH]:[cH]:[*]	-0.251
ECFP_6	642810091	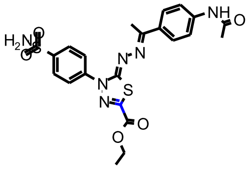 [*]C(=[*])[*]	-0.247

ECFP_6	-182236392	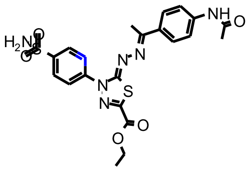 [*]:[cH]:[*]	-0.232

Erlotinib

TOPKAT_Carcinogenic_Potency_TD50_Mouse

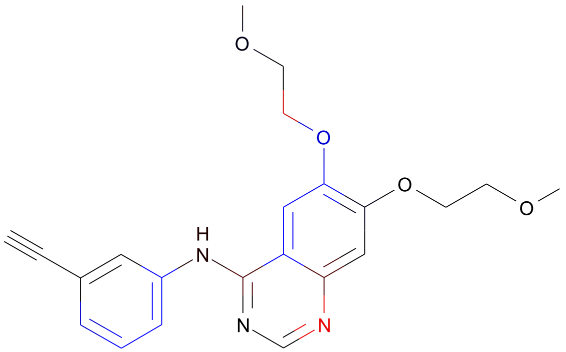

$C_{22}H_{23}N_3O_4$

Molecular Weight: 393.43572

ALogP: 4.309

Rotatable Bonds: 10

Acceptors: 7

Donors: 1

Model Prediction

Prediction: 39.8

Unit: mg/kg_body_weight/day

Mahalanobis Distance: 16.1

Mahalanobis Distance p-value: 1.15e-016

Mahalanobis Distance: The Mahalanobis distance (MD) is a generalization of the Euclidean distance that accounts for correlations among the X properties. It is calculated as the distance to the center of the training data. The larger the MD, the less trustworthy the prediction.

Mahalanobis Distance p-value: The p-value gives the fraction of training data with an MD greater than or equal to the one for the given sample, assuming normally distributed data. The smaller the p-value, the less trustworthy the prediciton. For highly non-normal X properties (e.g., fingerprints), the MD p-value is wildly inaccurate.

Structural Similar Compounds

Name	Compound LY171883	832	5,5´-(1,1´-Biphenyl)-2,5-dyl-bis (oxy)(2,2-dimethylpentanoic acid)
Structure	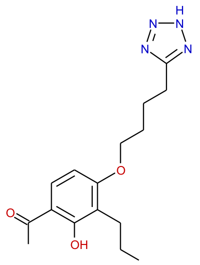	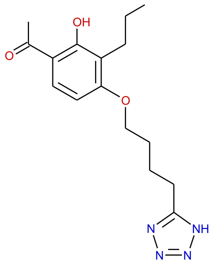	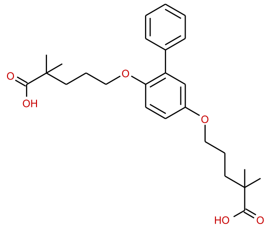
Actual Endpoint (-log C)	3.45372	3.45372	3.90166
Predicted Endpoint (-log C)	2.84749	2.80429	2.75893
Distance	0.772	0.782	0.796
Reference	CPDB	CPDB	CPDB

Model Applicability

Unknown features are fingerprint features in the query molecule, but not found or appearing too infrequently in the training set.

1. OPS PC13 out of range. Value: -3.2209. Training min, max, SD, explained variance: -3.068, 3.6909, 1.329, 0.0220.
2. Unknown ECFP_2 feature: -1253653003: [*]COC
3. Unknown ECFP_2 feature: -182178874: [*]#C[c](:c:[*]):c:[*]
4. Unknown ECFP_2 feature: 1139738044: [*]:[c](:[*])C#C

Feature Contribution

Top features for positive contribution			
**Fingerprint**	**Bit/Smiles**	**Feature Structure**	**Score**
ECFP_6	655739385	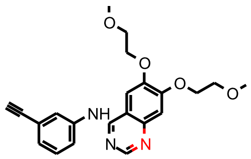 [*]N=[*]	0.229

ECFP_6	1559650422	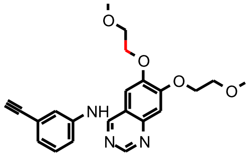 [*]C[*]	0.203
ECFP_6	1333660716	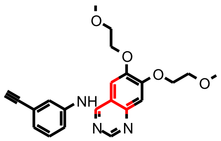 [*][c](:[*]):[c](:[cH]:[*]):[c](:[*]):[*]	0.0746
**Top Features for negative contribution**			
**Fingerprint**	**Bit/Smiles**	**Feature Structure**	**Score**
ECFP_6	1996767644	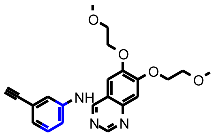 [*][c](:[*]):[cH]:[cH]:[*]	-0.251
ECFP_6	642810091	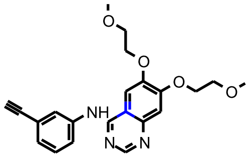 [*]C(=[*])[*]	-0.247
ECFP_6	-182236392	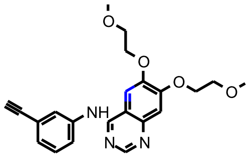 [*]:[cH]:[*]	-0.232

acetazolamide.cdx

TOPKAT_Carcinogenic_Potency_TD50_Mouse

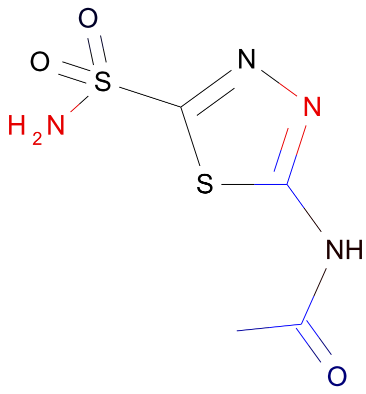

$C_4H_6N_4O_3S_2$

Molecular Weight: 222.24544

ALogP: -1.329

Rotatable Bonds: 2

Acceptors: 5

Donors: 2

Model Prediction

Prediction: 64.8

Unit: mg/kg_body_weight/day

Mahalanobis Distance: 11.7

Mahalanobis Distance p-value: 9.8e-005

Mahalanobis Distance: The Mahalanobis distance (MD) is a generalization of the Euclidean distance that accounts for correlations among the X properties. It is calculated as the distance to the center of the training data. The larger the MD, the less trustworthy the prediction.
Mahalanobis Distance p-value: The p-value gives the fraction of training data with an MD greater than or equal to the one for the given sample, assuming normally distributed data. The smaller the p-value, the less trustworthy the prediciton. For highly non-normal X properties (e.g., fingerprints), the MD p-value is wildly inaccurate.

Structural Similar Compounds

Name	Nithiazide	N-[5-(5-Nitro-2-furyl)-1,3,4-thia-di-azol-2-yl]acetamide	274
Structure	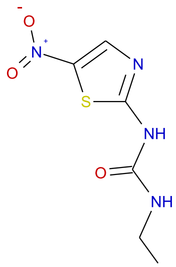	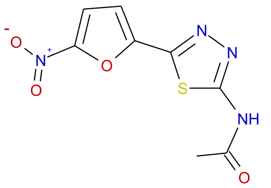	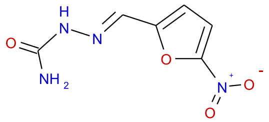
Actual Endpoint (-log C)	2.45522	4.57655	3.80841
Predicted Endpoint (-log C)	3.63942	3.40661	3.56025
Distance	0.600	0.635	0.636
Reference	CPDB	CPDB	CPDB

Model Applicability

Unknown features are fingerprint features in the query molecule, but not found or appearing too infrequently in the training set.

1. All properties and OPS components are within expected ranges.
2. Unknown ECFP_2 feature: 1221843808: [*]S(=[*])(=[*])[c]1:n:[*]:[*]:s:1

Feature Contribution

Top features for positive contribution			
**Fingerprint**	**Bit/Smiles**	**Feature Structure**	**Score**
ECFP_6	655739385	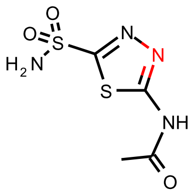 [*]N=[*]	0.229

ECFP_6	1572579716	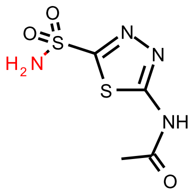 [*]N	0.225
ECFP_6	734603939	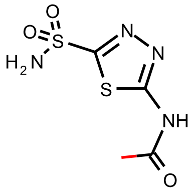 [*]C	0.0424
**Top Features for negative contribution**			
**Fingerprint**	**Bit/Smiles**	**Feature Structure**	**Score**
ECFP_6	642810091	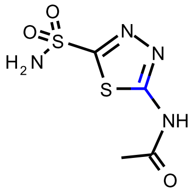 [*]C(=[*])[*]	-0.247
ECFP_6	866218936	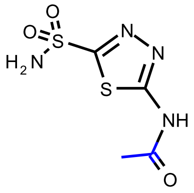 [*]C(=[*])C	-0.197
ECFP_6	2099970318	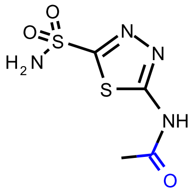 [*]C(=O)[*]	-0.118

Comp. 14

TOPKAT_Carcinogenic_Potency_TD50_Rat

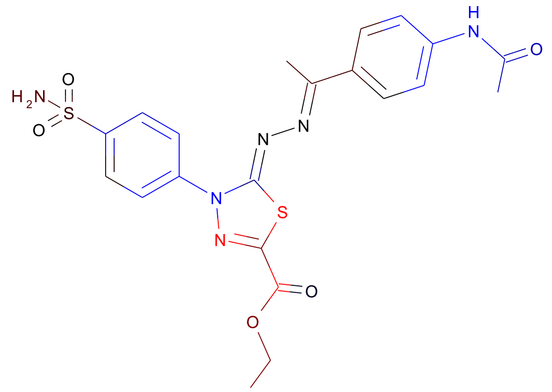

$C_{21}H_{22}N_6O_5S_2$

Molecular Weight: 502.56657

ALogP: 2.259

Rotatable Bonds: 8

Acceptors: 10

Donors: 2

Structural Similar Compounds

Name	623	Salicylazosulfapyridine	418
Structure	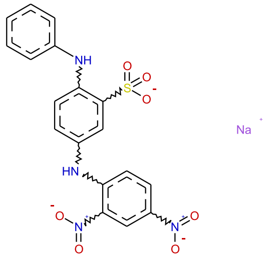	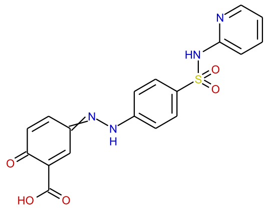	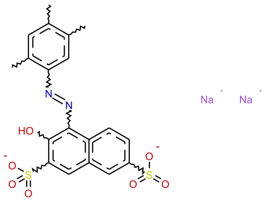
Actual Endpoint (-log C)	2.39985	2.39891	2.9349
Predicted Endpoint (-log C)	3.4177	3.17598	3.45907
Distance	0.641	0.714	0.763
Reference	CPDB	CPDB	CPDB

Model Prediction

Prediction: 36.9

Unit: mg/kg_body_weight/day

Mahalanobis Distance: 12.9

Mahalanobis Distance p-value: 7.31e-006

Mahalanobis Distance: The Mahalanobis distance (MD) is a generalization of the Euclidean distance that accounts for correlations among the X properties. It is calculated as the distance to the center of the training data. The larger the MD, the less trustworthy the prediction.
Mahalanobis Distance p-value: The p-value gives the fraction of training data with an MD greater than or equal to the one for the given sample, assuming normally distributed data. The smaller the p-value, the less trustworthy the prediciton. For highly non-normal X properties (e.g., fingerprints), the MD p-value is wildly inaccurate.

Model Applicability

Unknown features are fingerprint features in the query molecule, but not found or appearing too infrequently in the training set.

1. All properties and OPS components are within expected ranges.

Feature Contribution

Top features for positive contribution

Fingerprint	Bit/Smiles	Feature Structure	Score
FCFP_6	565998553	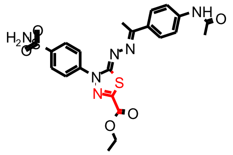 [*]C(=[*])C1=N[*][*]S 1	0.357

FCFP_6	1	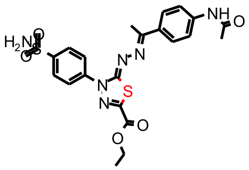<br>[*]S[*]	0.234
FCFP_6	203677720	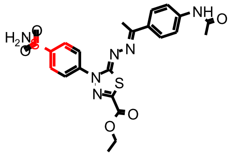<br>[*]S(=[*])(=[*])[c](:[cH]:[*]):[cH]:[*]	0.137
**Top Features for negative contribution**			
**Fingerprint**	**Bit/Smiles**	**Feature Structure**	**Score**
FCFP_6	16	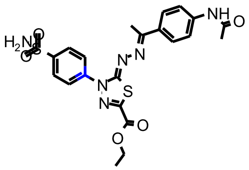<br>[*][c](:[*]):[*]	-0.354
FCFP_6	590925877	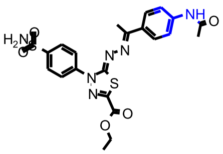<br>[*]N[c](:[cH]:[*]):[cH]:[*]	-0.323
FCFP_6	1674451008	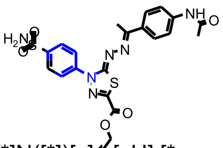<br>[*]N([*])[c]1:[cH]:[*]:[c]([*]):[cH]:[cH]:1	-0.233

Erlotinib

TOPKAT_Carcinogenic_Potency_TD50_Rat

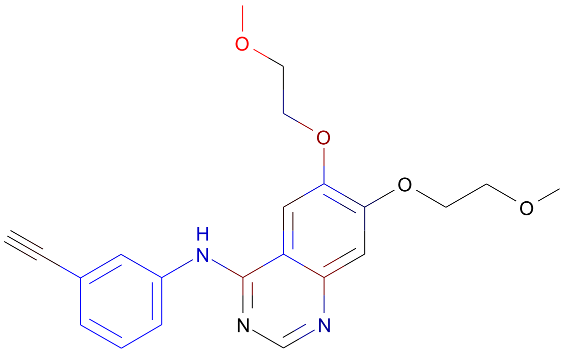

$C_{22}H_{23}N_{3}O_{4}$
Molecular Weight: 393.43572
ALogP: 4.309
Rotatable Bonds: 10
Acceptors: 7
Donors: 1

Model Prediction

Prediction: 8.06
Unit: mg/kg_body_weight/day
Mahalanobis Distance: 17.7
Mahalanobis Distance p-value: 3.99e-020

Mahalanobis Distance: The Mahalanobis distance (MD) is a generalization of the Euclidean distance that accounts for correlations among the X properties. It is calculated as the distance to the center of the training data. The larger the MD, the less trustworthy the prediction.
Mahalanobis Distance p-value: The p-value gives the fraction of training data with an MD greater than or equal to the one for the given sample, assuming normally distributed data. The smaller the p-value, the less trustworthy the prediciton. For highly non-normal X properties (e.g., fingerprints), the MD p-value is wildly inaccurate.

Structural Similar Compounds

Name	Loxtidine	C.I. direct brown 95	Omeprazole
Structure	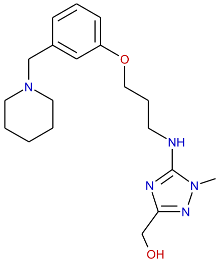	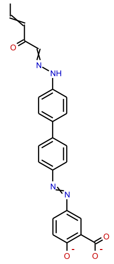	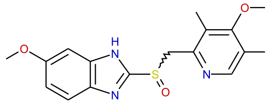
Actual Endpoint (-log C)	2.87532	5.31387	3.4628
Predicted Endpoint (-log C)	3.63996	4.30266	4.7324
Distance	0.685	0.715	0.741
Reference	CPDB	CPDB	CPDB

Model Applicability

Unknown features are fingerprint features in the query molecule, but not found or appearing too infrequently in the training set.

1. All properties and OPS components are within expected ranges.
2. Unknown FCFP_2 feature: 902193919: [*]:[c](:[*])C#C

Feature Contribution

Top features for positive contribution			
**Fingerprint**	**Bit/Smiles**	**Feature Structure**	**Score**
FCFP_6	136627117	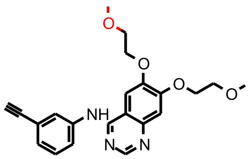 [*]OC	0.69

FCFP_6	1	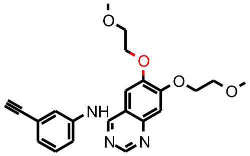 [*]S[*]	0.234
FCFP_6	203677720	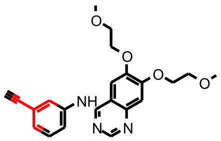 [*]S(=[*])(=[*])[c](: [cH]:[*]):[cH]:[*]	0.137
**Top Features for negative contribution**			
**Fingerprint**	**Bit/Smiles**	**Feature Structure**	**Score**
FCFP_6	991735244	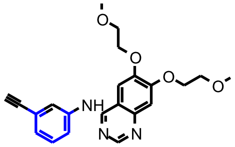 [*][c]1:[*]:[c]([*]): [cH]:[cH]:[cH]:1	-0.422
FCFP_6	16	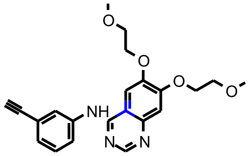 [*][c](:[*]):[*]	-0.354
FCFP_6	590925877	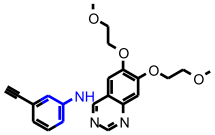 [*]N[c](:[cH]:[*]):[c H]:[*]	-0.323

acetazolamide.cdx

TOPKAT_Carcinogenic_Potency_TD50_Rat

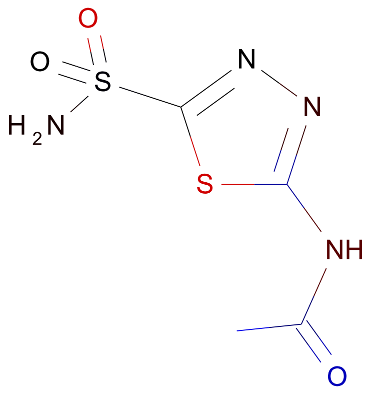

$C_4H_6N_4O_3S_2$
Molecular Weight: 222.24544
ALogP: -1.329
Rotatable Bonds: 2
Acceptors: 5
Donors: 2

Model Prediction

Prediction: 221
Unit: mg/kg_body_weight/day
Mahalanobis Distance: 11.9
Mahalanobis Distance p-value: 0.00077

Mahalanobis Distance: The Mahalanobis distance (MD) is a generalization of the Euclidean distance that accounts for correlations among the X properties. It is calculated as the distance to the center of the training data. The larger the MD, the less trustworthy the prediction.
Mahalanobis Distance p-value: The p-value gives the fraction of training data with an MD greater than or equal to the one for the given sample, assuming normally distributed data. The smaller the p-value, the less trustworthy the prediciton. For highly non-normal X properties (e.g., fingerprints), the MD p-value is wildly inaccurate.

Structural Similar Compounds

Name	Nithiazide	274	5-Nitro-2-furaldehyde semicarbazone
Structure	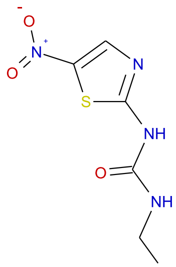	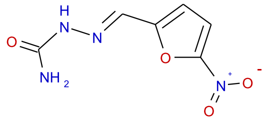	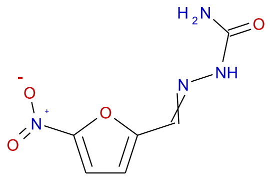
Actual Endpoint (-log C)	3.21762	4.45311	4.45311
Predicted Endpoint (-log C)	3.67171	3.27786	3.27786
Distance	0.569	0.591	0.591
Reference	CPDB	CPDB	CPDB

Model Applicability

Unknown features are fingerprint features in the query molecule, but not found or appearing too infrequently in the training set.

1. All properties and OPS components are within expected ranges.

Feature Contribution

Top features for positive contribution

Fingerprint	Bit/Smiles	Feature Structure	Score
FCFP_6	1	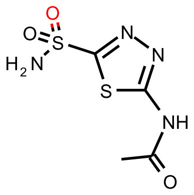 [*]S[*]	0.234

FCFP_6	-1151914249	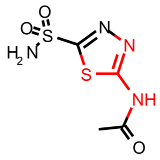 [*]N[c]1:n:[*]:[*]:s:1	0.204
FCFP_6	136597326	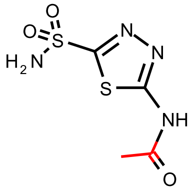 [*]C(=[*])C	0.0695
**Top Features for negative contribution**			
**Fingerprint**	**Bit/Smiles**	**Feature Structure**	**Score**
FCFP_6	16	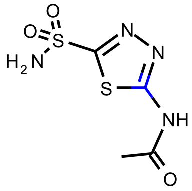 [*][c](:[*]):[*]	-0.354
FCFP_6	566058135	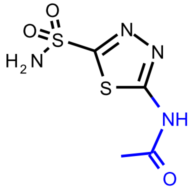 [*]NC(=O)C	-0.182
FCFP_6	17	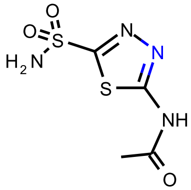 [*]:n:[*]	-0.149

Comp. 14

TOPKAT_Chronic_LOAEL

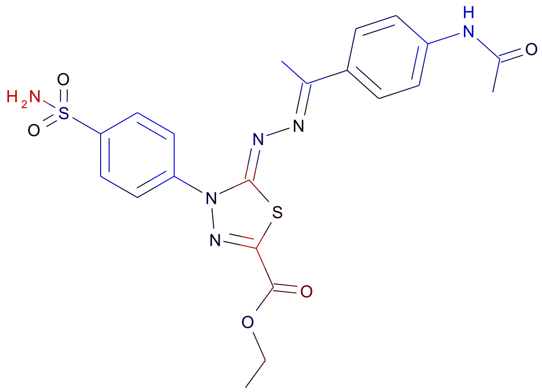

$C_{21}H_{22}N_6O_5S_2$

Molecular Weight: 502.56657

ALogP: 2.259

Rotatable Bonds: 8

Acceptors: 10

Donors: 2

Model Prediction

Prediction: 0.108

Unit: g/kg_body_weight

Mahalanobis Distance: 33.9

Mahalanobis Distance p-value: 9.26e-032

Mahalanobis Distance: The Mahalanobis distance (MD) is a generalization of the Euclidean distance that accounts for correlations among the X properties. It is calculated as the distance to the center of the training data. The larger the MD, the less trustworthy the prediction.
Mahalanobis Distance p-value: The p-value gives the fraction of training data with an MD greater than or equal to the one for the given sample, assuming normally distributed data. The smaller the p-value, the less trustworthy the prediciton. For highly non-normal X properties (e.g., fingerprints), the MD p-value is wildly inaccurate.

Structural Similar Compounds

Name	HARMONY	ALLY	C.I. ACID ORANGE 3
Structure	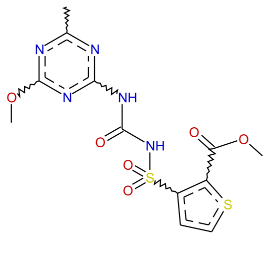	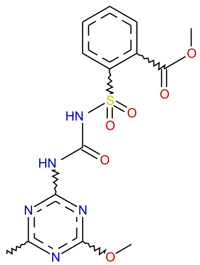	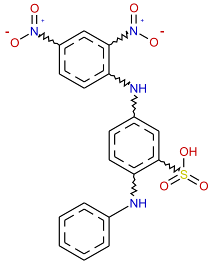
Actual Endpoint (-log C)	4.1902	3.1834	3.20573
Predicted Endpoint (-log C)	4.09361	3.59541	3.55956
Distance	0.640	0.666	0.687
Reference	EPA COVER SHEET 0337;881201;(1)	EPA COVER SHEET 0288;891101;(1)	NTP REPORT # 335

Model Applicability

Unknown features are fingerprint features in the query molecule, but not found or appearing too infrequently in the training set.

1. All properties and OPS components are within expected ranges.
2. Unknown ECFP_6 feature: 912478223: [*]S[*]
3. Unknown ECFP_6 feature: -797085356: [*]S(=[*])(=[*])[*]
4. Unknown ECFP_6 feature: 2122741631: [*]C1=[*][*]C(=[*])S1
5. Unknown ECFP_6 feature: -934225701: [*]C(=[*])C1=N[*][*]S1
6. Unknown ECFP_6 feature: 2092245922: [*]N1[*][*]C(=N1)[*]
7. Unknown ECFP_6 feature: -1110911409: [*]=C1[*][*]=NN1[c](:[*]):[*]
8. Unknown ECFP_6 feature: 189949281: [*]\N=C\1/S[*]=[*]N1[*]
9. Unknown ECFP_6 feature: 1430791942: [*]OC(=O)C(=[*])[*]
10. Unknown ECFP_6 feature: -175021654: [*]N([*])[c](:[cH]:[*]):[cH]:[*]
11. Unknown ECFP_6 feature: -177264675: [*]S(=[*])(=[*])[c](:[cH]:[*]):[cH]:[*]
12. Unknown ECFP_6 feature: -2121766239: [*]:[c](:[*])S(=O)(=O)N
13. Unknown ECFP_6 feature: 2102150379: [*]S(=[*])(=O)[*]
14. Unknown ECFP_6 feature: -934226723: [*]S(=[*])(=[*])N
15. Unknown ECFP_6 feature: -819426257: [*]C(=NN=[*])[*]
16. Unknown ECFP_6 feature: 562081661: [*]C(=NN=[*])[*]
17. Unknown ECFP_6 feature: 128986386: [*]\N=C(/C)\[c](:[*]):[*]
18. Unknown ECFP_6 feature: -175146122: [*]C(=[*])[c](:[cH]:[*]):[cH]:[*]
19. Unknown ECFP_6 feature: -177077903: [*]N[c](:[cH]:[*]):[cH]:[*]

20. Unknown ECFP_6 feature: -474544785: [*]NC(=O)C
21. Unknown ECFP_6 feature: -949601813: [*]OCC

Feature Contribution

Top features for positive contribution

Fingerprint	Bit/Smiles	Feature Structure	Score
FCFP_6	-1143715940	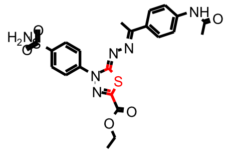 [*]C1=[*][*]C(=[*])S1	0.13
ECFP_6	1559650422	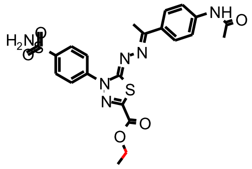 [*]C[*]	0.129
FCFP_6	3	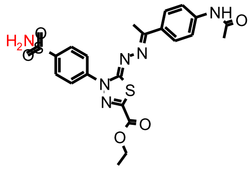 [*]N	0.0924

Top Features for negative contribution

Fingerprint	Bit/Smiles	Feature Structure	Score
FCFP_6	1	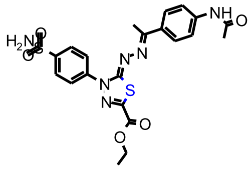 [*]S[*]	-0.102

ECFP_6	-1087070950	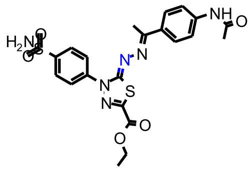 [*]N=[*]	-0.102
FCFP_6	-453677277	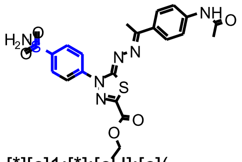 [*][c]1:[*]:[cH]:[c](:[cH]:[cH]:1)S(=[*])(=[*])[*]	-0.0906

Erlotinib

TOPKAT_Chronic_LOAEL

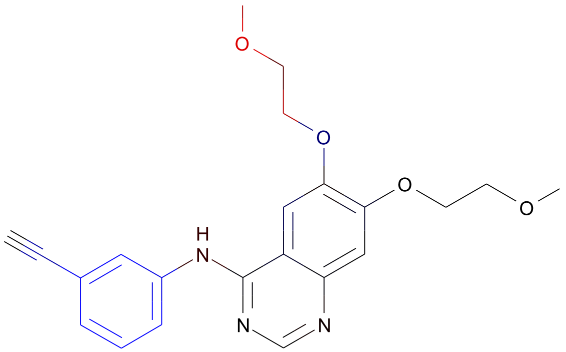

$C_{22}H_{23}N_3O_4$

Molecular Weight: 393.43572

ALogP: 4.309

Rotatable Bonds: 10

Acceptors: 7

Donors: 1

Model Prediction

Prediction: 0.0359

Unit: g/kg_body_weight

Mahalanobis Distance: 34.8

Mahalanobis Distance p-value: 2.91e-033

Mahalanobis Distance: The Mahalanobis distance (MD) is a generalization of the Euclidean distance that accounts for correlations among the X properties. It is calculated as the distance to the center of the training data. The larger the MD, the less trustworthy the prediction.

Mahalanobis Distance p-value: The p-value gives the fraction of training data with an MD greater than or equal to the one for the given sample, assuming normally distributed data. The smaller the p-value, the less trustworthy the prediciton. For highly non-normal X properties (e.g., fingerprints), the MD p-value is wildly inaccurate.

Structural Similar Compounds

Name	ASSURE	DILTIAZEM	RHODAMINE 6G
Structure	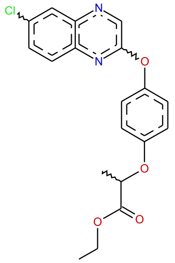	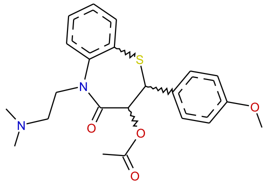	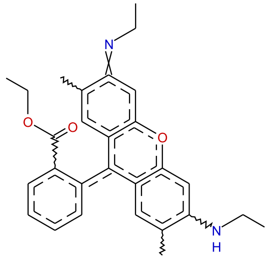
Actual Endpoint (-log C)	5.00328	4.21961	4.54906
Predicted Endpoint (-log C)	4.27671	4.005	4.6787
Distance	0.611	0.676	0.689
Reference	EPA COVER SHEET 0335;891001;(1)	NDA-18602	NTP 364 39

Model Applicability

Unknown features are fingerprint features in the query molecule, but not found or appearing too infrequently in the training set.

1. All properties and OPS components are within expected ranges.
2. Unknown FCFP_2 feature: 902193919: [*]:[c](:[*])C#C
3. Unknown FCFP_2 feature: 131784192: [*]C#C
4. Unknown ECFP_6 feature: -1114776580: [*]C#[*]
5. Unknown ECFP_6 feature: -1939823063: [*]#C
6. Unknown ECFP_6 feature: -1255706725: [*]CO[c](:[*]):[*]
7. Unknown ECFP_6 feature: -1253653003: [*]COC
8. Unknown ECFP_6 feature: -1242906247: [*]:[c](:[*])N[c](:[*]):[*]
9. Unknown ECFP_6 feature: -677309799: [*]:[cH]:n:[c](:[*]):[*]
10. Unknown ECFP_6 feature: 1333660716: [*][c](:[*]):[c](:[cH]:[*]):[c](:[*]):[*]
11. Unknown ECFP_6 feature: 1410041175: [*]:[cH]:[c](:n:[*]):[c](:[*]):[*]
12. Unknown ECFP_6 feature: 1049768340: [*]N[c](:n:[*]):[c](:[*]):[*]
13. Unknown ECFP_6 feature: -1790412586: [*]CCO[*]
14. Unknown ECFP_6 feature: -177077903: [*]N[c](:[cH]:[*]):[cH]:[*]
15. Unknown ECFP_6 feature: -710237522: [*]:n:[cH]:n:[*]
16. Unknown ECFP_6 feature: -182178874: [*]#C[c](:[cH]:[*]):[cH]:[*]
17. Unknown ECFP_6 feature: 1997021792: [*]:[cH]:[cH]:[cH]:[*]
18. Unknown ECFP_6 feature: 1139738044: [*]:[c](:[*])C#C
19. Unknown ECFP_6 feature: -1545539812: [*]C#C

Feature Contribution

Top features for positive contribution

Fingerprint	Bit/Smiles	Feature Structure	Score
FCFP_6	-1143715940	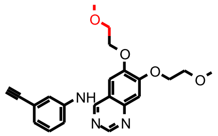 [*]C1=[*][*]C(=[*])S1	0.13
ECFP_6	1559650422	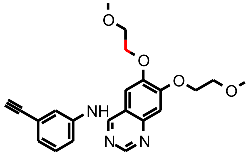 [*]C[*]	0.129
FCFP_6	3	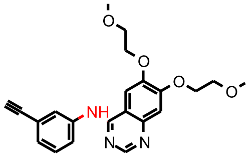 [*]N	0.0924

Top Features for negative contribution

Fingerprint	Bit/Smiles	Feature Structure	Score
FCFP_6	991735244	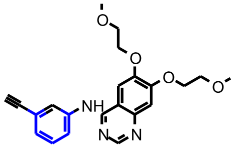 [*][c]1:[*]:[c]([*]):[cH]:[cH]:[cH]:1	-0.134

FCFP_6	1	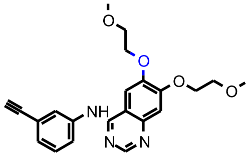 [*]S[*]	-0.102
FCFP_6	-453677277	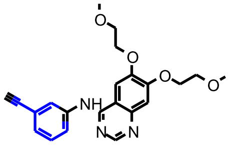 [*][c]1:[*]:[cH]:[c](:[cH]:[cH]:1)S(=[*]) (=[*])[*]	-0.0906

acetazolamide.cdx

TOPKAT_Chronic_LOAEL

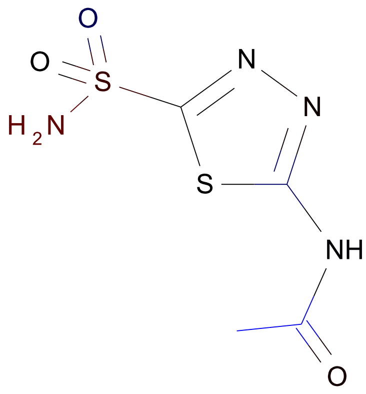

$C_4H_6N_4O_3S_2$

Molecular Weight: 222.24544

ALogP: -1.329

Rotatable Bonds: 2

Acceptors: 5

Donors: 2

Model Prediction

Prediction: 0.23

Unit: g/kg_body_weight

Mahalanobis Distance: 29.9

Mahalanobis Distance p-value: 1.56e-024

Mahalanobis Distance: The Mahalanobis distance (MD) is a generalization of the Euclidean distance that accounts for correlations among the X properties. It is calculated as the distance to the center of the training data. The larger the MD, the less trustworthy the prediction.
Mahalanobis Distance p-value: The p-value gives the fraction of training data with an MD greater than or equal to the one for the given sample, assuming normally distributed data. The smaller the p-value, the less trustworthy the prediciton. For highly non-normal X properties (e.g., fingerprints), the MD p-value is wildly inaccurate.

Structural Similar Compounds

Name	NITHIAZIDE	NITROFURAZONE	HYDROCHLOROTHIAZIDE
Structure	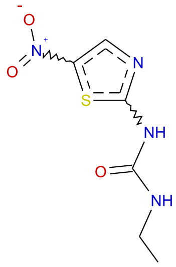	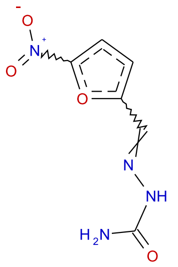	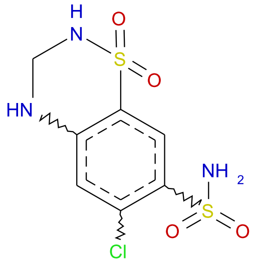
Actual Endpoint (-log C)	3.53901	4.10664	4.37691
Predicted Endpoint (-log C)	4.01443	4.00787	3.84479
Distance	0.549	0.572	0.577
Reference	NTP 146 22	NTP REPORT # 337	NTP REPORT # 357

Model Applicability

Unknown features are fingerprint features in the query molecule, but not found or appearing too infrequently in the training set.

1. All properties and OPS components are within expected ranges.
2. Unknown ECFP_6 feature: -797085356: [*]S(=[*])(=[*])[*]
3. Unknown ECFP_6 feature: 914325265: [*]:s:[*]
4. Unknown ECFP_6 feature: -474544785: [*]NC(=O)C
5. Unknown ECFP_6 feature: -1596132236: [*]N[c]1:n:[*]:[*]:s:1
6. Unknown ECFP_6 feature: 911256832: [*][c]1:[*]:[*]:n:n:1
7. Unknown ECFP_6 feature: 1221843808: [*]S(=[*])(=[*])[c]1:n:[*]:[*]:s:1
8. Unknown ECFP_6 feature: -2121766239: [*]:[c](:[*])S(=O)(=O)N
9. Unknown ECFP_6 feature: -934226723: [*]S(=[*])(=[*])N
10. Unknown ECFP_6 feature: 2102150379: [*]S(=[*])(=O)[*]
11. Unknown ECFP_6 feature: 85262808: [*][c]1:[*]:[*]:[c]([*]):s:1

Feature Contribution

Top features for positive contribution			
Fingerprint	Bit/Smiles	Feature Structure	Score

FCFP_6	3	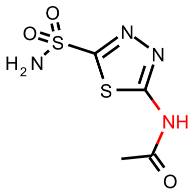 [*]N	0.0924
ECFP_6	2099970318	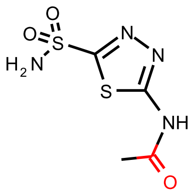 [*]C(=O)[*]	0.0766
ECFP_6	1572579716	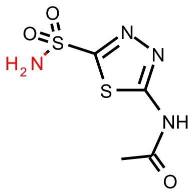 [*]N	0.0576
**Top Features for negative contribution**			
**Fingerprint**	**Bit/Smiles**	**Feature Structure**	**Score**
FCFP_6	1	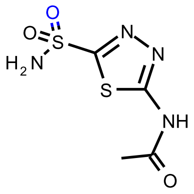 [*]S[*]	-0.102
FCFP_6	136597326	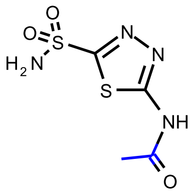 [*]C(=[*])C	-0.0815

ECFP_6	-1236483485	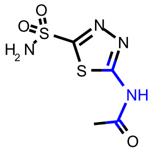 [*]C(=[*])N[c](:[*]):[*]	-0.0747

Comp. 14

TOPKAT_Rat_Maximum_Tolerated_Dose_Feed

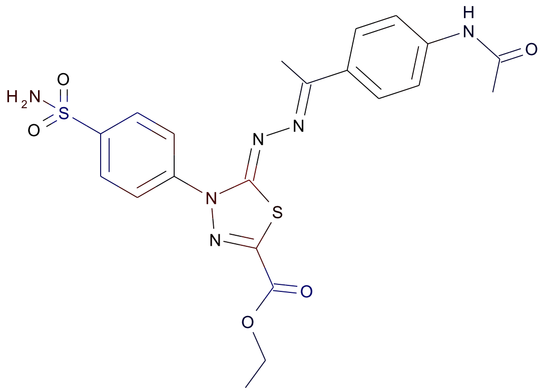

$C_{21}H_{22}N_6O_5S_2$
Molecular Weight: 502.56657
ALogP: 2.259
Rotatable Bonds: 8
Acceptors: 10
Donors: 2

Structural Similar Compounds

Name	SALICYLAZOSULFAPYRIDINE	4,4'-DIAMINO-2,2'-STILBENEDISULFONIC ACID.2NaSALT	C.I.PIGMENT RED 23
Structure	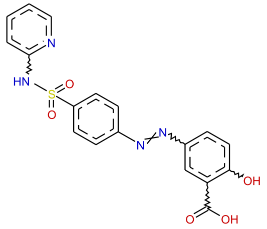	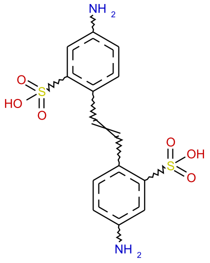	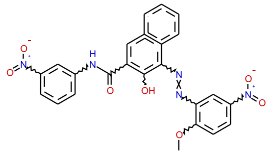
Actual Endpoint (-log C)	3.375	2.50759	2.30052
Predicted Endpoint (-log C)	2.80292	3.26068	3.55333
Distance	0.767	0.787	0.853
Reference	NCI/NTP TR-457	NCI/NTP TR-412	NCI/NTP TR-411

Model Prediction

Prediction: 0.0455
Unit: g/kg_body_weight
Mahalanobis Distance: 11.5
Mahalanobis Distance p-value: 9.03e-008

Mahalanobis Distance: The Mahalanobis distance (MD) is a generalization of the Euclidean distance that accounts for correlations among the X properties. It is calculated as the distance to the center of the training data. The larger the MD, the less trustworthy the prediction.
Mahalanobis Distance p-value: The p-value gives the fraction of training data with an MD greater than or equal to the one for the given sample, assuming normally distributed data. The smaller the p-value, the less trustworthy the prediciton. For highly non-normal X properties (e.g., fingerprints), the MD p-value is wildly inaccurate.

Model Applicability

Unknown features are fingerprint features in the query molecule, but not found or appearing too infrequently in the training set.

1. All properties and OPS components are within expected ranges.

Feature Contribution

Top features for positive contribution

Fingerprint	Bit/Smiles	Feature Structure	Score
FCFP_2	-1143715940	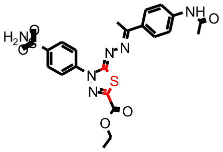 [*]C1=[*][*]C(=[*])S1	0.095

FCFP_2	3	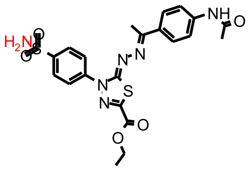 [*]N	0.0737
FCFP_2	332760439	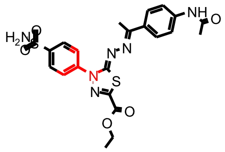 [*]N([*])[c](:[cH]:[*]):[cH]:[*]	0.0611
**Top Features for negative contribution**			
**Fingerprint**	**Bit/Smiles**	**Feature Structure**	**Score**
FCFP_2	1872154524	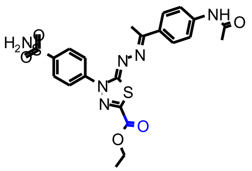 [*]C(=O)[*]	-0.105
FCFP_2	203677720	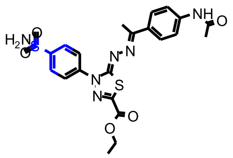 [*]S(=[*])(=[*])[c](: [cH]:[*]):[cH]:[*]	-0.0829
FCFP_2	1	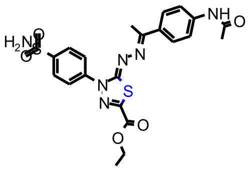 [*]S[*]	-0.0796

Erlotinib

TOPKAT_Rat_Maximum_Tolerated_Dose_Feed

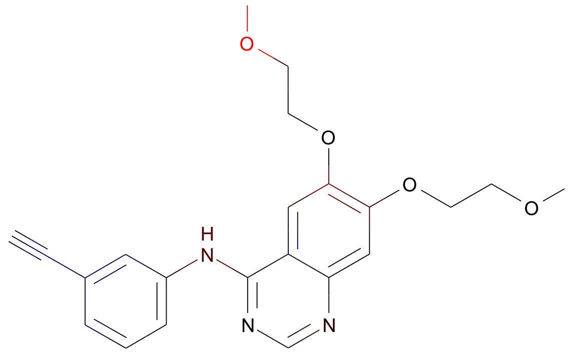

$C_{22}H_{23}N_3O_4$

Molecular Weight: 393.43572

ALogP: 4.309

Rotatable Bonds: 10

Acceptors: 7

Donors: 1

Structural Similar Compounds

Name	BUTYL BENZYL PHTHALATE	3,3'-DIMETHOXYBENZIDINE-4,4'-DIISOCYANATE	PYRILAMINE
Structure	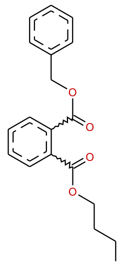	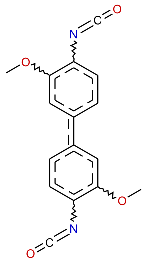	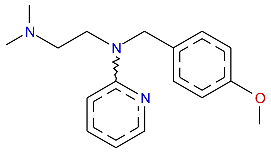
Actual Endpoint (-log C)	2.79569	2.17504	3.32511
Predicted Endpoint (-log C)	3.18498	3.78717	3.65163
Distance	0.758	0.795	0.820
Reference	NCI/NTP TR-458	NCI/NTP TR-128	NCI/NTP Report 10, Nov. 1987

Model Prediction

Prediction: 0.0828

Unit: g/kg_body_weight

Mahalanobis Distance: 9.37

Mahalanobis Distance p-value: 0.000417

Mahalanobis Distance: The Mahalanobis distance (MD) is a generalization of the Euclidean distance that accounts for correlations among the X properties. It is calculated as the distance to the center of the training data. The larger the MD, the less trustworthy the prediction.
Mahalanobis Distance p-value: The p-value gives the fraction of training data with an MD greater than or equal to the one for the given sample, assuming normally distributed data. The smaller the p-value, the less trustworthy the prediciton. For highly non-normal X properties (e.g., fingerprints), the MD p-value is wildly inaccurate.

Model Applicability

Unknown features are fingerprint features in the query molecule, but not found or appearing too infrequently in the training set.

1. All properties and OPS components are within expected ranges.
2. Unknown FCFP_2 feature: -124685461: [*]:n:c:n:[*]
3. Unknown FCFP_2 feature: 902193919: [*]:[c](:[*])C#C
4. Unknown FCFP_2 feature: 131784192: [*]C#C

Feature Contribution

Top features for positive contribution			
**Fingerprint**	**Bit/Smiles**	**Feature Structure**	**Score**
FCFP_2	136627117	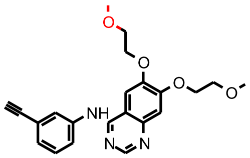 [*]OC	0.173

FCFP_2	-1143715940	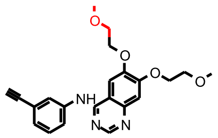<br>[*]C1=[*][*]C(=[*])S1	0.095
FCFP_2	1036089772	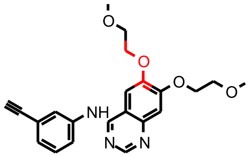<br>[*]CO[c](:[*]):[*]	0.0749
**Top Features for negative contribution**			
**Fingerprint**	**Bit/Smiles**	**Feature Structure**	**Score**
FCFP_2	203677720	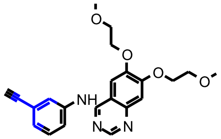<br>[*]S(=[*])(=[*])[c](:<br>[cH]:[*]):[cH]:[*]	-0.0829
FCFP_2	1	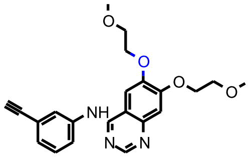<br>[*]S[*]	-0.0796
FCFP_2	16	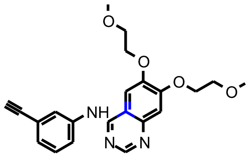<br>[*][c](:[*]):[*]	-0.0512

acetazolamide.cdx

TOPKAT_Rat_Maximum_Tolerated_Dose_Feed

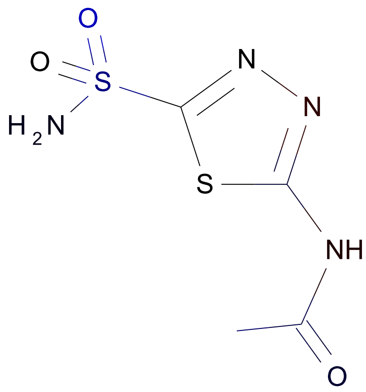

$C_4H_6N_4O_3S_2$

Molecular Weight: 222.24544

ALogP: -1.329

Rotatable Bonds: 2

Acceptors: 5

Donors: 2

Model Prediction

Prediction: 0.0522

Unit: g/kg_body_weight

Mahalanobis Distance: 9.06

Mahalanobis Distance p-value: 0.00116

Mahalanobis Distance: The Mahalanobis distance (MD) is a generalization of the Euclidean distance that accounts for correlations among the X properties. It is calculated as the distance to the center of the training data. The larger the MD, the less trustworthy the prediction.
Mahalanobis Distance p-value: The p-value gives the fraction of training data with an MD greater than or equal to the one for the given sample, assuming normally distributed data. The smaller the p-value, the less trustworthy the prediciton. For highly non-normal X properties (e.g., fingerprints), the MD p-value is wildly inaccurate.

Structural Similar Compounds

Name	NITHIAZIDE	NITROFURAZONE	HYDROCHLOROTHIAZIDE
Structure	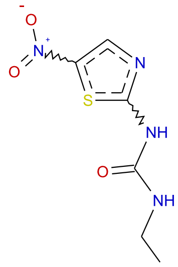	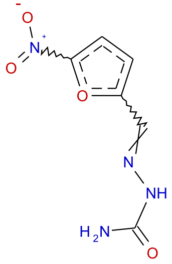	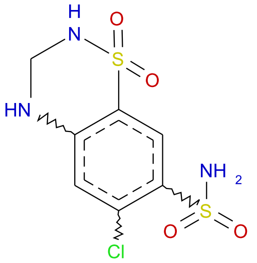
Actual Endpoint (-log C)	3.58476	4.21779	3.56001
Predicted Endpoint (-log C)	3.58855	3.40885	3.55045
Distance	0.500	0.549	0.563
Reference	NCI/NTP TR-146	NCI/NTP TR-337	NCI/NTP TR-357

Model Applicability

Unknown features are fingerprint features in the query molecule, but not found or appearing too infrequently in the training set.

1. All properties and OPS components are within expected ranges.

Feature Contribution

Top features for positive contribution

Fingerprint	Bit/Smiles	Feature Structure	Score
FCFP_2	3	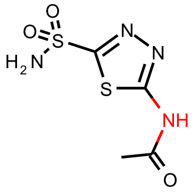 [*]N	0.0737

FCFP_2	17	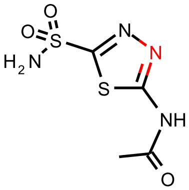 [*]:n:[*]	0.0441
FCFP_2	1294255210	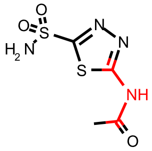 [*]C(=[*])N[c](:[*]):[*]	0.00319

Top Features for negative contribution

Fingerprint	Bit/Smiles	Feature Structure	Score
FCFP_2	1872154524	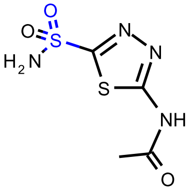 [*]C(=O)[*]	-0.105
FCFP_2	1	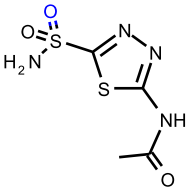 [*]S[*]	-0.0796
FCFP_2	16	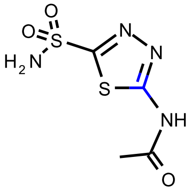 [*][c](:[*]):[*]	-0.0512

Comp. 14

TOPKAT_Rat_Maximum_Tolerated_Dose_Gavage

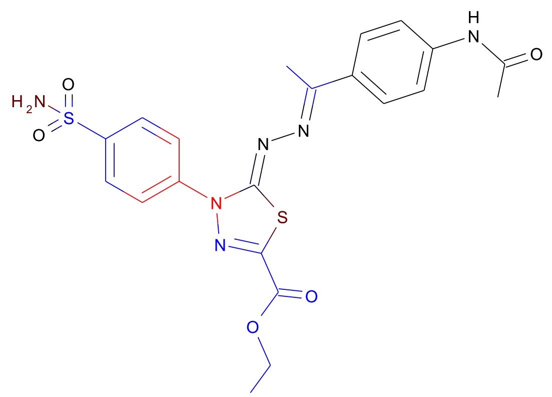

$C_{21}H_{22}N_6O_5S_2$

Molecular Weight: 502.56657

ALogP: 2.259

Rotatable Bonds: 8

Acceptors: 10

Donors: 2

Model Prediction

Prediction: 0.00189

Unit: g/kg_body_weight

Mahalanobis Distance: 11.5

Mahalanobis Distance p-value: 4.44e-008

Mahalanobis Distance: The Mahalanobis distance (MD) is a generalization of the Euclidean distance that accounts for correlations among the X properties. It is calculated as the distance to the center of the training data. The larger the MD, the less trustworthy the prediction.
Mahalanobis Distance p-value: The p-value gives the fraction of training data with an MD greater than or equal to the one for the given sample, assuming normally distributed data. The smaller the p-value, the less trustworthy the prediciton. For highly non-normal X properties (e.g., fingerprints), the MD p-value is wildly inaccurate.

Structural Similar Compounds

Name	PENICILLIN VK	OCHRATOXIN	AMPICILLIN TRIHYDRATE
Structure	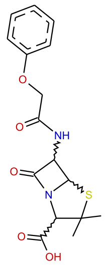	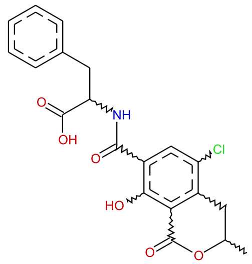	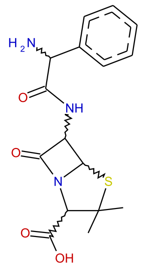
Actual Endpoint (-log C)	2.54455	6.28396	2.36724
Predicted Endpoint (-log C)	3.9702	5.12358	2.27651
Distance	1.099	1.119	1.204
Reference	NCI/NTP TR-336	NCI/NTP TR-358	NCI/NTP TR-318

Model Applicability

Unknown features are fingerprint features in the query molecule, but not found or appearing too infrequently in the training set.

1. Molecular_Weight out of range. Value: 502.57. Training min, max, mean, SD: 68.074, 434.63, 171.13, 85.06.
2. Num_H_Acceptors out of range. Value: 10. Training min, max, mean, SD: 0, 6, 1.6146, 1.644.
3. Molecular_PolarSASA out of range. Value: 261.53. Training min, max, mean, SD: 0, 223.97, 50.816, 55.15.
4. Molecular_PolarSurfaceArea out of range. Value: 189.56. Training min, max, mean, SD: 0, 138.03, 28.978, 32.1.
5. OPS PC1 out of range. Value: 8.9568. Training min, max, SD, explained variance: -4.0008, 7.9165, 2.861, 0.2531.
6. OPS PC6 out of range. Value: -2.6802. Training min, max, SD, explained variance: -2.4321, 2.9885, 1.256, 0.0488.
7. Unknown FCFP_2 feature: 580960234: [*]N1[*][*]C(=N1)[*]
8. Unknown FCFP_2 feature: -1986158408: [*]\N=C\1/S[*]=[*]N1[*]
9. Unknown FCFP_2 feature: -1549192822: [*]\N=C(/C)\[c](:[*]):[*]

Feature Contribution

Top features for positive contribution			
**Fingerprint**	**Bit/Smiles**	**Feature Structure**	**Score**

FCFP_2	332760439	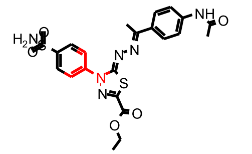 [*]N([*])[c](:[cH]:[*]):[cH]:[*]	0.672
FCFP_2	1	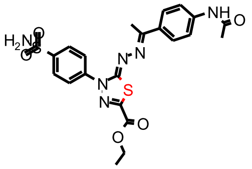 [*]S[*]	0.511
FCFP_2	3	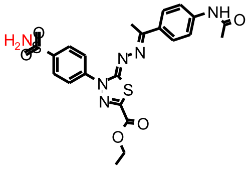 [*]N	0.104

Top Features for negative contribution			
**Fingerprint**	**Bit/Smiles**	**Feature Structure**	**Score**
FCFP_2	136597326	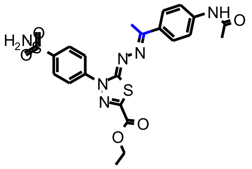 [*]C(=[*])C	-0.489
FCFP_2	203677720	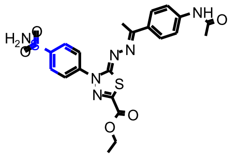 [*]S(=[*])(=[*])[c](:[cH]:[*]):[cH]:[*]	-0.406

FCFP_2	565998553	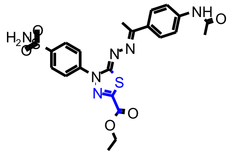 [*]C(=[*])C1=N[*][*]S1	-0.348

Erlotinib

TOPKAT_Rat_Maximum_Tolerated_Dose_Gavage

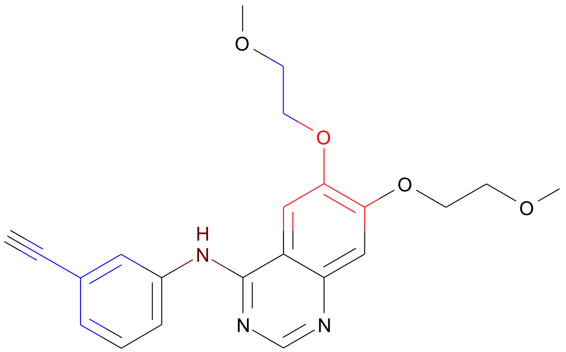

$C_{22}H_{23}N_3O_4$

Molecular Weight: 393.43572

ALogP: 4.309

Rotatable Bonds: 10

Acceptors: 7

Donors: 1

Model Prediction

Prediction: 0.000344

Unit: g/kg_body_weight

Mahalanobis Distance: 11.7

Mahalanobis Distance p-value: 2.74e-008

Mahalanobis Distance: The Mahalanobis distance (MD) is a generalization of the Euclidean distance that accounts for correlations among the X properties. It is calculated as the distance to the center of the training data. The larger the MD, the less trustworthy the prediction.
Mahalanobis Distance p-value: The p-value gives the fraction of training data with an MD greater than or equal to the one for the given sample, assuming normally distributed data. The smaller the p-value, the less trustworthy the prediciton. For highly non-normal X properties (e.g., fingerprints), the MD p-value is wildly inaccurate.

Structural Similar Compounds

Name	DIALLYL PHTHALATE	OCHRATOXIN	PROBENECID
Structure	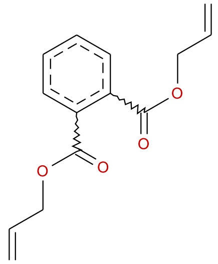	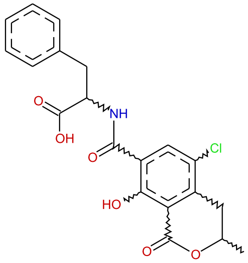	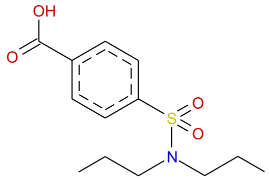
Actual Endpoint (-log C)	3.3914	6.28396	2.85333
Predicted Endpoint (-log C)	3.50093	5.12358	2.4258
Distance	1.113	1.136	1.152
Reference	NCI/NTP TR-284	NCI/NTP TR-358	NCI/NTP TR-395

Model Applicability

Unknown features are fingerprint features in the query molecule, but not found or appearing too infrequently in the training set.

1. Num_H_Acceptors out of range. Value: 7. Training min, max, mean, SD: 0, 6, 1.6146, 1.644.
2. Num_AromaticRings out of range. Value: 3. Training min, max, mean, SD: 0, 2, 0.5625, 0.693.
3. OPS PC6 out of range. Value: -3.0997. Training min, max, SD, explained variance: -2.4321, 2.9885, 1.256, 0.0488.
4. Unknown FCFP_2 feature: 1293778554: [*]:[c](:[*])N[c](:[*]):[*]
5. Unknown FCFP_2 feature: -124685461: [*]:n:c:n:[*]
6. Unknown FCFP_2 feature: 902193919: [*]:[c](:[*])C#C
7. Unknown FCFP_2 feature: 131784192: [*]C#C

Feature Contribution

Top features for positive contribution

Fingerprint	Bit/Smiles	Feature Structure	Score

FCFP_2	332760439	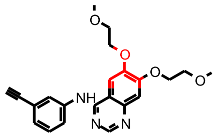<br>[*]N([*])[c](:[cH]:[*]):[cH]:[*]	0.672
FCFP_2	1	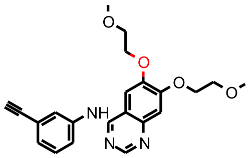<br>[*]S[*]	0.511
FCFP_2	3	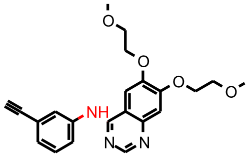<br>[*]N	0.104

Top Features for negative contribution

Fingerprint	Bit/Smiles	Feature Structure	Score
FCFP_2	203677720	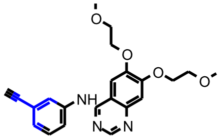<br>[*]S(=[*])(=[*])[c](:[cH]:[*]):[cH]:[*]	-0.406
FCFP_2	0	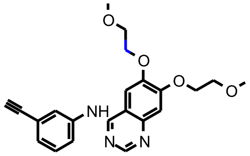<br>[*]C(=[*])[*]	-0.29

FCFP_2	-1272768868	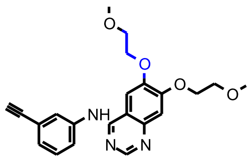 [*]OCC	-0.271

acetazolamide.cdx

TOPKAT_Rat_Maximum_Tolerated_Dose_Gavage

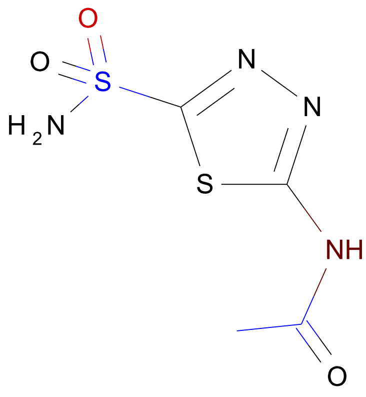

$C_4H_6N_4O_3S_2$

Molecular Weight: 222.24544

ALogP: -1.329

Rotatable Bonds: 2

Acceptors: 5

Donors: 2

Model Prediction

Prediction: 0.485

Unit: g/kg_body_weight

Mahalanobis Distance: 9.68

Mahalanobis Distance p-value: 1.54e-005

Mahalanobis Distance: The Mahalanobis distance (MD) is a generalization of the Euclidean distance that accounts for correlations among the X properties. It is calculated as the distance to the center of the training data. The larger the MD, the less trustworthy the prediction.
Mahalanobis Distance p-value: The p-value gives the fraction of training data with an MD greater than or equal to the one for the given sample, assuming normally distributed data. The smaller the p-value, the less trustworthy the prediciton. For highly non-normal X properties (e.g., fingerprints), the MD p-value is wildly inaccurate.

Structural Similar Compounds

Name	AMPICILLIN TRIHYDRATE	HC RED 3	SULFISOOXAZOLE
Structure	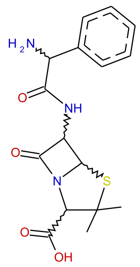	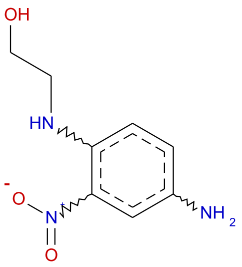	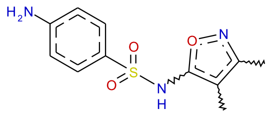
Actual Endpoint (-log C)	2.36724	2.59592	2.82494
Predicted Endpoint (-log C)	2.27651	3.285	3.0705
Distance	0.771	0.776	0.804
Reference	NCI/NTP TR-318	NCI/NTP TR-281	NCI/NTP TR-138

Model Applicability

Unknown features are fingerprint features in the query molecule, but not found or appearing too infrequently in the training set.

1. Molecular_PolarSASA out of range. Value: 248.68. Training min, max, mean, SD: 0, 223.97, 50.816, 55.15.
2. Molecular_PolarSurfaceArea out of range. Value: 151.66. Training min, max, mean, SD: 0, 138.03, 28.978, 32.1.
3. Unknown FCFP_2 feature: -1151914249: [*]N[c]1:n:[*]:[*]:s:1
4. Unknown FCFP_2 feature: -1539162406: [*]S(=[*])(=[*])[c]1:n:[*]:[*]:s:1

Feature Contribution

Top features for positive contribution

Fingerprint	Bit/Smiles	Feature Structure	Score
FCFP_2	1	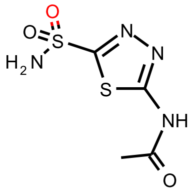 [*]S[*]	0.511

FCFP_2	3	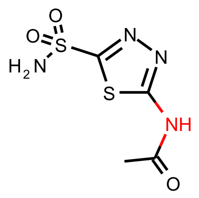 [*]N	0.104
**Top Features for negative contribution**			
**Fingerprint**	**Bit/Smiles**	**Feature Structure**	**Score**
FCFP_2	136597326	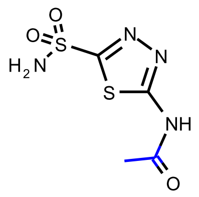 [*]C(=[*])C	-0.489
FCFP_2	1872154524	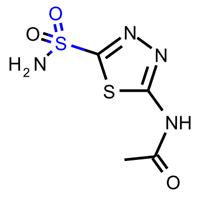 [*]C(=O)[*]	-0.307
FCFP_2	0	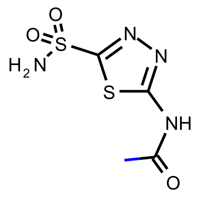 [*]C(=[*])[*]	-0.29

Comp. 14 **TOPKAT_Rat_Oral_LD50**

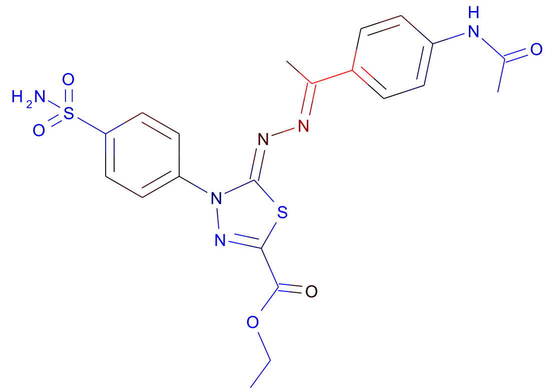

$C_{21}H_{22}N_6O_5S_2$
Molecular Weight: 502.56657
ALogP: 2.259
Rotatable Bonds: 8
Acceptors: 10
Donors: 2

Structural Similar Compounds

Name	LENAMPICILLIN .HCl (HCl STRIPPED)	4-(4-AMINO-3-SULFOPHENYLAZO)BENZENESULFONIC ACID	CEPHAPIRIN
Structure	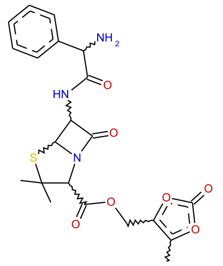	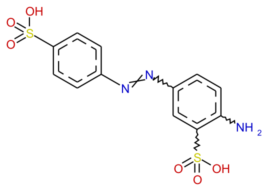	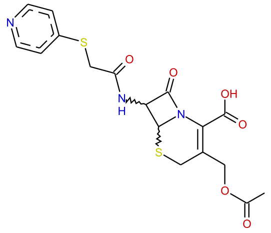
Actual Endpoint (-log C)	1.664	1.383	1.413
Predicted Endpoint (-log C)	2.37593	2.20116	2.17753
Distance	0.703	0.760	0.768
Reference	NKRZAZ 32(Suppl	28ZPAK -;192;72	TOIZAG 21;279;74

Model Prediction

Prediction: 15.8
Unit: g/kg_body_weight
Mahalanobis Distance: 28
Mahalanobis Distance p-value: 9.61e-050

Mahalanobis Distance: The Mahalanobis distance (MD) is a generalization of the Euclidean distance that accounts for correlations among the X properties. It is calculated as the distance to the center of the training data. The larger the MD, the less trustworthy the prediction.
Mahalanobis Distance p-value: The p-value gives the fraction of training data with an MD greater than or equal to the one for the given sample, assuming normally distributed data. The smaller the p-value, the less trustworthy the prediciton. For highly non-normal X properties (e.g., fingerprints), the MD p-value is wildly inaccurate.

Model Applicability

Unknown features are fingerprint features in the query molecule, but not found or appearing too infrequently in the training set.

1. All properties and OPS components are within expected ranges.
2. Unknown ECFP_2 feature: -934225701: [*]C(=[*])C1=N[*][*]S1
3. Unknown ECFP_2 feature: 189949281: [*]\N=C\1/S[*]=[*]N1[*]
4. Unknown ECFP_2 feature: -819426257: [*]C(=NN=[*])[*]
5. Unknown ECFP_2 feature: 562081661: [*]C(=NN=[*])[*]
6. Unknown ECFP_2 feature: 128986386: [*]\N=C(/C)\[c](:[*]):[*]
7. Unknown FCFP_6 feature: 16: [*][c](:[*]):[*]
8. Unknown FCFP_6 feature: 580960234: [*]N1[*][*]C(=N1)[*]
9. Unknown FCFP_6 feature: 675799546: [*]=C1[*][*]=NN1[c](:[*]):[*]
10. Unknown FCFP_6 feature: 1618154665: [*][c](:[*]):[cH]:[cH]:[*]

Feature Contribution

Top features for positive contribution			
**Fingerprint**	**Bit/Smiles**	**Feature Structure**	**Score**

ECFP_6	642810091	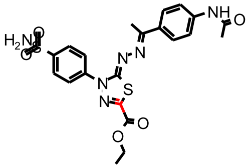 [*]C(=[*])[*]	0.281
ECFP_6	-1897341097	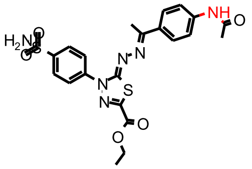 [*]N[*]	0.216
FCFP_6	-1549192822	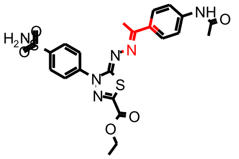 [*]\N=C(/C)\[c](:[*]) :[*]	0.168

Top Features for negative contribution

**Fingerprint**	**Bit/Smiles**	**Feature Structure**	**Score**
ECFP_6	1887306650	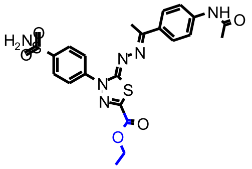 [*]C(=[*])OCC	-0.271
ECFP_6	655739385	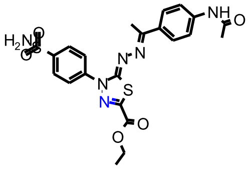 [*]N=[*]	-0.239

FCFP_6	-1096219292	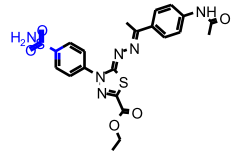 [*]:[c](:[*])S(=O)(=O)N	-0.225

Erlotinib

TOPKAT_Rat_Oral_LD50

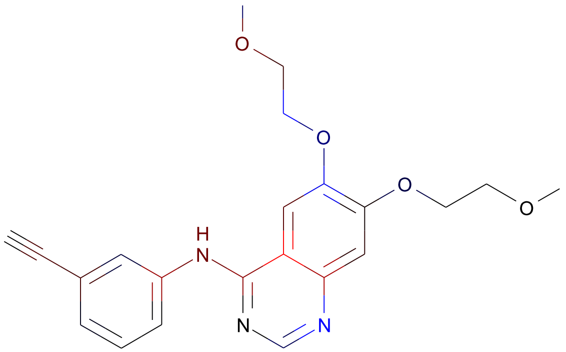

$C_{22}H_{23}N_3O_4$
Molecular Weight: 393.43572
ALogP: 4.309
Rotatable Bonds: 10
Acceptors: 7
Donors: 1

Structural Similar Compounds

Name	TALNIFLUMATE	3-QUINOLINECARBOXYLIC ACID; 6;7-bis-(CYCLOPROPYLMETHOXY)-4-HYDROXY-; ETHYL ESTER	DIXYRAZINE .HCl (HCl STRIPPED)
Structure	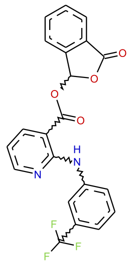	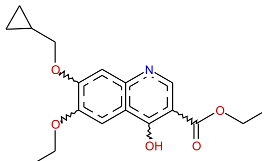	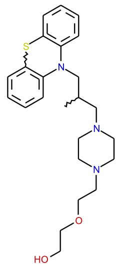
Actual Endpoint (-log C)	1.538	2.076	3.029
Predicted Endpoint (-log C)	2.82541	2.50101	2.47585
Distance	0.615	0.615	0.654
Reference	FRPSAX 36;372;81	TXAPA9 18;185;71	ANPBAZ 61;669;61

Model Prediction

Prediction: 0.662
Unit: g/kg_body_weight
Mahalanobis Distance: 20.8
Mahalanobis Distance p-value: 9.57e-012

Mahalanobis Distance: The Mahalanobis distance (MD) is a generalization of the Euclidean distance that accounts for correlations among the X properties. It is calculated as the distance to the center of the training data. The larger the MD, the less trustworthy the prediction.
Mahalanobis Distance p-value: The p-value gives the fraction of training data with an MD greater than or equal to the one for the given sample, assuming normally distributed data. The smaller the p-value, the less trustworthy the prediciton. For highly non-normal X properties (e.g., fingerprints), the MD p-value is wildly inaccurate.

Model Applicability

Unknown features are fingerprint features in the query molecule, but not found or appearing too infrequently in the training set.

1. All properties and OPS components are within expected ranges.
2. Unknown ECFP_2 feature: 1139738044: [*]:[c](:[*])C#C
3. Unknown FCFP_6 feature: 16: [*][c](:[*]):[*]
4. Unknown FCFP_6 feature: 1293778554: [*]:[c](:[*])N[c](:[*]):[*]
5. Unknown FCFP_6 feature: 1747237384: [*]:[cH]:n:[c](:[*]):[*]
6. Unknown FCFP_6 feature: 1618154665: [*][c](:[*]):[cH]:[cH]:[*]
7. Unknown FCFP_6 feature: -1151884458: [*]N[c](:n:[*]):[c](:[*]):[*]
8. Unknown FCFP_6 feature: -124685461: [*]:n:[cH]:n:[*]
9. Unknown FCFP_6 feature: 902193919: [*]:[c](:[*])C#C
10. Unknown FCFP_6 feature: 131784192: [*]C#C

Feature Contribution

Top features for positive contribution			
**Fingerprint**	**Bit/Smiles**	**Feature Structure**	**Score**

ECFP_6	642810091	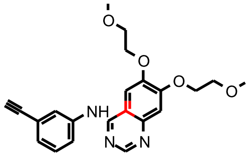 [*]C(=[*])[*]	0.281
ECFP_6	-1897341097	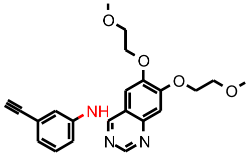 [*]N[*]	0.216
FCFP_6	136627117	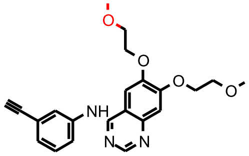 [*]OC	0.17

Top Features for negative contribution

Fingerprint	Bit/Smiles	Feature Structure	Score
ECFP_6	655739385	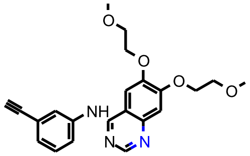 [*]N=[*]	-0.239
ECFP_6	734603939	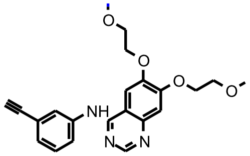 [*]C	-0.201

FCFP_6	1036089772	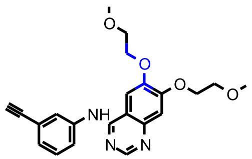 [*]CO[c](:[*]):[*]	-0.136

acetazolamide.cdx

TOPKAT_Rat_Oral_LD50

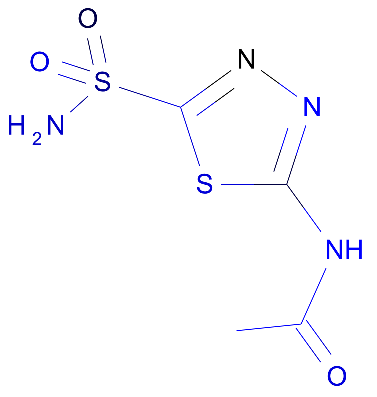

$C_4H_6N_4O_3S_2$

Molecular Weight: 222.24544

ALogP: -1.329

Rotatable Bonds: 2

Acceptors: 5

Donors: 2

Model Prediction

Prediction: 17.5

Unit: g/kg_body_weight

Mahalanobis Distance: 20.2

Mahalanobis Distance p-value: 1.15e-009

Mahalanobis Distance: The Mahalanobis distance (MD) is a generalization of the Euclidean distance that accounts for correlations among the X properties. It is calculated as the distance to the center of the training data. The larger the MD, the less trustworthy the prediction.

Mahalanobis Distance p-value: The p-value gives the fraction of training data with an MD greater than or equal to the one for the given sample, assuming normally distributed data. The smaller the p-value, the less trustworthy the prediciton. For highly non-normal X properties (e.g., fingerprints), the MD p-value is wildly inaccurate.

Structural Similar Compounds

Name	m-BENZENEDISULFONAMIDE; 4-CHLORO-	CHLOROTHIAZIDE	SULFAMETHIZOLE
Structure	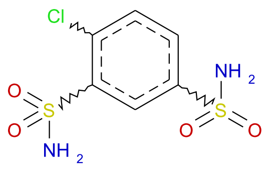	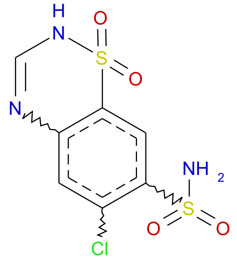	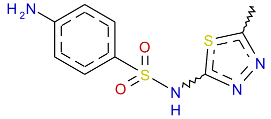
Actual Endpoint (-log C)	2.901	1.471	1.687
Predicted Endpoint (-log C)	1.8311	1.63932	1.85826
Distance	0.550	0.565	0.578
Reference	WRPCA2 9;119;70	YAKUD5 21;775;79	NIIRDN 6;388;82

Model Applicability

Unknown features are fingerprint features in the query molecule, but not found or appearing too infrequently in the training set.

1. All properties and OPS components are within expected ranges.
2. Unknown ECFP_2 feature: 1221843808: [*]S(=[*])(=[*])[c]1:n:[*]:[*]:s:1
3. Unknown FCFP_6 feature: 16: [*][c](:[*]):[*]
4. Unknown FCFP_6 feature: 4427049: [*][c]1:[*]:[*]:n:n:1
5. Unknown FCFP_6 feature: -1539162406: [*]S(=[*])(=[*])[c]1:n:[*]:[*]:s:1
6. Unknown FCFP_6 feature: 1747237384: [*]:[cH]:n:[c](:[*]):[*]

Feature Contribution

Top features for positive contribution

Fingerprint	Bit/Smiles	Feature Structure	Score

ECFP_6	642810091	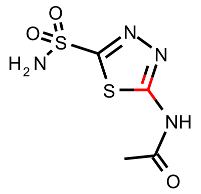 [*]C(=[*])[*]	0.281
ECFP_6	-1897341097	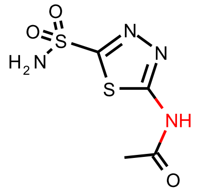 [*]N[*]	0.216
ECFP_6	-1074141656	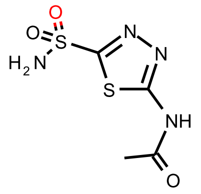 [*]=O	0.142
**Top Features for negative contribution**			
**Fingerprint**	**Bit/Smiles**	**Feature Structure**	**Score**
ECFP_6	655739385	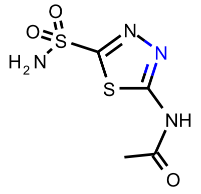 [*]N=[*]	-0.239
FCFP_6	-1096219292	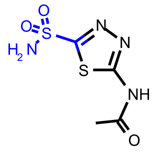 [*]:[c](:[*])S(=O)(=O)N	-0.225

FCFP_6	566058135	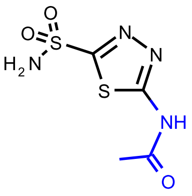 [*]NC(=O)C	-0.216
